# On the data privacy practices of Android OEMs

**DOI:** 10.1371/journal.pone.0279942

**Published:** 2023-01-18

**Authors:** Haoyu Liu, Paul Patras, Douglas J. Leith

**Affiliations:** 1 Department of Informatics, University of Edinburgh, Edinburgh, Scotland, United Kingdom; 2 School of Computer Science and Statistics, Trinity College Dublin, Dublin, Ireland; Universiti Tun Hussein Onn Malaysia, MALAYSIA

## Abstract

In this paper we present the first in-depth measurement study looking at the data privacy practices of the proprietary variants of the Android OS produced by Samsung, Xiaomi, Huawei and Realme. We address two questions: how are identifiers used in network connections and what types of data are transmitted. To answer these, we decrypt and decode the network traffic transmitted by a range of Android handsets. We find that all of the OEMs make undue use of long-lived hardware identifiers such as the hardware serial number, handset IMEI and so fail to follow best privacy practice. Hardware identifiers are also linked to the handset user’s real identity when they sign in to an OEM account on the handset. All of the OEMs collect the list of apps installed in a handset. This is a privacy concern since the list of installed apps can be used to profile user traits and preferences. All of the OEMs collect analytics/telemetry data, raising obvious privacy concerns.

## 1 Introduction

The privacy of mobile apps has been extensively studied, but much less attention has been paid to the privacy practices of the mobile OS itself. While the Android OS (which we take to mean the distribution as installed on a handset, not just the kernel) is partly open source it is usually customised by so-called Original Equipment Manufacturers (OEMs) before being deployed on their handset hardware. Most of these OS customisations are proprietary and closed, with little public documentation. In this paper we use measurements of the data transmitted by handsets manufactured by Samsung, Xiaomi, Huawei and Realme to analyse the data privacy practices of these OEMs. Together, these OEMs represent the great majority of the Android handset market in Europe (Samsung currently has by far the largest share of the Android handset market, followed by Xiaomi, Huawei and Oppo the parent company of Realme [1]).

We seek to address two questions. Firstly, how are identifiers used in the network connections made by the Android OEM software. Best practice, e.g. see [2], is to minimise the persistence of the identifiers used so as to enhance user privacy. For example, long-lived hardware identifiers such as the hardware serial number, handset IMEI, sim IMSI should only be used in a strictly limited way. It is also good practice to isolate identifiers used for different purposes e.g. advertising identifiers should be kept separate from identifiers used for other purposes and in particular kept separate from long-lived hardware identifiers.

Secondly, we ask what are the types of data that the Android OEM software transmits. A mobile OS is, of course, expected to communicate with servers to check for software updates, but we find that all of the OEMs studied also transmit advertising and analytics data as well as data for OEM services such as antivirus checkers, caller ID/blocking and remote device configuration. The latter occurs in our experiments despite the user opting out of OEM services when asked, and making no explicit use of these services.

We focus here on defining simple scenarios that can be applied uniformly to the handsets studied and that generate reproducible behaviour, so allowing direct comparisons. We assume a privacy-conscious but busy/non-technical user, who when asked does not select options that share data but otherwise leaves handset settings at their default value.

To the best of our knowledge this work is the first in-depth measurement study of the data privacy practices of popular proprietary variants of the Android OS. The analysis of whether mobile apps disclose sensitive information to their associated back-end servers has been the focus of much research [3–8], especially with a view to risks such user de-anonymization, location tracking, behaviour profiling, and cross-linking of data by different stakeholders in the device/software supply chain. Probably closest to the present work are recent analyses of the data that web browsers share with their back-end servers [9] and of the data shared by Google Play Services [10–12]. The latter is motivated in part by the emergence of Covid contact tracing apps based on the Google-Apple Exposure Notification (GAEN) system, which on Android requires that Google Play Services to be enabled. The present study is significantly broader in scope.

## 2 Threat model: What is meant by privacy?

The transmission of user data from mobile handsets to back-end servers is not intrinsically a breach of privacy. For instance, it can be useful to share details of the device model/version and the locale/country of the device when checking for software updates. This poses few privacy risks if the data is common to many handsets and therefore cannot be easily linked back to a specific handset/person [13, 14].

Two major issues in handset privacy are (i) release of sensitive data, and (ii) handset deanonymisation i.e. linking of the handset to a person’s real world identity.

*Release of sensitive data*. What counts as sensitive data is a moving target, but it is becoming increasingly clear that data can be used in surprising ways and that so-called metadata can be sensitive data. One example of potentially sensitive metadata is the list of apps installed on a handset. This can reveal user interests and traits [15, 16]. The list of apps can also acts as a handset fingerprint, unique to only a small number of handsets, and so be used for tracking. Another example is the name, timing and duration of the app windows viewed by a user. This can be used to discover the time and duration of phone calls, when texts/messages are sent and received, when a muslim prayer or gay dating app is used, and so on. More generally, such data reveals what apps a user spends most time viewing and which windows within the app they look at most.

Data which is not sensitive in isolation can become sensitive when combined with other data, see for example [17–19]. This is not a hypothetical concern since large vendors including Google, Samsung, Huawei, and Xiaomi operate mobile payment services and supply custom web browsers with the handsets they commercialize.

*Deanonymisation*. Android handsets can be directly tied to a person’s real identity in at least three ways, even when a user takes active steps to try to preserve their privacy. Firstly, via the SIM and phone number. When a person has a contract with a mobile operator then the SIM is tied to that contract and so to the person. In addition, several countries require presentation of photo ID to buy a SIM. Secondly, via the IMEI number which globally uniquely identifies each SIM slot on a handset. The IMEI is used by cellular operators to block network access for a stolen handset [20] and so is commonly linked to the users SIM, phone number and cellular contract. Thirdly, via vendor accounts. For example, Samsung, Xiaomi and Huawei all enccourage people to create user accounts and sign in to them while using one of their handsets. Also, on Android handsets the Google Play store is the main way that people install apps and use of the Google Play store requires login using a Google account. Creation of user accounts typically requires disclosure of personal information that is then linked to device identifiers such as the IMEI, and if used for mobile payments becomes linked to a persons financial details (e.g. credirt card).

## 3 Methodology

### 3.1 Hardware and software used

#### Mobile handsets

(i) Samsung Galaxy S9 (model SM-G960F)/Android 10 (build QP1A.190711.020, One UI v2.0), (ii) Xiaomi Redmi Note 9 (model M2003J15SG)/Android 10 (build QP1A.190711.020, MIUI Global 12.0.7 QJOMIXM), (iii) Realme 6 Pro (model RMX2063)/Android 10 (build RMX2063_11_A.38, realme UI v1.0), (iv) Huawei P10 Lite (model MAR-LX1B)/Android 9 (following US trade sanctions against Huawei, Android 9 is the latest version of Android available on a Huawei handset that we could root) (build 9.1.0.372, EMUI 9.1.0). Rooted using Magisk v20.4 and Magisk Manager v7.5.1.

#### WiFi access point

Raspberry Pi 4 Model B Rev 1.2/Raspbian GNU Linux 11/Mitmproxy 6.0.2 with iptables firewall configured to redirect HTTP/S traffic to port 8080 (on which mitmproxy listens) and also to block UDP traffic on HTTPS port 443 (so as to force any Google QUIC traffic to fall back to using TCP since we have no tools for decrypting QUIC).

### 3.2 Device settings

At the start of each test we removed any SIM card and carried out a hard factory reset of the handset, i.e. we used TWRP to manually wipe the data partition, thereby forcibly removing all user data and settings, all user installed apps and resetting any disk encryption. Note that we observed that simply clicking on the “factory reset” option in the UI sometimes did not fully remove user data and settings.

Following this factory reset, the handset reboots to a welcome screen and the user is then typically asked to agree to terms and conditions, and presented with a number of option screens. To mimic a privacy conscious user, we unchecked any of the options that asked to share data and only agreed to mandatory terms and conditions. *Samsung*: we unchecked the *Sending of Diagnostic Data*, *Information Linking*, *Receipt of Marketing Information* components of the terms and conditions, skipped the *Protect Your Phone screen*, did not sign into a Samsung account (which raises a warning that it disables Samsung Cloud, Bixby, Galaxy Themes, Find My Mobile, Samsung Pass, Galaxy Store, Secure Folder). *Xiaomi*: we unchecked the *Location*, *Send Diagnostic Data Automatically*, *Automatic System Updates*, *Personalised Ads*, *User Experience Programme* options. *Realme*: we unchecked the *User Experience Programme* and *Uploading Device Error Log Data* components of the terms of service, unchecked the *WiFi Assistant* and *Auto-update Overnight* options. *Huawei*: we selected *No Thanks* on the *Enhanced Services* screen, *Later* on the *User Experience Improvement Programme* screen, *Update Manually* on the *Keep Your Software Up To Date* screen. All of the mobile OSe also displayed a Google services screen on first startup. On this we unchecked the *Use Location*, *Allow Scanning*, *Send Usage and Diagnostic Data* options, and we did not log in to a Google user account.

### 3.3 Test design

We focus here on defining simple scenarios that can be applied uniformly to the handsets studied and that generate reproducible behaviour, so allowing direct comparisons. For each handset we carry out the following experiments:

1. Start the handset following a factory reset (mimicking a user receiving a new phone), recording the network activity.
2. Insert a SIM, recording the network activity.
3. Following startup, leave the handset untouched for several days (with power cable connected) and record the network activity. This allows us to measure the connections made when the handset is sitting idle. This test is repeated with the user being logged in and logged out, and with location enabled/disabled.
4. Open the settings app and view every option but leave the settings unchanged, recording the network activity. Then close the app.
5. Open the settings app and enable location, then disable. Record the network activity.
6. Make and receive a phone call, send and receive an SMS message. Record the network activity.
7. Create an OEM user account, if possible. Record the network activity.

### 3.4 Decrypting HTTPS connections

**Fig 1:**
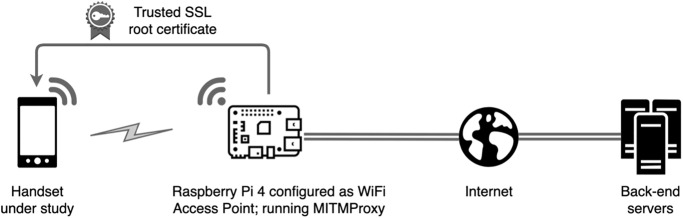
Measurement setup. Mobile handset configured to access the Internet using a WiFi access point hosted on a Raspberry Pi. A system certificate is installed on the phone to be able to decrypt outgoing traffic. The laptop pretends to any process running on the handset to be the destination server, creates a connection to the actual target, and relays requests and their replies between handset and server while logging the traffic.

All of the data we observe is sent over HTTPS connections and so encrypted using TLS/SSL. To decrypt these connections we route handset traffic via a WiFi access point (AP) that we control, configure this AP to use mitmdump as a proxy [21] and adjust the firewall settings to redirect all WiFi HTTP/HTTPS traffic to mitmdump so that the proxying is transparent to the handset. When a process running on the handset starts a new network connection, the mitmdump proxy pretends to be the destination server and presents a fake certificate for the target server. This allows mitmdump to decrypt the traffic. It then creates an onward connection to the actual target server and acts as an intermediary, relaying requests and their replies between the app and the target server while logging the traffic. The setup is illustrated schematically in Fig 1.

The mobile handsets typically carry out checks on the authenticity of server certificates received when starting a new connection and abort the connection when these checks fail. Installing the mitmproxy CA cert as a trusted certificate causes these checks to pass, except on the Huawei handset. On the Huawei handset each system app contains embedded server certificate SHA256 hashed and when starting an HTTPS connection checks that the certificate offered by the server matches one of these hashes. It is thus necessary to bypass these checks on each app individually and we used Riru/edXposed [22, 23] for this.

In addition to the use of HTTPS encryption, the data sometimes has one or more additional inner layers of encryption. In more detail:

*i) Xiaomi*. A number of system apps encrypt data using the Advanced Encryption Standard (AES) with an Electronic Codebook (ECB) [24]. To decrypt this data we used Frida to intercept the entry points to the various functions used to carry out the AES encryption and record the key as it is passed in, allowing the data to then be decrypted.

*ii) Realme*. The app com.heytap.mcs encrypts data with AES and Cipher Block Chaining (CBC). The 128-bit AES key and IV are hard-coded in the app and so can be extracted and used to decrypt the data sent. Messages sent by app com.nearme.romupdate are AES/CTR encrypted base-64 encoded JSON. A token that helps reconstruct the AES key using a custom encoding scheme is appended to the end of the base-64 message. Using this, the message can be decrypted.

*iii) Huawei*. We used Riru/edXposed to extract plaintext and/or encryption keys from the memory, allowing the data to be decrypted.

### 3.5 Categorising identifiers

Identifiers in network connections were extracted by manual inspection. Known values such as the handset IMEI, hardware serial number, Google Advertising Id can often be readily identified in the transmitted data. Otherwise, we manually examined the decompiled app to find the code that writes each value and so establish how the value is generated. This is necessary, for example, to identify values that are hashes of device identifiers.

Following Android’s guide on best practices for unique identifiers [2], we assign identifiers to one of the following persistence categories:

1. *Session-only*. The ID value changes every time the user restarts the app.
2. *Install-reset*. The ID value changes every time user uninstalls and reinstalls the app.
3. *FDR-reset*. The ID value changes every time the user factory-resets the device.
4. *FDR-persistent*. The ID value survives factory reset.

and also to one of the following scope categories:

1. *Single-app*. The ID is used by only one app.
2. *Group of apps*. The ID is used by a group of apps.
3. *Device*. The ID is accessible to all apps installed on the device.

In our measurements the persistence of each identifier is determined by observing whether its value changes after (i) a factory-reset of the handset (which will cause FDR-reset and Session-only identifiers to change value) and (ii) restart of the handset (which stops and restarts any running apps, causing Session-only identifiers to change value.). The *Install-reset* category is not relevant to Android system apps since they cannot be uninstalled. In our measurements the scope of each identifier is determined by (i) observing the number of apps using the identifier, (ii) inspecting where the identifier is stored so as to evaluate its device-wide accessibility.

Best privacy practice is to use identifiers with the smallest scope and shortest persistence possible, which is in line with more general privacy ideas of necessity, minimality and proportionality in the use of potentially sensitive data. For most purposes that means the use of identifiers with persistence no more than FDR-reset, the exception noted by [2] being the use of the handset IMEI and IMSI for use of carrier-related services (e.g. delivery of SMS messages over IP). In particular, common system tasks such as checking for software updates certainly do not require use of persistent handset/user identifiers, instead it is enough to send the device details (installed software version and perhaps hardware model). Similarly, system health monitoring (e.g. software crash reporting) has no need for persistent handset/user identifiers. Specific guidelines in [2] include: (i) avoid using hardware identifiers, (ii) use an advertising ID for advert targeting, measurement, remarketing, ad fraud detection, (iii) do not link the advertising ID to personally identifiable information, (iv) use a resettable ID for app analytics.

Note that these guidelines may change in future, especially those related to advertising and analytics. For example, in Android 12 there is now the option for users to disable use of the Google advertising identifier in apps (an all-zeros value is used instead).

We also note that this Google document is aimed at Android app developers. While it can be claimed that privacy best practices differ for vendors and developers, we argue that such a claim has little merit. Firstly, the distinction between developers and vendors is not a clear one since all of the vendors studied here also develop apps, and developers such as Google develop core parts of the Android OS and major system components such as Google Play Services. Secondly, it is hard to see why vendors and developers should be held to different privacy standards. To the best of our knowledge, no such distinction is made in Europe’s data privacy regulations.

### 3.6 Categorizing network connections

We assign network connections to one, or more, of the following categories:

1. *Firmware Updates*. Used to query the latest OS over-ther-air (OTA) update version.
2. *App Updates*. Used to query the latest version of individual apps or groups of apps (including system apps).
3. *Analytics*. Logging of device activity, including user interactions with apps (screens viewed etc).
4. *Services*. Provision of OEM system services. Examples: message push services (Xiaomi, Realme), configuration of device features/settings (Samsung, Realme), Caller ID and blocking (Samsung), Anti-virus checking (Huawei, Xiaomi), Swiftkey keyboard (Huawei).
5. *Advertising*. Download of adverts and tracking of impressions and user interactions with ads.

These categories were derived by analysis of the network connections that we measured. We note, however, that there is no widely agreed approach to categorising Android system network connections.

We observed that each URL is used for a single purpose, e.g. on the Realme handset all connections to adx-f.ads.heytapmobile.com relate to advertising, and so there is no need to distinguish between individual endpoints sharing the same URL.

## 4 Results

### 4.1 Scope/persistence of identifiers used by Android OEMs

**Fig 2:**
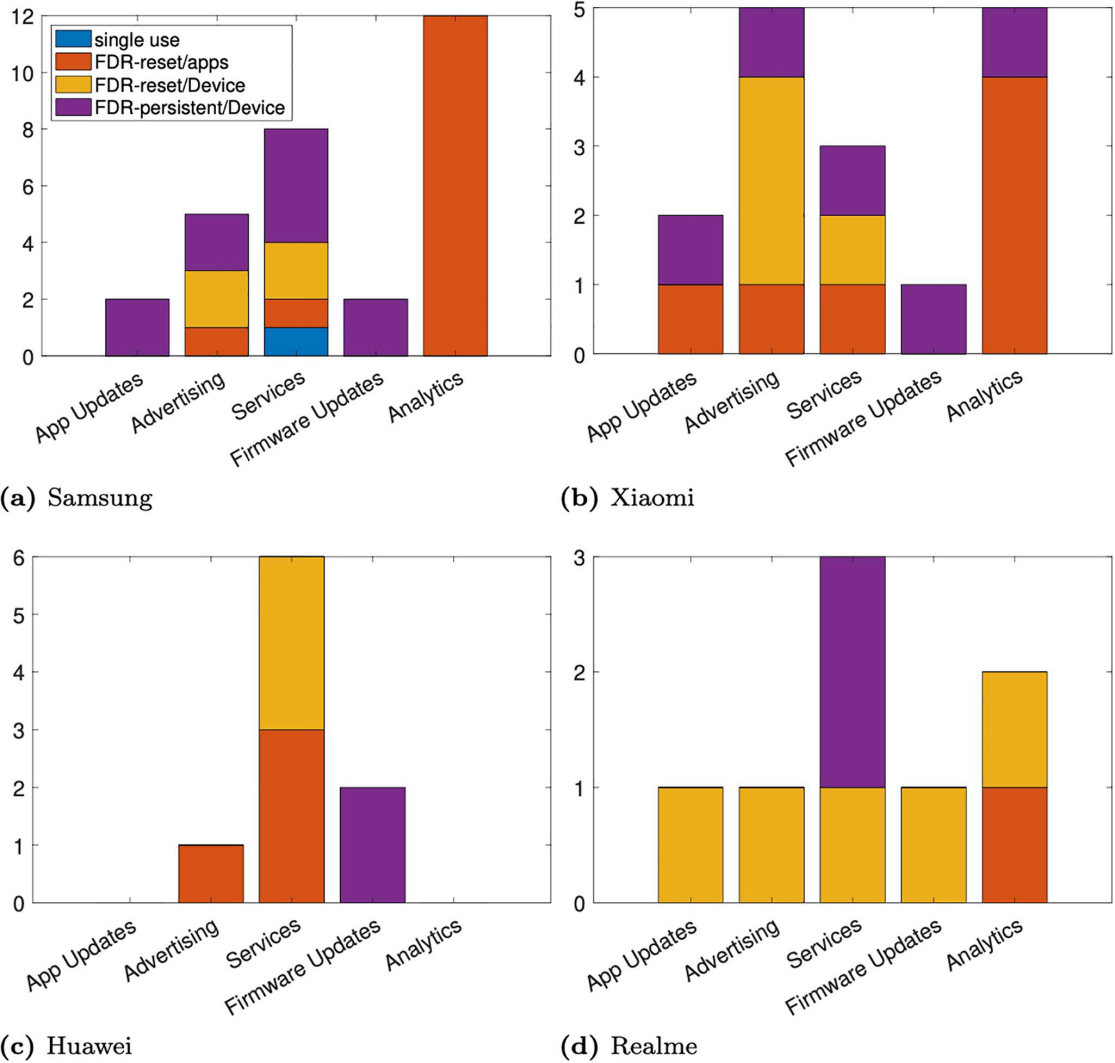
Summary of measured identifier scope/persistence vs category of network connection and OEM. Network connections without identifiers are not shown. (a) Samsung, (b) Xiaomi, (c) Huawei, and (d) Realme.

Fig 2 summarises the identifier scope and persistence for each category of network connection. It can be seen that Huawei and Realme make less use of FDR-persistent identifiers than do Samsung and Xiaomi, as well as tending to have fewer network connections contained identifiers. In more detail, we make a number of observations:

1. With the exception of Xiaomi, checking for firmware updates make use of FDR-persistent identifiers with the greatest persistence and scope e.g. the hardware serial number, handset IMEI. We found this surprising since firmware updates are shared by all devices of the same model. Checking for updates therefore does not require any individual device identifiers, only device model and software version. Similarly, FDR-persistent identifiers can also transmitted by Samsung and Xiaomi when checking for app updates.
2. With the notable exception of Xiaomi (discussed further below), resettable identifiers are used in analytics connections in accordance with good practice. Xiaomi sends the handset IMEI (which uniquely identifies the handset), sim IMSI (which uniquely identifies the SIM on the mobile network), a hash of the handset Wifi MAC address, and the security device ID to tracking.intl.miui.com. These identifiers are all FDR-persistent. This connection is used to record user interactions with the apps installed on the device (app screens viewed etc).
3. Huawei and Realme use resettable identifiers in advertising connections, in accordance with good practice. Samsung and Xiaomi, however, use a mix of resettable and FDR-persistent identifiers. Samsung sends the handset IMEI (an FDR-persistent identifier) in network connections to sdk.pushmessage.samsung.com that are associated with the Samsung Push Service which is used for marketing messages. Xiaomi sends a hash of the handset IMEI to sdkconfig.ad.intl.xiaomi.com used for advert configuration. Both seem avoidable.
4. All of the handsets use the Google Advertising ID in at least one of their network connections associated with advertising. This is an FDR-resettable identifier, in accordance with good practice, but serves to highlight the central role played by Google in Android advertising.
5. Various mixes of identifier types are used in network connections for OEM system services. With the exception of Huawei, all of the OEMs use FDR-persistent identifiers for at least one service, but resettable identifiers for other services, see Table 1. Recall that in our experiment setup the user opts out of OEM services when asked, and in any case makes no use of OEM services. All of these connections therefore ought to be avoidable.

**Table 1:** FDR-persistent identifiers used in network connections associated with OEM system services.

OEM	Service	FDR-persistent identifiers
Samsung	Knoxguard	Hashes of IMEI, hardware serial number
Samsung	Device config	Hardware serial number, Samsung Consumer ID
Xiaomi	Xiaomi Cloud	Xiaomi cloudsp_fid, security DeviceID
Realme	Warranty	IMEI
Realme	Device config	Heytap DeviceId

### 4.2 Linking identifiers to user’s real identity

Our experimental protocol involves the user creating/logging in to an OEM user account on each handset. This is to allow observation of which device identifiers are linked to user personal information during the sign-in process. Note that there was no OEM account login option on the Realme handset studied.

Table 2 reports the FDR-persistent identifiers sent alongside user personal identifiers (email etc) during sign-in. It can be seen that for all three OEMs which have user sign-in personal identifiers are sent alongside one or more FDR-persistent identifiers, thereby trivially linking these together.

**Table 2:** FDR-persistent identifiers linked to personal identifiers during sign-in to OEM account on handset. There was no OEM account login option on the Realme handset studied.

OEM	Personal identifiers	FDR-persistent identifiers
Samsung	Email, phone number	IMEI
Xiaomi	Email	Xiaomi cloudsp_fid, security DeviceID
Huawei	Email, phone number	Hardware serial number

Table 3 lists the FDR-persistent identifiers which are sent together in other connections (i.e. not during sign-in) and so trivially linkable. It can be seen from Table 3 that the FDR-persistent identifiers linked to user personal identifiers in Table 2 are also sent alongside most of the other FDR-persistent identifiers on each handset, thereby potentially linking user personal identifiers to all of these handset FDR-persistent identifiers.

**Table 3:** FDR-persistent identifiers sent together in same network connection (and so trivially linkable), plus whether Google Ad ID is sent in the same connection (so making it trivially linkable to the FDR-persistent identifiers).

OEM	FDR-persistent identifiers sent together	Google Ad ID sent
Samsung	IMEI, Hardware serial number, Samsung Consumer ID	no
Xiaomi	IMEI, IMSI, hash of WiFi MAC address, Xiaomi cloudsp_fid, security DeviceID	yes
Huawei	Hardware serial number, device cert	no
Realme	IMEI	no

In addition, it can be seen from Table 3 that on the Xiaomi handset the Google Ad ID is sent alongside the FDR-persistent identifiers, and so can be trivially linked to these and therefore also to user personal identifiers.

### 4.3 What sort of data is being collected?

The data that we observe being sent from handsets can be categorized as: (i) device configuration data, (ii) details of apps installed and (iii) event logging data/telemetry.

#### 4.3.1 Device configuration data

As already noted, sharing device hardware/system configuration data such as the device model, screen size, operating system version, radio version generally carries little privacy risk if the combination of device data values is common to many handsets (the situation is, of course, very different when the combination of device data is specific to an individual handset or a small number of handsets since it can then potentially be used for device fingerprinting). Such data is needed when checking for software updates and selecting the right version of an app to install.

Table 4 summarises the collection of device configuration data observed in our measurements. It can be seen that, as expected, all of the OEMs transmit this information when checking for firmware and app updates. This data is also transmitted by one or more services of every OEM (although as noted above in our experiment setup the user opts out of OEM services when asked and makes no use of OEM services). All of the OEMs transmit device configuration data to their advertising endpoints, while Xiaomi and Realme also transmit this data in their analytics connections.

**Table 4:** Connection categories sending device hardware/system configuration data.

	Connection Category				
	Firmware Updates	App Updates	Services	Advertising	Analytics
Samsung	x	x	x	x	-
Xiaomi	x	x	x	x	x
Huawei	x	-	x	x	-
Realme	x	x	x	x	x

#### 4.3.2 List of installed apps

This list of apps installed on a handset is potentially sensitive information since it can reveal traits of the handset user. For example a muslim prayer app, a period tracking app, a gay dating app, a mental health app can, respectively, reveal the user’s religion, gender, sexual preference and mental health status. The newspaper apps installed can reveal pollitical preferences. The set of apps installed is also more likely to be unique to one handset, or a small number of handsets, and so can act as a device fingerprint (especially when combined with device hardware/system configuration data). See, for example, [15, 16, 25–27] for recent analyses of such privacy risks. We note that in light of such concerns, Google recently categorised the list of apps installed as “personal and sensitive user data” and introduced restrictions on Play Store apps collection of this data [28], but such restrictions do not apply to system apps since these are not installed via the Google Play store.

Table 5 summarises the collection of the list of installed apps observed in our measurements. It can be seen that, with the exception of Huawei, all of the OEMs transmit this data. Xiaomi and Realme transmit this data to app update endpoints and in both cases only FDR-reset identifiers are sent in these connections, which is reasonable practice although it would enhance privacy to use less persistent identifiers e.g. session-based identifiers. Samsung and Realme transmit the list of installed apps to endpoints associated with OEM services. Xiaomi transmit the list of installed apps to an analytics endpoint, along with FDR-persistent identifiers, a poor privacy practice.

**Table 5:** Connection categories sending list of installed apps.

	Connection Category		
	App Updates	Services	Analytics
Samsung	-	Device Settings	-
Xiaomi	x	-	x
Huawei	-	-	-
Realme	x	Oppo Push	-

#### 4.3.3 Event logging data/teleemtry

All of the OEMs studied log user interactions with system apps, see Table 6. Note that this occurs despite the user opting out of diagnostics/analytics collection on the handsets during onboarding following factory reset. While some logging of app usage is reasonable, e.g. to allow early detection of app performance issues, ongoing logging of user activity can quickly become intrusive and a serious privacy concern.

**Table 6:** Summary of telemetry data sent in network connections.

	Telemetry Data Collected
Samsung	SIM insertion, making/receiving phone call, details of user interactions with selected system apps
Xiaomi	Details of user interactions with all apps (not just selected system apps)
Huawei	Events and settings associated with com.huawei.himovie.overseas system app, logs app details when a new app is installed. When the keyboard is used within an app, the app name and details of user interactions are logged.
Realme	Events associated with com.heytap.mcs system app.

Perhaps the most striking logging of user interactions is by Xiaomi, which logs user interactions with every app. Interactions logged include every opening and closing of app windows plus the duration a window is open. FDR-persistent identifiers and the Google Ad ID are sent alongside this data. Xiaomi system apps com.miui.msa.global, com.xiaomi.discover, com.android.thememanager also log user interactions using Google Analytics.

It can be seen from Table 6 that several Samsung system apps use Google Analytics to log user interaction events, including windows/activities viewed plus duration and timestamp (namely the apps com.wssyncmldm, com.samsung.android.samsungpass, com.samsung.android.authfw, com.samsung.android.bixby.agent, com.samsung.android.game.gamehome, com.sec.android.app.samsungapps). The caller ID/call blocking service logs the making/receiving of phone calls and the Google Ad ID is sent alongside this data.

Huwaei log events (e.g. app start up) associated with system app com.huawei.himovie.overseas and when a new app is installed the app details are sent to a scanning service. When the keyboard is used within an app, the app name, number of characters entered and an event timestamp are sent to Microsoft e.g. use of searchbar, contacts and messaging apps is logged. The Google Ad ID is sent alongside this data.

The Realme handset logs events (e.g. app start up) related to the system app com.heytap.mcs.

## 5 Discussion

### 5.1 Limitations

The analysis here is based on measurement data. The data itself is not subject to noise or measurement error since it is digital in nature. However, there are two main potential sources of error in the analysis.

The first is that the categorization of network connections may be inaccurate. OEMs provide no public documentation on purpose of the connections made by their handsets and so the categorization is based on our analysis of the content of network connections and of the apps that generated them. As partial validation, we have contacted all of the OEMs studied and presented our measurement data to them so we believe there are no significant errors in the categorization used.

A second potential source of error is that the behaviour of our rooted handsets may differ from that of unrooted handsets. As partial validation we also collected traffic measurements from unrooted handsets and compared these with the measurements on our rooted handsets. On unrooted phones the connections remain encrypted but the SNI can be extracted from the TLS handshake and used to discover the server name associated with each connection. This highlighted that on the Samsung handset connections to diagmon-serviceapi.samsungdm.com are present on unrooted phones but not on rooted phone—further investigation established that the DiagmonAgent app relies on trusted storage, which is disabled by Samsung on rooted handsets. However, we did not see connections on the rooted handsets which were not also present on the unrooted handsets, and so the analysis here is likely conservative in the sense that it applies to the subset of connections present on both rooted and unrooted handsets.

We note also that several of the OEMs use third-party apps to provide system services. Namely, Huawei use the Microsoft Swiftkey keyboard and the Avast and 360 Safe antivirus checkers, Samsung uses the Hiya caller ID/call blocking service, Realme partners with Heytapi. Since these services are installed by the OEM we include the associated network connections in our analysis. Excluding these network connections results in only small changes in the analysis.

All of the handsets studied have Google Play Services pre-installed and transmit data to Google servers. Since our focus here is on OEM practices we do not include these Google connections in the present analysis. For Google-specific measurement studies see e.g. [10, 11].

We study the European models of handsets from Samsung, Xiaomi, Huawei and Realme and use the handsets within Europe. The data collection behaviour on models targeted at other regions may differ.

### 5.2 GDPR

The data collection that we observe raises questions regarding data protection regulations in Europe (as already noted, the measurements were all carried out within Europe using handsets purchased in Europe). Roughly speaking, there are three main basis under GDPR for data collection, e.g. see [29]: (i) the data is anonymised, i.e. cannot reasonably be linked to an individual person, and so is not personal data, (ii) with informed consent for a defined purpose and (iii) for the legitimate interests of the vendor.

#### 5.2.1 Lack of anonymity

Samsung, Xiaomi and Huawei all encourage people to create user accounts and sign in to them while using one of their handsets, see Table 2 for a summary of the personal identifiers sent during account login. Also, on Android handsets the Google Play store is the main way that people install apps and use of the Google Play store requires login using a Google account.

*Samsung*. To create a Samsung account it is necessary to provide a working phone number (to receive a verification code). For many people this will be their own phone number. If in addition the user ever pays for a Samsung service, e.g. Samsung Cloud for sync, backup and photos, then their account becomes linked to their credit card details etc. A user’s Samsung account can therefore commonly be expected to be linked to the person’s real identity. During account login the handset IMEI is sent to Samsung, linking this to these user personal details. In other connections the IMEI sent alongside the Samsung consumer ID, handset serial number and Google Firebase authentication tokens thereby linking all of these to the user account details. Hence, if a user logs in to their Samsung account at least once on their handset then data recording user interactions with apps, details of installed apps etc become linked to the user account details.

*Xiaomi*. The situation with Xiaomi is similar. To create a Xiaomi/Mi account it is necessary to provide either a working email address or phone number (to receive a verification message) and if the user ever pays for a Xiaomi service then their account becomes linked to their credit card details etc. During account login the Xiaomi Security Device ID is sent to Xiaomi (which in turn is linked to the handset IMEIs, Wifi MAC address, the Google Ad ID, the handset CPUID and VAID), thereby linking the device to the user personal details and so also data recording user interactions with apps, details of installed apps etc.

*Huawei*. To create a Huawei account requires both a working phone number and a working email address (both are separately verified by sending a text/email with a verification code). If the user ever pays for a Huawei service then their account becomes linked to their credit card details etc. During account login the handset hardware serial number is sent to Huawei, linking the device to the user personal details and so also details of installed apps etc.

*Realme*. The Realme handset sends data to Realme and Heytap (a partner of Realme). However, on the EU handset we studied there is no provision for logging in to a Heytap account (in other regions, e.g. India, Realme handsets come pre-installed with apps such as Heytap Cloud) and so the data collected is presumably not linked to user personal details.

*Google*. When creating a Google account it is necessary to personal details (name, email) and users are also encouraged to supply a working phone number. Use of Google services such as buying a paid app on the Google Play store further links a person’s Google account to their credit card/bank details. During account login the Google Android ID is linked to the user personal details and via other data collected by Google Play Services the Google Android ID is linked to the handset hardware serial number, IMEI and Wifi MAC address. All of the events recorded via Google Analytics (e.g. by Samsung) are tagged with the user’s Google Advertising ID and the sender app’s Firebase ID. The app Firebase ID is directly linked to the handset Android ID when the app registers to use the Google Analytics service, and so to the device and Google user account.

In addition to data becoming linked to a person’s real identity when they login to a vendor user account on a device, much of the data that we observe to be collected is tagged with the Google Ad ID. There already exist commercial services that given a Google Ad ID offer to supply the name, address, email etc of the person [30].

#### 5.2.2 No informed consent

All of the handsets studied require agreement to terms and conditions on first startup following a factory reset, and these typically accessible from the Settings-About Phone screen. These are fairly long documents that are easy to click past (a separate link has to be clicked to view them) written in legalistic language and so it is unlikely that many people read them. Such an approach seems out of line with the requirement in GDPR for separate informed and active consent for each specific purpose. Specific consent for data collection for each separate and clearly specified purpose is generally not sought, and there is no opt out from the data collection we observe in this study. Note that when logging into a Huawei account on the Huawei handset then popup screens are displayed with details on information collected and the option given to exit from the login process, but this is a notable exception.

#### 5.2.3 Legitimate interest

Invoking legitimate interest requires the data to be collected for a specific purpose, that the data is necessary for the purpose, that the data collection is balanced against the interests and freedoms of the individual, and so on e.g. see [31]. The legitimate interest basis for data collection is the least clear, and probably best left to the lawyers. However, we note the specific purposes of the data collection that we observe here is generally not easy for an ordinary person to determine from the phone documentation.

### 5.3 Privacy scoring

Our analysis can be used as the basis for ranking the privacy of the handsets studied. Various metrics might be used for generating a score to use for ranking, but one approach is to rank handsets based on how many changes are needed to bring the use of identifiers into line with current best practice. Use of long-lived hardware identifiers is not warranted for the data transmitted in our experiments and so we score 1 for each connection using such identifiers. Device-wide identifiers are also not warranted, and should be replaced by single-app or group-of-apps identifiers. We score 0.5 for each connection using device-wide FDR-reset identifiers. Using this approach we arrive at the following scores: Samsung 8 + 4 × 0.5 = 10, Xiaomi 5 + 4 × 0.5 = 7, Huawei 2 + 3 × 0.5 = 3.5, Realme 2 + 5 × 0.5 = 4.5 and a ranking with Realme and Huawei most private, followed by Xiaomi and then Samsung.

This scoring system is certainly imperfect, e.g. it ignores the intrusive telemetry collection by Xiaomi (logging user interactions with all apps) and the failure by Xiaomi to keep the Google Ad Id separate from long-lived hardware identifiers, but it does highlight the clear difference between the use of identifiers by Huawei/Realme and Samsung/Xiaomi. It also serves to make the point that the data generated by the analysis here has the potential for use in privacy scoring, although we leave the development of more refined scoring approaches to future work.

## 6 Conclusions

In this paper we present the results of a measurement study looking at the data transmitted by several proprietary variants of the Android OS. Our measurements show thatt all of the OEMs use olong-lived hardware identifiers such as the hardware serial number, handset IMEI and these hardware identifiers are linked to the handset user’s real identity when they sign in to an OEM account on the handset. The list of apps installed on a handset is also collected by of the OEMs, which raises privacy concerns since this may be used to profile user traits and preferences. We observe that all of the OEMs collect analytics/telemetry data.

## Supporting information

S1 File Summary of measurement data. (PDF)

S2 File Content of network connections. (PDF)
