# Supplementary material for: On the data privacy practices of Android OEMs
Source: PLoS One. 2023 Jan 18;18(1):e0279942. doi: 10.1371/journal.pone.0279942 (PMC9847909; doi:10.1371/journal.pone.0279942)
Supplement: S1 File — (PDF) [file pone.0279942.s001.pdf]

S1 Summary Of Measurement Data

Identifier	Persistence	Scope
hardware serial number	FDR-persistent	device
IMEI	FDR-persistent	device
Samsung Consumer ID	FDR-persistent	device
secure settings android_id	FDR-reset	device
Firebase ID	FDR-reset	group of apps
Firebase tokens	FDR-reset	group of apps
Google Ad ID	FDR-reset	device
-odc.samsungapps.com transactionId	single use	single app
-odc.samsungapps.com sessionId	session only	single app
-odc.samsungapps.com logId (or stduk)	FDR-persistent	group of apps
-odc.samsungapps.com cookie JSESSIONID	session only	single app
gos-api.gos-gsp.io uuid	FDR-reset	single app
capi.samsungcloud.com device_id	FDR-reset	device
capi.samsungcloud.com access_token	FDR-reset	single app
us-api.mcsvc.samsung.com x-smcs-did	FDR-reset	device
us-rd.mcsvc.samsung.com sid	FDR-reset	single app
us-rd.mcsvc.samsung.com ri	FDR-reset	single app
samsung-directory.edge.hiyaapi.com X-Hiya-Request-Id	single use	single app
samsung-directory.edge.hiyaapi.com x-seq_id	single use	single app
samsung-directory.edge.hiyaapi.com X-Hiya-Installation-User-ID (also called hin)	FDR-reset	single app
samsung-directory.edge.hiyaapi.com X-Hiya-Device-User-ID	FDR-reset	single app
samsung-directory.edge.hiyaapi.com Authorization bearer	FDR-reset	single app
oath nonce/token	session only	single app
x-samsung-trace-id	single use	single app

Endpoint	Category	Identifiers
hub-odc.samsungapps.com	App Updates	transactionId, sessionId, logId
ie-odc.samsungapps.com	App Updates	IMEI, transactionId, sessionId, logId, cookie JSESSIONID
vas.samsungapps.com	App Updates	
gos-api.gos-gsp.io	Services/App Config	uuid, x-samsung-trace-id
api.omc.samsungdm.com	Services/Device Settings	hardware serial number, Samsung Consumer ID, Firebase ID/token (for com.samsung.android.app.omcagent)
dir-apis.samsungdm.com	Advertising	IMEI, hardware serial number, Samsung Consumer ID
api.gras.samsungdm.com	Services/Device Settings	hardware serial number, Firebase ID/token (for com.samsung.android.sdm.config)
fota-apis.samsungdm.com	Services/Device Settings	oath nonce
capi.samsungcloud.com	Services/Device Settings	device_id, access_token
gslb.secb2b.com	Services/Knoxguard	hash of hardware serial number
eu-kaf.samsungknox.com	Services/Knoxguard	hashes of IMEIs, hash of hardware serial number
eu-segd-api.secb2b.com	Services/Knoxguard	hashes of IMEIs, hash of hardware serial number
www.ospserver.net	Firmware Updates	IMEI, Samsung Consumer ID, hardware serial number, Firebase ID/token (for app wssyncmldm - an update monitor app), oath nonce
fota-cloud-dn.ospserver.net	Firmware Updates	-
dms.ospserver.net	Firmware Updates	IMEI, sid
sspapi-prd.samsungrs.com	Advertising	Google AID
sdk.pushmessage.samsung.com	Advertising	IMEI, p_deviceId/smpid value returned by server after request sending IMEI, Firebase ID/token (for app com.sec.spp.push "Samsung Push Service")
us-api.mcsvc.samsung.com	Advertising	x-smcs-did
us-cdn-gpp.mcsvc.samsung.com	Advertising	-
us-rd.mcsvc.samsung.com	Advertising	sid, ri, plus others: ii, pg, pi, li, cp downloaded with ad and all are FDR-persistent, also ri, sid downloaded with ad and these are FDR-reset.
samsung-directory.edge.hiyaapi.com	Services/CallerIdBlocking	Google Ad ID (X-Hiya-Advertising-ID), X-Hiya-Request-Id, x-seq id, X-Hiya-Installation-User-ID, X-Hiya-Device-User-ID, Authorization bearer, eventId
Firebase	Analytics	GAID, `com.sec.android.app.samsungapps`, `com.samsung.android.app.simplesharing`, `com.samsung.android.authfw`, `com.samsung.android.bixby.agent`, `com.samsung.android.kgclient`, `com.samsung.android.mobileservice`, `com.samsung.android.rubin.app`, , `com.samsung.android.themestore`, `com.sec.android.app.billing`, `com.samsung.android.game.gamehome` Firebase ids

Table 1: Samsung

Identifier	Persistence	Scope
IMEI	FDR-persistent	device
IMSI	FDR-persistent	device
Security DeviceID	FDR-persistent	device
Xiaomi VAID	FDR-reset	device
Google Ad Id/GAID	FDR-reset	device
android_id	FDR-reset	device
Xiaomi AAID	FDR-reset	device
Xiaomi devID	FDR-reset	device
cloudsp_fid	FDR-persistent	device
Wifi MAC address	FDR-persistent	device
data.mistat.intl.xiaomi.com sid	session only	single app
data.mistat.intl.xiaomi.com AES key	session only	single app
mcc.intl.inf.miui.com uid	FDR-reset	single app
global.market.xiaomi.com guid	FDR-reset	single app
Firebase ID	FDR-reset	group of apps
Firebase tokens	FDR-reset	single app
au.ff.avast.sec.miui.com uuid	FDR-reset	single app

Endpoint	Category	Identifiers
tracking.intl.miui.com	Analytics	IMEIs, IMSI, VAID, GAID,cloudsp_fid, hash of Wifi MAC address, instance id, CPUID, Security DeviceID
api.sec.intl.miui.com	App Updates	hash of android id in secure settings, hash of Security DeviceID
api.ad.intl.xiaomi.com	Advertising	GAID
sdkconfig.ad.intl.xiaomi.com	Advertising	hash of IMEI
privacy.api.intl.miui.com	Advertising	GAID
update.intl.miui.com	Firmware Updates	hash of IMEI, hash of Security DeviceID
fr.register.xmpush.global.xiaomi.com	Services/Xiaomi Push	GAID, VAID, AAID, devId
find.api.micloud.xiaomi.net	Services/Xiaomi Cloud	cloudsp_fid, Security DeviceID
data.mistat.intl.xiaomi.com	Advertising	sid, AES key, android_id
mcc.intl.inf.miui.com uid	Advertising	uid
global.market.xiaomi.com	App Updates	guid, instance_id (Firebase ID of app com.xiaomi.discover)
au.ff.avast.sec.miui.com	Services/Antivirus	uuid
Firebase	Analytics	GAID, com.google.android.apps.messages, com.miui.msa.global, com.xiaomi.discover, com.mi.android.globalFileexplorer Firebase ids

Table 2: Xiaomi

Identifier	Persistence	Scope
hardware serial number	FDR-persistent	device
device cert	FDR-persistent	device
huawei udid	-	
servicesupport.hicloud.com userId	FDR-reset	single app
360safe.com udid	FDR-reset	single app
android id	FDR-reset	device
avast.com ABUID	FDR-reset	single app
avast.com X-AVAST-KeyId	FDR-reset	single app
Google Ad ID /GAID	FDR-reset	device
pebed.dmevent.net instance_uuid	FDR-reset	single app
pebed.dmevent.net id/cookie	FDR-reset	single app
installid	FDR-reset	single app

Endpoint	Category	Identifiers
query.hicloud.com	Firmware Updates	hardware serial number, device cert, udid
configserver-dre.platform.hicloud.com	Firmware Updates	hardware serial number
servicesupport.hicloud.com	Services/UI Theme	userId
shepherd.sb.avast.com	Services/Antivirus	hash of android id, ABUID
apkrep.ff.avast.com	Services/Antivirus	hash of android id, ABUID, X-AVAST-KeyId
mvconf.cloud.360safe.com	Services/Antivirus	uid
mclean.cloud.360safe.com	Services/Antivirus	uid
pebed.dmevent.net	Advertising	GAID, instance_uuid, id/cookie
telemetry.api.swiftkey.com	Services/Keyboard	installid, GAID
in.appcenter.ms	Services/Keyboard	installid
Firebase	Analytics	GAID, com.google.android.apps.messages Firebase id

Table 3: Huawei

Identifier	Persistence	Scope
IMEI	FDR-persistent	device
Google Ad ID	FDR-reset	device
OUID	FDR-reset	device
DUID/VAID	FDR-reset	device
registrationId	FDR-reset	device
shorteuex.push.heytapmobile.com device_id	FDR-reset	single app
httpdns-euex-push.heytapmobile.com deviceID	FDR-persistent	device
guid	FDR-reset	device

Endpoint	Category	Identifiers
ifrus-eu.coloros.com	Firmware Updates	guid, registrationId
ifota-eu.realmemobile.com	Firmware Updates	guid, registrationId
icosa-eu.coloros.com	AppUpdates	guid
esa-reg-eup.myoppo.com	Services/Warranty	IMEI, guid
httpdns-euex-push.heytapmobile.com	Services/Config	deviceId, DUID/VAID
adx-f.ads.heytapmobile.com	Advertising	GAID, OUID
shorteuex.push.heytapmobile.com	Services/Oppo Push	DUID/VAID, OUID, device_id, Firebase id and token (for app com.heytap.mcs)
dceuex.push.heytapmobile.com	Analytics	DUID/VAID
Firebase	Analytics	GAID, com.heytap.mcs Firebase id

Table 4: Realme

Endpoint	Device Config	List of Apps	Telemetry
Samsung			
`gos-api.gos-gsp.io`		x	
`www.ospserver.net`	x		
`dms.ospserver.net`	x		
`sdk.pushmessage.samsung.com`	x		
`sspapi-prd.samsungrs.com`	x		
`us-api.mcsvc.samsung.com`	x		
`samsung-directory.edge.hiyaapi.com`		x	x
`api.omc.samsungdm.com`		x	
Xiaomi			
`tracking.intl.miui.com`	x	x	x
`global.market.xiaomi.com`	x	x	
`api.sec.intl.miui.com`	x		
`api.ad.intl.xiaomi.com`	x		
`fr.register.xmpush.global.xiaomi.com`	x		
`sdkconfig.ad.intl.xiaomi.com`	x		
`mcc.intl.inf.miui.com`	x		
Huawei			
`query.hicloud.com`	x		
`configserver-dre.platform.hicloud.com`	x		
`servicesupport.hicloud.com`	x		
`shepherd.sb.avast.com`	x		
`apkrep.ff.avast.com`	x		x
`mvconf.cloud.360safe.com/safeupdate`	x		
`telemetry.api.swiftkey.com`	x		x
`in.appcenter.ms`	x		x
`bibo.api.swiftkey.com`	x		
`pebed.dm-event.net`	x		x
Realme			
`ifrus-eu.coloros.com`	x		
`ifota-eu.realmemobile.com`	x		
`icosa-eu.coloros.com`		x	
`adx-f.ads.heytapmobile.com`	x		
`shorteuex.push.heytapmobile.com`	x	x	
`dceuex.push.heytapmobile.com`	x		x

Table 5: Type of data observed transmitted to each endpoint.
