# Supplementary material for: On the data privacy practices of Android OEMs
Source: PLoS One. 2023 Jan 18;18(1):e0279942. doi: 10.1371/journal.pone.0279942 (PMC9847909; doi:10.1371/journal.pone.0279942)
Supplement: S2 File — (PDF) [file pone.0279942.s002.pdf]

S2 CONTENT OF NETWORK CONNECTIONS

Note: To save space and reduce repetition, common Google Play Services and Google Play store connections are not included, e.g.

- 1) <https://play.googleapis.com/vn/log/batch>
- 2) <https://play.googleapis.com/play/log>
- 3) <https://www.googleapis.com/experimentsandconfigs>
- 4) <https://www.googleapis.com/androidantiabuse>

and connections made by Chrome e.g.

- 1) <https://safebrowsing.googleapis.com/v4/threatListUpdates:fetch>

that are already documented elsewhere.

I. SAMSUNG

Summary:

<i>Samsung system app endpoints:</i>	<i>Identifiers Sent:</i>
gos-api.gos-gsp.io	uuid
api.omc.samsungdm.com	hardware serial number, Samsung Consumer ID, Firebase IDs/tokens
dir-apis.samsungdm.com	IMEIs, hardware serial number, Samsung Consumer ID
api.gras.samsungdm.com	hardware serial number, regID
capi.samsungcloud.com	device_id, authorization value
pinning-02.secb2b.com	deviceid
eu-kaf.samsungknox.com	hashes of IMEIs, hash of hardware serial number
www.ospserver.net	IMEIs, Samsung Consumer ID, hardware serial number, mobile network cell ID/location
fota-cloud-dn.ospserver.net	IMEI, Firebase ID
sspapi-prd.samsungs.com	GAID
dms.ospserver.net	IMEIs
sdk.pushmessage.samsung.com	IMEI, Firebase ID
samsung-directory.edge.hiyaapi.com	GAID, other IDs
us-api.mcsvc.samsung.com	x-smcs-did
us-rd.mcsvc.samsung.com	sid
ie-odc.samsungapps.com	IMEI
<i>Third-party (non-Google) system app endpoints:</i>	<i>Identifiers Sent:</i>
sun-apps.sfr.com	Firebase ID
mobile.pipe.aria.microsoft.com	secure settings android_id, DeviceInfo.SDKUid, cai.device.id, TenantId
config.edge.skype.com	secure settings android_id
app.adjust.com	GAID, android_uuid
www.linkedin.com	bcookie, bscookie, lide, trackingId
samsung-directory.edge.hiyaapi.com	GAID X-Hiy-Installation-User-ID, hin, hui
Identifiers observed to persist across factory reset	IMEIs (including hashes of the IMEIs), hardware serial number, Samsung Consumer ID

TABLE I

SUMMARY OF IDENTIFIERS SENT IN SYSTEM APP CONNECTIONS (EXCLUDING GOOGLE SYSTEM APPS).

- 1) The handset sends the following device identifiers to api.omc.samsungdm.com and dir-apis.samsungdm.com in a way that links them all together: hardware serial numbers (both ro.serialno and ril.serialnumber in getProp), IMEI of both SIM slots and Samsung device/consumer id. In addition a Google Firebase authentication token is sent to api.omc.samsungdm.com, allowing linkage of data collection by Samsung and Google Firebase. A connection to dir-apis.samsungdm.com sends cell tower identifiers (LAC and UCID) that reveal the approximate device location.
- 2) Details of installed software are sent to os-api.gos-gsp.io/, which appears to be a Samsung domain associated with a software configuration service (gos may be an acronym for “game optimisation service”). The service returns app settings such as allow_more_heat, boost_launch, enableCpuMinFreqControl).
- 3) Device details, including the hardware serial number, are sent to api.gras.samsungdm.com. Also sent is a Google Firebase authentication token associated with com.samsung.android.sdm.config.

Telemetry	
api.omc.samsungdm.com	logs SIM insertion
samsung-directory.edge.hiyaapi.com	logs making/receiving phone call
sun-apps.sfr.com	time of last reboot, wakeup_success, wakeup_error, error stack traces, whether SIM inserted, duration app com.altice.android.myapps has been active
System apps com.wssyncmldm, com.samsung.android.samsungpass,com.samsung.android.authfw,com.altice.android.myapps,com.samsung.android.bixby.agent,com.samsung.android.game.gamehome,com.sec.android.app.samsungapps,com.sfr.android.sfrjeux	use Google Analytics to log user interaction, including screens/activities viewed plus duration and timestamp.
Device Data	
gos-api.gos-gsp.io	details of installed apps
www.ospserver.net, dms.ospserver.net	device details
api.omc.samsungdm.com, dir-apis.samsungdm.com, api.gras.samsungdm.com, ie-odc.samsungapps.com, sdk.pushmessage.samsung.com	device details
sspapi-prd.samsungs.com, us-api.mcsvc.samsung.com	device model
samsung-directory.edge.hiyaapi.com	installed apps
sun-apps.sfr.com	device details
mobile.pipe.aria.microsoft.com, vortex.data.microsoft.com	device details, details of installed Microsoft apps
app.adjust.com	device details
www.linkedin.com	device details

TABLE II

SUMMARY OF DATA SENT IN SYSTEM APP CONNECTIONS (EXCLUDING GOOGLE SYSTEM APPS).

- 4) Device and app identifiers are sent to capi.samsungcloud.com. This appears to be associated with the Samsung Cloud service.
- 5) What appear to be hashes of the IMEIs of both SIM slots and of the hardware serial number are sent to eu-kaf.samsungknox.com, gslb.secb2b.com/KnoxGSLB/v2/lookup/knoxguard. Binary messages are also sent to eu-kaf.samsungknox.com, the contents of which are unclear (they base64 decode to binary in what seems to be a proprietary format). These connections appears to be associated with the Samsung Knox service.
- 6) The IMEI’s of both SIM slots, the hardware serial number and the Samsung device/consumer id are sent to www.ospserver.net/, which appears to be a Samsung server. Cell tower identifiers are sent that reveal the approximate device location. The Firebase authentication token for app com.wssyncmldm is also sent, allowing linkage of data collection by Samsung and Google Firebase.
- 7) The Google adid/rdid advertising id is sent to spapi-prd.samsungs.com/AdConfiguration. The connection seems to associated with the app com.samsung.android.dynamiclock.
- 8) Other Samsung servers to which data is sent in-

clude: fota-apis.samsungdm.com, ota-cloud-dn.ospserver.net, hub-odc.samsungapps.com/, as.samsungapps.com/, dms.ospserver.net, sdk.pushmessage.samsung.com.

- 9) Samsung system apps that log handset activity using Google Analytics include: com.samsung.android.app.omcagent,com.samsung.android.app.simplesharing,com.samsung.android.authfw,com.samsung.android.bixby.agent,com.samsung.android.kgclient,com.samsung.android.mobileservice,com.samsung.android.rubin.app,com.samsung.android.themestore,com.sec.android.app.billing,com.sec.android.app.samsungapps,com.wssyncmld,com.samsung.android.game.gamehome. The messages sent to Google/Firebase Analytics are encoded as protobufs. We decoded these by reconstructing the protobuf definition from the decompiled Firebase code. Examples are shown below, but the messages log user interaction, including screens/activities viewed plus duration and timestamp.
- 10) When a SIM is first inserted into the handset following a factory reset a connection is made to api.omc.samsungdm.com/v5/api/device/simChange that sends the hardware serial number, Samsung device/consumer id, the mobile operator MNC/MCC identifiers and the the operator name. In addition SIM details are sent to Google as observed in previous studies, see “Mobile Handset Privacy: Measuring The Data iOS and Android Send to Apple And Google”, Proc SECURE-COM 2021.
- 11) When browsing the Settings app connections are made to ie-odc.samsungapps.com, auth2.samsungosp.com, us-cd-gpp.mcsvc.samsung.com, us-rd.mcsvc.samsung.com. The com.samsung.android.themestore and com.samsung.android.samsungpass apps send telemetry to Google Analytics and com.samsung.android.samsungpass calls firebaseremoteconfig.googleapis.com. The Google adid/rdid advertising identifier is sent to us-api.mcsvc.samsung.com/.

Pre-installed Non-Samsung System Apps

- 1) *Mobile Operator SFR/Altice*. The particular handset used in these measurements was bought secondhand online and appears to originally be from French mobile operator SFR/Altice France. The pre-installed system apps include com.sfr.android.sfrjeux and com.altice.android.myapps. Both make use of Google Analytics and custom telemetry is also sent to sun-apps.sfr.com/reportusage. The app com.sfr.android.sfrjeux and com.altice.android.myapps apps send device details and a Firebase authentication token to https://sun-apps.sfr.com (so linking data collection by SFR/Altice and Google Firebase) as well as what appears to be a persistent device identifier. It also appears to attempt to transmit the Wifi SSID. The app com.altice.android.myapps additionally uses the Firebase Crashlytics and Remote Configuration services.

Note that neither of apps were ever opened on the device, and no popup or request to send data was observed.

- 2) *Google*. The following pre-installed Google system apps were observed to send data to Google.
  - a) Google Play Services and Google Play store make many connections to Google servers. These share persistent device and user identifiers with Google including the device hardware serial number, SIM IMEI, Wifi MAC address, SIM IMSI and phone number, user email (when logged in). A substantial quantity of data is sent, in particular, to play.googleapis.com/vn/log/batch, play.googleapis.com/play/log and www.googleapis.com/experimentsandconfigs. This is consistent with other recent measurement studies, see “Mobile Handset Privacy: Measuring The Data iOS and Android Send to Apple And Google”, Proc SECURE-COM 2021.
  - b) Google Youtube sends device data, including persistent identifiers and the Google adid/rdid advertising identifier and the AndroidID, to www.googleadservices.com and youtubei.googleapis.com. Youtube also uses Google Analytics to log events, and presumably also user interaction.
  - c) Connections are periodically made to www.google.com/complete/search. These are associated with the searchbar embedded in the handset UI and send a cookie which acts to link these connections to persistent device and user identifiers. Less frequent connections are made to www.google.com/m/voice-search/down that contain what appears to be a persistent device identifier.
  - d) The com.google.android.googlequicksearchbox app is associated with the search bar embedded in the handset UI. It sends telemetry data to Google Analytics that logs user interaction (screens/activities viewed plus duration and timestamp, etc).
  - e) When logged in to a Google account, connections are made to mail.google.com/mail/ads, inbox.google.com/sync and www.googleapis.com/calendar that send identifiers linked to the device and user account. Note that account login was carried out via the Google Play app only. Syncing of gmail, contacts, calendar took place without the user being asked or opting in.
  - f) When logged in to a Google account, connections are also made to instantmessaging-pa.googleapis.com, people-pa.googleapis.com, footprints-pa.googleapis.com. It's not clear what the purpose of these connections is or what data is sent.
  - g) When location is enabled additional connections are made to lamsettings-pa.googleapis.com and mobilenetworkscoring-pa.googleapis.com/v1/GetWifiQuality. The connection to mobilenetworkscoring-pa.googleapis.com/v1/GetWifiQuality hashes of the Wifi MAC addresses of nearby access points, used to query a network

quality database to determine the best Wifi network to connect to.

- h) Connections are made to www.gstatic.com/commerce/wallet/ but were not observed to contain persistent identifiers.
- i) Google Chrome makes connections to Google servers. These connections are consistent with previously documented behaviour, see “Web Browser Privacy: What Do Browsers Say When They Phone Home?”, IEEE Access. DOI 10.1109/ACCESS.2021.3065243.

Note that none of these apps were opened on the device, and no popup or request to send data was observed.

- 3) *Microsoft*. The pre-installed Microsoft system apps connect to mobile.pipe.aria.microsoft.com and app.adjust.com (which appears to be a third-party analytics company, their website says “Adjust offers a number of analytics tools designed to give you the deepest insight into your user interaction, your marketing channels, and your campaign performance”). The data sent to mobile.pipe.aria.microsoft.com includes device hardware and software details together with persistent device identifiers `DeviceInfo.Id`, `DeviceInfo.SDKUid`, `cai.device.id` and `TenantId`. The document <https://docs.microsoft.com/en-us/deployoffice/privacy/required-diagnostic-data-states>:

- a) `DeviceInfo.Id` - A unique device identifier to help us detect device-specific issues
- b) `DeviceInfo.SDKUid` - The device unique identifier (similar to `DeviceInfo.Id`)

The data sent to app.adjust.com includes device details, the Google `adid`/`rdid` advertising id of the handset and what appears to be a persistent device identifier that acts to link connections together. Connections are also made to config.edge.skype.com, skyapi.live.net, oneclient.sfx.ms. Note that no Microsoft apps were ever opened on the device, and no popup or request to send data was observed.

- 4) *LinkedIn*. A first connection is made to www.linkedin.com/mob/tracking that responds by setting `bcookie`, `bscookie` and `lidc` cookies. The linkedin document <https://www.linkedin.com/legal/l/cookie-table> says:

- a) `bcookie`: Browser Identifier cookie to uniquely identify devices accessing LinkedIn to detect abuse on the platform
- b) `bscookie`: Used for saving the state of 2FA of a logged in user
- c) `lidc`: To optimize data center selection

These cookies are resent in later requests to www.linkedin.com/li/track, along with `trackingId` values.

- 5) *Hiya*. Connections are made to samsung-directory.edge.hiyaapi.com/v3/trackedevents. This appears to be associated

with a third-party call management service, with connections made when making/receiving a call. Data sent includes the Google `adid`/`rdid` advertising id, an `X-Hiy-Installation-User-ID` and an authorization token that when decoded contains `hin` and `hui` values that appear to be persistent device identifiers.

- 6) *Google Analytics*. Several pre-installed system apps log handset activity using Google Firebase Analytics. These include: `com.google.android.apps.maps`, `com.microsoft.skydrive`, `com.android.vending`, `com.google.android.googlequicksearchbox`, `com.sfr.android.sfrjeux`, `com.altice.android.myapps`

A. Selected Connections During Startup After Factory Reset

HEAD <http://www.google.com/>

<<< HTTP 200, 0.00B

Set-Cookie: `NID=212=X_FV28cwxhIedC_a...NXdU`

Almost the first connection following the factory reset (earlier connections are e.g. to http://connectivitycheck.gstatic.com/generate_204 to check for network connectivity) sets a Google cookie.

GET <http://gllto1.glps.com/4day/v5/latest/ltto2.dat>

Accept: `*/`, `application/vnd.wap.mms-message`, `application/vnd.wap.sic`
 x-wap-profile: <http://www.openmobilealliance.org/tech/profiles/UAPROF/ccppschem-20021212#>

<<< HTTP 200, 168.30KB

This appears to be associated with Broadcom Assisted-GPS.

GET <https://config.edge.skype.com/config/v1/com.microsoft.skydrive/5.40.5?clientId=cff4bd4ddb34000b>

<<< HTTP 200, 8.35KB

First connection to a Microsoft server, early in the startup process. This connection sends a `clientId` value (the value is the secure settings `android_id`) that is also sent in later connections as a “`DeviceInfo.Id`” value and acts as a device identifier.

GET https://app-measurement.com/config/app/1%3A126578593765%3Aandroid%3Ab2244cc320147605?app_instance_id=d4b47dc0a35001516161c1141fed695a&platform=android&gmp_version=19629

<<< HTTP 200, 428.00B

First connection to Google Analytics. Based on the `app_instance_id` value this connection is associated with the app, which seems to be associated with the mobile operator Altice

POST <https://www.googleapis.com/androidantibuse/v1/x/create?alt=PROTOCOL&key=AIzaSyBof...wzOIz-ITI>

Headers

User-Agent: `DroidGuard/19629037`
First of a sequence of connections to Google’s DroidGuard/Safety Net service. Sends device details to Google, including the hardware serial number

GET https://oneclient.sfx.ms/mobile/ts_configuration.jwt

<<< HTTP 200, 15.34KB

The response to this request is a jwt encoded message that contains certificate details for skydrive, skydrive_certificate_chain, excel_word_powerpoint_outlook_ync, skype, wunderlist, shiftr_df, cortana, cortana_chain, launcher, launcher_chain, powerapp, bing_chain, cheshire, cheshire_chain, bingapps, bingapps_chain, yammer, yammer_chain, etc

POST <https://hub-odc.samsungapps.com/ods.as>

<?xml version="1.0" encoding="UTF-8" standalone="yes"?><SamsungProtocol networkType="0" deviceModel="SM-G960F-TM" mcc="" mnc="0" csc="SFR" odcVersion="5.1.02.305" odcType="02" OTFVersion="9000000" openApiVersion="29" lang="EN" version="6.3" version2="3" filter="1" sessionId=""><request id="772300" name="countrySearchExForTheme" numParam="3"><param name="latestCountryCode"></param><param name="whoAml">odc</param><param name="autoSelfUpgradeYN">Y</param></request></SamsungProtocol>

<<< HTTP 200, 1.19KB

Set-Cookie: JSESSIONID=rSqFFFTz6P...k7k.s04w0110dc03; path=/, SCOUTER=x76fqjdog2gp7a

This is the first connection to Samsung. It sends device details and the response sets a cookie and sends config information presumably based on geolocation of the handset IP address (namely that the country is IRL, currency is euro etc). The handset as no SIM installed here.

POST https://app-measurement.com/a

POST body decoded as protobuf:

```
<...>
8: "android"
9: "10"
10: "SM-G960F"
11: "fr-fr"
12: 60
13: "manual_install"
14: "com.altice.android.myapps"
16: "1.5.4"
17: 15300
18: 19629
19: "cfb8a087-1c38-4bb9-bc15-5f51001b8df1"
20: 0
21: "d4b47dc0a35001516161c1141fed695a"
22: 8442310499665198199
23: 1
25: "1:126578593765:android:b2244cc320147605"
28: 1
30: "egHfAlJvXwI"
31: 1543000
35: 1585956163239078
<...>
```

System app *com.altice.android.myapps* registering with Google Analytics. The value *cfb8a087-1c38-4bb9-bc15-5f51001b8df1* is the Google *ridid/adid* advertising identifier. The *d4b47dc0a35001516161c1141fed695a* value is the *app_instance_id* and the *egHfAlJvXwI* value is later sent as the *X-appid* header. Both seem to be persistent app instance identifiers.

Similar Google Analytics connections are made by a number of other pre-installed system apps, including: *com.google.android.apps.maps*, *com.microsoft.skydrive*, *com.samsung.android.app.omcagent*, *com.samsung.android.app.simplesharing*, *com.samsung.android.authfw*, *com.samsung.android.bixby.agent*, *com.samsung.android.kgclient*, *com.samsung.android.mobileservice*, *com.samsung.android.rubin.app*, *com.samsung.android.themestore*, *com.sec.android.app.billing*, *com.sec.android.app.samsungapps*, *com.sfr.android.sfrjeux*, *com.wssyncmlid*, *com.samsung.android.game.gamehome*, *com.android.vending*, *om.google.android.googlequicksearchbox*, *com.altice.android.myapps*

POST https://android.googleapis.com/checkin

This checkin connection has been documented elsewhere. It links together several device identifiers including the IMEI, hardware serial number and Wifi MAC address, and sends extensive details of the device hardware and software to Google.

POST https://mobile.pipe.aria.microsoft.com/Collector/3.0/

Content-Type: application/bond-compact-binary

POST body:

```
\xd8\xfa\x83L.j@yUR\x93\x5d<...>
Sends telemetry to Microsoft. The Bond Compact Binary format is a Microsoft data serialisation format. The schema is needed to decode Bond Compact Binary data. Bond works by compiling the schema to Java code, and so we decompiled the app, manually reconstructed the schema from the decompiled code and then compiled a C++ programme based on this reconstructed schema using Microsoft's Bond compiler to yield a decoder that can deserialise the observed POST payload data, then re-serialise to json so that its human readable. The Bond documentation is unhelpful, to say the least, so this was quite a painful process. After decoding we observed three main types of event payload. One is act_stats, e.g.
{"DataPackages":[],"RequestRetryCount":0,"TokenToDataPackagesMap":{"7434683b182f4b49bc52295c8152518d-e1d93d3d-7e05-4dd3-94d4-eb44bc27b601-7301"},{"Source":"act_default_source","Ids":[],"DataPackageId":"39f999f3-3773-4a6c-919f-03088dfb241c","Timestamp":1617194704207,"SchemaVersion":1,"Records":[{"Id":"b4bf7086-3261-4832-a234-bc0f2c11c76d","Timestamp":1617185943946,"Type":"custom","EventType":"act_stats","Extension":{"AppInfo.Language":"en-GB","AppInfo.Version":"5.40.5","DeviceInfo.Id":"cf4bd4ddb34000b","DeviceInfo.Make":"samsung","DeviceInfo.Model":"SM-G960F","DeviceInfo
```

```
.NetworkCost","Unknown","DeviceInfo.NetworkType","Unknown","DeviceInfo.OsBuild","G960FXXU8DTC5","DeviceInfo.OsName","Android","DeviceInfo.OsVersion","10","DeviceInfo.SDKUid","dac15d07-c41e-4121-b672-c46f1496da7c","EventInfo.InitId","9460f0b-7ef7-4e35-90b4-c628d4564b34","EventInfo.Name","act_stats","EventInfo.SdkVersion","ACT-Android-Java-no-3.0.12.0-ECS","EventInfo.Sequence","1","EventInfo.Source","act_default_source","EventInfo.Time","2021-03-31T10:19:03.946Z","S_e","ECS","S_j","no","S_k","Java","S_p","Android","S_t","ACT","S_v","3.0.12.0","TenantId","8ce6eedc33864b2f856e8ee4f9ec4190","UserInfo.Language","en-GB","UserInfo.TimeZone","+01:00","eventpriority","High","tr_p","r_t","RecordType":1,"PIIExtensions":{"TypedExtensionBoolean":{"TypedExtensionDateTime":{"TypedExtensionInt64":{"ino1","2","n_inol","2","normal_priority_records_received_count","2","records_received_count","2","t_h","1","t_l","4","t_n","2","t_p","2"},"TypedExtensionDouble":{"TypedExtensionGuid":{"CustomerContentExtensions":{"}}}}}}
This contains a number of identifiers, sends device details and count stats e.g. records_received_count, normal_priority_records_received_count. The DeviceInfo.Id value cf4bd4ddb34000b acts as a persistent device identifier (it is the secure settings android_id value and is also sent in a connection to config.edge.skype.com). Also the DeviceInfo.SDKUid value dac15d07-c41e-4121-b672-c46f1496da7ca. The document https://docs.microsoft.com/en-us/deployoffice/privacy/required-diagnostic-data-states:

```

- 1) *DeviceInfo.Id* - A unique device identifier to help us detect device-specific issues
- 2) *DeviceInfo.SDKUid* - The device unique identifier (similar to *DeviceInfo.Id*)

Also a *TenantId* value.

A second type of event logged is *qosmobile*, e.g.

```
{"DataPackages":[],"RequestRetryCount":0,"TokenToDataPackagesMap":{"8ce6eedc33864b2f856e8ee4f9ec4190-e3bcabfe-1bc4-440c-80a7-7e09c0a2d3e7-7331"},{"Source":"act_default_source","Ids":[],"DataPackageId":"5086c0b7-2924-4fd9-8e8e-c9978eb80289","Timestamp":1617194706179,"SchemaVersion":1,"Records":[{"Id":"b052b531-3361-4977-b1e7-5dd7a34f6bc7","Timestamp":1617185884504,"Type":"custom","EventType":"qosmobile","Extension":{"AppInfo.Id","OneDrive_Android","AppInfo.Language","en-GB","AppInfo.Version","5.40.5","BuildType","Prod","DeviceInfo.Id","cf4bd4ddb34000b","DeviceInfo.Make":"samsung","DeviceInfo.Model":"SM-G960F","DeviceInfo.NetworkCost","Unknown","DeviceInfo.NetworkType","Unknown","DeviceInfo.OsBuild","G960FXXU8DTC5","DeviceInfo.OsName","Android","DeviceInfo.OsVersion","10","DeviceInfo.SDKUid","dac15d07-c41e-4121-b672-c46f1496da7c","Environment","Unknown","EventCategory","CrashReporting/PreviousProcessDetected","EventInfo.InitId","675f52a5-4ecf-471a-b816-777527e4bda0","EventInfo.Name","qosmobile","EventInfo.SdkVersion","ACT-Android-Java-no-3.0.12.0-ECS","EventInfo.Sequence","1","EventInfo.Source","act_default_source","EventInfo.Time","2021-03-31T10:18:04.504Z","EventName","QoS/CrashReporting/PreviousProcessDetected","EventSchemaVersion","20","EventType","QoS","IsIntentional","0","Name","QoS/CrashReporting/PreviousProcessDetected","ResultCode","CrashReporting/PreviousProcessDetected","ResultType","Success","SampleRate","1","UserAgent","OneDrive for Android/5.40.5 (Android/10; en-GB; samsung/SM-G960F)","UserInfo.Language","en-GB","UserInfo.TimeZone","+01:00","ai.device.id","91750293-82bd-41bd-ba5b-8a9b7f0623a4","eventpriority","Normal","RecordType":1,"PIIExtensions":{"TypedExtensionBoolean":{"TypedExtensionDateTime":{"TypedExtensionInt64":{"TypedExtensionDouble":{"TypedExtensionGuid":{"CustomerContentExtensions":{"Id":"e9cbd963-01c1-4b0c-9277-fc16973b2c42","Timestamp":1617185884686,"Type":"custom","EventType":"usagemoible","Extension":{"AccessibilityIsCaptionsEnabled","false","AccessibilityIsColorInversionEnabled","false","AccessibilityIsEnabled","false","AccessibilityIsHighContrastTextEnabled","false","AccountOverQuotaDialog","true","AccountType","Unknown","AddToMru","-1","Adjust","true","AllPhotosExcludeNoThumbnailFile","true","AllPhotosInitialCountLimit","true","AllPhotosProjection","true","AllPhotosSuperZoom","true","AppInfo.Id","OneDrive_Android","AppInfo.Language","en-GB","AppInfo.Version","5.40.5","Aria","true","Audio","false","AuthenticatorIsTokenBroker","false","AutoUploadEnabled","false","Autoplay","false","BlockIAPForAmazon","true","BuildType","Prod","BusinessAccountOnPremise","true","CSLFolder","true","CameraBackupExperiments","false","CameraBackupQosTelemetry","true","CameraRollNestedFolderBusiness","true","CameraRollNestedFolderConsumer","false","CameraRollNestedFolderFetchTimeout","300000","CameraRollNestedFolderMonthOptionBusiness","false","CameraRollNestedFolderMonthOptionConsumer","false","Camera_Android
```

"true", "ChargerSetting", "false", "CheckOfferEligible", "false",
 CheckUploadStatus", "true", "Chromecast_Android", "false", "CleanUpSpace",
 true", "CloudAccounts_Android", "true", "CrashReportToFabric2", "false",
 CrashReportToHockeyApp", "true", "DataLossPreventionPolicyTips", "true",
 DefaultPushNotificationAction", "true", "DetectXplatDbCorruption", "true",
 DeviceInfo.Id", "c44b4d4db34000b", "DeviceInfo.Make", "samsung",
 DeviceInfo.Model", "SM-G960F", "DeviceInfo.NetworkCost", "Unknown",
 DeviceInfo.NetworkType", "Unknown", "DeviceInfo.OsBuild",
 G960FXXU8DTC5", "DeviceInfo.OsName", "Android", "DeviceInfo.
 OsVersion", "10", "DeviceInfo.SDKUid", "dac15d07-c41e-4121-b672-
 c46f1496da7c", "DisableRoboAlbums", "true", "DiscoverView2", "true",
 DiscoverViewLocalNotification", "true", "Dogfood", "false",
 ECSCConfigTesting4", "0", "EditTagsV2_Android", "false",
 EnableCrashSearchStatistics", "false", "EnableCrashTelemetry", "true",
 EnableMRUv2_1", "true", "EnableRiverflowGrouping", "true",
 EnvironmentManagementState", "NO_MANAGEMENT", "EventInfo.InitId",
 "675f52a5-4ecf-471a-b816-777527e4bda0", "EventInfo.Name",
 "usagemobile", "EventInfo.Sequence", "2", "EventInfo.Source", "act_default_source",
 EventInfo.Time", "2021-03-31T10:18:04.686Z", "EventName", "Legacy/
 AppState/ProcessStart", "EventSampleList", "Legacy/Auth/TokenRefresh",
 EventSchemaVersion", "20", "EventType", "Legacy", "FastScroller", "true",
 FileExtensionsSetting", "true", "FilesRepair_Android", "true",
 FilesUploadSection", "false", "Freemium", "true",
 GooglePlayServicesAvailable", "true", "HeaderSwitchSort", "true",
 IapRecoveryManager", "true", "InAppPurchases_Android", "true",
 InstalledOnSdCard", "false", "IntuneLogging", "true", "IsDataCleared", "false",
 IsIntentional", "0", "JoinOpenPublicBeta", "true", "LGOffer", "false",
 LegacyEventName", "AppState/ProcessStart", "LensSDKBulkMode", "false",
 LensSDKCropMagnifier", "true", "LensSDKInk", "true", "LensSDKPageLimit",
 "10", "LensSDKPhotoModeTheme", "true", "LensSDKScan", "true",
 LensSDKSnapToEdge", "true", "LensSDKTapToSelect", "true",
 LensSDKTelemetry", "false", "LensSDKTextStickers", "true",
 LocalFolderCovers", "false", "LocalPhotoVideoStreams", "1", "LogsToFabric",
 false", "LoopDetection", "true", "MAMAllowedAccounts", "true",
 MarqueeSelect", "true", "MassDeletePushNotificationAction", "1",
 MediaTAScanSetMetadata", "true", "MediaTAScanVault", "true",
 MediaTAScanWithClientToken", "false", "MediaTAScan_v4", "true",
 MobileNetworkSetting", "settings_wifi_only", "MultiPageDocScan", "true",
 MultiPageDocScanODB", "true", "Name", "Legacy/AppState/ProcessStart",
 NativeCrashReportToFabric", "false", "NetworkChoice", "Unset",
 NewExperienceForBeta", "true", "NewDocSaveAs", "true",
 NewSamsungFlow_v2", "0", "NewUiAB", "1", "NewUiSurvey", "false",
 NotificationChannels", "true", "NotificationsBlocked", "false",
 NotificationsHistory_Android", "true", "NotificationsSettings", "true",
 O365ChunkUploading", "true", "ODBCameraBackup", "true",
 ODBEmbeddedViewer_v1", "true", "ODBPhotosView", "true",
 ODBRemoveFromSharedList", "false", "ODCExpirationLinks", "true",
 OdbDocCreation", "false", "OdbFreUpsell", "false", "OdbGetChangesForShared",
 "true", "OdbNotifications", "true", "OdbSharingDialog", "true",
 OdbVRoomSharedWithMe2", "true", "OdcBundleSharing", "false",
 OdcHevcStreaming2", "true", "OdcSharingDialog", "false",
 OdcSharingGoPremium", "true", "OdcSharingLearnMore", "true",
 OdcWebSharingDialog", "1", "OfferExpirationNotifications", "true",
 OfficeLensScanV2", "true", "OfficePdfPreview", "true", "OfficePdfPreviewOdb",
 "true", "OfficeUpsellSamsungPromotion", "false", "OfflineFoldersBusiness",
 true", "OfflineFoldersPersonal", "true", "OfflineNotification_Android", "true",
 OnBoardingUI", "false", "OnThisDay", "false", "OneDriveApiChunkFileUpload",
 "false", "OneDriveApiFileDownload", "true", "OneDriveApiSingleFileUpload",
 "true", "OneRmCampaigns", "true", "OutlookUpsellSamsungPromotionAB",
 "0", "PDFLocalNotification", "true", "PdfCrossFade", "true", "PeopleCard",
 "true", "PhotosSearch", "true", "PhotosUploadSection", "true", "PowerLift", "true",
 "PreVersion1RestoreAccessToken", "false",
 PreVersion1RestoreAccessTokenUrl", "https://onedrive.live.com/?v=restore",
 PreinstallManufacturer", "Samsung", "ProjectZeroUpsell", "false",
 PurchaseSuccess2", "1", "QuotaPushNotificationAction", "true",
 RansomwareHandling", "true", "RecoverFromEmptyOwnerCid", "true",
 RefreshUIWhenOnePageSynced", "true",
 RemoveSecondCameraBackupDialog", "true", "RepositioningExperiment",
 "1", "RestoreOneDriveEnableCookies", "true", "RestoreOneDriveEntryPoint",
 "true", "ResyncWhenMetadataCorrupted2", "false",
 RetryWhenMediaTAServiceError", "true", "SampleRate", "1",
 SamplingNonReportingDevice", "true", "SamsungAllowlistedRamp",
 testRamp", "SamsungDeal", "true", "SamsungOfferId", "ProjectZeroPointOne",
 SamsungOfferUpsellExperimentEvent", "NA", "SamsungOfferYears", "1",
 SamsungStorageAmount", "100", "Scan_Android", "true", "SendFeedback",
 true", "ServiceDrivenPositioning", "true", "ShakeToSendFeedback", "true",

ShareCustomization_Android", "true", "SharingLink_ODB_Android", "true",
 ShowFileExtensionsSetting", "false", "ShowReauthInvalidToken", "true",
 SkyDriveViewModelMarch", "true", "Snackbar", "true", "SoloAnnual", "false",
 SonyOffer", "false", "SortExtensions", "true", "StreamingUploadWriteBack",
 false", "SuggestAnIdea", "false", "SwitchToModernRateDialog", "true",
 SyncAlternatePhotoFolders", "true", "SyncSignalOverError", "false",
 Team_Sites", "true", "ThrottleLoops", "true",
 ThumbnailAndStreamingTelemetry", "true", "ThumbnailLoadingSamplingRate",
 "500", "TitleBarSharingIcon", "1", "TryImageToDocWhenMediaTAFailed",
 true", "UpsellSharepoint", "false", "UseOneDriveApi", "false",
 UseUserCidForStreamCacheFolder", "true", "UserAgent", "OneDrive for
 Android/5.40.5 (Android/10; en-GB; samsung/SM-G960F)",
 UserEngagementState", "LOW", "UserInfo.Language", "en-GB", "UserInfo.
 TimeZone", "+01:00", "UserQuotaStateMessaging", "true", "VaultEnabled_V2",
 "true", "VaultFixTokenExpirationTime", "true", "VideoPlayerUseHLS", "true",
 "VideoUploadSetting", "true", "VroomVideoStreaming", "true", "WORKSITE",
 "true", "Week1RetentionNotification2", "true",
 Week1RetentionNotificationExperiment", "25", "WhiteboardSharing", "true",
 WriteBackSupport", "true", "XplatDetectInvalidToken", "true", "ai.device.id",
 "91750293-82bd-41bd-ba5b-8a9b7f0623a4", "ariaAIDataValidate", "9c7bb3cb-
 2d3b-4965-89fa-00b38c31a841", "eventpriority", "Normal", "telemetryType",
 "customEvent", "RecordType": "1", "PIIExtensions": [],
 TypedExtensionBoolean": [], "TypedExtensionDate": [],
 TypedExtensionInt64": [], "TypedExtensionDouble": [], "TypedExtensionGuid",
 "": [], "CustomerContentExtensions": [], "Id": "0d223013-86e2-43fd-8904-
 bb68e129d8fe", "Timestamp": "1617194703872", "Type": "custom", "EventType",
 "": "qosmobile", "Extension": "AppInfo.Id", "OneDrive_Android", "AppInfo.
 Language", "en-GB", "AppInfo.Version", "5.40.5", "BuildType", "Prod",
 DeviceInfo.Id", "c44b4d4db34000b", "DeviceInfo.Make", "samsung",
 DeviceInfo.Model", "SM-G960F", "DeviceInfo.NetworkCost", "Unknown",
 DeviceInfo.NetworkType", "Wifi", "DeviceInfo.OsBuild", "G960FXXU8DTC5",
 "DeviceInfo.OsName", "Android", "DeviceInfo.OsVersion", "10", "DeviceInfo.
 SDKUid", "dac15d07-c41e-4121-b672-c46f1496da7c", "Environment",
 "Unknown", "EventCategory", "CrashReporting/PreviousProcessDetected",
 EventInfo.InitId", "b21ec774-7477-4be5-a971-fd031e14efd8", "EventInfo.
 Name", "qosmobile", "EventInfo.SdkVersion", "ACT-Android-Java-no-
 -3.0.12.0-ECS", "EventInfo.Sequence", "1", "EventInfo.Source",
 "act_default_source", "EventInfo.Time", "2021-03-31T12:45:03.872Z",
 EventName", "QoS/CrashReporting/PreviousProcessDetected",
 EventSchemaVersion", "20", "EventType", "QoS", "IsIntentional", "0", "Name",
 "QoS/CrashReporting/PreviousProcessDetected", "ResultCode",
 CrashReporting/PreviousProcessDetected", "ResultType", "Success",
 SampleRate", "1", "UserAgent", "OneDrive for Android/5.40.5 (Android/10;
 en-GB; samsung/SM-G960F)", "UserInfo.Language", "en-GB", "UserInfo.
 TimeZone", "+01:00", "ai.device.id", "91750293-82bd-41bd-ba5b-
 8a9b7f0623a4", "eventpriority", "Normal", "RecordType": "1", "PIIExtensions",
 "": [], "TypedExtensionBoolean": [], "TypedExtensionDate": [],
 TypedExtensionInt64": [], "TypedExtensionDouble": [], "TypedExtensionGuid",
 "": [], "CustomerContentExtensions": [], "Id": "e0d3e236-9bab-49ea-84a9-
 b28a572d77d6", "Timestamp": "1617194704262", "Type": "custom", "EventType",
 "": "usagemobile", "Extension": "AccessibilityIsCaptionsEnabled", "false",
 AccessibilityIsColorInversionEnabled", "Unknown", "AccessibilityIsEnabled",
 "false", "AccessibilityIsHighContrastTextEnabled", "false",
 AccountOverQuotaDialog", "true", "AccountType", "Unknown", "AddToMr",
 "1", "Adjust", "true", "AllPhotosExcludeNoThumbnailFile", "true",
 AllPhotosInitialCountLimit", "true", "AllPhotosProjection", "true",
 AllPhotosSuperZoom", "true", "AppInfo.Id", "OneDrive_Android", "AppInfo.
 Language", "en-GB", "AppInfo.Version", "5.40.5", "Aria", "true", "Audio", "false",
 "AuthenticatorIsTokenBroker", "false", "AutoUploadEnabled", "false",
 Autoplay", "false", "BlockIAPForAmazon", "true", "BuildType", "Prod",
 BusinessAccountOnPremise", "true", "CSLFolder", "true",
 CameraBackupExperiments", "false", "CameraBackupQosTelemetry", "true",
 CameraRollNestedFolderBusiness", "true",
 CameraRollNestedFolderConsumer", "false",
 CameraRollNestedFolderFetchTimeout", "300000",
 CameraRollNestedFolderMonthOptionBusiness", "false",
 CameraRollNestedFolderMonthOptionConsumer", "false", "Camera_Android",
 "true", "ChargerSetting", "false", "CheckOfferEligible", "false",
 CheckUploadStatus", "true", "Chromecast_Android", "false", "CleanUpSpace",
 true", "CloudAccounts_Android", "true", "CrashReportToFabric2", "false",
 CrashReportToHockeyApp", "true", "DataLossPreventionPolicyTips", "true",
 DefaultPushNotificationAction", "true", "DetectXplatDbCorruption", "true",
 DeviceInfo.Id", "c44b4d4db34000b", "DeviceInfo.Make", "samsung",
 DeviceInfo.Model", "SM-G960F", "DeviceInfo.NetworkCost", "Unknown",
 DeviceInfo.NetworkType", "Wifi", "DeviceInfo.OsBuild", "G960FXXU8DTC5",
 "DeviceInfo.OsName", "Android", "DeviceInfo.OsVersion", "10", "DeviceInfo.
 SDKUid", "dac15d07-c41e-4121-b672-c46f1496da7c", "DisableRoboAlbums

```

    "true", "DiscoverView2", "true", "DiscoverViewLocalNotification", "true",
    "Dogfood", "false", "ECSConfigTesting4", "0", "EditTagsV2_Android", "false",
    "EnableCrashSearchStatistics", "false", "EnableCrashTelemetry", "true",
    "EnableMRUV2_1", "true", "EnableRiverflowGrouping", "true",
    "EnvironmentManagementState", "NO_MANAGEMENT", "EventInfo.InitId",
    "b21ec774-7477-4be5-a971-fd031e14ef88", "EventInfo.Name", "usagemobile",
    "EventInfo.SdkVersion", "ACT-Android-Java-no-3.0.12.0-ECS",
    "EventInfo.Sequence", "2", "EventInfo.Source", "act_default_source", "EventInfo",
    ".Time", "2021-03-31T12:45:04.262Z", "EventName", "Legacy/AppState/ProcessStart",
    "EventSampleList", "Legacy/Auth/TokenRefresh", "EventSchemaVersion", "20",
    "EventType", "Legacy", "FastScroller", "true", "FileExtensionsSetting",
    "true", "FilesRepair_Android", "true", "FilesUploadSection", "false",
    "Freemium", "true", "GooglePlayServicesAvailable", "true", "HeaderSwitchSort",
    "true", "IapRecoveryManager", "true", "InAppPurchases_Android", "true",
    "InstalledOnSdCard", "false", "IntuneLogging", "true", "IsDataCleared", "false",
    "IsIntentional", "0", "JoinOpenPublicBeta", "true", "LGOffer", "false",
    "LegacyEventName", "AppState/ProcessStart", "LensSDKBulkMode", "false",
    "LensSDKCropMagnifier", "true", "LensSDKInk", "true", "LensSDKPageLimit",
    "10", "LensSDKPhotoModeTheme", "true", "LensSDKScan", "true",
    "LensSDKSnapToEdge", "true", "LensSDKTapToSelect", "true",
    "LensSDKTelemetry", "false", "LensSDKTextStickers", "true",
    "LocalFolderCovers", "false", "LocalPhotoVideoStreams", "1", "LogsToFabric",
    "false", "LoopDetection", "true", "MAMAllowedAccounts", "true",
    "MarqueeSelect", "true", "MassDeletePushNotificationAction", "1",
    "MediaTAScanSetMetadata", "true", "MediaTAScanVault", "true",
    "MediaTAScanWithClientToken", "false", "MediaTAScan_v4", "true",
    "MobileNetworkSetting", "settings_wifi_only", "MultiPageDocScan", "true",
    "MultiPageDocScanODB", "true", "Name", "Legacy/AppState/ProcessStart",
    "NativeCrashReportToFabric", "false", "NetworkChoice", "Unset",
    "NewExperienceForBeta", "true", "NewOdcSaveAs", "true",
    "NewSamsungFlow_v2", "0", "NewUiAB", "1", "NewUiSurvey", "false",
    "NotificationChannels", "true", "NotificationsBlocked", "false",
    "NotificationsHistory_Android", "true", "NotificationsSettings", "true",
    "O365ChunkUploading", "true", "ODBCameraBackup", "true",
    "ODBEmbeddedViewer_v1", "true", "ODBPhotosView", "true",
    "ODBRemoveFromSharedList", "false", "ODCExpirationLinks", "true",
    "OdbDocCreation", "false", "OdbFreUpsell", "false", "OdbGetChangesForShared",
    "true", "OdbNotifications", "true", "OdbSharingDialog", "true",
    "OdbVRoomSharedWithMe2", "true", "OdcBundleSharing", "false",
    "OdcHevcStreaming2", "true", "OdcSharingDialog", "false",
    "OdcSharingGoPremium", "true", "OdcSharingLearnMore", "true",
    "OdcWebSharingDialog", "1", "OfferExpirationNotifications", "true",
    "OfficeLensScanV2", "true", "OfficePdfPreview", "true", "OfficePdfPreviewOdb",
    "true", "OfficeUpsellSamsungPromotion", "false", "OfflineFoldersBusiness",
    "true", "OfflineFoldersPersonal", "true", "OfflineNotification_Android", "true",
    "OnBoardingUI", "false", "OnThisDay", "false", "OneDriveApiChunkFileUpload",
    "false", "OneDriveApiFileDownload", "true", "OneDriveApiSingleFileUpload",
    "true", "OneRmCampaigns", "true", "OutlookUpsellSamsungPromotionAB",
    "0", "PDFLocalNotification", "true", "PdfCrossFade", "true", "PeopleCard",
    "true", "PhotosSearch", "true", "PhotosUploadSection", "true", "PowerLift", "true",
    "PreVersion1RestoreAccessToken", "false",
    "PreVersion1RestoreAccessTokenUrl", "https://onedrive.live.com/?v=restore",
    "PreinstallManufacturer", "Samsung", "ProjectZeroUpsell", "false",
    "PurchaseSuccess2", "1", "QuotaPushNotificationAction", "true",
    "RansomwareHandling", "true", "RecoverFromEmptyOwnerCid", "true",
    "RefreshUIWhenOnePageSynced", "true",
    "RemoveSecondCameraBackupDialog", "true", "RepositioningExperiment",
    "1", "RestoreOneDriveEnableCookies", "true", "RestoreOneDriveEntryPoint",
    "true", "ResyncWhenMetadataCorrupted2", "false",
    "RetryWhenMediaTAServiceError", "true", "SampleRate", "1",
    "SamplingNonReportingDevice", "true", "SamsungAllowListedRamp",
    "testRamp", "SamsungDeal", "true", "SamsungOfferId", "ProjectZeroPointOne",
    "SamsungOfferUpsellExperimentEvent", "NA", "SamsungOfferYears", "1",
    "SamsungStorageAmount", "100", "Scan_Android", "true", "SendFeedback",
    "true", "ServiceDrivenPositioning", "true", "ShakeToSendFeedback", "true",
    "ShareCustomization_Android", "true", "SharingLink_ODB_Android", "true",
    "ShowFileExtensionsSetting", "false", "ShowReauthInvalidToken", "true",
    "SkyDriveViewModelMarch", "true", "Snackbar", "true", "SoloAnnual", "false",
    "SonyOffer", "false", "SortExtensions", "true", "StreamingUploadWriteBack",
    "false", "SuggestAnIdea", "false", "SwitchToModernRateDialog", "true",
    "SyncAlternatePhotoFolders", "true", "SyncSignalOverError", "false",
    "Team_Sites", "true", "ThrottleLoops", "true",
    "ThumbnailAndStreamingTelemetry", "true", "ThumbnailLoadingSamplingRate",
    "500", "TitleBarSharingIcon", "1", "TryImageToDocWhenMediaTAFailed",
    "true", "UpsellSharepoint", "false", "UseOneDriveApi", "false",
    "UseUserCidForStreamCacheFolder", "true", "UserAgent", "OneDrive for

```

```

Android/5.40.5 (Android/10; en-GB; samsung/SM-G960F)",
    "UserEngagementState", "LOW", "UserInfo.Language", "en-GB", "UserInfo",
    "TimeZone", "+01:00", "UserQuotaStateMessaging", "true", "VaultEnabled_V2",
    "true", "VaultFixTokenExpirationTime", "true", "VideoPlayerUseHLS", "true",
    "VideoUploadSetting", "true", "VroomVideoStreaming", "true", "WORKSITE",
    "true", "Week1RetentionNotification2", "true",
    "Week1RetentionNotificationExperiment", "25", "WhiteboardSharing", "true",
    "WriteBackSupport", "true", "XplatDetectInvalidToken", "true", "ai.device.id",
    "91750293-82bd-41bd-ba5b-8a9b7f0623a4", "ariaAIDataValidate", "e1eddb42-
    abd0-47d8-93e4-c6fa5610b866", "eventpriority", "Normal", "telemetryType",
    "customEvent", "RecordType": "1", "PIIExtensions": [],
    "TypedExtensionBoolean": [], "TypedExtensionDateTime": [],
    "TypedExtensionInt64": [], "TypedExtensionDouble": [], "TypedExtensionGuid",
    "": [], "CustomerContentExtensions": []]]]]

```

which sends device details, configuration information and has an additional ai.device.id value that is set when the app is first started (i.e. following factory reset) and acts as a persistent identifier.

A third type of event observed logged is k2app, e.g.

```

{"DataPackages": [], "RequestRetryCount": 0, "TokenToDataPackagesMap": [{"0",
    "f7e2d7f1132433b82c0b49a3e7da349-48b984f1-c586-4e69-9a6b-
    3cb9597e0bc-7008", [{"Source": "act_default_source", "Ids": []}],
    "DataPackageId": "3c1d4463-033b-4da0-bdfe-07b769a69a4a", "Timestamp",
    "1617106647229", "SchemaVersion": 1, "Records": [{"Id": "e797726c-a0b4-43
    be-8bb0-f55635f83097", "Timestamp": "1617106645234", "Type": "custom",
    "Event": "k2app", "Extension": [{"AppInfo.Language": "en-GB", "AppInfo",
    "Version": "16.1.0.1", "DeviceInfo.Id": "a22c7065bd8902db", "DeviceInfo.Make",
    "samsung", "DeviceInfo.Model": "SM-G960F", "DeviceInfo.NetworkCost",
    "Unknown", "DeviceInfo.NetworkType": "Wifi", "DeviceInfo.OsBuild",
    "G960FXXU8DTC5", "DeviceInfo.OsName": "Android", "DeviceInfo",
    "OsVersion": "10", "DeviceInfo.SDKUid", "4cccbaf-f8ab-431b-af8f-
    b7c103deeddf", "EventInfo.InitId": "605ce793-51a1-48bd-b863-43
    ae8690239e", "EventInfo.Name", "k2app", "EventInfo.SdkVersion", "ACT-
    Android-Java-no-3.0.18.0-no", "EventInfo.Sequence", "1", "EventInfo.Source",
    "act_default_source", "EventInfo.Time", "2021-03-30T12:17:25.234Z",
    "UserInfo.Language", "en-GB", "UserInfo.TimeZone", "+01:00", "com.microsoft",
    ".appmanager_APPTYPE", "Unknown AppType", "com.microsoft.office",
    "excel_APPTYPE", "stub", "com.microsoft.office.excel_BOOTED", "No", "com",
    "microsoft.office.powerpoint_APPTYPE", "stub", "com.microsoft.office",
    "powerpoint_BOOTED", "No", "com.microsoft.office.word_APPTYPE", "stub",
    "com.microsoft.office.word_BOOTED", "No", "com.microsoft",
    "skydrive_APPTYPE", "stub", "eventpriority", "Normal", "k2appUID",
    "a9d68aae-c502-3dbc-b426-be07ed4941c4", "RecordType": "1", "PIIExtensions",
    "": [], "TypedExtensionBoolean": [], "TypedExtensionDateTime": [],
    "TypedExtensionInt64": [], "TypedExtensionDouble": [], "TypedExtensionGuid",
    "": [], "CustomerContentExtensions": []]]]]

```

which logs details of installed Microsoft apps.

```

POST https://vortex.data.microsoft.com/collect/v1
{"ver": "2.1", "name": "Microsoft.Windows.MobilityExperience.Agents",
    "ExpFeatureUsage": "time": "2021-08-20T12:41:36.305Z", "popSample",
    "100.0", "epoch": "4785621991705672117", "seqNum",
    "2", "iKey": "A-MMXSDK", "flags": "514", "os": "Android", "osVer": "10", "appId",
    "A:com.microsoft.appmanager", "appVer": "1.21072.151.0", "cV":
    "yUn+2yS7DUFJvCRGpYDfIT.0", "ext": {"user": {"ver",
    "1.0", "localId": ""}, "os": {"ver": "1.0", "locale": "en-IE"}, "device": {"ver",
    "1.0", "localId": "a:349083c4ed18d002", "deviceClass": "Android.Phone"},
    "android": {"ver": "1.0", "libVer": "3.
    171208.0"}}, "data": {"romeVersion": "1.6.1", "mmxAgentVersion",
    "1.21072.151.0", "ringName": "PRODUCTION", "isPreload": true, "model":
    "SM-G960F", "manufacturer": "samsung", "featureName":
    "YPPServicesBrokenCircuitWaitTime", "featureValue": "120000", "changeTime",
    "OnRead", "dataSource": "Default"}}

```

```

POST https://vortex.data.microsoft.com/collect/v1
{"ver": "2.1", "name": "Microsoft.Windows.MobilityExperience.Agents",
    "ExpResponseResult": "time": "2021-08-20T12:41:36.354Z", "popSample",
    "100.0", "epoch": "4785621991705672117", "seqN",
    "um": "3", "iKey": "A-MMXSDK", "flags": "257", "os": "Android", "osVer": "10",
    "appId": "A:com.microsoft.appmanager", "appVer": "1.21072.151.0", "cV":
    "yUn+2yS7DUFJvCRGpYDfIT.0", "ext": {"user": {"ve",
    "r": "1.0", "localId": ""}, "os": {"ver": "1.0", "locale": "en-IE"}, "device": {"ver",
    "1.0", "localId": "a:349083c4ed18d002", "deviceClass": "Android.Phone"},
    "android": {"ver": "1.0", "libVer":
    "3.171208.0"}}, "data": {"romeVersion": "1.6.1", "mmxAgentVersion",
    "1.21072.151.0", "ringName": "PRODUCTION", "isPreload": true, "model":
    "SM-G960F", "manufacturer": "samsung", "requestDurat

```

```
ionMS":783,"requestStatus":"Succeeded"}}
{"ver":"2.1","name":"Microsoft.Windows.MobilityExperience.Health.
RomeInitialization.DeviceRegistrarRomeAsyncInitActivity","time
":"2021-08-20T12:41:37.708Z","popSample":100.
0,"epoch":"-4785621991705672117","seqNum":9,"iKey":"A-MMXSDK","
flags":257,"os":"Android","osVer":"10","appId":"A:com.microsoft.
appmanager","appVer":"1.21072.151.0","cV":"yUn+2y
S7DUFJvCRGpYDfIT.0","ext":{"user":{"ver":"1.0","localId":"","os":{"
ver":"1.0","locale":"en-IE"},"device":{"ver":"1.0","localId":"a:349083
c4ed18d002","deviceClass":"Android.Pho
ne"},"android":{"ver":"1.0","libVer":"3.171208.0"},"data":{"romeVersion
":"1.6.0","ringName":"PRODUCTION","isPreload":false,"model":"SM-
G960F","manufacturer":"samsung","dim3":
"3.3.0-development.2107.29001","activityStatus":1,"result":0,"resultDetail":
"START_ASYNC","correlationId":"9837bd3a-6eed-4f6c-a5dc-
aad3953ca924","relatedId":"508c8e5a-fbae-4d12-
961d-a51199404b8d"}}}
{"ver":"2.1","name":"Microsoft.Windows.MobilityExperience.Health.
RomeInitialization.DeviceRegistrarRomeAsyncInitActivity","time
":"2021-08-20T12:41:37.738Z","popSample":100.
0,"epoch":"-4785621991705672117","seqNum":10,"iKey":"A-MMXSDK","
flags":257,"os":"Android","osVer":"10","appId":"A:com.microsoft.
appmanager","appVer":"1.21072.151.0","cV":"yUn+2
yS7DUFJvCRGpYDfIT.0","ext":{"user":{"ver":"1.0","localId":"","os":{"
ver":"1.0","locale":"en-IE"},"device":{"ver":"1.0","localId":"a:349083
c4ed18d002","deviceClass":"Android.Ph
one"},"android":{"ver":"1.0","libVer":"3.171208.0"},"data":{"romeVersion
":"1.6.0","ringName":"PRODUCTION","isPreload":false,"model":"SM-
G960F","manufacturer":"samsung","dim3":
"3.3.0-development.2107.29001","activityStatus":2,"result":3,"resultDetail":
"GET_NOTIFICATION_PROVIDER","correlationId":"9837bd3a-6eed-4f6c-
a5dc-aad3953ca924","relatedId":"508c
8e5a-fbae-4d12-961d-a51199404b8d"},"details":"Notification provider does
not have a valid token"}}}
{"ver":"2.1","name":"Microsoft.Windows.MobilityExperience.Health.
RomeInitialization.DeviceRegistrarRomeAsyncInitActivity","time
":"2021-08-20T12:41:38.811Z","popSample":100.
0,"epoch":"-4785621991705672117","seqNum":13,"iKey":"A-MMXSDK","
flags":257,"os":"Android","osVer":"10","appId":"A:com.microsoft.
appmanager","appVer":"1.21072.151.0","cV":"yUn+2
yS7DUFJvCRGpYDfIT.0","ext":{"user":{"ver":"1.0","localId":"","os":{"
ver":"1.0","locale":"en-IE"},"device":{"ver":"1.0","localId":"a:349083
c4ed18d002","deviceClass":"Android.Ph
one"},"android":{"ver":"1.0","libVer":"3.171208.0"},"data":{"romeVersion
":"1.6.0","ringName":"PRODUCTION","isPreload":false,"model":"SM-
G960F","manufacturer":"samsung","dim3":
"3.3.0-development.2107.29001","activityStatus":1,"result":0,"resultDetail":
"GET_NOTIFICATION_PROVIDER","correlationId":"4aec3f8e-0721-4793-
b812-d73c00a29de8","relatedId":"508c
8e5a-fbae-4d12-961d-a51199404b8d"}}}
```

GET https://gos-api.gos-gsp.io/v4/devices/startle
Headers

```
x-samsung-trace-id: 1-60630b89-17e1be6ae93a26a3f71507d5
user-agent: (gms_version:100.005;gos_version:300100025;device_name:
startle;model_name:SM-G960F;version_r:10;version_i:G960FXXU8DTC5;
uuid:c0a49953a5de45ea81f494f435fe8c99;installed_sec_game_family:com.
sec.android.app.samsungapps=enabled,com.samsung.android.game.
gamehome=enabled,com.samsung.android.game.gametools=disabled;
samsung_errorlog_agree:0)
gos-gsp.io appears to be a Samsung domain related to games (gos=game
optimisation service?). The uuid value c0a49953a5de45ea81f494f435fe8c99
links connections to this domain together and so acts as a device identifier.
```

GET https://www.googleadservices.com/pagead/conversion/1001680686/?
bundleid=com.google.android.youtube&appversion=14.47.50&osversion
=10&sdkversion=ct-sdk-a-v2.2.4&gms=1&lat=0&rrid=cfb8a087-1c38-
4bb9-bc15-5f51001b8df1&timestamp=1617103754.324&remarketing_only
=1&usage_tracking_enabled=0&data.screen_name=%3
CAndroid_YT_Open_App%3E
The rrId value here is the Google advertising identifier. This connection
appears to be the pre-installed Google Youtube app registering with the
Google ad server.

POST https://youtubei.googleapis.com/deviceregistration/v1/devices?key=

A1zaSyA8ei...yQ_vz_yYM39w&rawDeviceId=bc38110e74f1b6e5

Headers

```
user-agent: com.google.android.youtube/14.47.50(Linux; U; Android 10;
en_GB; SM-G960F Build/QP1A.190711.020) gzip
<<< HTTP 200, 233.00B
The pre-installed Youtube app also sends a device identifier to
youtubei.googleapis.com.
```

POST https://android.clients.google.com/c2dm/register3

Headers

```
Authorization: AidLogin 4513679355944265196:3426139596944398072
app: com.sfr.android.sfrjeux
X-subtype=637441263799&sender=637441263799&X-app_ver=7133000&
X-osv=29&X-cliv=fiid-12451000&X-gmsv=19629037&X-appid=
dwD2or6tu3s&X-scope=*&X-gmp_app_id=1%3A637441263799%3
Aandroid%3A659db5ceb13b5646&X-Firebase-Client=fire-android%2F+fire
-core%2F16.1.0&X-app_ver_name=7.1.3&app=com.sfr.android.sfrjeux&
device=4513679355944265196&app_ver=7133000&info=w-
QYjhNq2p0UQKri541rkWUINVuiBc&gcm_ver=19629037&plat=0&cert=
f051dd40028dc13...a08920c9&target_ver=28
<<< HTTP 200, 158.00B
```

This connection is the app com.sfr.android.sfrjeux registering with Google
Firebase. The value 4513679355944265196 of the device parameter is the
AndroidID (a persistent device identifier that requires a factory reset to
change). The X-appid value is a persistent identifier of the app
instance. Similar calls to Google Firebase are also made by:
com.google.android.youtube, com.google.android.gms,
com.samsung.android.app.omcagent, com.sec.android.app.samsungapp,
com.samsung.android.sdm.config, com.google.android.apps.maps,
com.android.vending, com.samsung.android.game.gamehome,
com.samsung.android.authfw, com.microsoft.skydrive,
com.altice.android.myapps, com.samsung.android.kgclient,
com.samsung.android.app.reminder, com.samsung.android.mobileservice,
com.samsung.android.sdm.config, com.google.android.googlequicksearchbox,
com.sec.spp.push, com.samsung.android.rubin.app,
com.samsung.android.themestore

POST https://api.omc.samsungdm.com/v5/api/device/registerDeviceInfo
Headers

```
Authorization: auth_identifier="OMC-AUTH",server_id="q95ikwe7b6",
device_id="227ae90a14017ece",signature="fh+VJ3UETemoZkT2ukY/
TFJM7ILLU9VvXQ1NeJviS7Mg="
User-Agent: samsung SM-G960F OMC-AGENT
{"carrierId":"SFR","clientVersion":"5.2.25","deviceId":"
227ae90a14017ece","deviceUN":"CE0218227AE90A14017E","emStatus
":"0","fingerPrint":"samsung/startltxx/startle:10/QP1A.190711.020/
G960FXXU8DTC5:user/release-keys","fwVersion":"G960FXXU8DTC5/
G960FOXJ8DTC5/G960FXXU8DTC5","modelName":"SM-G960F","
multiCscCode":"OXJ","omcEnable":"off","omcVersion":"SAOMC_SM-
G960F_OXJ_SFR_QQ_0007","osVersion":"10","salesCode":"SFR","
tMccCode":"","tMncCode":""}
<<< HTTP 200, 249.00B
```

Set-Cookie: WMONID=VmCnmYd4PTd

Sends device details to Samsung. These are tagged with persistent device
identifiers (the deviceId value 227ae90a14017ece is the hardware serial
number, the deviceUN value CE0218227AE90A14017E is a Samsung
device/user identifier). The response sets a cookie.

POST https://www.googleapis.com/affiliation/v1/affiliation:lookup?alt=proto
&key=A1zaSyA...XHqV_mXHhZIk

Headers

POST body decoded as protobuf:

```
1: "android://ABi2fbt8vkzj7SJ8aD...gh9w84O1Xgg==@com.samsung.
android.provider.filterprovider"
<list of all apps installed on handset plus signatures>
```

POST https://auditrecording-pa.googleapis.com/google.internal.api.
auditrecording.v1.AuditRecordingMobileService/CreateAuditRecord
Headers

```
x-goog-spatula: CjYKFmNvbS5nb.../uUBKGA=
POST body:
<device details>
```

GET https://www.youtube.com/csi_204?v=3&s=youtube_android&action=
process&yt_lt=cold&yt_fi=1&mod_li=0&conn=3&it=ndps.276.proc_k.-146,
app.1.353.f_proc.1100&cplatform=mobile&cbr=com.google.android.youtube
&c=android&cmodel=SM-G960F&cos=Android&cscdk=29&cbrver

SamsungProtocol>

<<< HTTP 200, 1.21KB

Set-Cookie: JSESSIONID=rSqFFFTz6PZ8X6XxWiGvpo7GbK4-L5wvtuFJsk7k.s04w0110dc03; path=/, SCOUTER=x76fqjd0g2gp7a
This is followed later by several similar connections. The sessionId and lgld values are repeated in later requests to ie-odc.samsungapps.com/ods.as, linking them together. The cookies seem to be scrubbed i.e are not resent.

POST https://app.adjust.com/session

Headers

Client=SDK: android4.11.0
 country=FR&api_level=29&hardware_name=QP1A.190711.020.
 G960FXXU8DTC5&event_buffering_enabled=0&app_version=5.40.5&
 app_token=wb0aq6rfd0qo&created_at=2021-03-30T12%3A14%3A56.998
 Z%2B0100&device_type=phone&language=fr&gps_adid=cfb8a087-1c38-4bb9-bc15-5f51001b8df1&device_manufacturer=samsung&display_width=1080&device_name=SM-G960F&needs_response_details=1&os_build=QP1A.190711.020&cpu_type=arm64-v8a&screen_size=normal&
 screen_format=long&os_version=10&vm_isa=arm&android_uuid=56005a2b-a7bd-4374-9409-314a4668c93f&environment=production&
 screen_density=high&attribution_deeplink=1&session_count=1&
 package_name=com.microsoft.skydrive&display_height=2076&os_name=android&tracking_enabled=1&sent_at=2021-03-30T12%3A29%3A24.012Z
 %2B0100&queue_size=4
 <<< HTTP 200, 84.00B

This is followed by several similar requests to app.adjust.com. This connection appears to be associated with app com.microsoft.skydrive. The gps_adid value is the Google adid/rdid advertising identifier. The android_uuid value appears to be a persistent device identifier that links requests to app.adjust.com together.

GET https://app.adjust.com/attribution?environment=production&
 needs_response_details=1&event_buffering_enabled=0&app_token=wb0aq6rfd0qo&attribution_deeplink=1&created_at=2021-03-30T12%3A29%3A22.578Z%2B0100&gps_adid=cfb8a087-1c38-4bb9-bc15-5f51001b8df1&tracking_enabled=1&sent_at=2021-03-30T12%3A29%3A26.276Z%2B0100
 <<< HTTP 200, 156.00B

GET https://www.linkedin.com/mob/tracking

<<< HTTP 200, 4.00B

Set-Cookie: bcookie="v=2&18241c78-09ac-4d7f-84c7-164f8c151745"; domain=linkedin.com; bscookie="v=1&202103301...dtRPWKOO4rxp"; domain=www.linkedin.com; lidc="b=TGST09:s=T:r=T:a=T:p=T:g=1984;u=1;i=1617103750;t=1617190150;v=2;sig=AQFCV4RXoAxiucp-3LPLVh6omCRPKLE9"; domain=linkedin.com;

No identifiers are sent, the purpose of this request seems to be to set cookies. The linkedin document https://www.linkedin.com/legal/cookie-table says:

- 1) bcookie: Browser Identifier cookie to uniquely identify devices accessing LinkedIn to detect abuse on the platform
- 2) bscookie: Used for saving the state of 2FA of a logged in user
- 3) lidc: To optimize data center selection

These cookies are resent by handset in later requests.

POST https://www.linkedin.com/li/track

Headers

Cookie: bcookie=v=2&18241c78-09...uicp-3LPLVh6omCRPKLE9
 [{"eventInfo":{"eventName":"AndroidAppActivationEvent","topicName":"AndroidAppActivationEvent","appId":"com.linkedin.flagshippreinstall.p_android"},"eventBody":{"header":{"memberId":-1,"time":"1617103754354","server":"","service":"","guid":"","pageInstance":{"pageUrn":"urn:li:page:p_flagshippreinstall_unknown"},"trackingId":"ogJU9kNTsq4AgQRCPRU9Q=="},"clientApplicationInstance":{"applicationUrn":"urn:li:application:(seed-stub-app,Stub)","version":"0.0.149","trackingId":"xsgcue0iQYe+6Qgyhy7NIw=="},"requestHeader":{"pageKey":"p_flagshippreinstall_unknown","interfaceLocale":"en_GB"},"mobileHeader":{"osName":"Android OS","osVersion":"10","deviceModel":"samsung_SM-G960F","appVersion":"1","isAdTrackingLimited":true,"appState":"APPLICATION_BACKGROUND","connectionType":"WIFI"},"rawReferrer":"oem_preinstall","activationState":"PRE_INSTALL_FIRST_BOOT"}]}]
 <<< HTTP 200, 0.00B

POST https://www.linkedin.com/li/track?nc=1629470133502

x-udid: 6603b920-344a-4411-8861-284b4b456c8b
 csrf-token: ajax:8941214435171228506

x-li-track: {"osName":"Android OS","osVersion":"29","clientVersion":"4.1.607","clientMinorVersion":151500,"model":"samsung_SM-G960F","displayDensity":3,"displayWidth":1080,"displayHeight":2076,"dpi":"xxhdpi","deviceType":"android","appId":"com.linkedin.android","deviceId":"6603b920-344a-4411-8861-284b4b456c8b","timezoneOffset":1,"timezone":"Europe/Dublin","storeId":"us_googleplay","isAdTrackingLimited":false,"mpName":"voyager-android","mpVersion":"0.683.36"}
 content-encoding: gzip
 x-li-lang: en_US
 user-agent: com.linkedin.android/151500 (Linux; U; Android 10; en-IE; SM-G960F; Build/QP1A.190711.020; Cronet/83.0.4103.83)
 accept-encoding: gzip, deflate
cookie: JSESSIONID=ajax:894121443517122...hGBu2OPqqsdbTKcZxqg9d

GET https://www.linkedin.com/voyager/api/configuration?nc=1629470036672

x-udid: 6603b920-344a-4411-8861-284b4b456c8b
 csrf-token: ajax:8941214435171228506
 x-li-page-instance: urn:li:page:p_flagship3_background;0
 TxanqhXS4yqelTVSs1cjQ==
 x-li-track: {"osName":"Android OS","osVersion":"29","clientVersion":"4.1.607","clientMinorVersion":151500,"model":"samsung_SM-G960F","displayDensity":3,"displayWidth":1080,"displayHeight":2076,"dpi":"xxhdpi","deviceType":"android","appId":"com.linkedin.android","deviceId":"6603b920-344a-4411-8861-284b4b456c8b","timezoneOffset":1,"timezone":"Europe/Dublin","storeId":"us_googleplay","isAdTrackingLimited":false,"mpName":"voyager-android","mpVersion":"0.683.36"}
 user-agent: com.linkedin.android/151500 (Linux; U; Android 10; en-IE; SM-G960F; Build/QP1A.190711.020; Cronet/83.0.4103.83)
 accept-encoding: gzip, deflate
cookie: JSESSIONID=ajax:894121443...u2OPqqsdbTKcZxqg9d

POST https://www.linkedin.com/sensorCollect/?action=reportMetrics&debug=false&nc=1629471543687 HTTP/2.0

x-udid: 6603b920-344a-4411-8861-284b4b456c8b
 csrf-token: ajax:8941214435171228506
 x-li-track: {"osName":"Android OS","osVersion":"29","clientVersion":"4.1.607","clientMinorVersion":151500,"model":"samsung_SM-G960F","displayDensity":3,"displayWidth":1080,"displayHeight":2076,"dpi":"xxhdpi","deviceType":"android","appId":"com.linkedin.android","deviceId":"6603b920-344a-4411-8861-284b4b456c8b","timezoneOffset":1,"timezone":"Europe/Dublin","storeId":"us_googleplay","isAdTrackingLimited":false,"mpName":"voyager-android","mpVersion":"0.683.36"}
cookie: JSESSIONID=ajax:894121443517...PqqsdbTKcZxqg9d
 POST body:
 action: reportMetrics
 debug: false
 nc: 162947154368

POST https://api.omc.samsungdm.com/v5/api/device/reportSUWComplete
 Headers

Authorization: auth_identifier="OMC-AUTH",server_id="q95ikwe7b6",device_id="227ae90a14017ece",signature="71/TPbDQ...5N6A1gY="

User-Agent: samsung SM-G960F OMC-AGENT

{"carrierId":"SFR","clientVersion":"5.2.25","deviceId":"227ae90a14017ece","deviceUN":"CE0218227AE90A14017E","emStatus":"0","fingerprint":"samsung/startltxx/startltx:10/QP1A.190711.020/G960FXXU8DTC5:user/release-keys","fwVersion":"G960FXXU8DTC5/G960FOXJ8DTC5/G960FXXU8DTC5","initType":"SUW","modelName":"SM-G960F","multiCscCode":"OXJ","omcEnable":"off","omcVersion":"SAOMC_SM-G960F_OXJ_SFR_QQ_0007","osVersion":"10","salesCode":"SFR","tMccCode":"","tMncCode":""}

<<< HTTP 200, 0.00B

Set-Cookie: WMONID=Nsj_tX75P9W

POST https://sun-apps.sfr.com/updateapp/v1

Headers

Authorization: Basic MS4wLjA6NzY3OTAYYjNlYjgxOGNjMA==

{"application":{"id":"com.altice.android.myapps","pushConnector":"firebase","pushSettings":{"pushToken":"egHfAlJvXwI:AP...Zsh1lpTku2_9Y8rr"},"versionCode":1543000,"versionName":"1.5.4"},"device":{"freeSpaceInMb":53661,"id":"25808231f441821b","lastReboot":"20210330110839Z","manufacturer":"samsung","name":"SM-G960F","playServicesVersion":"19.6.29","ramInMb

```

":3786,"totalSpaceInMb":55675,"version":1585545517000},"network":{"
bearer":"WIFI","operator":"","simCode":"00000","ssid":"<unknown ssid
>"},"os":{"name":"android","versionCode":29,"versionName":"10"},"ts
":"20210330113017Z"}
<<< HTTP 200, 175.00B

```

POST https://youtubei.googleapis.com/youtubei/v1/browse?key=AIzaSyA8eiZmM1FaDVjRy-df2KTyQ_vz_YM39w

Headers

```

x-goog-api-format-version: 2
x-goog-device-auth: device_id=AP+lc786CHuO...mg1/RDVg
x-goog-visitor-id: CgtKdFBMaz...Pl4yDBg\%3D\%3D
user-agent: com.google.android.youtube/14.47.50(Linux; U; Android 10;
en_GB; SM-G960F Build/QP1A.190711.020) gzip
POST body decoded as protobuf:
<device details>

```

POST <https://capi.samsungcloud.com/dp/v1/devicetoken>

Headers

```

Authorization: Basic
bnk2ZjJkNnBiZTpQUDBFR..PV1owMFBXMA==
{"device_id":"04d0902b87fb024468b93f313864b6efbe04d78f50","appid":"
ny6f2d6pbe","country":"IRL"}
<<< HTTP 200, 29.00B

```

The authorization header base64 decodes to ny6f2d6pbe:PP0EF4XRW7YRYLF8LQI07FEBOWZ00PW0. The device_id and appid values appear to be a persistent device identifiers. This connection is followed by many connections (~70) to capi.samsungcloud.com and then to d2ihp1a8eyb4of.cloudfront.net.

GET <https://pinning-02.secb2b.com/service/umc/leafcert>

```

<<< HTTP 200, 13.25KB

```

This appears to be Samsung Knox starting up.

POST <https://gslb.secb2b.com/KnoxGSLB/v2/lookup/knoxguard>

```

{"data":{"deviceid":"
qpFPcEeDZWJn4c5fnfKSr1OHDvF2cF2Fn2nnBmx1gA=","country_iso
":"FR","csc":"SFR"},"signature":"jLiNR02f...JWfhze1TbdJcx"}
<<< HTTP 200, 164.00B

```

The deviceid value appears to be a hash of the hardware serial number

POST <https://eu-kaf.samsungknox.com/af/v1/sal/devices/enhanced/start>

Headers

```

X-CLIENT-TRACE-ID: 0FD9BA49D54...C360F165
X-WSM-SERVICEID: af
{
  "deviceIdentifier": {
    "deviceId": null,
    "imeiHash1": "CuJczHjwZKBF11b...0UGxWo=",
    "imeiHash2": "c1qbNnHeX8lFWbG...tzayjpw=",
    "pbaUn": "
WMzRGqLTB6Cb5MyKKzlfGciV9tS0yBR+WrD112mC3fw=",
    "serialHash": "qpFPcEeDZWJn4c5f/nfKSr...mx1gA="
  },
  "requestType": "enroll"
}

```

This connection appears to send hashes of the IMEIs for both SIM slots and of the handset serial number. This is followed by a sequence of Knox connections.

POST <https://eu-kaf.samsungknox.com/af/v1/sal/devices/enhanced/deviate>

Headers

```

X-CLIENT-TRACE-ID: BB56123C5...0A6F116
X-WSM-SERVICEID: af
{
  "deviceMessage": "n/hGxgYOw9ZSvx7...VOBFksWsOo="
}
<<< HTTP 200, 723.00B

```

The contents of the deviceMessage in the POST body are unclear.

B. Connections When Phone Idle

GET <https://fota-apis.samsungdm.com/auth/time>

Headers

```

Authorization: oauth_nonce=2a33de25de,oauth_consumer_key=
j5p7lIlg33,oauth_signature_method=HmacSHA1,oauth_timestamp
=1617107246,oauth_version=1.0,oauth_signature=0vxfQEcd/2
iqMGc313XIPOG6etU=
X-Sec-Dm-DeviceModel: SM-G960F
x-osp-version: v1
User-Agent: SAMSUNG-Android
X-Sec-Dm-CustomerCode: SFR
<<< HTTP 200, 128.00B

```

POST <https://www.ospserver.net/device/fumo/device>

Headers

```

Authorization: oauth_nonce=c405351f0f,oauth_consumer_key=2
cbmvps5z4,oauth_signature_method=HmacSHA1,oauth_timestamp
=1617107243920,oauth_version=1.0,oauth_signature=ccoJIn9...cHl4r0=
X-Sec-Dm-DeviceModel: SM-G960F
User-Agent: SAMSUNG-Android
X-Sec-Dm-CustomerCode: SFR
<?xml version="1.0" encoding="UTF-8"?><FumoDeviceVO><
deviceUniqueID>IMEI:357171099697326</deviceUniqueID><
devicePhysicalAddressText>IMEI:357171099697326</
devicePhysicalAddressText><uniqueNumber>
CE0218227AE90A14017E</uniqueNumber><deviceSerialNumber>
227ae90a14017e</deviceSerialNumber><deviceTypeCode>PHONE
DEVICE</deviceTypeCode><deviceModelID>SM-G960F</
deviceModelID><deviceName>SM-G960F</deviceName><
customerCode>SFR</customerCode><mobileCountryCode>901</
mobileCountryCode><mobileNetworkCode>00</mobileNetworkCode><
mobileCountryCodeByTelephony><mobileNetworkCodeByTelephony><
deviceNetworkCellIDParams>GSM|48500181-30600</
deviceNetworkCellIDParams><terms>Y</terms><termsVersion>3.0</
termsVersion><firmwareVersion>G960FXXU8DTC5/G960FOXJ8DTC5/
G960FXXU8DTC5</firmwareVersion><fotaClientVer>3.4.24</
fotaClientVer><carrierID>SFR</carrierID><authenticateType>0</
authenticateType></FumoDeviceVO>
<<< HTTP 200, 456.00B

```

Set-Cookie: WMONID=mRztaCXg3zd

This connection links the IMEI's for both slots, the Samsung device/customer id, the hardware serial number and cell tower info (LAC and UCID, so rough location) together.

GET <https://fota-cloud-dn.ospserver.net/firmware/SFR/SM-G960F/version.xml>

Headers

```

User-Agent: SAMSUNG-Android
<<< HTTP 200, 1.41KB

```

POST <https://www.ospserver.net/device/fumo/ippushregister>

Headers

```

Authorization: oauth_nonce=84113ec845,oauth_consumer_key=2
cbmvps5z4,oauth_signature_method=HmacSHA1,oauth_timestamp
=1617107243922,oauth_version=1.0,oauth_signature=tn66qip/LC/
mSsM0MhTZjDGXVew=
X-Sec-Dm-DeviceModel: SM-G960F
x-osp-version: v1
User-Agent: SAMSUNG-Android
X-Sec-Dm-CustomerCode: SFR
<?xml version="1.0" encoding="UTF-8"?><PushInfoVO><deviceID>
IMEI:357171099697326</deviceID><pushType>FCM</pushType><
registrationID>dw9LlloPFD5c:APA91Mavu7YdF</registrationID></
PushInfoVO>
<<< HTTP 200, 4.00B

```

Set-Cookie: WMONID=NbgD6C0GdrO

The registrationID value is the Firebase authentication token for app com.wssyncml dm

GET <https://sspapi-prd.samsungrs.com/AdConfiguration?appid=com.samsung.android.dynamicclock&deviceModel=SM-G960F&gaid=cfb8a087-1c38-4bb9-bc15-5f51001b8df1>

Headers

```

x-mas-accesskeyid: 8932ad5e8d...618d223c608
x-mas-csc: SFR
<<< HTTP 400, 132.00B

```

This connection seems to be associated with the app com.samsung.android.dynamicclock. The gaid value is the Google adid/rdid advertising identifier

GET https://fota-cloud-dn.ospserver.net/firmware/SFR/SM-G960F/version.xml?px-nb=UGtezEZ854jbmFcvWGxLEA==&px-nac=RRoDsoxpJsUxDpOZ52Fu6GWAHt02CaeNtADmIECD3w=

Headers

User-Agent: SAMSUNG-Android

<<< HTTP 200, 1.41KB

The contents of the base64 px-nb and px-nac values is unclear.

POST https://dms.ospserver.net/v1/device/magicsync/mdm?sid=33ff257...5bf72a1

Headers

User-Agent: samsung SM-G960F SyncML DM Client

%02j%1d-%2f%2fSYNML%2f%2fDTD%20SyncML%201.2...hr%01S

%03org.openmobilealliance.dm.firmwareupdate.devicerequest%01%01O

%030%01%01%01%12%01%01

<<< HTTP 200, 494.00

Set-Cookie: WMONID=522i0qkn3pf

The POST body is XML binary encoded as WBXML. Decoding using libwbxml gives e.g.

<?xml version="1.0"?>

<DOCTYPE SyncML PUBLIC "-//SYNCML/DTD SyncML 1.2/EN" "

http://www.openmobilealliance.org/tech/DTD/OMA-TS-

SyncML_RepPro_DTD-V1_2.dtd">

<SyncML xmlns="SYNCML:SYNCML1.2">

<SyncHdr>

<VerDTD>1.2</VerDTD>

<VerProto>DM/1.2</VerProto>

<SessionID>81</SessionID>

<MsgID>5</MsgID>

<Target>

<LocURI>https://dms.ospserver.net/v1/device/magicsync/mdm/sc?sid=

33ff257...5bf72a1</LocURI>

</Target>

<Source>

<LocURI>IMEI:357171099697326</LocURI>

<LocName>IMEI:357171099697326</LocName>

</Source>

<Meta>

<MaxMsgSize xmlns="syncml:metinf">5120</MaxMsgSize>

<MaxObjSize xmlns="syncml:metinf">1048576</MaxObjSize>

</Meta>

</SyncHdr>

<SyncBody>

<Status>

<CmdID>1</CmdID>

<MsgRef>4</MsgRef>

<CmdRef>0</CmdRef>

<Cmd>SyncHdr</Cmd>

<TargetRef>IMEI:357171099697326</TargetRef>

<SourceRef>https://dms.ospserver.net/v1/device/magicsync/mdm/sc?sid=

33ff257...4c5bf72a1</SourceRef>

<Data>212</Data>

</Status>

<Status>

<CmdID>2</CmdID>

<MsgRef>4</MsgRef>

<CmdRef>2</CmdRef>

<Cmd>Replace</Cmd>

<TargetRef>./FUMO/DownloadAndUpdate/PkgURL</TargetRef>

<Data>200</Data>

</Status>

<Status>

<CmdID>3</CmdID>

<MsgRef>4</MsgRef>

<CmdRef>3</CmdRef>

<Cmd>Exec</Cmd>

<TargetRef>./FUMO/DownloadAndUpdate</TargetRef>

<Data>202</Data>

</Status>

<Status>

<CmdID>4</CmdID>

<MsgRef>4</MsgRef>

<CmdRef>4</CmdRef>

<Cmd>Replace</Cmd>

<TargetRef>./FUMO/Ext/DoCheckingRooting</TargetRef>

<Data>450</Data>

</Status>

<Status>

<CmdID>5</CmdID>

<MsgRef>4</MsgRef>

<CmdRef>5</CmdRef>

<Cmd>Replace</Cmd>

<TargetRef>./FUMO/Ext/Priority</TargetRef>

<Data>200</Data>

</Status>

<Status>

<CmdID>6</CmdID>

<MsgRef>4</MsgRef>

<CmdRef>6</CmdRef>

<Cmd>Replace</Cmd>

<TargetRef>./FUMO/Ext/Postpone</TargetRef>

<Data>200</Data>

</Status>

<Status>

<CmdID>7</CmdID>

<MsgRef>4</MsgRef>

<CmdRef>7</CmdRef>

<Cmd>Replace</Cmd>

<TargetRef>./FUMO/Ext/ForceInstall</TargetRef>

<Data>200</Data>

</Status>

<Status>

<CmdID>8</CmdID>

<MsgRef>4</MsgRef>

<CmdRef>8</CmdRef>

<Cmd>Replace</Cmd>

<TargetRef>./FUMO/Ext/DownloadConnType</TargetRef>

<Data>200</Data>

</Status>

<Final/>

</SyncBody>

</SyncML>

It can be seen that the message contains the handset IMEI. This is followed by several similar connections

GET http://vas.samsungapps.com/stub/stubUpdateCheck.as?appId=com.samsung.android.timezone.data_Q&callerId=com.samsung.android.timezone.updater&versionCode=100000000&deviceId=SM-G960F&mcc=&mnc=&csc=SFR&sdkVer=29&pd=0

<<< HTTP 302, 0.00B

Set-Cookie: JSESSIONID=6YDxCJa_V4I-

fMbPIWJOBmn8aDboVjn2li2q5hE-.aivas06.s05w06vas06; path=/,

SCOUTER=x2r15d1u1nut9v

This is followed by several similar connections, with changing appId and callerId parameter values, it seems to be checking for updates to system apps. The cookie set in the response seems to be scrubbed i.e. is not resent in later connections.

POST https://ie-odc.samsungapps.com/ods.as?reqId=2315

Headers

Cookie: JSESSIONID=xblg5hlCFqI8xV_Ze_D0MsJPlm-

gpVxHKABYfiom.s02w06odc01; SCOUTER=x1g7b2h9mm9i13

<?xml version="1.0" encoding="UTF-8" standalone="yes" ?><

SamsungProtocol networkType="0" version2="3" lang="EN"

openApiVersion="29" deviceModel="SM-G960F" deviceMakerName="

samsung" deviceMakerType="0" mcc="272" mnc="00" csc="SFR"

odcVersion="4.5.10.9" scVersion="1000000" storeFilter="

themeDeviceModel=SM-G960F_TM||OTFVersion=9000000"

supportFeature="ARW||AOD" version="6.3" filter="1" odcType="01"

systemId="1617102519146" sessionId="315e750ce202103311345" logId

=027528aa91d16f...850e" deviceFeature="locale=en_GB||abi32=armeabi-

v7a:armeabi||abi64=arm64-v8a"><request name="

getEmergencyDownloadList" id="2315" numParam="5" transactionId="315

e750ce001"><param name="extuk">a59839d085b95518</param><

param name="stduk">027528aa91d16f8de...c39d754850e</param><

param name="testMode">N</param><param name="imei">

357171099697326</param><param name="predeployed">0</param></

request></SamsungProtocol>

<<< HTTP 200, 248.47KB

Set-Cookie: JSESSIONID=

x3YIMME34qKscs7Qef9yFA13Ecdt2rpYiWKPpYk3.s02w03odc05; path=/

Sends the device IMEI. The cookie set in the response is echoed in the next connection to ie-odc.samsungapps.com.

POST https://sdk.pushmessage.samsung.com/v3/applications/aCie9ev7Sw/
smpid
{
"did": "357171099697326"
}<<< HTTP 200, 44.00B
The did value is the handset IMEI

POST https://sdk.pushmessage.samsung.com/v3/applications/aCie9ev7Sw/
clients/5a89e022b7686e1a0cf8258ac966d40f
{
"currentdts":1617124593525,"basic":{"initsts":0,"dce":{"FR":"","lc":{"en_GB","os":{"Android":"","osv":{"29","mcc":"","nmcc":"","mnc":"","nmnc":"","model":{"SM-G960F"},"sdv":{"3.0.4"},"appv":{"3.0.14"},"channel":{"notice":true,"marketing":true},"confv":-1,"pid":{"eW0y0wXQNs4:APA...EYSyyYko1"},"ptype":{"fcm"},"uid":"","optin":true,"optintime":1617124591470}}}
}<<< HTTP 200, 139.00B
The pid value is the Firebase authentication token associated with app com.sec.spp.push

GET https://gos-api.gos-gsp.io/v4/gos/devices/starlte/policy?os_sdk_version=29&gms_version=100.005&gos_version=300100025

Headers
x-samsung-trace-id: 1-6063b297-4e5034adf7dde0662f8f82d2
user-agent: (gms_version:100.005;gos_version:300100025;device_name:starlte;model_name:SM-G960F;version_r:10;version_i:G960FXXU8DTC5;uuid:c0a49953a5de45ea81f494f435fe8c99;installed_sec_game_family:com.sec.android.app.samsungapps=enabled,com.samsung.android.game.gamehome=enabled,com.samsung.android.game.gametools=disabled;samsung_errorlog_agree:0)
<<< HTTP 200, 2.63KB
This is followed by several similar connections

GET https://pinning-02.sec2b2b.com/service/umc/leafcert
<<< HTTP 200, 8.06k
Response is a set of SSL certs.

POST https://eu-kaf.samsungknox.com/af/v1/sal/devices/enhanced/start
Headers
X-CLIENT-TRACE-ID: F70C49836CFF2C1969B744C46A05F6BB28
X-WSM-SERVICEID: af
{
"deviceIdentifier": {
"deviceId": null,
"imeiHash1": "CuJczHjwZKBF1b...PVNAiP0UGxWo=",
"imeiHash2": "c1qbNnHeX8fW...qJgT1Fr54tzayjpw=",
"pbaUn": "WMzRGqLTB6Cb5M...BR+Wrd112mC3fw=",
"serialHash": "qpFPcEeDZWJ...2cF2Fn2nnBmx1gA="},
"requestType": "enroll"
}
<<< HTTP 404, 2.04KB

POST https://eu-kaf.samsungknox.com/af/v1/sal/devices/enhanced/start
Headers
X-CLIENT-TRACE-ID: 9F7D93C7DA3DE52297C3228ED5363A19DE
X-WSM-SERVICEID: af
{
"deviceIdentifier": {
"deviceId": null,
"imeiHash1": "CuJczHjwZKBF1b...PVNAiP0UGxWo=",
"imeiHash2": "c1qbNnHeX8fW...qJgT1Fr54tzayjpw=",
"pbaUn": "WMzRGqLTB6Cb5MyKK...R+Wrd112mC3fw=",
"serialHash": "qpFPcEeDZWJn4...vF2cF2Fn2nnBmx1gA="},
"requestType": "deviate"
}
<<< HTTP 404, 2.04KB

POST https://eu-kaf.samsungknox.com/af/v1/sal/devices/enhanced/deviate
Headers
X-CLIENT-TRACE-ID: 9F7D93C7DA3DE52297C3228ED5363A19DE
X-WSM-SERVICEID: af
{
"deviceMessage": "DEKtXrxTregmtjgUbzOpqPK/jz0...E7O1I5lpH3ffM="}
}<<< HTTP 200, 723.00B
POST https://eu-kaf.samsungknox.com/af/v1/sal/devices/enhanced/finish
Headers
X-WSM-REPEAT: true
X-CLIENT-TRACE-ID: 9F7D93C7DA3DE52297C3228ED5363A19DE
X-WSM-BINARYVERSION: G960FXXU8DTC5

X-WSM-TENANTID:
{
"agentVersion": "2.2.28",
"deviceIdentifier": {
"deviceId": null,
"imeiHash1": "CuJczHjwZKBF1b...PVNAiP0UGxWo=",
"imeiHash2": "c1qbNnHeX8fW...qJgT1Fr54tzayjpw=",
"serialHash": "qpFPcEeDZW...2cF2Fn2nnBmx1gA="},
"deviceMessage": null,
"kgLockscreen": null,
"pushId": null,
"requestType": "deviate",
"vkDetailErrorCode": null,
"vkState": "Checking"
}
<<< HTTP 200, 2.00B

POST https://dir-apis.samsungdm.com/api/v1/device/mktInfoSubscription
Headers
Authorization: consumer_id="IMEI:357171099697326", signature="IfwGiVY...Da9pAtDA=="
<?xml version='1.0' encoding='UTF-8' standalone='yes' ?>
<deviceVO>
"actType">SU</actType>
"agreementFlag">N</agreementFlag>
"clientVersion">1110100003</clientVersion>
"date">2021/03/30 11:18:04</date>
"deviceId">IMEI:357171099697326</deviceId>
"deviceModelName">SM-G960F</deviceModelName>
"mcc">235</mcc>
</deviceVO>
<<< HTTP 200, 0.00B
Set-Cookie: WMONID=GdBORLhcN7W

HEAD https://youtubei.googleapis.com/generate_204
Headers
user-agent: com.google.android.youtube/1447503000 (Linux; U; Android 10; en_GB; SM-G960F; Build/QP1A.190711.020; Cronet/80.0.3955.6)
<<< HTTP 204, 0.00B
GET https://www.googleadservices.com/pagead/conversion/1001680686/?
bundleid=com.google.android.youtube&appversion=14.47.50&osversion=10&sdversion=ct-sdk-a-v2.2.4&gms=1&lat=0&rdid=cfb8a087-1c38-4bb9-bc15-5f51001b8df1&timestamp=1617185042.374&remarketing_only=1&usage_tracking_enabled=0&data.screen_name=%3CAndroid_YT_Open_App%3E
<<< HTTP 200, 0.00B

GET https://www.youtube.com/csi_204?v=3&s=youtube_android&action=process&yt_it=cold&yt_fi=0&mod_li=0&conn=3&it=ndps.245.proc_k.-115.ndpe.290.app_1.325.f_proc.484&cplatform=mobile&cbr=com.google.android.youtube&c=android&cmodel=SM-G960F&cos=Android&csdk=29&cbrver=14.47.50&cver=14.47.50&cosver=10&cbrand=samsung&proc=8
Headers
x-goog-visitor-id: CgtKdFBMazNBdVBTQSiPl4yDBg\%3D\%3D
user-agent: com.google.android.youtube/14.47.50(Linux; U; Android 10; en_GB; SM-G960F Build/QP1A.190711.020) gzip
<<< HTTP 204, 0.00B

POST https://youtubei.googleapis.com/youtubei/v1/account/get_setting?key=AlzaSyA8eiZmM...yQ_vz_YM39w
Headers
x-goog-api-format-version: 2
x-goog-device-auth: device_id=AP+lc786CHuO...BzQwe0RIBeMA
x-goog-visitor-id: CgtKdFBMazNBdVBTQSiPl4yDBg\%3D\%3D
user-agent: com.google.android.youtube/14.47.50(Linux; U; Android 10; en_GB; SM-G960F Build/QP1A.190711.020) gzip
POST body decoded as protobuf:
<device details>
25: "4513679355944265196" //the AndroidID
<...>

GET https://config.edge.skype.com/config/v1/com.microsoft.skydrive/5.40.5?
clientId=cf4bd4ddb34000b
<<< HTTP 200, 8.33KB

GET https://oneclient.sfx.ms/mobile/ts_configuration.jwt

<<< HTTP 304, 0.00B

POST https://mobile.pipe.aria.microsoft.com/Collector/3.0/

Content-Type: application/bond-compact-binary
See above for description of data sent.

POST https://app.adjust.com/event

country=GB&api_level=29&hardware_name=QP1A.190711.020.
G960FXXU8DTC5&event_buffering_enabled=0&app_version=5.40.5&
app_token=wb0aq6rfd0qo&event_count=5&session_length=866&created_at
=2021-03-31T11%3A18%3A04.861Z%2B0100&device_type=phone&
language=en&gps_adid=cfb8a087-1c38-4bb9-bc15-5f51001b8df1&
device_manufacturer=samsung&display_width=1080&time_spent=866&
event_token=3xbva7&device_name=SM-G960F&needs_response_details
=1&os_build=QP1A.190711.020&cpu_type=arm64-v8a&screen_size=
normal&screen_format=long&subsession_count=1&os_version=10&vm_isa=
arm&android_uuid=56005a2b-a7bd-4374-9409-314a4668c93f&environment
=production&screen_density=high&attribution_deeplink=1&session_count
=1&package_name=com.microsoft.skydrive&display_height=2076&os_name
=android&tracking_enabled=1&sent_at=2021-03-31T13%3A45%3A15.934
Z%2B0100&queue_size=1
<<< HTTP 500, 58.00B

This is followed by several similar connections

POST https://skyapi.live.net/API/2/RedeemSpecialOffer

Headers

appid: 1276168582
{ "body": "ODAYnYaEN0O2RhIMyY0TYBVYzz...MfH\nPq7QhtU=\n","init
": "jSWIJwlcVAqtqrd4k02VNw==\n"}
<<< HTTP 200, 65.00B

GET https://www.google.com/complete/search?oe=utf-8&safe=images&gcc
=ie&ctzn=Europe%2FDublin&ctf=1&v=10.85.11.21.arm64&ntyp=1&
ram_mb=3610&ar=0&inn=asst&hl=en-GB&noj=1&client=qs-b-android-
asbl-pb&qsubts=1617110160317&devloc=0&padt=200&padb=692&cs=0&
cds=4&gs_pcr=t&q=&cp=0&psi=W3Uqb0heQWU.1617110160324.0&ech
=0&dpr=3.0&gs_pcr=1&xssi=t&getexp=1

Headers

x-client-data: au4DH4sIAAAAAAAAAAA3O8U...
RTjeBp_2Mof_4ptJuuhmLNaugK0a3MOXgCAAA
cookie: CONSENT=PENDING+027

NID=212=gZkhf2r5Dp8p...xhRutyM

<<< HTTP 200, 19.05KB

The psi value appears to be a persistent device identifier that links together connections to www.google.com/complete/search. A cookie is sent that links this connection to the earlier Google checkin connection and so to multiple persistent device and user identifiers. This connection is followed by several similar connections

POST https://www.google.com/m/voice-search/down?pair=65b1ca92-ecaf-43a4-8d6f-82401eae9494

POST body: 9KB binary but looks like device details and telemetry

<<< HTTP 200, 15.13KB

GET https://app-measurement.com/config/app/1%3A1086610230652%3A
Aandroid%3A131e4c3db28fca84?app_instance_id=
ea750a113af6b275cff7a19ef1d0c5ab&platform=android&gmp_version
=210915

<<< HTTP 304, 0.00B

POST https://app-measurement.com/a

POST body decoded as protobuf:

<device details>

14: "com.google.android.googlequicksearchbox"
16: "10.85.11.21.arm64"
17: 20005
18: 210915
19: "cfb8a087-1c38-4bb9-bc15-5f51001b8df1"
20: 0
21: "ea750a113af6b275cff7a19ef1d0c5ab"
22: 12815780039134672363
23: 3
25: "1:1086610230652:android:131e4c3db28fca84"
26: 1617137403864
<telemetry>
30: "cw1U7RyDat4"
<...>

}

<<< HTTP 204, 0.00B

GET https://www.gstatic.com/commerce/wallet/20110109/

jhfae70rio980yhbns0x6vkc9sjkdcuy223hns08udmnnds8776vp6n5744ghopeewdx
/lottie/Y29yZG90b2tld9ZlY3Rvcg/gpay_to_nfc_token_selector_2.json
<<< HTTP 200, 24.06KB

POST https://samsung-directory.edge.hiYaapi.com/v3/track_events

Headers

X-Hiya-Request-Id: 90b24251-685...620f789fd0ae
X-Hiya-Device-Info: samsung/SM-G960F
X-Hiya-OS-Info: Android29/G960FXXU8DTC5
X-Hiya-Device-Locale: en-GB
x-seq_id: 12846521-bf4...362439e3e
X-Hiya-Date: 1617356221509
X-Hiya-Product-Name: Samsung
X-Hiya-Installation-User-ID: 74da709a-76..e055a3720
X-Hiya-Product-Version-Code: 20509
X-Hiya-User-TZ-Offset-Seconds: 3600
X-Hiya-Device-User-ID: b527702066a24291
X-Hiya-Samsung-Sales-Code: SFR
X-Hiya-Country-Code: IE
X-Hiya-Product-AAR-Version: 2.5.6-145
X-Hiya-Product-Version: 2.5.9-samsung-414
Accept-Language: en-GB
X-Hiya-Advertising-ID: cfb8a087-1c3...5f51001b8df1
Authorization: Bearer

eyJraWQioiJDMEI0NzV...IdMqBnlsTF383R24f2w

{ "trackEvents": { "eventCreatedNanoId": 29754077439, "eventCreatedTime": "2021-04-02T09:37:01.498Z", "eventId": "5d986043-3cc8-49b1-84cb-b3297a5b43a3", "eventSentNanoId": 29758170670, "eventType": "View", "properties": { "trigger": { "stringValue": "SC_ShowNotification" } }, "screen": "Notification" } }
<<< HTTP 200, 0.00B

The X-Hiya-Advertising-ID value is the Google adid/rdid advertising id. The Authorization: Bearer header value is a jwt token that decodes as:

"aud": [
"smartcall.edge.hiyaapi.com",
"places.edge.hiyaapi.com",
"samsung-phones.edge.hiyaapi.com",
"samsung-ingestion.edge.hiyaapi.com",
"samsung-directory.edge.hiyaapi.com",
"samsung-callerprofile.edge.hiyaapi.com"
],
"exp": 1617870564,
"iat": 1617262634,
"hgt": "hiya_grant",
"hpn": "Samsung",
"hin": "74da709a-76ef-45d7-91e8-6b9e055a3720",
"has": [],
"hui": "7c2599e2-b655-40da-bfe0-fc5924509bdb"

POST https://sun-apps.sfr.com/initapp/v1

Headers

Authorization: Basic MS4wLjA6NzY3OTAyYjNIYjgxOGNjMA==
{ "application": { "id": "com.altice.android.myapps", "pushConnector": "firebase", "pushSettings": { "7": "pushToken" }, "egHfAlJvXwI:APA91bES2yYI3Ug...1lpTku2_9Y8rr-","versionCode": 1543000, "versionName": "1.5.4", "device": { "freeSpaceInMb": 53179, "id": "25808231f4418216", "lastReboot": "20210330110834Z", "manufacturer": "samsung", "name": "SM-G960F", "playServicesVersion": "21.09.15", "ramInMb": 3786, "totalSpaceInMb": 55675, "version": "1585545517000", "network": { "bearer": "WIFI", "operator": "", "simCode": "27211", "ssid": "<unknown ssid>", "os": { "name": "android", "versionCode": 29, "versionName": "10", "ts": "20210331165829Z" } } }
<<< HTTP 200, 832.00B

POST https://sun-apps.sfr.com/reportusage/v1

Headers

Authorization: Basic MS4wLjA6NzY3OTAyYjNIYjgxOGNjMA==
{ "application": { "id": "com.altice.android.myapps", "pushConnector": "firebase", "pushSettings": { "7": "pushToken" }, "egHfAlJvXwI:APA...Zsh1lpTku2_9Y8rr-","versionCode": 1543000, "versionName": "1.5.4", "device": { "freeSpaceInMb": 53179, "id": "25808231f4418216", "lastReboot": "20210330110834Z", "manufacturer": "samsung", "name": "SM-G960F", "playServicesVersion": "21.09.15", "ramInMb": 3786,

```
totalSpaceInMb":55675,"version":"1585545517000"},"network":{"bearer":"WIFI","operator":"","simCode":"27211","ssid":"<unknown ssid>"},"os":{"name":"android","versionCode":29,"versionName":"10"},"sessions":[{"application":{"id":"com.altice.android.myapps","versionCode":1543000,"versionName":"1.5.4"},"duration":106988,"tags":[{"ts":"20210330205025Z","type":"core","key":"first_wakeup_error","value":"NO_SIM"}]}]}<stack traces for com.altice.android.myapps>
```

GET http://apps.inovatel.cdn.sfr.net/ANDROID_APK/com.altice.android.myapps/MyApps-1.6.0-logoff-obson-prodMyAppsRelease.apk
Headers

User-Agent: AndroidDownloadManager/10 (Linux; U; Android 10; SM-G960F Build/QP1A.190711.020)
<<< HTTP 200, 18.74MB

POST <https://firebaseinstallations.googleapis.com/v1/projects/my-apps-5bda1/installations>
Headers

X-Android-Package: com.altice.android.myapps
x-goog-fis-android-iid-migration-auth:
egHfAlJvXwI:APA91bES2y...lpTku2_9Y8rr-
{"fid":"egHfAlJvXwI","appId":"1:126578593765:android:b2244cc320147605","authVersion":"FIS_v2","sdkVersion":"a:16.3.2"}
<<< HTTP 200, 536.00B

GET https://firebase-settings.crashlytics.com/spi/v2/platforms/android/gmp/1:126578593765:android:b2244cc320147605/settings?instance=374c0ba4abb3fedecca347e71ef2ded37597b37b&build_version=1603000&display_version=1.6.0&source=4
Headers

x-crashlytics-device-model: samsung/SM-G960F
x-crashlytics-installation-id: **d52294546738443bab497a006e17ae67**
x-crashlytics-google-app-id: 1:126578593765:android:b2244cc320147605
<<< HTTP 200, 420.00B
1:126578593765:android:b2244cc320147605 is google id of com.altice.android.myapps

POST <https://firebase-remoteconfig.googleapis.com/v1/projects/126578593765:namespaces/fireperf:fetch>
Headers

X-Android-Package: com.altice.android.myapps
X-Goog-Firebase-Installations-Auth:
eyJhbGciOiJFUzI1NiIsInR5cCI6IiwWwIpcnkbVGJd
{"appInstanceId":"egHfAlJvXwI","appVersion":"1.6.0","countryCode":"GB","analyticsUserProperties":{"appId":"1:126578593765:android:b2244cc320147605","platformVersion":"29","timeZone":"Europe/Dublin","sdkVersion":"19.2.0","packageName":"com.altice.android.myapps","appInstanceIdToken":"eyJhbGciOiJFUzI1NiIsInR5cCI6IiwWwIpcnkbVGJd","languageCode":"en-GB"}
<<< HTTP 200, 1.14KB
X-Goog-Firebase-Installations-Auth value is a jwt token that decodes to give "fid": "egHfAlJvXwI". Similarly appInstanceIdToken value. This is the Firebase Id of com.altice.android.myapps

POST <https://sun-apps.sfr.com/initapp/v1>
Headers

Authorization: Basic **MS4wLjA6NzY3OTAyYjNIYjgxOGNjMA==**
{"application":{"id":"com.altice.android.myapps","pushConnector":"firebase","pushSettings":{"pushToken":"egHfAlJvXwI:APA..._9Y8rr-","versionCode":1603000,"versionName":"1.6.0"},"device":{"freeSpaceInMb":53147,"id":"25808231f4418216","lastReboot":"20210330110834Z","manufacturer":"samsung","name":"SM-G960F","playServicesInstallDate":"20081231150000Z","playServicesVersion":"21.09.15","ramInMb":3786,"totalSpaceInMb":55675,"version":"1585545517000"},"network":{"bearer":"WIFI","operator":"27205","simCode":"27211","ssid":"<unknown ssid>"},"os":{"name":"android","versionCode":29,"versionName":"10"},"ts":"20210331170329Z"}
<<< HTTP 200, 792.00B
The pushToken is the Firebase authentication token associated with app com.altice.android.myapps

POST <https://sun-apps.sfr.com/reportusage/v1>
Headers

Authorization: Basic **MS4wLjA6NzY3OTAyYjNIYjgxOGNjMA==**
{"application":{"id":"com.altice.android.myapps","pushConnector":"firebase","pushSettings":{"pushToken":"egHfAlJvXwI:AP...u2_9Y8rr-","

```
versionCode":1603000,"versionName":"1.6.0"},"device":{"freeSpaceInMb":53147,"id":"25808231f4418216","lastReboot":"20210330110834Z","manufacturer":"samsung","name":"SM-G960F","playServicesInstallDate":"20081231150000Z","playServicesVersion":"21.09.15","ramInMb":3786,"totalSpaceInMb":55675,"version":"1585545517000"},"network":{"bearer":"WIFI","operator":"27205","simCode":"27211","ssid":"<unknown ssid>"},"os":{"name":"android","versionCode":29,"versionName":"10"},"sessions":[{"application":{"id":"com.altice.android.myapps","versionCode":1603000,"versionName":"1.6.0"},"duration":2,"tags":[{"ts":"20210331170330Z","type":"core","key":"system_notif_enabled","value":"true"},{"ts":"20210331170330Z","type":"core","key":"wakeup_first_success","value":"OOBEWakeUpAlarm"},{"ts":"20210331170331Z","type":"core","key":"wakeup_success","value":"standard"}],"trigger":"OTHER","ts":"20210331170330Z","type":"background"}],"ts":"20210331170331Z"}<<< HTTP 200, 110.00B
```

This appears to send telemetry data from app com.altice.android.myapps. It is followed by several similar connections

POST <https://app-measurement.com/a>

Calls to Google Analytics are made by: com.wssyncml dm, com.samsung.android.samsungpass, com.samsung.android.authfw, com.altice.android.myapps, com.samsung.android.bixby.agent, com.samsung.android.game.gamehome, com.google.android.googlequicksearchbox, com.sec.android.app.samsungapps, com.sfr.android.sfrjeux
The POST body is a protobuf. We decoded it by reconstructing the protobuf definition from the decompiled Firebase code. An example of a decoded Google/Firebase Analytics protobuf associated with com.sec.android.app.samsungapps is:
POST <https://app-measurement.com/a>

```
body {
  always_one: 1
  event {
    event_info {
      setting_code: "_o" // firebase_event_origin
      data_str: "auto"
    }
    event_info {
      setting_code: "_sc" // firebase_screen_class
      data_str: "SamsungAppsMainActivity"
    }
    event_info {
      setting_code: "_si" // firebase_screen_id
      data_int: 1593673781183374425
    }
    event_code: "_vs" // screen_view
    event_timestamp: 1617263297792
  }
  event {
    event_info {
      setting_code: "_o" // firebase_event_origin
      data_str: "auto"
    }
    event_info {
      setting_code: "_pc" // firebase_previous_class
      data_str: "SamsungAppsMainActivity"
    }
    event_info {
      setting_code: "_pi" // firebase_previous_id
      data_int: 1593673781183374425
    }
    event_info {
      setting_code: "_sc" // firebase_screen_class
      data_str: "DisclaimerActivity"
    }
    event_info {
      setting_code: "_si" // firebase_screen_id
      data_int: 1593673781183374426
    }
    event_code: "_vs" // screen_view
    event_timestamp: 1617263298343
    previous_event_timestamp: 1617263297792
  }
  event {
    event_info {
      setting_code: "_o" // firebase_event_origin
      data_str: "auto"
```

```

    }
    event_info {
      setting_code: "_sc" // firebase_screen_class
      data_str: "DisclaimerActivity"
    }
    event_info {
      setting_code: "_si" // firebase_screen_id
      data_int: 1593673781183374426
    }
    event_code: "_s" // session_start
    event_timestamp: 1617263307859
  }
  event {
    event_info {
      setting_code: "_o" // firebase_event_origin
      data_str: "auto"
    }
    event_info {
      setting_code: "_et" // engagement_time_msec
      data_int: 13889
    }
    event_info {
      setting_code: "_sc" // firebase_screen_class
      data_str: "DisclaimerActivity"
    }
    event_info {
      setting_code: "_si" // firebase_screen_id
      data_int: 1593673781183374426
    }
    event_info {
      setting_code: "_fr"
      data_int: 1
    }
    event_code: "_e" // user_engagement
    event_timestamp: 1617263312233
    previous_event_timestamp: 1617102970810
  }
  event {
    event_info {
      setting_code: "_o" // firebase_event_origin
      data_str: "auto"
    }
    event_info {
      setting_code: "_pc" // firebase_previous_class
      data_str: "DisclaimerActivity"
    }
    event_info {
      setting_code: "_pi" // firebase_previous_id
      data_int: 1593673781183374426
    }
    event_info {
      setting_code: "_sc" // firebase_screen_class
      data_str: "SamsungAppsMainActivity"
    }
    event_info {
      setting_code: "_si" // firebase_screen_id
      data_int: 1593673781183374425
    }
    event_info {
      setting_code: "_et" // engagement_time_msec
      data_int: 13889
    }
    event_code: "_vs" // screen_view
    event_timestamp: 1617263312262
    previous_event_timestamp: 1617263298343
  }
  event {
    event_info {
      setting_code: "_o" // firebase_event_origin
      data_str: "auto"
    }
    event_info {
      setting_code: "_pc" // firebase_previous_class
      data_str: "SamsungAppsMainActivity"
    }
    event_info {
      setting_code: "_pi" // firebase_previous_id

```

```

      data_int: 1593673781183374425
    }
    event_info {
      setting_code: "_sc" // firebase_screen_class
      data_str: "SamsungAppsMainActivity"
    }
    event_info {
      setting_code: "_si" // firebase_screen_id
      data_int: 1593673781183374427
    }
    event_code: "_vs" // screen_view
    event_timestamp: 1617263324719
    previous_event_timestamp: 1617263312262
  }
  event {
    event_info {
      setting_code: "_o" // firebase_event_origin
      data_str: "auto"
    }
    event_info {
      setting_code: "_pc" // firebase_previous_class
      data_str: "SamsungAppsMainActivity"
    }
    event_info {
      setting_code: "_pi" // firebase_previous_id
      data_int: 1593673781183374427
    }
    event_info {
      setting_code: "_sc" // firebase_screen_class
      data_str: "DisclaimerActivity"
    }
    event_info {
      setting_code: "_si" // firebase_screen_id
      data_int: 1593673781183374428
    }
    event_code: "_vs" // screen_view
    event_timestamp: 1617263325068
    previous_event_timestamp: 1617263324719
  }
  event {
    event_info {
      setting_code: "_o" // firebase_event_origin
      data_str: "auto"
    }
    event_info {
      setting_code: "_et" // engagement_time_msec
      data_int: 7630
    }
    event_info {
      setting_code: "_sc" // firebase_screen_class
      data_str: "DisclaimerActivity"
    }
    event_info {
      setting_code: "_si" // firebase_screen_id
      data_int: 1593673781183374428
    }
    event_info {
      setting_code: "_fr"
      data_int: 1
    }
    event_code: "_e" // user_engagement
    event_timestamp: 161726332697
    previous_event_timestamp: 1617263312233
  }
  event {
    event_info {
      setting_code: "_o" // firebase_event_origin
      data_str: "auto"
    }
    event_info {
      setting_code: "_pc" // firebase_previous_class
      data_str: "DisclaimerActivity"
    }
    event_info {
      setting_code: "_pi" // firebase_previous_id
      data_int: 1593673781183374428
    }

```

```

event_info {
  setting_code: "_sc" // firebase_screen_class
  data_str: "SamsungAppsMainActivity"
}
event_info {
  setting_code: "_si" // firebase_screen_id
  data_int: 1593673781183374427
}
event_info {
  setting_code: "_et" // engagement_time_msec
  data_int: 7630
}
event_code: "_vs" // screen_view
event_timestamp: 1617263332720
previous_event_timestamp: 1617263325068
}
user {
  timestamp: 1617102970810
  setting: "_fot"
  data_int: 1617105600000
}
user {
  timestamp: 1617102970810
  setting: "_fi"
  data_int: 1
}
user {
  timestamp: 1617266211168
  setting: "_lte"
  data_int: 21520
}
message_timestamp: 1617266211155
event_timestamp: 1617263297792
bundle_end_timestamp: 1617263332720
last_bundle_end_timestamp: 1617102970810
operating_system: "android"
operating_system_version: "10"
Build_MODEL: "SM-G960F"
language_country: "en-gb"
timezone_offset_mins: 60
app_store: "manual_install"
package_name: "com.sec.android.app.samsungapps"
app_version: "4.5.10.9"
gmp_version: 13001
gms_version: 210915
google_ad_id: "cfb8a087-1c38-4bb9-bc15-5f51001b8df1"
random_hex: "75ab873c05ee2a0c55c84ae0a12cab46"
dev_cert_hash: 8649123895590768868
daily_conversions_count: 2
gmp_app_id: "1:177401601629:android:51de35712f94c846"
last_bundle_end_timestamp2: 1617102970810
always_true: true
firebase_instance_id: "eeTixjRYmms"
app_version_int: 451009140
config_version: 1586455363470139
M: 23180912
unknown: "G1—"
}

```

It can be seen that the analytics message sends details of the screens/activities viewed by the user plus the duration and a timestamp. In this message the screens/activities are named SamsungAppsMainActivity, DisclaimerActivity – the com.sec.android.app.samsungapps app opens to show a page asking for consent to data collection before continuing, in our test the app was closed without agreeing to data collection and the analytics message appears to be recording this user interaction with the app.

An example analytics message associated with app com.sfr.android.sfrjeux is:

```

body {
  always_one: 1
  event {
    event_info {
      setting_code: "_o" // firebase_event_origin
      data_str: "auto"
    }
  }
  event_info {
    setting_code: "_sc" // firebase_screen_class
    data_str: "SFRJeuxMain"
  }
}

```

```

}
event_info {
  setting_code: "_si" // firebase_screen_id
  data_int: -5200661649680566566
}
event_code: "_vs" // screen_view
event_timestamp: 1617263143778
}
user {
  timestamp: 1617102973930
  setting: "_fot"
  data_int: 1617105600000
}
user {
  timestamp: 1617102973930
  setting: "_fi"
  data_int: 1
}
user {
  timestamp: 1617266211438
  setting: "_lte"
  data_int: 1
}
message_timestamp: 1617266211427
event_timestamp: 1617263143778
bundle_end_timestamp: 1617263143778
last_bundle_end_timestamp: 1617102973930
operating_system: "android"
operating_system_version: "10"
Build_MODEL: "SM-G960F"
language_country: "en-gb"
timezone_offset_mins: 60
app_store: "manual_install"
package_name: "com.sfr.android.sfrjeux"
app_version: "7.1.3"
gmp_version: 15300
gms_version: 210915
google_ad_id: "cfb8a087-1c38-4bb9-bc15-5f51001b8df1"
random_hex: "ddbf9369f3693d89b52e8d00a36e3cf6"
dev_cert_hash: 13200777164556411895
daily_conversions_count: 2
gmp_app_id: "1:637441263799:android:659db5ceb13b5646"
last_bundle_end_timestamp2: 1617102973930
always_true: true
firebase_instance_id: "dwD2or6tu3s"
app_version_int: 7133000
config_version: 1585956163239078
M: 23180912
unknown: "G1—"
}

```

Note that this app was not opened. The screen view being logged here may be associated with browsing of the Settings app, or may not.

Additional connections when logged in to Google:

POST <https://android.googleapis.com/auth>

Headers

```

device: 3ea3cf75b11265ec
app: com.google.android.gms
User-Agent: GoogleAuth/1.4 (starlte QP1A.190711.020); gzip
androidId=3ea3cf75b11265ec&lang=en-GB&google_play_services_version=210915039&sdk_version=29&device_country=ie&it_caveat_types=2&app=com.google.android.gms&check_email=1&oauth2_foreground=0&Email=doug.leith%40gmail.com&token_request_options=CAA4AVAB&service=oauth2%3Ahttps%3A%2F%2Fwww.googleapis.com%2Fauth%2Fcclog&client_sig=38918a453d07199354f8b19af05ec6562ced5788&system_partition=1&callerPkg=com.google.android.gms&Token=aas_et%2FAKppINb...Zbbli-WtAv9h0KLzM%3D&callerSig=38918a453d07199354f8b19af05ec6562ced5788
<<< HTTP 200, 573.00B

```

POST <https://instantmessaging-pa.googleapis.com/google.internal.communications.instantmessaging.v1.LighterCloudMessageService/CheckBootstrapRequired>

Headers

```

x-goog-spatula: CjYKFmNvbS5nb29nbGUu...JfoLDdiwerl
authorization: Bearer ya29.m.CvkBARMXbjgvkMsW...SvrsoiAggB

```

POST body decoded as protobuf:

```
1: 16
2: "doug.leith@gmail.com"
3: "GMM"
```

GET https://www.google.com/complete/search?oe=utf-8&gcc=ie&ctzn=Europe%2FDublin&ctf=1&v=10.85.11.21.arm64&ntyp=1&ram_mb=3610&ar=0&inm=asst&hl=en-GB&nojl=1&client=qsb&android-asbl-pb&qsubts=1617261328207&devloc=0&padt=200&padb=692&cs=0&cds=4&gs_pcr=t&q=&cp=0&psi=wNX_Jd47rug.1617210928445.0&ech=7&dpr=3.0&gs_pcr=1&xssi=t&getexp=1

Headers

```
x-client-instance-id: 30a9375469fc3...e578dc7b51896af
x-client-data: auMDH4sIAAAAAAAAAAA3...7TaU_47asKtpAgAA
cookie: CONSENT=PENDING+027; NID=212=gZkhf2r5Dp8p9LL...
HC22UxhRutyM;SID=8AefxyzWE-uK...MG9E_-RniQk5RGLw.;HSID=
AMPaVqj6ExNP5B8YT;APISID=d4KkRpGmxRtJ2UUA/
A-kiwUg-OsgMWHtZf;_Secure-3PSID=8AefxyzWE-uK4xX2...
dqLCy-RmP-Ttg.;SSID=A7KMSOqZKUVQhYwiT;SAPISID=
Zc5q-YEUMsfibxGF/AMGKNkiPS587PDBTN;_Secure-3PAPISID=
Zc5q-YEUMsfibxGF/AMGKNkiPS587PDBTN
```

Additional cookies are sent to www.google.com/complete/search when logged in

POST https://mail.google.com/mail/ads/main?rt=b&client=27

Headers

```
user-agent: Android-Gmail/61993624 (sw360dp; 480dpi) (starlte QP1A
.190711.020)
authorization: OAuth ya29.a0AfH6SMD81IK0vJm...RqUMb2Mca4
cookie: COMPASS=gmail=CrABAAlr...bbGnMZgyPW4M
```

POST body decoded as protobuf:

<device details, plus data>

This is followed by several similar connections

POST https://inbox.google.com/sync/i/s?hl=en_GB&c=2

Headers

```
x-google-btd: 1
x-gmail-btai: GskBMABQAVgBaAF4...hnmAHKi+7XiC+oAcXkTg==
user-agent: Android-Gmail/61993624 (sw360dp; 480dpi) (starlte QP1A
.190711.020)
authorization: OAuth
```

ya29.a0AfH6SMD81IK0vJmN...iccRhZrQUMb2Mca4

cookie: COMPASS=bigtop-sync=

Co4BAALriVe3PJcd...2VJBzXjCilyC70;

NID=212=N0IE4wRYRXXC-4Piw1Y0IYGxB...IIL9gBNMxKAAdC4jna

POST body decoded as protobuf:

The x-gmail-btai value base64 decodes to device details plus other data. This connection is followed by several similar connections. They appear to be syncing the device with Google Gmail.

POST https://people-pa.googleapis.com/google.internal.people.v2.

InternalPeopleService/ListContactGroups

Headers

```
x-goog-spatula: CjYKfMnVbS5nb29nbGUuYW5...NjfoLDdiwerl
authorization: Bearer
```

ya29.m.CvBARMXbjjMON1Tmmf...P4Mtxx4RayICCAE

<<< HTTP 200, 19.00B

POST https://footprints-pa.googleapis.com/footprints.oneplatform.

FootprintsService/GetActivityControlsSettings

Headers

```
user-agent: com.google.android.gms/210915039 (Linux; U; Android 10;
en_GB; SM-G960F; Build/QP1A.190711.020; Cronet/85.0.4183.127) grpc-
java-cronet/1.37.0-SNAPSHOT
```

authorization: Bearer **ya29.m.Cv4DARMXbjjZvsVh3UO12ve...E-vl-UMGrIlgIAQ**

GET https://www.googleapis.com/calendar/v3/internal/users/me/calendarList?

maxResults=100&supportsAllDayReminders=true

Headers

```
Authorization: OAuth ya29.a0AfH6SMCzo2eC3PQ...or2X7mVw-Dy4
User-Agent: samsung/starltxx/starlte:10/QP1A.190711.020/
G960FXXU8DTC5:user/release-keys:com.google.android.syncadapters.
calendar:2016184095:release Google-HTTP-Java-Client/1.26.0-
SNAPSHOT (gzip)
```

x-goog-api-client: java/0 http-google-bkk/1.26.0 linux/4.9.118

<<< HTTP 200, 2.34KB

The authorization: Bearer acts to link this connection to device and user details. This connection appears to be syncing with Google calendar.

GET https://www.googleapis.com/calendar/v3/internal/calendars/
doug.leith@gmail.com/acl

Headers

Authorization: OAuth **ya29.a0AfH6SMCzo2eC3P...jZor2X7mVw-Dy4**

User-Agent: samsung/starltxx/starlte:10/QP1A.190711.020/

G960FXXU8DTC5:user/release-keys:com.google.android.syncadapters.

calendar:2016184095:release Google-HTTP-Java-Client/1.26.0-

SNAPSHOT (gzip)

x-goog-api-client: java/0 http-google-bkk/1.26.0 linux/4.9.118

<<< HTTP 200, 372.00B

Additional connections when location enabled

POST https://lamssettings-pa.googleapis.com/personalization.settings.

oneplatform.OnePlatformUserSettingsService/SyncSettings

Headers

```
user-agent: com.google.android.gms/210915039 (Linux; U; Android 10;
en_GB; SM-G960F; Build/QP1A.190711.020; Cronet/85.0.4183.127)
```

authorization: Bearer

ya29.m.CvkBARMXbjj0sSZG65c7lRzT...mW7XMKVVLguPIgIIAQ

The authorization: Bearer acts to link this connection to device and user details

POST https://mobilenetworkscoring-pa.googleapis.com/v1/GetWifiQuality?
key=AIzaSyBr...o4ZidOeA6ThtVczU

Headers

X-Goog-Spatula: CjYKfMnVbS5nb29nbGUuYW5...foLDdiwerl

User-Agent: GmsCore/210915039 (starlte QP1A.190711.020); gzip

```
2 {
```

```
1: w0a74hNSpMdf43ZIJKqlafPMOxF2GKE6VLjoRYPK8pM
```

```
}
2 {
```

```
1: 46DBeKN7TTiPXKePbcGbogPyr4kjVAYsq68wLU6GW3Y
```

```
}
3: 27211
```

```
4: ..
```

<<< HTTP 200, 181.00B

Set-Cookie: NID=212=raJYGP3IHK...zYwJJxGVq8

The cookie acts to link this connection to device and user details. The 27211 value in the payload is the cellular operator MNC/MCC identifier. Based on discussions with Google the other values are hashes of the Wifi MAC addresses of nearby access points, used to query a network quality database to determine the best Wifi network to connect to.

Connections When Insert Sim

POST https://api.omc.samsungdm.com/v5/api/device/simChange

Headers

```
Authorization: auth_identifier="OMC-AUTH",server_id="q95ikwe7b6",
device_id="227ae90a14017ece",signature="1DFU8P/4
```

XmokevfvIJeOE6NNI9Hae55QR1XMOHwMHBk="

User-Agent: samsung SM-G960F OMC-AGENT

```
{"carrierId":"SFR","clientVersion":"5.2.25","deviceId":"
```

```
227ae90a14017ece","deviceUN":"CE0218227AE90A14017E","emStatus
```

```
":"0","fingerPrint":"samsung/starltxx/starlte:10/QP1A.190711.020/
```

```
G960FXXU8DTC5:user/release-keys","fwVersion":"G960FXXU8DTC5/
```

```
G960FOXJ8DTC5/G960FXXU8DTC5","modelName":"SM-G960F","
```

```
multiCscCode":"OXJ","omcEnable":"off","omcVersion":"SAOMC_SM-
```

```
G960F_OXJ_SFR_QQ_0007","osVersion":"10","salesCode":"SFR","simList
```

```
":["canonicalId":"10130","gid1Code":"0
```

```
FFFFFFFFFFFFFFFFFFFFFFFFFFFFFFFF","mccCode":"272","
```

```
mncCode":"11","networkCode":"SFR","networkNameID":"","spnCode":"
```

Tesco

```
Mobile","subsetCode":"01038"}],tMccCode":"","tMncCode":""}
```

<<< HTTP 200, 19.00B

Set-Cookie: WMONID=NNpYIXVUfSQ

Plus usual Google connections when a Sim is inserted

C. Connections When Interacting With Settings App

POST http://ie-odc.samsungapps.com/ods.as
 <?xml version="1.0" encoding="UTF-8" standalone="yes"?><
 SamsungProtocol networkType="0" deviceModel="SM-G960F_TM" mcc
 ="272" mnc="11" csc="SFR" odcVersion="5.1.02.305" odcType="02"
 OTFVersion="9000000" openApiVersion="29" lang="EN" version="6.3"
 version2="3" filter="1" sessionId="31b62e084202103311733"><request
 id="775060" name="terminformationForTheme" numParam="2"
 transactionId="31b62e084000"><param name="fileFlag">0</param><
 param name="flag">4</param></request></SamsungProtocol>
 <<<< HTTP 200, 1.75KB
Set-Cookie: JSESSIONID=DIWUuCiSIKT7s7...KZLZ5.s02w03odc03; path
 =/, SCOUTER=x7hkg7h1pgch0j

GET https://app-measurement.com/config/app/1%3A327451421140%3
 Aandroid%3A9c547b5ed466b580?app_instance_id=
ce46c2ad3c233fd7076aedb0d4e67ab8&platform=android&gmp_version
 =210915

POST https://app-measurement.com/a
 Google Analytics is called by com.samsung.android.themestore and
 com.samsung.android.samsungpass

POST https://firebaseremoteconfig.googleapis.com/v1/projects
 /441321514896/namespaces/fireperf:fetch?key=AlzaSyAb...zc2vVGP4
 Headers
 User-Agent: Google-API-Java-Client Google-HTTP-Java-Client
 /1.26.0-SNAPSHOT (gzip)
 x-goog-api-client: java/0 http-google-ca/1.26.0 linux/4.9.118
 x-android-package: com.samsung.android.samsungpass
 {"analyticsUserProperties":{"appId":"1:441321514896:android:
 bb3362342ca50fca","appInstanceId":"f2UsrZMi7X0","appVersion
 ":"2.0.02.14","countryCode":"GB","languageCode":"en_GB","packageName
 ":"com.samsung.android.samsungpass","platformVersion":"29","sdkVersion
 ":"17.0.0","timeZone":"Europe/Dublin"}
 <<<< HTTP 200, 1.14KB

POST https://android.clients.google.com/c2dm/register3
 Headers
 Authorization: AidLogin **4513679355944265196**:3426139596944398072
 app: com.samsung.android.samsungpass
 gcm_ver: 210915039
 User-Agent: Android-GCM/1.5 (starlte QP1A.190711.020)
 X-subtype=441321514896&sender=441321514896&X-app_ver
 =200214900&X-osv=29&X-cliv=fiid-12451000&X-gmsv=210915039&X-
 appid=f2UsrZMi7X0&X-scope=*&X-gmp_app_id=1%3A441321514896
 %3AAndroid%3Abb3362342ca50fca&X-Firebase-Client=fire-abt%2F17
 .1.1+fire-iiid%2F18.0.0+fire-core%2F19.0.0+fire-rc%2F17.0.0+fire-android
 %2F+fire-perf%2F18.0.1+fire-analytics%2F17.2.0&X-app_ver_name
 =2.0.02.14&app=com.samsung.android.samsungpass&device=
4513679355944265196&app_ver=200214900&info=w-
 QYjhNq2p0UQKri541rkWUINVAuiBc&gcm_ver=210915039&plat=0&cert
 =9ca5170f3819...143b3163&target_ver=29
 <<<< HTTP 200, 158.00B

GET https://auth2.samsungosp.com/v2/license/open/whoareyou
 <<<< HTTP 200, 127.00B

GET https://us-api.mcsvc.samsung.com/contents/v1.0/cmn/banners?
 pageDomainName=tips.phone.q

Headers
 x-smcs-cc2: GC
 x-smcs-prod: 0vMpROaaRW2QxKuds1xUcA
 x-smcs-pt: 01
 x-smcs-lang: en
 x-smcs-did: **AH9gFoLwOxrhmasRLWIJeBmJ1Pfx-**
GMcF7wDEq5uebFLk=
 x-smcs-model-id: SM-G960F
 x-smcs-mnfctr: samsung
 x-smcs-os: 29
 x-smcs-sales-cd: OXA
 x-smcs-android-id: 0000
 x-smcs-ad-id: 0000
 x-smcs-mcc: 272
 x-smcs-mnc: 5
 x-smcs-ver: 2.0.33.0
 <<<< HTTP 200, 165.07KB
Set-Cookie: SCOUTER=z5blthkqot019p; Expires=Mon, 18-Apr-2089

19:53:46 GMT; Path=/

GET https://us-cdn-gpp.mcsvc.samsung.com/gpp_us
 /1247322517039484930/en/inventoryImage_26fcdad6lq.png?marker
 =1568590082534%5EinventoryImage_26fcdad6lq.png
 Plus other similar connections

GET https://us-rd.mcsvc.samsung.com/statistics/impression?ep=4
 B760ABCA39FF8C5857844F7A3EAAEA443C795239368F2B81D8F6C3447DE7E9B73312
 ;ii=1246838895491878922;cc=GC;od=1;si=21;pg=83410538838167555;md=
 SM-G960F;pi=0vMpROaaRW2QxKuds1xUcA;li=1247321967272988684;cp
 =1247321891022311425;svr=bnr;rtp=st;ap=2.0.33.0;ri=
 hv381O79Qbq4eBVXej1p&fc=1&sid=**guSx3aiGRXmyCI3vBlu5Jg**
 <<<< HTTP 200, 0.00B
 Plus other similar connections

D. Connections When Make/Receive Phone Call

POST https://samsung-directory.edge.hiyaapi.com/v3/track_events
 X-Hiya-Request-Id: e822210e-dafc-4db2-ba8f-f53b2f14d898
 X-Hiya-Device-Info: samsung/SM-G960F
 X-Hiya-Os-Info: Android29/G960FXXU8DTC5
 X-Hiya-Device-Locale: en-GB
 x-seq_id: 48187500-1e3b-4b19-8868-f8ad4675aca5
 X-Hiya-Date: 1617263385326
 X-Hiya-Product-Name: Samsung
 X-Hiya-Installation-User-ID: **74da709a-76ef-45d7-91e8-6b9e055a3720**
 X-Hiya-Product-Version-Code: 20509
 X-Hiya-User-TZ-Offset-Seconds: 3600
 X-Hiya-Device-User-ID: **b527702066a24291**
 X-Hiya-Samsung-Sales-Code: SFR
 X-Hiya-Country-Code: IE
 X-Hiya-Product-AAR-Version: 2.5.6-145
 X-Hiya-Product-Version: 2.5.9-samsung-414
 X-Hiya-Advertising-ID: **cfb8a087-1c38-4bb9-bc15-5f51001b8df1**
 Authorization: Bearer eyJraWQioiJDMEiON...383R24f2w
 {
 "trackEvents": [
 {
 "eventCreatedNanoId": 152643634632320,
 "eventCreatedTime": "2021-04-01T07:49:45.322Z",
 "eventId": "e9b4786f-ddb4-4d44-a2f4-7bac871a6257",
 "eventSentNanoId": 152643636596397,
 "eventType": "Action",
 "properties": {
 "trigger": {
 "stringValue": "PlacesTab"
 },
 "screen": "Phone"
 },
 },
 ],
 }
 }
 <<< 200 OK 0b

The Authorization bearer header value is a JWT token that decodes to:

```
{
  "iss": "auth.edge.hiyaapi.com",
  "aud": [
    "smartcall.edge.hiyaapi.com",
    "places.edge.hiyaapi.com",
    "samsung-phones.edge.hiyaapi.com",
    "samsung-ingestion.edge.hiyaapi.com",
    "samsung-directory.edge.hiyaapi.com",
    "samsung-callerprofile.edge.hiyaapi.com"
  ],
  "exp": 1617870564,
  "iat": 1617262634,
  "hgt": "hiya_grant",
  "hpn": "Samsung",
  "hin": "74da709a-76ef-45d7-91e8-6b9e055a3720",
  "has": [],
  "hui": "7c2599e2-b655-40da-bfe0-fc5924509bdb"
}
```

A similar connection with "stringValue": "SC_ShowNotification" is also sent.

II. XIAOMI

Summary:

<i>Xiaomi system app endpoints:</i><br>tracking.intl.miui.com	<i>Identifiers Sent:</i><br>IMEIs, IMSI, VAID, GAID, fid, hash of Wifi MAC address, instance_id
api.sec.intl.miui.com	hash of deviceID, hash of android_id in secure settings, hash of devID
api.ad.intl.xiaomi.com	GAID
update.intl.miui.com	hash of IMEI
fr.register.xmpush.global.xiaomi.com	GAID, VAID
find.api.micloud.xiaomi.net	fid, devID
data.mistat.xiaomi.com	sid, AES key
sdkconfig.ad.intl.xiaomi.com	i
mcc.intl.inf.miui.com	uid
global.market.xiaomi.com	guid, Firebase IDs
<i>Third-party (non-Google) system app endpoints:</i><br>moaps.tmo.net	<i>Identifiers Sent:</i><br>uid
www.facebook.com, graph.facebook.com	ASHAS, handset IP address
Identifiers observed to persist across factory reset	IMEI, IMSI, fid, devID, deviceID, Wifi MAC address (including hashes of these values), ASHAS, handset IP address

TABLE III

SUMMARY OF IDENTIFIERS SENT IN SYSTEM APP CONNECTIONS (EXCLUDING GOOGLE SYSTEM APPS).

<i>Telemetry</i>	
tracking.intl.miui.com	Logs app windows/activities displayed to user, with timestamp and duration. Logs, for example, time/duration of phone calls.
System apps com.miui.msa.global, com.xiaomi.discover, com.android.thememanager	logs events using Google Analytics
moaps.tmo.net	Logs events enableractivated
www.facebook.com, graph.facebook.com	device sensor data (accelerometer, rotation, battery)
<i>Device Data</i>	
tracking.intl.miui.com	device details, installed apps
global.market.xiaomi.com	device details, installed apps
api.sec.intl.miui.com	device details
api.ad.intl.xiaomi.com	device details
fr.register.xmpush.global.xiaomi.com	device details
sdkconfig.ad.intl.xiaomi.com, mcc.intl.inf.miui.com	device details
moaps.tmo.net	device details
www.facebook.com, graph.facebook.com	device details
<i>Notes:</i>	
The messaging app on the handset is com.google.android.apps.messaging. This Google app uses Google Analytics to log user interaction, including screens/activities viewed plus duration and timestamp, and logs the event that text is sent.	

TABLE IV

SUMMARY OF DATA SENT IN SYSTEM APP CONNECTIONS (EXCLUDING GOOGLE SYSTEM APPS).

- 1) The handset sends the following device identifiers to tracking.intl.miui.com: (i) the device IMEIs, (ii) the Security DeviceID (from service miui.sedc, SecurityDeviceCredentialManager), (iii) an MD5 hash of the device Wifi MAC address, (iv) the Google adid/rdid/gaid advertising id, (v) the device VAID, (vi) the device CPUID. The IMEIs, Security DeviceID and Wifi MAC address are all

long-lived device identifiers that persist across a factory reset. The Google advertising id changes on a factor reset but can be relinked to the device by Google (long-lived device identifiers such as the hardware serial number, IMEI and Wifi MAC address are recorded by Google sent in connections along with the Google advertising id). The data shared in connections to tracking.intl.miui.com therefore allows persistent, long-lived linking of the device data collected by Xiaomi and Google. The VAID value changes upon a factory reset, and in that sense seems akin to the Google Android ID.

- 2) The Google advertising id is sent in connections to: r.register.xmpush.global.xiaomi.com, api.ad.intl.xiaomi.com, privacy.api.intl.miui.com and, as already mentioned, tracking.intl.miui.com.
- 3) Long-lived device identifiers that are hashes of the IMEI and Security DeviceID are sent to: update.intl.miui.com, api.sec.intl.miui.com. The Security DeviceID is sent to find.api.micloud.xiaomi.net.
- 4) Detailed telemetry of user interaction with the device is sent to tracking.intl.miui.com. Note that this occurs even though the ‘User Experience Programme’, ‘Send diagnostic data automatically’ and ‘Personalized ad recommendations’ options in the device Xiaomi settings are set off (Google ‘Usage and Diagnostics’ is set off too). This telemetry reveals, for example, the start and end time of phone calls. It also reveals user interactions with the device privacy and permissions settings, amongst others.
- 5) The Xiaomi com.miui.msa.global, com.xiaomi.discover and com.mi.android.globalFileexplorer system apps log handset activity using Google Analytics and Crashlytics.
- 6) Details of installed software are sent to tracking.intl.miui.com and global.market.xiaomi.com.
- 7) When a SIM is inserted into the handset the SIM IMSI (which uniquely identifies the SIM) is sent in connections to tracking.intl.miui.com. In addition, SIM details are sent to Google as observed in previous studies, see ‘Mobile Handset Privacy: Measuring The Data iOS and Android Send to Apple And Google’, Proc Securecomm 2021.
- 8) When making/receiving a phone call details of user interaction with the dialer app are sent to tracking.intl.miui.com. In addition, when making an outgoing call the phone number dialed is sent to Google at dialercallinfolookuppa.googleapis.com, and similarly when receiving a call the incoming number is also sent to dialercallinfolookuppa.googleapis.com.
- 9) When sending a text, the messaging app com.google.android.apps.messaging uses Google Analytics to log user interaction, including screens/activities viewed plus duration and timestamp.
- 10) When browsing the Settings app connections are made to privacy.mi.com, privacypolicy.truste.com, cdn.cnbj1.fds.api.miimg.com, graph.facebook.com, global.market.

xiaomi.com, pagead2.google syndication.com. Details of user interactions with the Settings app are sent to /tracking.intl.miui.com.

Pre-installed Non-Xiaomi System Apps

- 1) *Mobile Operator*. The particular handset used in these measurements was bought secondhand online and appears to originally be from German mobile operator Deutsche Telekom. The pre-installed system apps include de.telekom.tsc. This app sends device details and telemetry to moaps.tmo.net (this domain appears to be owned by Deutsche Telekom).
  - 2) *Facebook*. Handset data is sent to www.facebook.com and graph.facebook.com. Data sent includes device details, device accelerometer and rotation measurement data, the handset IP address and the mobile carrier name. The ASHAS value sent along with this data appears to act as a long-lived device identifier (it persists across factory resets).
  - 3) *Google*. The following pre-installed Google system apps were observed to send data to Google.
    - a) Google Play Services and Google Play store make many connections to Google servers. These share persistent device and user identifiers with Google including the device hardware serial number, SIM IMEI, Wifi MAC address, SIM IMSI and phone number, user email (when logged in). A substantial quantity of data is sent, in particular, to play.googleapis.com/vn/log/batch, play.googleapis.com/play/log and www.googleapis.com/experimentsandconfigs. This is consistent with other recent measurement studies, see “Mobile Handset Privacy: Measuring The Data iOS and Android Send to Apple And Google”, Proc SECURE-COM 2021.
    - b) Google Youtube sends device data, including persistent identifiers and the Google adid/rdid advertising identifier and the AndroidID, to www.googleadservices.com and youtubei.googleapis.com. Youtube also uses Google Analytics to log events, and presumably also user interaction.
    - c) Connections are periodically made to www.google.com/complete/search. These are associated with the com.google.android.googlequicksearchbox searchbar app embedded in the handset UI and send a cookie which acts to link these connections to persistent device and user identifiers. Less frequent connections are made to www.google.com/m/voice-search/down that contain what appears to be a persistent device identifier. The com.google.android.googlequicksearchbox app also sends telemetry data to Google Analytics.
    - d) The system messaging app com.google.android.apps.messaging uses Google Analytics to log user interaction, including screens/activities viewed plus duration and timestamp. For example, when sending a text the user interactions with the WelcomeActivity, HomeActivity and ConversationActivity are logged, and a FIRST_MESSAGE_SENT event is also recorded.
    - e) When logged in to a Google account, connections are made to mail.google.com/mail/ads, inbox.google.com/sync and www.googleapis.com/calendar that send identifiers linked to the device and user account. Note that account login was carried out via the Google Play app only. Syncing of gmail, contacts, calendar took place without the user being asked or opting in.
    - f) When logged in to a Google account, connections are also made to instantmessaging-pa.googleapis.com, people-pa.googleapis.com, footprints-pa.googleapis.com. It's not clear what the purpose of these connections is or what data is sent.
    - g) When location is enabled additional connections are made to lamssettings-pa.googleapis.com.
    - h) The com.google.android.dialer app makes connections to businesscalls.googleapis.com. As already noted, when making an outgoing call the phone number dialed is sent to dialercallinfolookuppa.googleapis.com, and similarly when receiving a call the incoming number is also sent to dialercallinfolookuppa.googleapis.com.
    - i) Google Chrome makes connections to Google servers. These connections are consistent with previously documented behaviour, see “Web Browser Privacy: What Do Browsers Say When They Phone Home?”, IEEE Access. DOI 10.1109/ACCESS.2021.3065243.
- Note that none of these apps, apart from the dialer app, were opened on the device. No popup or request to send data was observed.
- 4) *Google Analytics*. Several pre-installed system apps log handset activity using Google Analytics and Crashlytics. These include: com.miui.msa.global, com.xiaomi.discover, com.mi.android.globalFileexplorer, com.google.android.apps.walletnfcrel, com.google.android.dialer, com.google.android.apps.maps, com.google.android.gm, com.google.android.calendar, com.google.android.youtube, com.google.android.apps.messaging, com.google.android.gms, com.android.vending.

A. Selected Connections During Startup After Factory Reset

POST http://globalapi.ad.xiaomi.com/filter/text?clientInfo=alpha&appKey=MiAdSdk_i18n&version=-1

POST <https://firebaseinstallations.googleapis.com/v1/projects/originfileexplorer/installations>

Headers

X-Android-Package: com.mi.android.globalFileexplorer
 {"fid":"eBuB7fuPSvG1JmpwGFct1E","appId":"1:599532519894:android:c497eda781bcf4","authVersion":"FIS_v2","sdkVersion":"a:16.3.5"}
 <<< HTTP 200, 576.00B

App com.mi.android.globalFileexplorer connecting with Google Firebase. Similar connections are made by com.google.android.apps.walletnfcrel, com.xiaomi.discover, com.google.android.dialer, com.google.android.apps.maps, com.google.android.gm,

com.google.android.calendar, com.google.android.youtube. Later connections to android.clients.google.com/c2dm/register3 link the app Firebase ID with the device Google AndroidID.

POST https://fr.register.xmpush.global.xiaomi.com/pass/v2/register
 aaid=b8e43b13-a2df-4d40-8960-4e6b6ab499f3&appid=1000271&apptoken=420100086271&appversion=30006011&board=merlinnfc&brand=Redmi&devid=a-352F8AC09AEB8A4490A4288F17B564DE3C76EC70&gaid=d90967c6-d79d-4345-8de5-07357871b0ee&model=M2003J15SC&oad_type=1&os=10-V12.0.7.0.QJOMIXM&packagename=com.xiaomi.xmsf&ram=4.0GB&rom=128.0GB&sdkversion=30800&space_id=0&vaid=465ddbcb3dc3dce

<<<< HTTP 200, 394.00B

This connection seems to be registering the device with a Xiaomi push service. The gaid value is the Google adid/rdid advertising identifier. The deviceid, aaid (Application Anonymous Device Identifier?) and vaid (Vender Anonymous Device Identifier?) values are generated by calls to com.android.id.impl.IdProviderImpl and act as device identifiers.

POST https://data.mistat.intl.xiaomi.com/get_all_config
 ai=1005545&av=7.4.3&m=M2003J15SC&rc=S&rg=IE&sv=3.0.17&t=2
 <<<< HTTP 200, 321.00B

This is followed by a sequence of similar connections.

GET https://resolver.msg.global.xiaomi.net/gslb/?ver=4.0&type=wifi&uaid=0&list=fr.app.chat.global.xiaomi.net%2Cresolver.msg.global.xiaomi.net&countrycode=IE&sdkver=41&osver=29&os=M2003J15SC%3AV12.0.7.0.QJOMIXM&mi=3&key=59a16fe82d...5c4d5b9
 <<<< HTTP 200, 2.80KB

GET https://api.g.micloud.xiaomi.net/micAnonymous/mic/config
 Headers

Cookie: sdkVersion=Android-0.0.0-Alpha
 <<<< HTTP 200, 414.00B

POST https://data.mistat.xiaomi.com/get_all_config
 ai=2882303761517144351&av=10&m=M2003J15SC&rc=S&rg=IE&sv=3.0.16&t=2
 <<<< HTTP 200, 288.00B

POST https://update.intl.miui.com/updates/carrierChannel.php
 q=6dvS64SIVHGgVsy...16Wvsij6&s=9827EF..63D3DF&t=1626857420&sid=1
 <<<< HTTP 200, 32.00B
 The q value is AES/CBC encrypted (using a hardwired key and iv) and then base64 encoded, it decodes to:
 {"d":"merlin_global","i":"8e65202eb33628ca34d0ffb9f8882ff83044b4c96bf2b43a854d58056603e51c"}

The i value is an SHA256 hash of the device IMEI.

POST https://api.ad.intl.xiaomi.com/brand/pushConfig
 clientInfo={"deviceInfo":{"screenWidth":1080,"screenHeight":2340,"screenDensity":2,"model":"M2003J15SC","device":"merlinnfc","androidVersion":"10","miuiVersion":"V12.0.7.0.QJOMIXM","miuiVersionName":"V12","bc":"S","make":"xiaomi","isInter":true,"os":"android","modDevice":"merlin_global","customizedRegion":"","restrictImei":false},"userInfo":{"locale":"en_US","language":"en","country":"IE","customization":"","networkType":-1,"connectionType":"WIFI","ua":"Dalvik/2.1.0+(Linux;U;+Android+10;+M2003J15SC+MIUI/V12.0.7.0.QJOMIXM)","serviceProvider":"","triggerId":"3609af241...dff41"},"gaid":"d90967c6-d79d-4345-8de5-07357871b0ee"},"isPersonalizedAdEnabled":false},"applicationInfo":{"platform":"xiaomi","packageName":"com.miui.msa.global","version":2020072000}}&v=1.0&isbase64=false&appKey=GLOBAL_NOTIFICATION&sign=d8b35927...800b220e4ad
 <<<< HTTP 200, 1.16KB

The gaid value is the Google adid/rdid advertising identifier.

POST https://api.ad.intl.xiaomi.com/brand/splashConfig
 'clientInfo={"applicationInfo":{"packageName":"com.miui.msa.global","platform":"xiaomi","version":2020072000},"deviceInfo":{"androidVersion":"10","bc":"S","customizedRegion":"","device":"merlinnfc","firstBootTime":0,"isInter":true,"make":"xiaomi","miuiVersion":"V12.0.7.0.QJOMIXM","miuiVersionName":"V12","modDevice":"merlin_global","model":"M2003J15SC","os":"android","screenDensity":2,"screenHeight":2340,"screenWidth":1080},"userInfo":{"connectionType":"WIFI","country":"IE","customization":"","gaid":"d90967c6-d79d-4345-8de5-07357871b0ee"}

isPersonalizedAdEnabled":false,"language":"en","locale":"en_GB","networkType":-1,"serviceProvider":"","triggerId":"0678...0a680","ua":"Dalvik/2.1.0 (Linux; U; Android 10; M2003J15SC MIUI/V12.0.7.0.QJOMIXM)"},"&nonce=32ec52e2da...fa30bd09&sv=0&packageName=com.miui.msa.global&isbase64=false&appKey=system_splash&sign=5b737...82fdb'
 <<<< HTTP 200, 3.35KB

POST https://update.intl.miui.com/api/v1/carrier
 n=F8A7ED5B...A9467679&q=BRvAhe4...3D&s=1&t=&ts=1626857421117&sid=1
 <<<< HTTP 200, 0.00B
 The q value is AES/CBC encrypted then base64 encoded, it decodes to:
 {"mnc":"","-1","d":"merlin_global","imei":"8e65202eb33628ca34d0ffb9f8882ff83044b4c96bf2b43a854d58056603e51c","mcc":"","-1","gid1":null,"sim_spn":"","cid":"","-1"}
 The i value is an SHA256 hash of the device IMEI.

POST https://update.intl.miui.com/updates/miotaV3.php
 Headers

Cookie: serviceToken=;
 Cache-Control: no-cache
 q=zbMQ2f3hwq...D&s=1&t=1626857420991&sid=1
 <<<< HTTP 200, 5.94KB

The q value decodes to:
 {"a":"0","b":"F","c":"10","unlock":"","d":"merlin_global","f":"2","g":"e1971844738ce834b968581eb62d7d2e","channel":"","isR":"","l":"en_US","sys":"","n":"","r":"IE","bv":"12","v":"MIUI-V12.0.7.0.QJOMIXM","id":"","sn":"0xb288b354cf1b8e61182ee71fb2541f30102dcd96","sdk":"","29","pn":"merlinnfc_global","options":{"zone":"2","ab":"","0","previewPlan":"","0"}}
 The g value is an md5 as of the device android_id. The sn value is device property ro.boot.cpubid.

POST https://update.intl.miui.com/api/v1/apex
 n=4A73F8BBB...960EACBB4F0&q=ZnUrGbgLlgCXt...3D&s=1&t=&ts=1626857420991&sid=1
 <<<< HTTP 200, 320.00B
 The q value decodes to:
 {"b":"F","r":"IE","c":"10.0","d":"merlin_global","bv":"V12.0.7.0.QJOMIXM","f":"2","g":"e1971844738ce834b968581eb62d7d2e","id":"","l":"","en_US","o":"UNKNOWN"}

GET https://api.ad.intl.xiaomi.com/track/pi/v1.0?nonce=cc64f454...2a9525&ts=1626857421106&value=eyJhZHYiOi...lmxpc3QifQ==&sign=e357798d0b5...ee97b093a0
 <<<< HTTP 200, 75.00B
 The "value" parameter base64 decodes to:
 {"adv":"","10","at":1626857090098,"cfgId":"","null","cv":2020072000,"device":"merlinnfc","ile":"","system","make":"xiaomi","mi":true,"miui":"V12.0.7.0.QJOMIXM","model":"M2003J15SC","n":"","-1","nonce":"","cc64f45468981a01f1e1e325ae2a9525","region":"IE","st":1626857421106,"pilst":["com.facebook.katana","com.miui.android.fashiongallery","com.duokan.phone.remotecontroller","com.agoda.mobile.consumer","cn.wps.xiaomi.abroad.lite","com.micredit.in","com.tencent.igxiaomi","com.netflix.mediaclient"],"event":"list"}

GET https://moaps.tmo.net/live/v2/xx?os-version=29&app-version=90200000&device-variant=OM&locale=en_US&manufacturer=Xiaomi&model=Xiaomi-M2003J15SC-om&spn=empty&mcc=null&mnc=null&gid1=null&device-size=6.6&cuid=cdc229b6-6ffc-471d-97de-9fd58f02e2e1
 Headers

User-Agent: AppTokens/Android/xx/Xiaomi/M2003J15SC/10/9.2.0/-/-/-/-/-/-
 <<<< HTTP 200, 7.25KB

This connection is associated with app de.telekom.tsc and the domain moaps.tmo.net belongs to Deutsche Telekom AG. The cuid value is a device identifier (randomly generated when app de.telekom.tsc is first started) that is included in later connections.

POST https://moaps.tmo.net/events
 Headers

Authorization: u1fEuOeiz...xaVDfcUbaP5q
 {"events":[{"_uid":"cdc229b6-6ffc-471d-97de-9fd58f02e2e1","_event":"sim-read","action":"no-sim-detected","deviceLanguageCode":"English","manufacturer":"Xiaomi","device-model":"M2003J15SC","device-type":"OM","brand_id":"xx","client":"de.telekom.tsc","client-version":"9.2.0","os-version":"10","created-time":"2021-07-21T16:42:29.805+0800","fw-build-

number":"QP1A.190711.020"},{"_uid":"cdc229b6-6ffc-471d-97de-9fd58f02e2e1","_event":"enabler-activated","versionCode":"9020000","manufacturer":"Xiaomi","device-model":"M2003J15SC","device-type":"OM","brand_id":"xx","client":"de.telekom.tsc","client-version":"9.2.0","os-version":"10","created-time":"2021-07-21T16:42:29.787+0800","fw-build-number":"QP1A.190711.020"]}]

<<< HTTP 200, 15.00B

The `_uid` value matches the `cuid` device identifier in the first connection to `moaps.tmo.net` and acts as a device identifier.

POST https://api.sec.intl.miui.com/common/whiteList/listByModule
appVersion=29&carrier=unknown&dataVersion=0&device=merlinnfc&imei=EEEBAD9FD7521590AD9F715BD07CE7&initdev=false&isDiff=true&miuiVersion=V12.0.7.0.QJOMIXM&module=RestrictAppControl&param=dXBkYXR1&region=IE&t=stable&sign=51E29E8505...1AE074E
<<< HTTP 200, 20.45KB

POST https://www.googleapis.com/androidantiabuse/v1/x/create?alt=PROTO&key=AlzaSyB...zOIZ-ITI
Sends device details to Google, including the hardware serial number. Followed by a sequence of related Google DroidGuard connections.

GET https://api.ad.intl.xiaomi.com/track/pi/v1.0?nonce=90f504c5...7ea73&ts=1626857423387&value=eyJhZHYiOi...16ImFjdG12ZS9&sign=7e9db6a783...8a551254

The "value" parameter base64 decodes to:
{ "adv": "10", "at": "1626857036533", "cfgId": "null", "channel": "oobe", "cv": "2020072000", "device": "merlinnfc", "ile": "unknown", "make": "xiaomi", "mi": true, "miui": "V12.0.7.0.QJOMIXM", "model": "M2003J15SC", "n": -1, "nonce": "90f50...b9ca7ea73", "region": "IE", "st": "1626857423387", "apn": "com.miui.android.fashiongallery", "event": "active" }

GET http://t5.a.market.xiaomi.com/download/AppStore/07b1d5589f30739af165e04fcd4a44edb4430a8c
<<< HTTP 200, 3.27KB

POST https://tracking.intl.miui.com/track/key_get
secretKey=jPeShAWRSwODWU..amkDug%3D&sign=c75ff...7111b0a861fd6
<<< HTTP 200, 186.00B

This is an exchange of the AES key used to encrypt later telemetry sent to `tracking.intl.miui.com`. The `secretKey` value is an RSA encoded AES key (presumably the server has the private key allowing decryption). The response is an `sid` value and a `key` value. The `sid` value is then sent with later connections to identify the encryption key used.

GET http://t7.a.market.xiaomi.com/thumbnaill/gif/w1000/AdCenter/0fb205a49acb4e3c060e060d6150ef6a88a4331b4
<<< HTTP 206, 1.00B
Followed by several similar connections

POST https://data.mistat.intl.xiaomi.com/key_get
skey_rsa=MYKGrS7gXfZtX5...B5yrkdqLok4%3D
<<< HTTP 200, 271.00B
Similar to the `tracking.intl.miui.com` `key_get` call above.

POST https://api.sec.intl.miui.com/common/whiteList/allList
aid=576ABF0FC0BFD6CC5B11DFA5EE0E413A&av=10&bv=V12&c=&d=merlinnfc&dataVersion=0&e=%5B%5D&ihash=UaPr15RD6JyXS9Vj&im=EEEBAD9FD7521590AD9F715BD07CE7&l=en_US&r=IE&si=29&sign=61B5FB65E2...&t=stable&v=MIUI-V12.0.7.0.QJOMIXM
<<< HTTP 200, 307.55KB
The `aid` value is an MD5 hash of the device secure settings `android_id`. The `ihash` value is the first 16 chars of the base64 encoded SHA1 hash of the `deviceID`, it stays the same across factory resets and so acts as a long-lived device identifier. Similarly the `im` value also stays the same across factory resets.

GET https://find.api.micloud.xiaomi.net/mic/find/v4/anonymous/device/key?cloudsp_fid=73497474313b4f22384e493f73743f50304d2f5776353a4f26324d292e76763e_b288b354cf1b8e61182ee71fb2541f30102dcd96&cloudsp_devId=UaPr15RD6JyXS9Vj&cloudsp_service=allService
The `cloudsp_devId` value matches the `ihash` value in the previous connection, a long-lived device identifier. The `cloudsp_fid` value is the value returned by a call to `getSecurityDeviceId()` from service `miui.sedc` (`SecurityDeviceCredentialManager`). It is a long-lived device identifier that

persists across factory resets.

POST http://global.market.xiaomi.com/apm/intl/statistics/appactive?lo=IE_n=1626857422652_370&p=lo%253Bparams%253B_n&s=irHMnhPjYurjw0Wozx1-V8l&params=d3xH8xwM%2Byc6jFWX0%...Cu7uWTX
<<< HTTP 200, 29.00B

This connection seems to be checking for updates to the installed system apps. The `params` value an RSA encrypted list with details of installed system apps.

GET http://t5.a.market.xiaomi.com/download/AppStore/07b1d5589f30739af165e04fcd4a44edb4430a8c
<<< HTTP 200, 3.27KB

POST https://api.ad.intl.xiaomi.com/getSplashScreenAds
clientInfo={"deviceInfo":{"screenWidth":1080,"screenHeight":2340,"screenDensity":2,"model":"M2003J15SC","device":"merlinnfc","androidVersion":"10","miuiVersion":"V12.0.7.0.QJOMIXM","miuiVersionName":"V12","bc":"S","make":"xiaomi","isInter":true,"os":"android","customizedRegion":"","userInfo":{"locale":"en_US","language":"","en":"country":"IE","customization":"","networkType":-1,"connectionType":"WIFI","ua":"Dalvik/2.1.0 (Linux; U; Android 10; M2003J15SC MIUI/V12.0.7.0.QJOMIXM)","serviceProvider":"","triggerId":"","d31a23421a8abdd19b5f73cdea22eb2f","gaid":"","d90967c6-d79d-4345-8de5-d31357871b0ee"},"isPersonalizedAdEnabled":false},"applicationInfo":{"platform":"xiaomi","packageName":"com.miui.msa.global","version":"2020072000"},"appInfo":{"packageName":"com.miui.securitycenter"},"version":["100481"],"impRequests":[{"template":"5.1.5.4.5.6.5.7.5.12.5.13.5.14.5.15.16"}],"context":{"hasUC":0}}&pre=true&sv=0&nonce=9d2c928f1...637bdc4c&isbase64=false&appKey=system_splash&sign=8578fc5bd...7795ad
<<< HTTP 200, 164.00B

GET https://api.ad.intl.xiaomi.com/track/pi/v1.0?nonce=744df0288...b5860&ts=1626857423813&value=eyJhZHYiOi...16ImFjdG12ZS9&sign=4747ff33...dff0ff3
The "value" parameter base64 decodes to:
{ "adv": "10", "at": "1626857036584", "cfgId": "null", "channel": "oobe", "cv": "2020072000", "device": "merlinnfc", "ile": "unknown", "make": "xiaomi", "mi": true, "miui": "V12.0.7.0.QJOMIXM", "model": "M2003J15SC", "n": -1, "nonce": "744df0...b5860", "region": "IE", "st": "1626857423813", "apn": "com.duokan.phone.remotecontroller", "event": "active" }

POST https://update.intl.miui.com/updates/incompatibleAppsList.php
q=bmoyPE...3D&s=E0A2EA9...71D9FE21&t=1626857423
<<< HTTP 200, 53.00B
The `q` value is AES/CBC encrypted and then base64 encoded. It decodes to:
{ "cc": "10.0", "d": "merlin_global", "pv": "V12.0.1.0.RJOMIXM", "v": "MIUI-V12.0.7.0.QJOMIXM", "tc": "11.0" }

GET https://global.market.xiaomi.com/apm/intl/config?
clientConfigVersionCode=0&co=US&cpuArchitecture=arm64-v8a%2Carneabi-v7a%2Carneabi&densityScaleFactor=2.75&deviceType=0&guid=0e0cfcf747a76f852e7b8b5a0be99a77&installDay=0&instance_id=dTwKkIEwSoCC9HoK3gmx2D&international=1&la=en&launchDay=-1&lo=IE&marketVersion=2000541&mipicksVersion=4001462&miuiBigVersionCode=10&miuiBigVersionName=V12&model=M2003J15SC&network=wifi&os=V12.0.7.0.QJOMIXM&pageConfigVersion=118&productName=discover&resolution=1080*2110&resourceVersionCode=527&ro=unknown&romLevel=24%2C15%2C12&sdk=29&tabVersionCode=118&webResVersion=527&n=1626857422630_190&s=4yECyt1QH7qiMKmHyUXNCBoD4f3&p=clientConfigVersionCode;co;cpuArchitecture;densityScaleFactor;deviceType;guid;installDay;instance_id;international;la;launchDay;lo;marketVersion;mipicksVersion;miuiBigVersionCode;miuiBigVersionName;model;network;os;pageConfigVersion;productName;resolution;resourceVersionCode;ro;romLevel;sdk;tabVersionCode;webResVersion;n
<<< HTTP 200, 1024.00B
The `instance_id` value is the Google Firebase id (`fid`) associated with app `com.xiaomi.discover`.

POST https://data.mistat.intl.xiaomi.com/key_get
skey_rsa=GiY1Q2mCvf1kc...fXc%3D
<<< HTTP 200, 271.00B
This is followed by several similar connections

GET http://fgeb0.market.xiaomi.com/download/AppStore/072724201

Plus many similar connections that send device details and are linked by the same guid value.

GET <http://h6.market.xiaomi.com/download/AppStore/0b3c8cedde6d6443ab21ad97089892e55bfb4d64c>

Headers

User-Agent: AndroidDownloadManager (Linux; U; Android 10; M2003J15SC MIUI/V12.0.7.0.QJOMIXM) (discover; 2000560; 479)
ref: localAutoUpdateAll
<<< HTTP 200, 11.96MB

And similar connections

GET https://firebase-settings.crashlytics.com/spi/v2/platforms/android/gmp/1:231981482949:android:f7a409249c23233916c1ac/settings?instance=ad48cb921e1064d0f947f13c45b1686d0801331a&build_version=2000560&display_version=21.6.17.560&source=4

Headers

X-CRASHLYTICS-DEVELOPER-TOKEN: 470fa2b4a...cec591fa
X-CRASHLYTICS-DEVICE-MODEL: Xiaomi/M2003J15SC
X-CRASHLYTICS-INSTALLATION-ID:
978bd8ea4c6e41aba378c62d19e2f8cf
X-CRASHLYTICS-GOOGLE-APP-ID: 1:231981482949:android:f7a409249c23233916c1ac
<<< HTTP 200, 435.00B

Connection to Google Firebase Crashlytics by app *com.xiaomi.discover*. A similar connection is made by *com.miui.securitycenter*.

POST <https://firebase-remoteconfig.googleapis.com/v1/projects/231981482949/namespaces/fireperf:fetch>

Headers

X-Goog-API-Key: AIzaSyC...oGNwt4fpzK8
X-Android-Package: com.xiaomi.discover
X-Goog-Firebase-Installations-Auth: eyJhbGciOiJIJF...DPA2ptQ1
{"appInstanceId":"c35kOa6BRC6YFGH2MTRv-L","appVersion":21.6.17.560,"countryCode":"US","analyticsUserProperties":{"appId":1:231981482949:android:f7a409249c23233916c1ac,"platformVersion":29,"timeZone":"Europe/Dublin","sdkVersion":21.0.0,"packageName":"com.xiaomi.discover","appInstanceIdToken":"eyJhbGciOi...DPA2ptQ1","languageCode":"en-US","appBuild":2000560}}
<<< HTTP 200, 1.14KB

The X-Goog-Firebase-Installations-Auth header value (which is the same as the appInstanceIdToken value in the POST body) is a jwt token that decodes to:

```
{
  "appId": "1:231981482949:android:f7a409249c23233916c1ac",
  "exp": 1627465148,
  "fid": "c35kOa6BRC6YFGH2MTRv-L",
  "projectNumber": 231981482949
}
```

The fid value is the Firebase ID of app *com.xiaomi.discover*.

GET https://app-measurement.com/config/app/1%3A357317899610%3AAndroid%3A4765c0ded882c665?app_instance_id=191b32bd25304ece33516e277e2c6dcd&platform=android&gmp_version=212418
<<< HTTP 200, 1.04KB

POST <https://app-measurement.com/a>

POST body decoded as protobuf:

<device details>

```
14: "com.google.android.apps.messaging"
16: "8.4.037 (Upas_RC08.phone_dynamic)"
17: 42041
18: 212418
19: "d90967c6-d79d-4345-8de5-07357871b0ee"
20: 0
21: "191b32bd25304ece33516e277e2c6dcd"
22: 7599436411597265477
23: 2
25: "1:357317899610:android:4765c0ded882c665"
26: 1626856951922
28: 1
30: "csXV7kuoRM0EvUXQjXpUST"
```

...
Connection to Google Analytics by *com.google.android.apps.messaging*. Connections are also made by *com.google.android.apps.walletnfcrel*, *com.google.android.googlequicksearchbox*, *com.google.youtube*, *com.xiaomi.discover*, *com.miui.msa.global*, *com.miui.securitycenter*.

POST <https://app-measurement.com/a>

There are periodic connections to Google Analytics by apps *com.google.android.apps.messaging*, *com.google.android.apps.walletnfcrel*, *com.google.android.googlequicksearchbox*, *com.google.youtube*, *com.xiaomi.discover*, *com.miui.msa.global*. Connections are more frequent for the apps *com.miui.msa.global* and *com.google.android.googlequicksearchbox*. The POST body is a protobuf. A decoded example of a message sent by *com.miui.msa.global* is:

```
body {
  always_one: 1
  event {
    event_info {
      setting_code: "ad_switch_off"
      data_int: 1
    }
    event_info {
      setting_code: "ad_switch_reason"
      data_str: "ad_switch_reason_new_protect"
    }
    event_info {
      setting_code: "_o" // firebase_event_origin
      data_str: "app"
    }
    event_info {
      setting_code: "ad_switch_pn"
      data_str: "com.miui.msa.global"
    }
    event_info {
      setting_code: "_c" // firebase_conversion
      data_int: 1
    }
    event_info {
      setting_code: "_r" // realtime
      data_int: 1
    }
    event_code: "event_ad_switch"
    event_timestamp: 1619703816774
    previous_event_timestamp: 1619690374682
  }
  user {
    timestamp: 1619603573281
    setting: "_fot"
    data_int: 1619604000000
  }
  user {
    timestamp: 1619603573281
    setting: "_fi" // first_install
    data_int: 1
  }
  user {
    timestamp: 1619603623964
    setting: "device"
    data_str: "merlinnfc"
  }
  user {
    timestamp: 1619703817740
    setting: "_lte" // lifetime_engagement
    data_int: 1
  }
  message_timestamp: 1619703817730
  event_timestamp: 1619703816774
  bundle_end_timestamp: 1619703816774
  last_bundle_end_timestamp: 1619690374682
  operating_system: "android"
  operating_system_version: "10"
  Build_MODEL: "M2003J15SC"
  language_country: "en-gb"
  timezone_offset_mins: 60
  app_store: "manual_install"
  package_name: "com.miui.msa.global"
  app_version: "2020.07.20.00-release"
  gmp_version: 15300
  gms_version: 211213
  google_ad_id: "a4f87f3f-4661-4334-a8d8-a4e207eb3b7c"
  random_hex: "1af5ecd3e74e2ea8d62f737084728841"
  dev_cert_hash: 10920849302771273491
```

```
daily_conversions_count: 14
gmp_app_id: "1:701165479720:android:6e7c88f234869758"
last_bundle_end_timestamp2: 1619690374681
always_true: true
firebase_instance_id: "fG-3PBoeG-M"
app_version_int: 2020072000
config_version: 1617928841962180
M: 23180934
M: 23180923
M: 23180912
unknown: "G1—"
}
```

POST <https://flash.sec.intl.miui.com/gc/sp/rules>
andApiVer=29&miuiCompVer=stable&miuiVer=V12.0.7.0.QJOMIXM&
appVer=stub+%2855.1.0%29&sign=B30AA1C...39EB3E89A&ruleVer=1&
sdkVer=1.0.0&lang=en_US&pkg=com.facebook.katana&device=
M2003J15SC&oid=&andVer=10
<<< HTTP 200, 404.00B

POST <https://flash.sec.intl.miui.com/gc/sp/rules>
andApiVer=29&miuiCompVer=stable&miuiVer=V12.0.7.0.QJOMIXM&
appVer=&sign=70DD806E...7094D&ruleVer=1&sdkVer=1.0.0&lang=en_US
&pkg=com.whatsapp&device=M2003J15SC&oid=&andVer=10
<<< HTTP 200, 5.51KB

POST <https://au.ff.avast.sec.miui.com/>
data=CHgQAR...TRIYjgz
<<< HTTP 403, 269.00B
Value of "data" base64 decodes to:
Android??s??#?
M2003J15SC?Xiaomi?\$409a1088-2d59-4fc7-a407-de92ba390af8??
P50D8B4A941C26B89482C94AB324...53F42820AEBD52C?1.0.8?1?com.
miui.guardprovider?AV_SDK?QP1A.190711.020?Redmi??10?&\$409a1088-
2d59-4fc7-a407-de92ba390af8??4(
f8d5ba2a5b2488abd09b9a22ec96000473a4eb83
Followed by several similar connections.

POST <https://mcc.intl.inf.miui.com/cloud/app/getData>
appVersion=61&packageName=com.milink.service&versionName=1.1.61&
version=0&deviceInfo=%7B%22uid%22%3A%2222447d40-3915-40a0-
b381-7faa6a8079af%22%2C%22d%22%3A%22merlinnfc%22%2C%22r
%22%3A%22IE%22%2C%22l%22%3A%22en_US%22%2C%22v%22%3A
%22V12.0.7.0.QJOMIXM%22%2C%22bv%22%3A%22V12%22%2C%22t
%22%3A%22stable%22%2C%22av%22%3A%2210%22%2C%22p%22%3A
%22android%22%7D&sign=D4DC0F88...74E099BC8
<<< HTTP 200, 56.00B

POST <https://firebaselogging-pa.googleapis.com/v1/firelog/legacy/batchlog>
Headers
User-Agent: datatransport/3.0.0 android/
X-Goog-API-Key: AlZaSyCck...ld15aOG8ozKo
{ "logRequest": { "requestTimeMs": 1626864684310, "requestUptimeMs": 7887490, "clientInfo": { "clientType": "ANDROID_FIREBASE", "androidClientInfo": { "sdkVersion": 29, "model": "M2003J15SC", "hardware": "mt6769z", "device": "merlinnfc", "product": "merlinnfc_global", "osBuild": "QP1A.190711.020", "manufacturer": "Xiaomi", "fingerprint": "Redmi/merlinnfc_global/merlinnfc:10/QP1A.190711.020/V12.0.7.0.QJOMIXM:user/release-keys", "locale": "en", "country": "US", "mccMnc": "", "applicationBuild": "2000560" }, "logSourceName": "FIREPERF", "logEvent": { "eventTimeMs": 1626864628015, "eventUptimeMs": 7831195, "sourceExtension": "CnUKLTE6MjMxO...yNzly", "timezoneOffsetSeconds": 3600, "networkConnectionInfo": { "networkType": "WIFI", "mobileSubtype": "UNKNOWN_MOBILE_SUBTYPE" }, "eventTimeMs": 1626864629022, "eventUptimeMs": 7832203, "sourceExtension": "CnUKLTE6...5MjcyMg==", "timezoneOffsetSeconds": 3600, "networkConnectionInfo": { "networkType": "WIFI", "mobileSubtype": "UNKNOWN_MOBILE_SUBTYPE" }, "qosTier": "DEFAULT" } } }
<<< HTTP 200, 151.00B
The sourceExtension base64 decodes to:
-1:231981482949:android:
f7a409249c23233916c1acc35kOa6BRC6YFGH2MTRv-L*
com.xiaomi.discover20.0.121.6.17.560
=https://global.market.xiaomi.com/apm/intl/updateinfo/diffsize...
04769fbcdbde4e28826436f0c1f92722

POST <https://firebaseinstallations.googleapis.com/v1/projects/security-center>

-89b35/installations

Headers

```
X-Android-Package: com.miui.securitycenter
x-firebase-client: device-name/merlinnfc_global device-brand/Redmi
android-min-sdk/23 device-model/merlinnfc fire-android/29 fire-cl/17.4.1
fire-core/19.5.0 android-installer/com.xiaomi.discover android-platform/
android-target-sdk/29 fire-installations/16.3.5 fire-analytics/18.0.3
{"fid":"e8H4ayluRDCjnN2p7LBC03","appId":"1:436805358632:android:6
af670fc95cb7011dfd11d","authVersion":"FIS_v2","sdkVersion":"a:16.3.5"}
<<< HTTP 200, 583.00B
```

GET https://www.google.com/complete/search?oe=utf-8&safe=images&gcc=ie&ctzn=Europe%2FDublin&ctf=0&v=12.27.9.23.arm64&ntyp=1&ram_mb=3754&ar=0&inm=asst&hl=en-US&noj=1&client=qsab-android-asbl-pb&qsubts=1626868504761&padt=200&padb=768&cs=0&cds=2&psm=0&gs_pcr=t&q=&cp=0&psi=Lhdntzq5rXc.1626857796076.0&ech=2&dpr=2.75&gs_pcr=1&xssi=t&getexp=1
Headers

```
x-client-data: asURH4sIAAA...AwAAA
cookie: CONSENT=PENDING+671
<<< HTTP 200, 25.05KB
```

And several similar connections.

POST <https://businesscalls.googleapis.com/google/communications.businesscalls.mobile.v1.MobileService/RecordVcallUserConsent>
Headers
user-agent: com.google.android.dialer/7603277 (Linux; U; Android 10; en_US; M2003J15SC; Build/QP1A.190711.020; Cronet/91.0.4472.101) grpc-java-cronet/1.39.0-SNAPSHOT
x-goog-spatula: CjkKGWN...K7Rtw=

```
2: 2
3: 1
7: "Dialer-67.0.383690429"
8 {
  1: 1626860785
  2: 443000000
}
12: "2021-07-21T10:27:11.096Z"
13: "0"
16: "dRaokJ3SQmu46khyPOYkSv"
```

And many similar connections by com.google.android.dialer, also to https://growth-pa.googleapis.com/google.internal.identity.growth.v1.GrowthApiService/GetPromos.

GET https://www.googleadservices.com/pagead/conversion/1001680686/?bundleid=com.google.android.youtube&appversion=16.27.34&osversion=10&sdkversion=ct-sdk-a-v2.2.4&gms=1&lat=0&rrid=d90967c6-d79d-4345-8de5-07357871b0ee&timestamp=1626872277.585&remarketing_only=1&usage_tracking_enabled=0&data.screen_name=%3CAndroid_YT_Open_App%3E
Headers

```
user-agent: com.google.android.youtube/1521997248 (Linux; U;
Android 10; en_US; M2003J15SC; Build/QP1A.190711.020; Cronet
/93.0.4557.4)
<<< HTTP 200, 0.00B
```

And several similar connections.

POST <https://privacy.api.intl.miui.com/collect/privacy/agree/v1?r=IE>
Headers
x-mi-xkey: 9049bb719...kJXdaw==
The POST body is AES encrypted. The key is protected (the x-mi-xkey header value is not the key, just an identifier for the key) but can be extracted by appropriately hooking the com.miui.msa.global app using Frida. An example decoded payload is:
{ "pkg": "com.miui.msa.global", "timestamp": 1626875745373, "idType": "3_0", "idContent": "d90967c6-d79d-4345-8de5-07357871b0ee", "miuiVersion": "V12.0.7.0.QJOMIXM", "apkVersion": "2021.06.29.00-release", "language": "en_US", "region": "IE" }

POST <https://api.ad.intl.xiaomi.com/post/302>

Headers

```
x-mi-xkey: 9049bb71971...akJXdaw==
```

The POST body is AES encrypted. The key is protected (the x-mi-xkey header value is not the key, just an identifier for the key) but can be extracted by appropriately hooking the com.miui.msa.global app using Frida. An example decoded payload is:
clientInfo={ "deviceInfo": { "screenWidth": 1080, "screenHeight": 2340, "

```
screenDensity":2,"model":"M2003J15SC","device":"merlinfc","
androidVersion":"10","miuiVersion":"V12.0.7.0.QJOMIXM","
miuiVersionName":"V12","bc":"S","make":"xiaomi","isInter":true,"os":
"android","modDevice":"merlin_global","customizedRegion":"","restrictIimei":false},
"userInfo":{"locale":"en_US","language":"en","country":"IE",
"customization":"","networkType":"-1","connectionType":"WIFI","ua":"Dalvik
/2.1.0 (Linux; U; Android 10; M2003J15SC MIUI/V12.0.7.0.QJOMIXM)
","serviceProvider":"","gaid":"d90967c6-d79d-4345-8de5-07357871b0ee",
"isPersonalizedAdEnabled":false},"applicationInfo":{"platform":"xiaomi",
"packageName":"com.miui.msa.global","version":2021062900},"context":{"
hasUc":0},"impRequests":[{"tagId":"1.317.1.1"}]}&v=3.0&isbase64=false&
appKey=MSA_GLOBAL_REDIRECT_URL&sign=
e5ea02cabd32b8a8da90702a64557d85
```

POST https://api.ad.intl.xiaomi.com/brand/splashConfig
Headers

x-mi-xkey: 9049bb71971...akJXdaw=

POST https://api.ad.intl.xiaomi.com/brand/pushConfig
Headers

x-mi-xkey: 9049bb71971...JXdw==

POST https://api.ad.intl.xiaomi.com/interRemoteConfig
Headers

x-mi-xkey: 9049bb71971f...kJXdaw==

The payloads in these three POST connections is AES encrypted, contents are similar to api.ad.intl.xiaomi.com/post/302 connection.

POST https://data.mistat.intl.xiaomi.com/get_all_config
rc=S&sv=3.0.16&t=2&av=2.2.18-global&rg=IE&ai=2882303761517492012&m=M2003J15SC
<<< HTTP 200, 322.00B

POST https://data.mistat.intl.xiaomi.com/key_get
skkey_rsa=UiEdGiZs...4DRSc%3D

POST https://data.mistat.intl.xiaomi.com/mistats/v3
p=VcntdiKLZ...eu9G6ew=&sv=3.0.19&rg=IE&pv=3.0&ai=2882303761517327742&fc=0&sid=180b4c...fef03c1d&sn=64807e04ba74bf9cb8c844ae48b3e819
<<< HTTP 200, 47.00B

The p parameter value is AES encrypted.

GET https://api.setting.intl.miui.com/setting/v1/config?app_id=GLOBAL_SETTING&cids=global_setting&d=M2003J15SC&l=en&miui_version=V12&os_version=10&pkg=com.android.settings&r=IE&sign=4a09c97...b2294d&t=stable&timestamp=1626875996724
<<< HTTP 200, 217.00B

POST https://tracking.intl.miui.com/track/v4

Headers
OT_SID: 1904b90...536c63d4
OT_ts: 1626876907134
OT_net: WIFI
OT_sender: com.miui.analytics
OT_protocol: 3.0

And several similar connections. The POST body is AES encrypted. The key is protected but can be extracted by appropriately hooking the com.miui.analytics app using Frida. It decodes to json that consists of a header with handset details and the Android ID, followed by a series of event entries. An example header is:

```
"H": {
  "event": "onetrack_usage",
  "gaid": "d90967c6-d79d-4345-8de5-07357871b0ee",
  "oaid": "",
  "mfrs": "Xiaomi",
  "model": "M2003J15SC",
  "platform": "Android",
  "miui": "V12.0.7.0.QJOMIXM",
  "build": "S",
  "os_ver": "10",
  "app_ver": "2.44.0",
  "e_ts": 1626858269248,
  "tz": "GMT",
  "net": "WIFI",
  "region": "IE",
  "user_id": 0,
  "app_id": "001",
  "pkg": "com.miui.analytics"
```

}
One type of event appears to consist of a timestamped sequence of user actions. For example the following seems to be associated with opening the Settings app, enabling Wifi and entering the Wifi password :

```
"seq": [
  {
    "event": 2,
    "pkg": "com.miui.home",
    "class": "com.miui.home.launcher.Launcher",
    "ts": 1626857404139,
    "vn": "RELEASE-4.17.2.2233-02231157",
    "duration": 0,
    "stat": "app_end",
    "app_duration": 0
  },
  {
    "event": 1,
    "pkg": "com.android.settings",
    "class": "com.android.settings.MiuiSettings",
    "ts": 1626857404147,
    "vn": "10",
    "stat": "app_start"
  },
  {
    "event": 2,
    "pkg": "com.android.settings",
    "class": "com.android.settings.MiuiSettings",
    "ts": 1626857408304,
    "vn": "10",
    "duration": 4157
  },
  {
    "event": 1,
    "pkg": "com.android.settings",
    "class": "com.android.settings.Settings$WifiSettingsActivity",
    "ts": 1626857408317,
    "vn": "10"
  },
  {
    "event": 10001,
    "pkg": "com.google.android.inputmethod.latin",
    "ts": 1626857410690,
    "vn": "9.9.14.333092878-release-arm64-v8a"
  },
  {
    "event": 10002,
    "pkg": "com.google.android.inputmethod.latin",
    "ts": 1626857419855,
    "vn": "9.9.14.333092878-release-arm64-v8a",
    "duration": 9165
  },
  {
    "event": 1,
    "pkg": "com.miui.home",
    "class": "com.miui.home.launcher.Launcher",
    "ts": 1626858265233,
    "vn": "RELEASE-4.17.2.2233-02231157"
  },
  {
    "event": 2,
    "pkg": "com.android.settings",
    "class": "com.android.settings.Settings$WifiSettingsActivity",
    "ts": 1626858265259,
    "vn": "10",
    "duration": 856942,
    "stat": "app_end",
    "app_duration": 861112
  }
]
```

Another type of event seems to be associated with app installation and updating, e.g.

```
"apps": [
  {
    "pkg": "com.miui.screenrecorder",
    "vn": "1.7.1",
```

```

"vc": 71,
"installer": "",
"fit": 1230768000000,
"lut": 1230768000000,
"status": 4
},
<similar entries for com.google.android.apps.subscriptions.red,
com.google.android.youtube, com.agoda.mobile.consumer, com.google.
android.googlequicksearchbox, cn.wps.xiaomi.abroad.lite, com.miui.fm, com
.google.android.apps.googleassistant, com.xiaomi.account, com.miui.gallery,
com.xiaomi.payment etc>
Another type of entry transmits and links various device identifiers, e.g.
"H": {
  "event": "onetrack_active",
  "gaid": "d90967c6-d79d-4345-8de5-07357871b0ee",
  "oaid": "",
  "instance_id": "365cecdc-5d51-4529-9d53-051e3cb6c78a",
  <device details>
  "pkg": "com.miui.analytics"
},
"B": {
  "real_model": "M2003J15SC",
  "product": "merlinnfc_global",
  "device": "merlin_global",
  "device_type": "Phone",
  "screen": "2340*1080",
  "imei": "593654058b07cea81dace906b0774c0c",
  "sn": "",
  "android_id": "49ec933a0be9eb7a",
  "vaid": "465dddbcd3dc3dcd",
  "udid": "",
  "mac": "e73b19880deb349abe6facb2332fac4f",
  "imeis": "[
593654058b07cea81dace906b0774c0c,eeebaded9fd7521590ad9f715bd07ce7]",
  "meids": "",
  "imsis": "[,]",
  "oaid_stat": 1,
  "language": "en_US",
  "ram": "4GB",
  "rom": "128GB",
  "free_rom": "107.33GB",
  "ui_ver": "V12",
  "android_ver_int": 29,
  "uep": false,
  "sign": "701478a1",
  "cust_variant": "ie",
  "intl": true,
  "desc": "merlinnfc-user 10 QP1A.190711.020 release-keys",
  "radio": "",
  "radio2": "",
  "first_boot": true,
  "bind_stats": "0",
  "release_time": 1470758400000,
  "first_connect_time": 1626876913000,
  "lock_state": "unlocked",
  "rootable": "0",
  "tz_content": "a2zddIIB...PZZuAyA=",
  "tz_sign": "30440...c426a",
  "tz_fid": "
73497474313b4f22384e493f73743f50304d2f5776353a4f26324d292e76763e_
b288b354cf1b8e61182ee71fb2541f30102dcd96",
  "tz_support": true,
  "tz_cpuid": "0xb288b354cf1b8e61182ee71fb2541f30102dcd96"

```

The imei/imeis values are MD5 hashes of the handset IMEIs (it has two SIM slots, so two IMEI values). The mac value is an MD5 hash of the handset Wifi MAC address. The android_id value is a device identifier (but seems distinct from the Google AndroidID), and the vaid value also appears to be a device identifier. The tz_fid value is the value returned by a call to getSecurityDeviceCred() from service miui.sedc (SecurityDeviceCredentialManager), it is a long-lived device identifier that persists across factory resets. The tz_cpuid value is the value of the handset ro.boot.cpuid property. The tz_content value is RSA encrypted (so triply-encrypted using SSL, AES and RSA) and decrypts to:

```

{"imei1":"866077052332823","imei2":"866077052332831","sn":"","i1":"","i2":""}

```

The imei1 and imei2 are the IMEIs of the two handset slots (this time not

hashed). When a SIM is inserted into slot 1, see later, the i1 value reported is the SIM IMSI.

GET https://find.api.micloud.xiaomi.net/mic/find/v4/anonymous/challenge?cloudsp_fid=73497474313b4f22384e493f73743f50304d2f5776353a4f26324d292e76763e_b288b354cf1b8e61182ee71fb2541f30102dcd96&cloudsp_devId=UaPr15RD6JyXS9Vj

And several similar connections.

POST [https://mcc.intl.inf.miui.com/cloud/app/getDataappVersion=61&packageName=com.milink.service&versionName=1.1.61&version=0&deviceInfo={"uid":"22447d40-3915-40a0-b381-7faa6a8079af","d":"merlinnfc","r":"IE","l":"en_US","v":"V12.0.7.0.QJOMIXM","bv":"V12","t":"stable","av":"10","p":"android"}&sign=D4DC...E099BC8](https://mcc.intl.inf.miui.com/cloud/app/getDataappVersion=61&packageName=com.milink.service&versionName=1.1.61&version=0&deviceInfo={)
<<< HTTP 200, 56.00B

GET <https://resolver.msg.global.xiaomi.net/gslb/?ver=4.0&type=wifi&uuid=0&list=fr.app.chat.global.xiaomi.net%2Cresolver.msg.global.xiaomi.net&countrycode=IE&sdkver=41&osver=29&os=M2003J15SC%3AV12.0.7.0.QJOMIXM&mi=3&key=59a16f...e5c4d5b9>
<<< HTTP 200, 2.80KB

POST https://flash.sec.intl.miui.com/gc/sp/rulesandApiVer=29&miuiCompVer=stable&miuiVer=V12.0.7.0.QJOMIXM&appVer=&sign=70DD806..E67094D&ruleVer=1&sdkVer=1.0.0&lang=en_US&pkg=com.whatsapp&device=M2003J15SC&oaid=&andVer=10
<<< HTTP 200, 5.51KB

POST <https://moaps.tmo.net/events>

Headers

```

Authorization: u1fEuOeizp_fN9nzMK0QHxNzCiguxaVDfcUbaP5q
Transfer-Encoding: chunked
{"events":[{"_uid":"cdc229b6-6ffc-471d-97de-9fd58f02e2e1","_event":
content="download","contentDownloadReason":"scheduled_loading",
contentDownloadTrigger":"SCHEDULED_UPDATE","manufacturer":
Xiaomi","device-model":"M2003J15SC","device-type":"OM","brand_id":
xx","client":"de.telekom.tsc","client-version":"9.2.0","os-version":"10",
created-time":"2021-07-21T09:50:23.559+0100","fw-build-number":
QP1A.190711.020}],{"_uid":"cdc229b6-6ffc-471d-97de-9fd58f02e2e1",
_event":"content-download","contentDownloadReason":"scheduled_loading",
contentDownloadTrigger":"SCHEDULED_UPDATE","manufacturer":
Xiaomi","device-model":"M2003J15SC","device-type":"OM","brand_id":
xx","client":"de.telekom.tsc","client-version":"9.2.0","os-version":"10",
created-time":"2021-07-21T09:50:30.417+0100","fw-build-number":
QP1A.190711.020}]]}
<<< HTTP 200, 15.00B

```

Additional connections when logged in to Google:

Connections to Google servers are similar to those for the Samsung handset, in particular connections to:

- android.googleapis.com/auth,
- inbox.google.com,
- mail.google.com,
- lamssettings-pa.googleapis.com,
- youtubei.googleapis.com,
- googleapis.com,i.ytimg.com,
- www.googleadservices.com,
- app-measurement.com,
- www.google.com/complete/search,
- footprints-pa.googleapis.com,
- people-pa.googleapis.com,
- geller-pa.googleapis.com.

POST <https://graph.facebook.com/v3.3/2263965750590514/activities>

Headers

```

User-Agent: FBAndroidSDK.5.1.1
format=json&sdk=android&custom_events=[{"_eventName":
fb_mobile_deactivate_app","_eventName_md5":"92255b491...3665affe",
_logTime":"1619690511","_ui":"LoginActivity","_session_id":"8c632d70-
e71e-4017-a0c7-32d922341c3f","fb_mobile_time_between_sessions":
session_quanta_0","fb_mobile_launch_source":"Unclassified",
fb_mobile_app_interruptions":"1","_valueToSum":58.318,"_inBackground":
"1"}]&event=CUSTOM_APP_EVENTS&advertiser_id=d90967c6-d79d-
4345-8de5-07357871b0ee&advertiser_tracking_enabled=true&anon_id=
XZ72bca91c-7ba4-426d-9f0f-04f95fb4ee9&application_tracking_enabled=
true&extinfo=[a2","com.xiaomi.account",12000016,"12.0.0.16","10",
M2003J15SC","en_GB","GMT+01:00",

```

Tesco+Mobile,1080,2110,"2.75",8,105,103,"Europe\Dublin"]&
application_package_name=com.xiaomi.account&
<<<< HTTP 200, 16.00B
Shortly after Google account login, a request is sent to Facebook when
includes the Google adid/rdid and also mobile operator details.

POST https://mcc.intl.miui.com/cloud/app/getData
appVersion=61&packageName=com.milink.service&versionName=1.1.61&
version=0&deviceInfo={"uid":"cddfabbe-5d0f-4b7c-9e88-9250c864539a",
d":"merlinnfc","r":"IE","l":"en_GB","v":"V12.0.7.0.QJOMIXM","bv":"V12
","t":"stable","av":"10","p":"android"}&sign=101...EC852A
<<<< HTTP 200, 56.00B

POST https://moaps.tmo.net/events
Headers

Authorization: **u1fEuOei...aVDFcUbaP5q**
{ "events": [{"_uid": "7a916e86-9640-4a1e-be16-821d00eefec", "_event": "
content-download", "contentDownloadReason": "scheduled_loading",
contentDownloadTrigger": "SCHEDULED_UPDATE", "manufacturer": "
Xiaomi", "device-model": "M2003J15SC", "device-type": "OM", "brand_id": "
xx", "client": "de.telekom.tsc", "client-version": "9.2.0", "os-version": "10", "
created-time": "2021-04-28T10:56:45.250+0100", "fw-build-number": "
QP1A.190711.020"}], {"_uid": "7a916e86-9640-4a1e-be16-821d00eefec", "
_event": "content-download", "contentDownloadReason": "scheduled_loading
", "contentDownloadTrigger": "SCHEDULED_UPDATE", "manufacturer": "
Xiaomi", "device-model": "M2003J15SC", "device-type": "OM", "brand_id": "
xx", "client": "de.telekom.tsc", "client-version": "9.2.0", "os-version": "10", "
created-time": "2021-04-28T10:56:49.616+0100", "fw-build-number": "
QP1A.190711.020"}]}"
<<<< HTTP 200, 15.00B

POST https://app-measurement.com/a
POST body decoded as protobuf:

```
<...>
  1: "ad_switch_reason"
  2: "ad_switch_reason_new_protect"
<...>
 14: "com.miui.msa.global"
<...>
```

Connections to Google Analytics app-measurement.com are made by
com.miui.msa.global, com.miui.home, com.google.android.youtube.

Additional connections when location enabled

POST https://tracking.intl.miui.com/track/v4
Headers

OT_SID: 1904b90...536c63d4
OT_ts: 1626983557245
OT_net: WIFI
OT_sender: com.miui.analytics
<...>

```
"B": {
  "start": 1626982536876,
  "end": 1626983436977,
  "radio": "27205",
  "radio2": "",
  "seq": [
    {
      "event": 1,
      "pkg": "com.miui.home",
      "class": "com.miui.home.launcher.Launcher",
      "ts": 1626982570048,
      "vn": "RELEASE-4.17.2.2233-02231157",
      "stat": "app_start"
    },
    {
      "event": 2,
      "pkg": "com.android.settings",
      "class": "com.android.settings.SubSettings",
      "ts": 1626982570068,
      "vn": "10",
      "duration": 240751,
      "stat": "app_end",
      "app_duration": 248264
    }
  ]
}
```

}
Xiaomi telemetry records the user interaction with the Settings app to
enable location.

Connections When Insert Sim

POST https://tracking.intl.miui.com/track/v4
Headers

OT_SID: 1904b90139...3536c63d4
OT_ts: 1626980404564
OT_net: WIFI
OT_sender: com.miui.analytics
OT_protocol: 3.0
The POST decrypted POST body is:
{
 "H": {
 "event": "onetrack_active",
 "gaid": "d90967c6-d79d-4345-8de5-07357871b0ee",
 "oaid": "",
 "instance_id": "365cecdde-5d51-4529-9d53-051e3cb6c78a",
 "mfrs": "Xiaomi",
 "model": "M2003J15SC",
 "platform": "Android",
 "miui": "V12.0.7.0.QJOMIXM",
 "build": "S",
 "os_ver": "10",
 "app_ver": "2.57.0",
 "e_ts": 1626980403225,
 "tz": "GMT+00:00",
 "net": "WIFI",
 "region": "IE",
 "user_id": 0,
 "app_id": "001",
 "pkg": "com.miui.analytics"
 },
 "B": {
 "real_model": "M2003J15SC",
 "product": "merlinnfc_global",
 "device": "merlin_global",
 "device_type": "Phone",
 "screen": "2340*1080",
 "imei": "593654058b07cea81dace906b0774c0c",
 "sn": "",
 "android_id": "49ec933a0be9eb7a",
 "vaid": "465ddbcdb3dc3dcc",
 "udid": "",
 "mac": "e73b19880deb349abe6fach2332fac4f",
 "imeis": "[593654058b07cea81dace906b0774c0c,
eeebaded9fd7521590ad9f715bd07ce7]",
 "meids": "",
 "imsis": "[cd8eaa2b2fba95f5fcd9802c54d62f22,]",
 "oaid_stat": 1,
 "language": "en_US",
 "ram": "4GB",
 "rom": "128GB",
 "free_rom": "107.20GB",
 "ui_ver": "V12",
 "android_ver_int": 29,
 "uep": false,
 "sign": "701478a1",
 "cust_variant": "ie",
 "intl": true,
 "desc": "merlinnfc-user 10 QP1A.190711.020 release-keys",
 "radio": "",
 "radio2": "",
 "first_boot": true,
 "bind_stats": "0",
 "release_time": 1470758400000,
 "first_connect_time": 1626876913000,
 "lock_state": "unlocked",
 "rootable": "0",
 "tz_content": "OLXpogDf6...+uyAHWrdPxrq0=",
 "tz_sign": "3045022055e...72308",
 "tz_fid": ""
 }
}

73497474313b4f22384e493f73743f50304d2f5776353a4f26324d292e76763e_
b288b354cf1b8e61182ee71fb2541f30102dcd96",

```

    "tz_support": true,
    "tz_cpuid": "0xb288b354cf1b8e61182ee71fb2541f30102dcd96"
  }
}
The imsis value is a hash of the SIM IMSI (that uniquely identifies the SIM).
The tz_content value is RSA encrypted and then base64 encoded, it decrypts
to:
{"imei1":"866077052332823","imei2":"866077052332831","sn":"","i1":"
272110104345142","i2":""}
The i1 value is the SIM IMSI (not hashed). The IMEI values are the IMEIs
of the two handset SIM slots.

```

POST <https://android.clients.google.com/fdfe/uploadDynamicConfig>

Headers

```

x-dfe-device-id: 37224f3dad58187c
x-dfe-device-config-token: CisaKQoTM..xMTYz
x-dfe-device-checkin-consistency-token: ABFEt1W..Z7bI
x-dfe-mccmnc: 27211
x-dfe-client-id: am-android-xiaomi
x-dfe-phenotype: H4sL...EAAA
x-dfe-encoded-targets: CAESBPYi...YvKrwY

```

POST body:

```

1 {
  1: "GMT+00:00"
  2 {
    1 {
      1: 272110104300000 // Truncated IMSI
      2: "Tesco Mobile"
      3: "0AFFFFFFFFFFFFFFFFFFFFFFFFFFFFFFFFFFFFFFFF"
      6: 2154
      7: 18446744073709551615
    }
  }
  4: "e7gthBCASvGd_VhTNaY5qd:APA9...7F"
}
<<< HTTP 200, 102.00B

```

POST <https://android.googleapis.com/checkin>

Cookie: NID=219=b8nxLQR93...Mfs

```

1: "866077052332823" // IMEI
2: 4195921141417082689 // AndroidID
3: "1-bb737db53d5e95bcae090abbf82a7da06fc31659"
4 {
  1 {
    1: "Redmi/merlinnfc_global/merlinnfc:10/QP1A.190711.020/V12.0.7.0.
QJOMIXM:user/release-keys"
    2: "mt6769z"
    3: "Redmi"
    4: "MOLY.LR12A.R3.MP.V98.P80,MOLY.LR12A.R3.MP.V98.P80"
    5: "unknown"
    6: "android-xiaomi"
    7: 1614092848
    8: 212418037
    9: "merlinnfc"
    10: 29
    11: "M2003J15SC"
    12: "Xiaomi"
    13: "merlinnfc_global"
  }
}
<...>
6: "27202" // Mobile carrier MNC
7: "27211" // Mobile carrier MCC
8: "WIFI:"
9: 0
14: 2
15 {
  1: 5
  2: 1
  3: "unspecified"
  4: ""
  5: 0
}
16 {
  1: "27211"
  2: "3IRL\342\200\224TescoMobile" // Mobile carrier
  3: "0"
  4: 1

```

```

4: 2
6: "272110104300000" // Truncated SIM IMSI
7: "0AFFFFFFFFFFFFFFFFFFFFFFFFFFFFFFFFFFFFFFFF" //Group ID
Level 1
8: "T\232"
9: 2154
}
18: 1
19: "WIFI"
21: 2154
6 {
  12: 0x53552d6e // Handset Wifi MAC address
}
9: "509839f6e54e"
11: ""
12: "Europe/Dublin"
13: 0x2f6f8c7835ebf144 // security token
14: 3
15: "BjEWgNvC9cNFbRt/mD9RhOsfcls="
16: "102dcd960409" // Handset hardware serial number
<device hardware and software details>
19: "wifi"
20: 0
22: 0
24: "CgZKtoH...L // DroidGuardResultsRequest binary blob, contents
unknown
Google logs the SIM details together with a collection of device identifiers.

```

POST https://www.facebook.com/adnw_sync2

Headers

```

Accept-Charset: UTF-8
payload={"request":{"prefetch_urls":"update","bidder_token_info":"update
"},"bundles":{"prefetch_urls":{"fingerprint":{"last_prefetch_timestamp
":0},"bidder_token_info":{"fingerprint":null},"context":{"COPPA":"false
"},"APPBUILD":"20210506","ID_CACHE_TS_MS":"-1","
KG_RESTRICTED":"false","CAPPED_IDS":"","VALPARAMS":{"
is_emu":"","false","apk_size":"","21196163","timezone_offset
":"","0","app_started_reason":"","LAUNCHER_FOUND_API21","
is_debuggable":"","false","debug_value":"","N\\A\\","build_type":"","
N\\A\\"},"UNITY":"false","ACCESSIBILITY_ENABLED":"false","
APPNAME":"File+Manager","HAS_EXOPLAYER":"true","AFP":"
60d6d62ac3329edbc75a6938a848be44"},"SESSION_TIME
":"","1627012585.328","PLACEMENT_ID":"","MAKE":"Xiaomi","
REQUEST_TIME":"1627023402.673","CARRIER":"Tesco+Mobile","
SDK_CAPABILITY":["3,4,5,7,11,16,17,18"],"TEMPLATE_ID":"0","
CLIENT_REQUEST_ID":"be447347-23c6-41f6-8473-3c9525699692","
DENSITY":"2.75","AD_REPORTING_CONFIG_LAST_UPDATE_TIME
":"","0","SCREEN_HEIGHT":"767","SDK_VERSION":"5.10.0","
SCREEN_WIDTH":"392","ID_SOURCE":"NO_GMS","SDK":"android","
OSVERS":"10","APP_MIN_SDK_VERSION":"19","OS":"Android","
ANALOG":{"total_memory":"","3936739328","accelerometer_y
":"","1.5","rotation_x":"","-0.0019005437","accelerometer_x
":"","1.365","accelerometer_z":"","9.581","charging":"","1","
available_memory":"","1604005888","rotation_z":"","-7.8486523E
-4","rotation_y":"","7.7926955E-4","battery":"","100.0","free_space
":"","107181363200"},"DATA_PROCESSING_OPTIONS":"null","
ROOTED":"1","MODEL":"M2003J15SC","BUNDLE":"com.mi.android.
globalFileexplorer","ASHAS":"","
a1a11dfb0ea9abee25a310a1c3b40def38e6734","LOCALE":"en_US","
NETWORK_TYPE":"","0","IDFA":"","","ATtribution_ID":"","","APPVERS":"
V1-210506","DATA_PROCESSING_OPTIONS_COUNTRY":"","null","
INSTALLER":"com.android.vending","
DATA_PROCESSING_OPTIONS_STATE":"","null","
BIDDER_TOKEN_EXTRAS":{"ip":"","37.228.208.120"},"
IDFA_FLAG":"","0","SESSION_ID":"","c6572577-496b-4142-8775-954
a44c385ec"}}
<<< HTTP 200, 203.00B

```

Connection to Facebook logs mobile carrier name.

C. Connections When Interacting With Settings App

GET https://privacy.mi.com/all/en_US

GET <https://privacy-policy.truste.com/privacy-seal/seal?rid=2a8dc25b-165b-449b-aeb4-fea7228974fa>

GET https://cdn.cnbj1.fds.api.mi-img.com/mcfe--privacy-markdown-fe/static/page-data/all/en_US/page-data.1626831905339.json

Headers

```
origin: https://privacy.mi.com
x-requested-with: com.android.htmlviewer
sec-fetch-site: cross-site
sec-fetch-mode: cors
referer: https://privacy.mi.com/
```

<<< HTTP 200, 62.69KB

And a series of similar connections when viewing the privacy policy screen.

GET https://graph.facebook.com/v3.2/2263965750590514/mobile_sdk_gk?fields=gatekeepers&format=json&sdk_version=5.1.1&sdk=android&platform=android

Headers

```
User-Agent: FBAndroidSDK.5.1.1
```

<<< HTTP 200, 1.81KB

POST https://graph.facebook.com/v3.3/2263965750590514/activities

Headers

```
User-Agent: FBAndroidSDK.5.1.1
```

```
format=json&sdk=android&event=MOBILE_APP_INSTALL&advertiser_id=d90967c6-d79d-4345-8de5-07357871b0ee&advertiser_tracking_enabled=true&installer_package=com.xiaomi.discover&anon_id=XZ89003f4a-fd27-41f0-8206-23cdb19c6a8e&application_tracking_enabled=true&extinfo=["a2","com.xiaomi.account",12010005,"12.1.0.5","10","M2003J15SC","en_US","GMT+01:00","Tesco+Mobile",1080,2110,"2.75",8,105,100,"Europe\Dublin"]&application_package_name=com.xiaomi.account&
```

<<< HTTP 200, 16.00B

And a sequence of similar connections when the Mi Account page in the Settings app is opened.

POST https://global.market.xiaomi.com/apm/intl/updateinfo/v2?<...>guid=0e0cfcf747a76f852e7b8b5a0be99a77&installDay=1&instance_id=c35kOa6BRC6YFGH2MTRv-L<...>

Sends details of installed apps, checking for updates.

GET https://app-measurement.com/config/app/1%3A231981482949%3Aandroid%3Aa7a409249c2323916c1ac?app_instance_id=84121acd84fb1785eaaea323e8c86087&platform=android&gmp_version=212418

Sends data related to the update activity to Google Analytics.

GET https://pagead2.googlesyndication.com/pagead/gen_204?id=gmob-apps&action=ads_settings_page_view&device=Xiaomi%20M2003J15SC&js=212418037.212418000&os=10&api=29&eids=318497819,318475418,318489659,318495357,318495359,318494842&appid=com.google.android.gms

<<< HTTP 204, 0.00B

POST https://tracking.intl.miui.com/track/v4

Headers

```
OT_SID: 1904b9013...536c63d4
OT_ts: 1627024060966
OT_net: WIFI
OT_sender: com.miui.analytics
```

The payload is AES encrypted, but when decrypted it contains details of the user interaction with the Settings app, including which pages were viewed and the page open/close times, e.g.

```
{
  "event": 1,
  "pkg": "com.miui.securitycenter",
  "class": "com.miui.permcenter.settings.PrivacySettingsActivity",
  "ts": 1627023464771,
  "vn": "5.2.2-210430.1.2",
  "stat": "app_start"
},
{
  "event": 2,
  "pkg": "com.miui.securitycenter",
  "class": "com.miui.permcenter.settings.PrivacySettingsActivity",
  "ts": 1627023479140,
  "vn": "5.2.2-210430.1.2",
  "duration": 14369,
  "stat": "app_end",
  "app_duration": 14369
},
{
  "event": 1,
  "pkg": "com.miui.securitycenter",
  "class": "com.miui.permcenter.settings.PrivacySettingsActivity",
```

```
"ts": 1627023483435,
"vn": "5.2.2-210430.1.2",
"stat": "app_start"
},
{
  "event": 2,
  "pkg": "com.miui.securitycenter",
  "class": "com.miui.permcenter.settings.PrivacySettingsActivity",
  "ts": 1627023484763,
  "vn": "5.2.2-210430.1.2",
  "duration": 1328,
  "stat": "app_end",
  "app_duration": 1328
}
```

Other entries include: com.android.settings.MiuiSettings, com.android.settings.SubSettings, com.miui.system.LicenseActivity, com.xiaomi.market.ui.JoinActivity, com.xiaomi.market.ui.UpdateAppsActivityInner, com.android.phone.settings.MobileNetworkSettings, miui.notification.management.activity.NotificationAppListActivity, com.miui.home.settings.MiuiHomeSettingActivity, com.miui.miwallpaper.activity.WallpaperSettingActivity, com.android.thememanager.activity.ThemeSettingsActivity, com.android.packageinstaller.permission.ui.ManagePermissionsActivity, com.miui.powercenter.PowerMainActivity, com.google.android.apps.wellbeing.settings.SettingsActivity, com.xiaomi.account.ui.LoginActivity, com.google.android.gms.app.settings.GoogleSettingsActivity, com.google.android.gms.accountsettings.mg.ui.main.MainActivity, com.android.settings.Settings\$PrivacyDashboardActivity, com.google.android.gms.usagereporting.settings.UsageReportingActivity, com.google.android.gms.adsidentity.settings.AdsIdentitySettingsActivity.

D. Connections When Making/Receiving A Phone Call

POST https://tracking.intl.miui.com/track/v4

Headers

```
OT_SID: 1904b901...536c63d4
OT_sender: com.miui.analytics
```

<...>

```
{
  "event": 1,
  "pkg": "com.google.android.dialer",
  "class": "com.android.dialer.main.impl.MainActivity",
  "ts": 1627027673117,
  "vn": "67.0.383690429",
  "stat": "app_start"
},
{
  "event": 2,
  "pkg": "com.google.android.dialer",
  "class": "com.google.android.play.core.common.
PlayCoreDialogWrapperActivity",
  "ts": 1627027705602,
  "vn": "67.0.383690429",
  "duration": 53,
  "stat": "app_end",
  "app_duration": 32435
},
{
  "event": 1,
  "pkg": "com.android.vending",
  "class": "com.google.android.finsky.inappreviewdialog.
InAppReviewActivity",
  "ts": 1627027705627,
  "vn": "26.2.22-21 [0] [PR] 385030365",
  "stat": "app_start"
},
{
  "event": 2,
  "pkg": "com.android.vending",
  "class": "com.google.android.finsky.inappreviewdialog.
InAppReviewActivity",
  "ts": 1627027705694,
  "vn": "26.2.22-21 [0] [PR] 385030365",
  "duration": 67,
```

```

    "stat": "app_end",
    "app_duration": 67
  },
  {
    "event": 1,
    "pkg": "com.google.android.dialer",
    "class": "com.google.android.play.core.common.
PlayCoreDialogWrapperActivity",
    "ts": 1627027705716,
    "vn": "67.0.383690429",
    "stat": "app_start"
  },
  {
    "event": 2,
    "pkg": "com.google.android.dialer",
    "class": "com.android.dialer.main.impl.MainActivity",
    "ts": 1627027707839,
    "vn": "67.0.383690429",
    "duration": 2096,
    "stat": "app_end",
    "app_duration": 2121
  }
]

```

Xiaomi telemetry logs the user interaction with the dialer app when making a phone call, including the start and end times of the call.

POST <https://tracking.intl.miui.com/track/v4>

Headers

```

OT_SID: 1904b90...536c63d4
OT_ts: 1627029461128
OT_net: WIFI
OT_sender: com.miui.analytics
"seq": [
  {
    "event": 1,
    "pkg": "com.google.android.dialer",
    "class": "com.android.incallui.InCallActivity",
    "ts": 1627028918422,
    "vn": "67.0.383690429",
    "stat": "app_start"
  },
  {
    "event": 2,
    "pkg": "com.google.android.dialer",
    "class": "com.android.incallui.InCallActivity",
    "ts": 1627028934973,
    "vn": "67.0.383690429",
    "duration": 16551,
    "stat": "app_end",
    "app_duration": 16551
  }
]

```

Xiaomi telemetry logs the user interaction with the dialer app when receiving a phone call, including the start and end times of the call.

POST <https://dialercallinfolookup-pa.googleapis.com/google.internal.dialer.v1.DialerCallInfoLookupService/GetCallInfo>

Headers

```

user-agent: com.google.android.dialer/7302227 (Linux; U; Android 10;
en_GB; M2003J15SC; Build/QP1A.190711.020; Cronet/85.0.4183.127) grpc
-java-cronet/1.37.0-SNAPSHOT
content-type: application/grpc
x-goog-spatula: CjkKGWNv...B0jdv
authorization: Bearer ya29.m.Cvw...gIIAQ
+35387...3510 // phone number
<<< HTTP 200, 19.00B

```

The phone number is posted to google when making a phone call. An similar connection is seen when receiving a phone call. The response is the identity associated with the phone number, in this case "Doug Leith".

E. Connections When Sending A Text

POST <https://app-measurement.com/a>

When sending a text the messaging app com.google.android.apps.messaging logs user interactions using Google Analytics. The POST body is a protobuf that decodes, for example, to:

body {

```

always_one: 1
event {
  event_info {
    setting_code: "_o" // firebase_event_origin
    data_str: "auto"
  }
  event_code: "_ab" // app_background
  event_timestamp: 1620200906284
}
event {
  event_info {
    setting_code: "_o" // firebase_event_origin
    data_str: "auto"
  }
  event_info {
    setting_code: "_et" // engagement_time_msec
    data_int: 22542
  }
  event_info {
    setting_code: "_sc" // firebase_screen_class
    data_str: "HomeActivity"
  }
  event_info {
    setting_code: "_si" // firebase_screen_id
    data_int: 8474595296904148254
  }
  event_info {
    setting_code: "_fr"
    data_int: 1
  }
  event_code: "_e" // user_engagement
  event_timestamp: 1620200950238
  previous_event_timestamp: 1620200906281
}
event {
  event_info {
    setting_code: "_o" // firebase_event_origin
    data_str: "auto"
  }
  event_info {
    setting_code: "_pc" // firebase_previous_class
    data_str: "HomeActivity"
  }
  event_info {
    setting_code: "_pi" // firebase_previous_id
    data_int: 8474595296904148254
  }
  event_info {
    setting_code: "_sc" // firebase_screen_class
    data_str: "ConversationActivity"
  }
  event_info {
    setting_code: "_si" // firebase_screen_id
    data_int: 8474595296904148256
  }
  event_info {
    setting_code: "_et" // engagement_time_msec
    data_int: 22542
  }
  event_code: "_vs" // screen_view
  event_timestamp: 1620200950513
  previous_event_timestamp: 1620200896833
}
event {
  event_info {
    setting_code: "_o" // firebase_event_origin
    data_str: "app"
  }
  event_info {
    setting_code: "_c" // firebase_conversion
    data_int: 1
  }
  event_info {
    setting_code: "_r" // realtime
    data_int: 1
  }
  event_code: "ACTIVE_EVENT"
}

```

```

    event_timestamp: 1620201017976
  }
  event {
    event_info {
      setting_code: "_o" // firebase_event_origin
      data_str: "auto"
    }
    event_info {
      setting_code: "_et" // engagement_time_msec
      data_int: 67468
    }
    event_info {
      setting_code: "_sc" // firebase_screen_class
      data_str: "ConversationActivity"
    }
    event_info {
      setting_code: "_si" // firebase_screen_id
      data_int: 8474595296904148256
    }
    event_code: "_e" // user_engagement
    event_timestamp: 1620201017982
    previous_event_timestamp: 1620200950238
  }
  event {
    event_info {
      setting_code: "_o" // firebase_event_origin
      data_str: "auto"
    }
    event_code: "_ab" // app_background
    event_timestamp: 1620201018000
    previous_event_timestamp: 1620200906284
  }
  user {
    timestamp: 1619603483322
    setting: "_fot"
    data_int: 1619604000000
  }
  user {
    timestamp: 1619603483322
    setting: "_fi" // first_install
    data_int: 1
  }
  user {
    timestamp: 1620200895446
    setting: "_sno" // session_number
    data_int: 1
  }
  user {
    timestamp: 1620200895446
    setting: "_sid" // session_id
    data_int: 1620200895
  }
  user {
    timestamp: 1620201028459
    setting: "_lte" // lifetime_engagement
    data_int: 100517
  }
  user {
    timestamp: 1620201028462
    setting: "_se" // session_scoped_engagement
    data_int: 100516
  }
  message_timestamp: 1620201028443
  event_timestamp: 1620200906284
  bundle_end_timestamp: 1620201018000
  last_bundle_end_timestamp: 1620200906281
  operating_system: "android"
  operating_system_version: "10"
  Build_MODEL: "M2003J15SC"
  language_country: "en-gb"
  timezone_offset_mins: 60
  package_name: "com.google.android.apps.messaging"
  app_version: "7.8.064 (Oak_RC01.phone_dynamic)"
  gmp_version: 40007
  gms_version: 211213
  google_ad_id: "a4f87f3f-4661-4334-a8d8-a4e207eb3b7c"
  random_hex: "b08442668ca3816d7e12430efa978599"

```

```

dev_cert_hash: 7599436411597265477
daily_conversions_count: 4
gmp_app_id: "1:357317899610:android:4765c0ded882c665"
last_bundle_end_timestamp2: 1620200895446
always_true: true
firebase_instance_id: "dYVOPRv2bbHUi4LKF1Z8s"
app_version_int: 78064063
config_version: 1591635597861345
M: 23180934
M: 23180923
M: 23180912
dynamite_version: 49
unknown: "G1—"
}

```

commentIt can be seen that user interactions with the HomeActivity and ConversationActivity are logged. An ACTIVE_EVENT event is also logged that appears to correspond to sending a text.

swiftkey

III. HUAWEI

Summary:

<i>Huawei system app endpoints:</i><br>query.hicloud.com	<i>Identifiers Sent:</i><br>hardware serial number, device cert, uidid
configserverdre.platform.hicloud.com	hardware serial number
servicesupport.hicloud.com	userId
shepherd.sb.avast.com,<br>apkrep.ff.avast.com	hash of android_id, ABUID
mvconf.cloud.360safe.com,<br>mclean.cloud.360safe.com	uuid
pebed.dmevent.net, dmxleo,<br>dailymotion.com	GAID
<i>Third-party (non-Google) system app endpoints:</i><br>in.appcenter.ms	<i>Identifiers Sent:</i><br>installId
telemetry.api.swiftkey.com	installId, GAID
Identifiers observed to persist across factory reset	hardware serial number, device cert

TABLE V

SUMMARY OF IDENTIFIERS SENT IN SYSTEM APP CONNECTIONS (EXCLUDING GOOGLE SYSTEM APPS).

Telemetry	
telemetry.api.swiftkey.com	When the keyboard is used within an app, the app name, number of characters entered and an event timestamp are sent e.g. use of searchbar, contacts and messaging apps is logged. Interactions with the keyboard, e.g. opening the clipboard, viewing/modifying the settings, are also logged.
in.appcenter.ms	Logs Swiftkey app crashes, including stack traces.
pebed.dmevent.net	Logs events and settings associated with com.huawei.himovie.overseas app e.g. event initStart.
apkrep.ff.avast.com	Logs app details when a new app is installed.
Device Data	
query.hicloud.com	device details
configserverdre.platform.hicloud.com	device details
servicesupport.hicloud.com	device details
shepherd.sb.avast.com,<br>apkrep.ff.avast.com	device details
mvconf.cloud.360safe.com/<br>safeupdate	device details, installed apps
in.appcenter.ms, bibo.api.swiftkey.com,<br>telemetry.api.swiftkey.com	device details
pebed.dmevent.net	device details
<i>Notes:</i><br>The messaging app on the handset is com.google.android.apps.messaging. This Google app uses Google Analytics to log user interaction, including screens/activities viewed plus duration and timestamp, and logs the event that text is sent.	

TABLE VI

SUMMARY OF DATA SENT IN SYSTEM APP CONNECTIONS (EXCLUDING GOOGLE SYSTEM APPS).

- 1) The handset hardware serial number is sent to query.hicloud.com and configserverdre.platform.hicloud.com (domains that appear to be registered to Huawei). A device certificate is also sent to query.hicloud.com that contains a CN value that appears to be a device identifier.

When a SIM is inserted the mobile carrier id is sent to query.hicloud.com.

- 2) Device details are sent to servicesupport.hicloud.com, together with a userId value .
- 3) Connections made by com.huawei.himovie.overseas:
  - a) *Daily Motion*. The app makes connections to www.dailymotion.com that send the device Google adid/r-did advertising id as a parameter in the URL. The response sets several cookies plus an HTML document. The HTML contains embedded device/user identifiers, including the Google adid/r-did and client_id, client_secret values.
  - b) Following the connection to www.dailymotion.com, connections are made to a sequence of other third-party content servers, all with a referrer header value set to the original request to www.dswiftkeyailymotion.com, so presumably these are prompted by processing of the HTML document sent by www.dailymotion.com. Since the handset Google adid/r-did is embedded in the URL, this is shared with all of these third-party content servers, including static1.dmcnd.net, imasdk.googleapis.com, s0.2mdn.net, url-pagead2.googleadsyndication.com.
  - c) Connections are also made to pebed.dm-event.net, a domain registered to Daily Motion. These connections send the handset Google adid/r-did in the referrer header, but also device details and instance_uuid, id values in the POST body that appear to be device/user identifiers, including one of the cookie values set in the response to the request to www.dailymotion.com. This connection appears to send analytics/telemetry.
  - d) Connections are made speedtest.dailymotion.com. These connections send the handset Google adid/r-did in the referrer header, as well as the cookie set by the response to the request to www.dailymotion.com. Similarly for connections to dmxleo.dailymotion.com.
- 4) Connections made by com.huawei.systemmanager:
  - a) *Avast App Scanning Service*. The com.huawei.systemmanager app, which has the package com.avast.android.sdk embedded within it, makes connections to auth.ff.avast.com, shepherd.sb.avast.com, apkrep.ff.avast.com, analytics.ff.avast.com/receive3. This appears to be associated with an apk scanning service (e.g. connections are reliably generated whenever an apk is installed). The data sent in these connections are AES encrypted protobufs. When decrypted and decoded as a protobuf, periodic authentication connections to shepherd.sb.avast.com/V1/MD send a hash of the device android_id and an ABUID value that also acts as a persistent identifier. When decrypted and decoded as a protobuf, connections to apkrep.ff.avast.com/apk/reputation and apkrep.ff.avast.com/apk/touch send app details (filename, signing cert). The payload also appears to contain persistent device identifiers, and

the encryption scheme used also allows messages from the same device/session to be linked together.

- b) *Qihoo 360*. The `com.huawei.systemmanager` app also has the packages `com.qihoo.cleandroid.sdk`, `com.qihoo.cleandroid.cleanwx.sdk`, `urlcom.qihoo.cleandroid.mobilesmart.sdk`, `com.qihoo.qvssdk`, `com.qihoo.security.engine`, `com.qihoo.protection` embedded within it. The app makes connections to `mvconf.cloud.360safe.com/safeupdate` and `mclean.cloud.360safe.com/CleanQuery` periodically (every 1-2 days), and a connection to `aiclean.us.cloud.360safe.com/video/clean` is observed once. The messages are encrypted using a JNI C library and are encoded in a custom binary format. When decrypted it can be seen that the connections contain a persistent device identifier (labelled “imei” but the value is generated randomly when the app is first started after a factory reset).
- 5) When sending a text, the messaging app `com.google.android.apps.messaging` uses Google Analytics to log user interaction, including screens/activities viewed plus duration and timestamp.

Pre-installed Non-Huawei System apps

1) Microsoft

- a) The handset uses Microsoft’s `com.touchtype.swiftkey` keyboard. This sends data to `in.appcenter.ms/logs`, `bibo.api.swiftkey.com` with a persistent install-ID and device details. The `in.appcenter.ms/logs` connection appears to relate to logging of app errors and sometimes includes stack traces.
- b) Telemetry data is sent to `telemetry.api.swiftkey.com` which includes device details and logs events. Data is encoded in gzipped avro format. To decode the avro data the schema was extracted from the app by using `edXposed` to execute a `getSchema()` call on app startup and dumping the (large, about 200KB) response to disk. After decoding using this scheme it can be seen that the data sent includes the Google `adid/rdid` Advertising id, a Firebase `id/token` and an `installId` value. User interaction with apps is logged when the keyboard is used within the app, with the app name, number of characters entered and a millisecond accuracy event timestamp sent to `telemetry.api.swiftkey.com`. Such event logging was observed, for example, when using the contacts, messaging, searchbar and notepad apps. Interactions with the keyboard, e.g. opening the clipboard, viewing/modifying the settings, are also logged.

2) Google. The following pre-installed Google system apps were observed to send data to Google.

- a) Google Play Services and Google Play store make many connections to Google servers. These share persistent device and user identifiers with Google including the device hardware serial number, SIM IMEI, Wifi MAC address, SIM IMSI and phone number,

user email (when logged in). A substantial quantity of data is sent, in particular, to `play.googleapis.com/vn/log/batch`, `play.googleapis.com/play/log` and `www.googleapis.com/experimentsandconfigs`. This is consistent with other recent measurement studies, see “Mobile Handset Privacy: Measuring The Data iOS and Android Send to Apple And Google”, Proc SECURE-COM 2021.

- b) Google Youtube sends device data, including persistent identifiers and the Google `adid/rdid` advertising identifier and the `AndroidID`, to `www.googleadservices.com` and `youtubei.googleapis.com`. Youtube also uses Google Analytics to log events, and presumably also user interaction.
- c) Connections are periodically made to `www.google.com/complete/search`. These are associated with the `com.google.android.googlequicksearchbox` searchbar app embedded in the handset UI and send a cookie which acts to link these connections to persistent device and user identifiers. Less frequent connections are made to `www.google.com/m/voice-search/down` that contain what appears to be a persistent device identifier. The `com.google.android.googlequicksearchbox` app also sends telemetry data to Google Analytics that logs user interaction (screens/activities viewed plus duration and timestamp, etc).
- d) The system messaging app `com.google.android.apps.messaging` uses Google Analytics to log user interaction, including screens/activities viewed plus duration and timestamp. For example, when sending a text the user interactions with the `WelcomeActivity`, `HomeActivity` and `ConversationActivity` are logged, and a `FIRST_MESSAGE_SENT` event is also recorded.
- e) The `com.google.android.apps.tachyon` logs events using Google Analytics.
- f) When logged in to a Google account, connections are made to `mail.google.com/mail/ads`, `inbox.google.com/sync` and `www.googleapis.com/calendar` that send identifiers linked to the device and user account. Note that account login was carried out via the Google Play app only. Syncing of gmail, contacts, calendar took place without the user being asked or opting in. When logged in to a Google account, connections are also made to `instantmessaging-pa.googleapis.com`, `people-pa.googleapis.com`, `footprints-pa.googleapis.com`. It’s not clear what the purpose of these connections is or what data is sent. In addition, the following google services are authenticated: `googleplay`, `android_video`, `drive.metadata.readonly`, `drive.labels.readonly`, `drive.activity.readonly`, `docs`, `drive.readonly`, `peopleapi.readonly`, `drive.apps`, `cloudprint`, `activity`, `drive.file`, `gmail.readonly`, `subscriptions`, `memento`, `notifications`, `spreadsheets`, `vouchers`, `discussions`, `userlocation.reporting`, `peopleapi.legacy.readwrite`, `reminders`, `calendar`, `play-`

atoms, mobileapps.doritos.cookie, peopleapi.readwrite, OAuthLogin, sierra, webhistory, gmail.full_access, gmail.ads, gmail.locker.read, taskassist.readonly, gmail.publisher_first_party, experimentsandconfigs, tachyon, numberer, firebase.messaging, userinfo.email, gcm, android_checkin, login_manager, cryptauth, notifications.

- g) When location is enabled additional connections are made to www.googleapis.com/geolocation/v1/geolocate and mobilenetworkscoring-pa.googleapis.com/v1/GetWifiQuality. The connection to mobilenetworkscoring-pa.googleapis.com/v1/GetWifiQuality sends encoded data and it's not clear what the contents are but they include the mobile operator MNC and MCC identifiers.
- h) Google Chrome makes connections to Google servers. These connections are consistent with previously documented behaviour, see "Web Browser Privacy: What Do Browsers Say When They Phone Home?", IEEE Access. DOI 10.1109/ACCESS.2021.3065243.
- i) When a SIM is inserted into the handset SIM details are sent to Google as observed in previous studies, see "Mobile Handset Privacy: Measuring The Data iOS and Android Send to Apple And Google", Proc Securecomm 2021.

Note that none of these apps were opened on the device, and no popup or request to send data was observed.

A. Selected Connections During Startup After Factory Reset

POST [https://servicesupport.hicloud.com/servicesupport/theme/getSupportOnlineInfo.do?firmware=9&locale=English&version=2.3&buildNumber=MAR-L21B9.1.0.372\(C431E2R4P1\)&phoneType=MAR-LX1B&isoCode=&ver=1.5](https://servicesupport.hicloud.com/servicesupport/theme/getSupportOnlineInfo.do?firmware=9&locale=English&version=2.3&buildNumber=MAR-L21B9.1.0.372(C431E2R4P1)&phoneType=MAR-LX1B&isoCode=&ver=1.5)

Headers

```
businessType: 2
apkVersion: 90100017
buildNumber: MAR-L21B9.1.0.372(C431E2R4P1)
```

&versionCode=420000

POST https://query.hicloud.com/sp_ard_common/v2/onestopCheck.action?verType=true&autoInstall=y&latest=true&logOnly=true
{ "commonRules": { "Fingerprint": "HUAWEI/MAR-LX1BEEA/HWMAR:9/HUAWEIMAR-L21B/9.1.0.372C431:user/release-keys", "DeviceName": "MAR-L21B", "VendorCountry": "hw-eea", "BoardID": "7928", "Reserved1": "", "Reserved2": "", "vendor": "MAR-L21B-hw-eea", "deviceId": "FUNDU20924007430", "udid": "0C3B05F3379E4EDB40D5...08F4A39E09C0B", "Language": "en-ie", "OS": "Android 9", "HotaVersion": "9.1.30.391", "saleinfo": "black|eea|hw|N|EmotionUI_9.1.0|6.4 GB|256 GB|8_2.2GHz|Y|blue|9.1.30.391|Y", "C_version": "C431", "D_version": "D000", "Subgroup": "", "Vergroup": "", "devicetoken": "", "PackageType": "psi", "ControlFlag": "0", "extra_info": "42932F1...9348DDCFB2B", "DeviceDisplayVersion": "MAR-L21B 9.1.0.372(C431E2R4P1)", "ProductBaseVersion": "MAR-LGRP2-OVS 9.1.0.372", "Emui": "EmotionUI_9.1.0", "versionPackageRules": [{ "versionPackageType": 2, "rules": { "FirmWare": "MAR-LGRP2-OVS 9.1.0.372" } }, { "versionPackageType": 3, "rules": { "FirmWare": "MAR-L21B-CUST 9.1.0.2(C431)" } }, { "versionPackageType": 4, "rules": { "FirmWare": "MAR-L21B-PRELOAD 9.1.0.1(C431R4)" } }], "keyAttestation": "r00AB...dAAFWC41MDk=" }
"versionPackageRules": [

```
{
  "rules": {
    "FirmWare": "MAR-LGRP2-OVS 9.1.0.372"
  }
},
```

```
    "versionPackageType": 2
  },
  {
    "rules": {
      "FirmWare": "MAR-L21B-CUST 9.1.0.2(C431)"
    },
    "versionPackageType": 3
  },
  {
    "rules": {
      "FirmWare": "MAR-L21B-PRELOAD 9.1.0.1(C431R4)"
    },
    "versionPackageType": 4
  }
]
```

<<< HTTP 200, 159.00B

The deviceId value is the handset hardware serial number ro.boot.serialno. The udid value is a device identifier obtained by calling the getUDID() methods of the device_identifiers service running on the handset. The extra_info consists of three sections, separated by "——". The first section is AES encrypted by a custom obfuscated JNI library libencrypt_data_jni.so, the second section AES encrypted in Java and the third section is the RSA encrypted AES key (presumably the server possesses the private key to decrypt this. The extra_info value decrypts to:

"extra_info": "FUNDU20924007430|||87404|0|0000...000010000000700000000...000060000010000050000000404010004020101010000...0002020004020100...00|FUNDU20924007430|27211|||5379766007768122"

The FUNDU20924007430 value is the device hardware serial number, the 27211 value the mobile carrier id, the 5379766007768122 is the AES key. This is followed by several similar connections.

POST <https://android.clients.google.com/c2dm/register3>

Headers

```
Authorization: AidLogin 4454149769076196282:1724068696247828183
The AidLogin value include the Google AndroidID.
```

POST <https://app-measurement.com/a>

Connections to Google Analytics by app com.google.android.apps.maps. Similar connections also made by apps om.google.android.apps.messaging, com.google.android.apps.work.oobconfig, com.google.android.gms, com.android.vending, com.google.android.youtube, com.google.android.videos, com.google.android.apps.tachyon, com.google.android.googlequicksearchbox

GET https://grs.hicloud.com/grs/1.0/hwouc/router?ser_country=DE&cp=2021-04-22T08%3A59%3A42.440%7C79

Headers

```
user-agent: hwouc
ser_country: DE
cp: 2021-04-22T08:59:42.440|79
```

POST [https://servicesupport.hicloud.com/servicesupport/theme/getSupportOnlineInfo.do?firmware=9&locale=English&version=2.3&buildNumber=MAR-L21B9.1.0.372\(C431E2R4P1\)&phoneType=MAR-LX1B&isoCode=&ver=1.5](https://servicesupport.hicloud.com/servicesupport/theme/getSupportOnlineInfo.do?firmware=9&locale=English&version=2.3&buildNumber=MAR-L21B9.1.0.372(C431E2R4P1)&phoneType=MAR-LX1B&isoCode=&ver=1.5)

Headers

```
businessType: 2
apkVersion: 90100017
buildNumber: MAR-L21B9.1.0.372(C431E2R4P1)
&versionCode=420000
```

POST https://query.hicloud.com/sp_ard_common/v2/onestopCheck.action?verType=true&autoInstall=y&latest=true
{ "commonRules": { "Fingerprint": "HUAWEI/MAR-LX1BEEA/HWMAR:9/HUAWEIMAR-L21B/9.1.0.372C431:user/release-keys", "DeviceName": "MAR-L21B", "BoardID": "7928", "VendorCountry": "hw-eea", "Reserved1": "", "Reserved2": "", "deviceId": "FUNDU20924007430", "udid": "0C3B05F3379E4EDB40D51C5...08F4A39E09C0B", "Language": "en-ie", "OS": "Android 9", "HotaVersion": "9.1.30.391", "C_version": "C431", "D_version": "D000", "DeviceDisplayVersion": "MAR-L21B 9.1.0.372(C431E2R4P1)", "ProductBaseVersion": "MAR-LGRP2-OVS 9.1.0.372", "Emui": "EmotionUI_9.1.0", "vendorCota": "", "vendorExpiredTime": "0", "ControlFlag": "0", "PackageType": "full", "Type": "ATL", "versionPackageRules": [{ "versionPackageType": 5, "rules": { "FirmWare": "Cota000" } }], "keyAttestation": "r00AB...BVguNTA5", "deviceCertificate": "

r00ABXNyAC1qYX...C41MDk="}

Followed by several similar connections. The deviceCertificate value base64 decodes to an SSL cert, signed by Huawei. The CN field in this cert is HUAWEI_HWMAR_d68b6418-8fdb-478f-8a8e-32bad4d4ea9, which appears to be a device identifier.

GET https://configserver-dre.platform.hicloud.com/servicesupport/updateserver/data/COTA?ParamId=NONCELL&ParamVersion=1.10.18.101&CompatibleVersion=1&SubType=EMUI9&DeviceName=MAR-LX1B&EMUI=9.1.0&Vendorcountry=hw_eea&BaseVersion=MAR-LGRP2-OVS%209.1.0.372

Headers

Device-ID: **FUNDU20924007430**
App-ID: hwouc

POST https://servicesupport1.hicloud.com/servicesupport/theme/login-client.do
'userId=726f7612-c0b4-4bbc-94f1-c067a4b1e10e&firmware=9&locale=en&screen=2312*1080&realscreen=2312*1080&density=3.0&version=9.1.0.017&buildNumber=MAR-L21B9.1.0.372(C431E2R4P1)&phoneType=MAR-LX1B&mcc=460&mnc=00&versionCode=90100017&ver=1.6&type=2'

<<< HTTP 200, 927.00B

POST https://servicesupport.hicloud.com/servicesupport/theme/getThemeMagazine.do
sign=064B10065211NU...7AF6A7&language=English&themenam=Balance%28magazine%29&author=Huawei+Emotion+UI&version=2.3&screen=2312*1080&phoneType=MAR-LX1B&buildNumber=MAR-L21B9.1.0.372(C431E2R4P1)&isoCode=&ver=1.0&romversion=9.1.0&versionCode=420000

GET https://theme.dbankcdn.com/magazine/2199/01/2_20201217113334_01_114588345899111.zip

Headers

User-Agent: AndroidDownloadManager/9 (Linux; U; Android 9; MAR-LX1B Build/HUAWEIMAR-L21B)
<<< HTTP 200, 2.95MB

POST https://android.googleapis.com/checkin
Sends, and links together, many device identifiers. Followed by many other connections to Google.

GET https://configdownload-dre.dbankcdn.com/nsp-configserver-p01-dre/update/44eda3f92c1cbb11133bce4cd0e52de99c5972b3bb9d3178df232cf6aab7579d/filelist.xml
<<< HTTP 200, 366.00B

B. Connections When the phone is idle

GET https://grs.dbankcloud.com/grs/1.0/hicloud/router?reg_country=SG&country_source=APP
Headers
User-Agent: com.huawei.hidisk/10.6.0.310 (Linux; Android 9; MAR-LX1B) NetworkKit-grs/1.0.9.301
<<< HTTP 200, 3.98KB

POST https://query.hicloud.com/sp_ard_common/v2/onestopCheck.action?verType=true&autoInstall=y&latest=true
{ "commonRules": { "Fingerprint": "HUAWEI/MAR-LX1BEEA/HWMAR:9/HUAWEIMAR-L21B/9.1.0.372C431:user/release-keys", "DeviceName": "MAR-L21B", "VendorCountry": "hw-eu", "BoardID": "7928", "Reserved1": "", "Reserved2": "", "vendor": "MAR-L21B-hw-eea", "deviceId": "FUNDU20924007430", "udid": "0C3B05F3379E4EDB40D5...AF08F4A39E09C0B", "Language": "en-ie", "OS": "Android 9", "HotaVersion": "9.1.30.391", "saleinfo": "black|eea|hw|N|EmotionUI_9.1.0|6.4 GB|256 GB|8_2.2GHz|Y|blue|9.1.30.391|Y", "C_version": "C431", "D_version": "D000", "Subgroup": "", "Vergroup": "", "devicetoken": "", "PackageType": "patch", "ControlFlag": "0", "extra_info": "041B4996...1116", "DeviceDisplayVersion": "MAR-L21B 9.1.0.372(C431E2R4P1)", "ProductBaseVersion": "MAR-LGRP2-OVS 9.1.0.372", "Emui": "EmotionUI_9.1.0", "versionPackageRules": { "versionPackageType": "2", "rules": { "FirmWare": "MAR-LGRP2-OVS 9.1.0.372(patch00)" } } }, "keyAttestation": "r00ABXN...BVguNTA5", "deviceCertificate": "r00ABXNyAC1qYXZhLnNlY3VyaXR5LmNlc...C41MDk=" }

"versionPackageRules": [{ "rules": { "FirmWare": "Cota000" }, "versionPackageType": 5 }]

<<< HTTP 200, 83.00B

Followed by several similar connections

GET https://grs.hicloud.com/grs/1.0/systemmanager/router?ser_country=DE
Headers
user-agent: systemmanager
<<< HTTP 200, 317.00B

POST https://servicesupport.hicloud.com/servicesupport/theme/getSupportOnlineInfo.do?firmware=9&locale=English&version=2.3&buildNumber=MAR-L21B9.1.0.372(C431E2R4P1)&phoneType=MAR-LX1B&isoCode=&ver=1.5
Headers
businessType: 2
apkVersion: 90100017
buildNumber: MAR-L21B9.1.0.372(C431E2R4P1)
&versionCode=420000
<<< HTTP 200, 48.00B

POST https://servicesupport.hicloud.com/servicesupport/theme/getThemeMagazine.do
Headers
businessType: 2
apkVersion: 90100017
buildNumber: MAR-L21B9.1.0.372(C431E2R4P1)
sign=064B10065...7AF6A7&language=English&themenam=Balance%28magazine%29&author=Huawei+Emotion+UI&version=2.3&screen=2312*1080&phoneType=MAR-LX1B&buildNumber=MAR-L21B9.1.0.372(C431E2R4P1)&isoCode=&ver=1.0&romversion=9.1.0&versionCode=420000
<<< HTTP 200, 913.00B

POST https://store.hispace.hicloud.com/hwmarket/api/clientApi
User-Agent: com.huawei.hwid3.0.3.300_HUAWEI
buildNumber=MAR-L21B%209.1.0.372%28C431E2R4P1%29&clientPackage=com.huawei.hwid&cno=4010002&code=0500&density=480&emuiApiLevel=19&emuiVer=&firmwareVersion=9&gmsSupport=0&isSubUser=0&isUpdateSdk=1&locale=en_IE&mapleVer=0&method=client.https.front&net=1&packageName=com.huawei.hwid&phoneType=MAR-LX1B&resolution=1080_2107&screen=1080_2107&serviceType=0&supportMaple=0&sysBits=2&theme=true&thirdId=updatesdk_com.huawei.hwid&ts=1628056949995&ver=1.1&version=3.0.3&versionCode=30003300&zone=1

POST https://youtubei.googleapis.com/deviceregistration/v1/devices?key=AIzaSyA8...yYM39w&rawDeviceId=6c09529dca6348b
<<< HTTP 200, 233.00B

GET https://www.googleadservices.com/pagead/conversion/1001680686/?bundleid=com.google.android.youtube&appversion=14.47.50&osversion=9&sdkversion=ct-sdk-a-v2.2.4&gms=1&lat=0&rdid=228b814c-14fe-4da3-b2a8-cdf2382a02ba&timestamp=1619083875.177&remarketing_only=1&usage_tracking_enabled=0&data.screen_name=%3CAndroid_YT_Open_App%3E
<<< HTTP 200, 0.00B
Followed by several similar connections.

POST https://youtubei.googleapis.com/youtubei/v1/visitor_id?key=AIzaSyA...yYM39w
Headers
x-goog-device-auth: device_id=AP+lc793oNp1...KDG0VxNX0g
user-agent: com.google.android.youtube/14.47.50(Linux; U; Android 9; en_IE; MAR-LX1B Build/HUAWEIMAR-L21B) gzip
POST body decoded as protobuf:
<device details>
1 {
1: "ms"
2: "CoACsQy...hUj8Ew"

<...>

Followed by several similar connections.

POST https://auth.ff.avast.com/V1/REG

POST body decoded as protobuf:

```
1 {
  1: ""g|\3...3j}m"
}
2: ""
```

Response decoded as protobuf:

```
1 {
  1: "2cfb498c\000\000\001x\321\325\261("
}
2: ""000\360\376HR\010\366\250\000\001y\"#\311\0322cfb498c\000\000\001x\322\|n\273"
3: "2\241\336\276r\004P\005\326\367j\022\273\037\335\334\300\253\303vt\3222B\002\3077\240\362E\352"
4: 1619689041178
```

The first connection with avast.com, apparently registering with what seems to be an apk scanning service. Presumably the POST body data sent is an API key/authentication.

POST http://shepherd.sb.avast.com/V1/MD/

00F29FB75208F6...00017AA4717298/1627974747380

\xbc\xdl\x8i...x1a'\xba0\x88

The POST body is encrypted, the first bytes are the IV (randomised), the last 20 bytes are a HMAC checksum and the intervening bytes are an AES encrypted protobuf. An example of the decrypted content decoded as a protobuf is:

```
1 {
  1: "5\262\213\177\240j\3549"
}
2: ""
4: 0
5: 6
6: 1627974747380
7 {
  1: 13
  3 {
    1: "IE"
    2: "Tesco Mobile"
    3 {
      1: "9"
      2: "HUAWEIMAR-L21B"
      3: "MAR-LX1B"
      4: "HUAWEI"
      5: "HUAWEI"
      6: 28
    }
    5: "0"
    6: "id_not_available"
    7: "725D6E40BFFAC3C147E0983ECDB8ABC8C115D3D7"
    10: "56275433477153784753" // ABUID
  }
  15: 1
  16 {
    1 {
      1: "a4e0379764c...9be65206534a" // API key
      2: "com.huawei.systemmanager"
      3: "\212\360\366M\371 \266\004$\331\002\031\230\rKI"
    }
  }
}
```

The 725D6E40BFFAC3C147E0983ECDB8ABC8C115D3D7 value is an SHA1 hash of the secure settings android_id value d0729a3b1ca6232a. The 56275433477153784753 ABUID value appears to be a random number generated on first startup, so acting as a persistent identifier. The highlighted string 00F29FB75208F6...00017AA4717298 in the URL appears to act as a session identifier (likely it is an identity token returned by an earlier call to auth.ff.avast.com/V1/REG).

POST http://apkrep.ff.avast.com/apk/reputation

Headers

X-AVAST-SeqNum: 1619084238760

X-AVAST-KeyId: 00F0FE485208...78D27C0ABB

POST body:

\xf7\xbb\xe9{\x7f\xaf\xe4\xbe1\x10\x...\x0c\xf9\x9c\x1d

POST http://apkrep.ff.avast.com/apk/touch

Headers

X-AVAST-SeqNum: 1619084242512

X-AVAST-KeyId: 00F0FE485208F6A...7C0ABB

POST body:

b"\xd9\xcb?\xef\xee\x7fiT(\...\x90\x13"

POST http://analytics.ff.avast.com/receive3

Headers

X-AVAST-SeqNum: 1619084244871

X-AVAST-KeyId: 00F0FE48520...300000178D27C0ABB

POST body:

b' \xc8\xf4\xf6...x98\xc5}'

The X-AVAST-KeyId header value acts as a session identifier (it is the same for several connections, but changes over time). The X-AVAST-SeqNum value is a timestamp. The POST bodies are AES encrypted. An example of the data sent to apkrep.ff.avast.com/apk/touch (generated after install of the com.covidtracker.hse app), decrypted and decoded as a protobuf, is:

```
1: 0
2 {
  1 {
    4: "\244\3407\227d\303yl\301\200d\336\273\272\233\346R\006SJ"
  }
  5: 3
}
3: "$\331\002\031\230\rKI\212\360\366M\371 \266\004"
5 {
  2: 4612940
  3: "/data/app/com.covidtracker.hse-SfJ-rhufe6NZ9pFHNJOCKw==/base.apk"
}
6 {
  1: "c\315E%\020\020{N\3346L\371M<\265\003\304xt"
  2: "CN=Android,OU=Android,O=Google Inc.,L=Mountain View,ST=California,C=US"
  3: "CN=Android,OU=Android,O=Google Inc.,L=Mountain View,ST=California,C=US"
  4: 1587138298000
  5: 2533823098000
  6: "539341327954379552131647825952406305660406727894"
  7: "SHA256withRSA"
  8: "META-INF/BNDLTOOL.RSA"
}
8: "\203Q\246k\003r\004\233\314\354\302\204\024\264\017PQ\227+Y\247o\035A\303T\321\365X@u\363"
}
```

It can be seen that the payload seems to consist of the apk filename and details from the cert used to sign the apk. Field 3 "£\331\002\03... \266\004" and field 4 "\244\3407... \346R\006SJ" stay the same when other apks are installed and so may act as device identifiers. An example of the data sent to apkrep.ff.avast.com/apk/reputation, decrypted and decoded as a protobuf, is:

```
1: 0
2 {
  2 {
    1: "c\315E%\020\020{N\3346L\371M<\265\003\304xt"
  }
  4: "\203Q\246k\003r\004\233\314\354\302\204\024\264\017PQ\227+Y\247o\035A\303T\321\365X@u\363"
}
6 {
  4: "\244\3407\227d\303yl\301\200d\336\273\272\233\346R\006SJ"
}
5: 2
}
3: 984
```

Field 4 "\244\3407... \346R\006SJ" stays the same when other apks are installed and so may act as a device identifier.

GET http://sp-8f237ea3.honzik.avcdn.net/defs/android-vps/release.xml.lzma

GET http://sp-8f237ea3.honzik.avcdn.net/diffs/8b333/bfb8/1808/8

b33fb81808d77b810ffeb345b62abbcefd5b72dc50df576f8a88da135d5735-bb0df2e6c2e8c5b55b7a514d858fce26a2c3ccb1572fa21a978b5695936a0206.bs43

GET http://sp-8f237ea3.honzik.avcdn.net/universe/bb0df2e6c2e8/

bb0df2e6c2e8c5b55b7a514d858fce26a2c3ccb1572fa21a978b5695936a0206.lzma

GET http://sp-8f237ea3.honzik.avcdn.net/diffs/f0ef/a50d/345b/f0efa50d345b0864454808920433d4a9f127d4626428e743ce090bb8a71738ed-ca8139b39efca9a0ad291c2955347c4133eb569ff53ffe69475d3db0d409a816.bs43

And similar connections. Domain *avcdn.net* is registered to Avast.

POST http://mvconf.cloud.360safe.com/safe_update
Headers

User-Agent: 360clearsdk

POST http://mclean.cloud.360safe.com/CleanQuery
Headers

User-Agent: 360clearsdk

These two connections are made periodically. The POST bodies are in a custom binary format with the payload encrypted using a JNI C library libmobilesafe360_clear-jni-6.7.so. An example of the decrypted mvconf.cloud.360safe.com/safe_update payload (extracted from the app memory before encryption) is:

```
sdkid=2
permission=com.qihoo.antivirus.update.permission.clear_sdk_default
uilang=936
pa=x86
mid=81d47510792a6f616daa03580517e215
cid=107415
product=clear_sdk_multilang
combo=mobile
sdk=28
updsene=2
updsetting=1
pkg=com.huawei.systemmanager
connect_type=1
ver=6.4.7.1005
date=2021.08.04 07:02:30
imei=3e8913e0-5b79-47b9-8fde-a6671bffb66d
free_disk=0
wifi=1
brand=HUAWEI
model=MAR-LX1B
free_disk_x=0
uv=1
```

The mid value 81d47510792a6f616daa03580517e215 is an MD5 hash of the imei value 3e8913e0-5b79-47b9-8fde-a6671bffb66d. This is a random UUID generated when the app is first started, i.e. after factory reset, and so acts as a persistent device identifier. An example of the decrypted payload sent to mclean.cloud.360safe.com/CleanQuery is:

```
\n\xf3\x01\x08\x0b\x10e\x1a\x10\x81\x4d\x10y*oam\xaa\x03X\x05
\x17\xe2\x15"$3e8913e0-5b79-47b9-8fde-a6671bffb66d(d0\x97\x7c\x06
:\n6.4.7.1005B\x06HUAWEIJ\x08MAR-LX1B\x0228Z\x019'\x00j
\x05en_WWr\x17cleandroid_huawei.cloudzX{"clean_get_commonpath":1,"
cid":"107415","cleansdk_user_pkg":"com.huawei.systemmanager"}\x82
\x01\x04c:1\xa2\x01\x06\x10\x00\x18\x00 \x00B\xea\xce\x01\n\x1e
\x12\x1c$360$com.android.calculator2\n\x1b\x12\x19$360$com.android.
calendar\n\x19\x12\x17$360$com.android.chrome\n\x1b\x12\x19$360$com.android.contacts\n\x1c\x12\x1a$360$com.android.deskclock
\n\x1e\x12\x1c$360$com.android.documentsui\n<details of installed apps
and media folders/files>
```

The plaintext payload is in a custom binary format but it can be seen that the header contains the device identifier value 3e8913e0-5b79-47b9-8fde-a6671bffb66d.

POST https://in.appcenter.ms/logs?api-version=1.0.0

Headers

Install-ID: cc4de3ad-a19c-4801-b854-2668f3b0c74c

App-Secret: 17a7cdbe...be17a1

```
{
  "logs": [
    {
      "type": "startService",
      "timestamp": "2021-04-25T23:20:04.173Z",
      "device": {
        "sdkName": "appcenter.android",
        "sdkVersion": "3.3.0",
        "model": "MAR-LX1B",
        "oemName": "HUAWEI",
        "osName": "Android",
        "osVersion": "9",
        "osBuild": "HUAWEIMAR-L21B",
        "osApiLevel": 28,
        "locale": "en_IE",
        "timeZoneOffset": 60,
        "screenSize": "1080x2107",
        "appVersion": "7.7.7",
        "carrierName": "Tesco Mobile",
        "carrierCountry": "ie",
        "appBuild": "954663024",
        "appName": "com.touchtype.swiftkey",
        "services": ["Crashes"]
      }
    }
  ]
}
```

The connection is initiated by app com.touchtype.swiftkey, a virtual keyboard developed by microsoft. The Install-ID is a device identifier (likely randomly generated when com.touchtype.swiftkey is first started). The carrier name and device details are sent in the message. These connections seem related to logging of app errors and sometimes include stacktraces.

GET https://tigger-citadel.touchtype-fluency.com/v1/store/items?format=670a8edf-0d05-48b3-ab1d-d5c267562062%3A1&locale=en_IE&package_name=com.touchtype.swiftkey&limit=100&referrer_id=HUAWEI

GET https://jenson.api.swiftkey.com/swiftkey/sksdk-3.0/sk-7.8.4/market/languagePacksSSL.json

<<< HTTP 200, 314.20KB

Set-Cookie: ApplicationGatewayAffinityCORS=

b6eb1f2645efcc010e3fc55b65613b25; Path=/; SameSite=None; Secure, ApplicationGatewayAffinity=b6eb1f2645efcc010e3fc55b65613b25; Path=/

POST https://bibo.api.swiftkey.com/v1/models

```
{
  "installId": "5bf64736-06...a8ec19e7",
  "params": {
    "cpuCount": 8,
    "deviceLocales": [
      "en_IE"
    ],
    "deviceManufacturer": "HUAWEI",
    "deviceModel": "MAR-LX1B",
    "fluencyVersion": "5.0.0.54",
    "imeVersion": "7.8.3.5",
    "isB2C": false,
    "packageName": "com.touchtype.swiftkey",
    "platformVersion": "28",
    "ramSize": 6000033792,
    "referrer": "HUAWEI",
    "totalDiskSpace": 1833746432
  },
  "supported": [
    {
      "category": "fluency",
      "subCategory": "params"
    }
  ],
}
```

<...>

The installId value is labelled as the installation_id in the app shared preferences file.

GET https://cloud-bibo-verizon-clsprd-eun.azureedge.net/sk-cloud/exp/bibo/models/BuqsKoKAfWwoOs...uVqj-zGUmfMYrT

POST https://telemetry.api.swiftkey.com/v1/bark-logs

Headers

Authorization: SwiftKeyTelemetry SwiftKey_Android_prod:

p5qpKmHth91cUQWD...Clz/IV0LzABbE=

The SwiftKey_Android_prod header value is a base64 encoded SHA256 hash of an API key, secret and the POST body. The POST body is gzipped data encoded in avro format. After extracting the schema from the app memory using Frida, the payload consists of a sequence of telemetry events. An example payload entry decodes as:

```
{
  "event": {
    "metadata": {
      "installId": "b'\xe7\x19\xec\xa8KD\xff\xa1&E\xa3\x066G'\xf6",
      "appVersion": "7.8.3.5",
      "timestamp": {
        "utcTimestamp": 1628056714179,
        "utcOffsetMins": 0
      },
      "vectorClock": {
        "major": 97,
        "minor": 1,
        "order": 100
      },
      "deviceInfo": {
        "os": {
          "name": "ANDROID",
          "version": "9",
          "model": "MAR-LX1B",
          "manufacturer": "HUAWEI",
          "architecture": "arm64-v8a",
          "cpus": 8,
          "totalRam": 6000033792,
          "screenMetrics": {
            "density": 480,
            "width": 1080,
            "height": 2107
          },
          "deviceId": "",
          "operator": {
            "name": "Tesco Mobile",
            "country": "ie",
            "locale": "en_IE",
            "language": "en",
            "advertisingId": "1628164900936-4da3-b2a8-cdf2382a02ba",
            "accessibilityScreenReaderEnabled": false,
            "pushNotificationId": "dDZHXIWncf0:APA91bFx6mNyUuy0Vp4...BpyFspBK_PD6A",
            "sdkVersion": "5.0.0.54"
          }
        }
      }
    }
  }
}
```

Observe that the Google adid/rdid advertising id is sent, as well as the mobile operator name. The pushNotificationId value is a Firebase authentication token for app with Firebase ID dDZHXIWncf0.

Other examples of events logged after typing briefly on the notepad app, searching within the contacts and messaging apps and using the searchbar:

```
{
  "event": {
    "metadata": {
      "installId": "b'\xe7\x19\xec\xa8KD\xff\xa1&E\xa3\x066G'\xf6",
      "appVersion": "7.8.3.5",
      "timestamp": {
        "utcTimestamp": 1628164900936,
        "utcOffsetMins": 0
      },
      "vectorClock": {
        "major": 103,
        "minor": 277,
        "order": 100
      },
      "application": "com.example.android.notepad",
      "durationMillis": 8995,
      "typingStats": {
        "totalTokensEntered": 4,
        "tokensFlowed": 3,
        "tokensPredicted": 0,
        "t"
      }
    }
  }
}
```

```
okensCorrected': 0, 'tokensVerbatim': 1, 'tokensPartial': 0, '
netCharsEntered': 19, 'deletions': 0, 'characterKeystrokes': 0, '
predictionKeystrokes': 0, 'remainderKeystrokes': 0, 'predictionSumLe
ngth': 0, 'typingDurationMillis': 1028, 'emojisEntered': 0, '
totalTokensEnteredEdited': 0, 'tokensFlowedEdited': 0, '
tokensPredictedEdited': 0, 'tokensCorrectedEdited': 0, '
tokensVerbatimEdited':
  0, 'tokensPartialEdited': 0}, 'languagesUsed': 0, 'termsPerLanguage': {
  en_GB': 2}, 'tokensPerSource': {'': 1, 'en_GB/en_GB.lm': 2}, '
tokensShownPerSource': {'en_GB/en_GB.lm': 26, 'en_GB/en_GB_w
ord_c.lm1': 4, 'user/dynamic.lm': 3}, 'userHandle': 0}}
```

```
{'event': {'metadata': {'installId': b'\xe7\x19\xec\xa8KD\xff\xa1&E\
xa3\x066G\xfb', 'appVersion': '7.8.3.5', 'timestamp': {'utcTimestamp':
1628164986036, 'utcOffsetMins': 0}, 'vectorClock': {'ma
jor': 103, 'minor': 359, 'order': 100}}, 'application': '
com.android.contacts', 'durationMillis': 6140, 'typingStats': {
  totalTokensEntered': 1, 'tokensFlowed': 0, 'tokensPredicted': 0, 'tokensCo
rrected': 0, 'tokensVerbatim': 1, 'tokensPartial': 0, 'netCharsEntered': 4, '
deletions': 0, 'characterKeystrokes': 0, 'predictionKeystrokes': 0, '
remainderKeystrokes': 0, 'predictionSumLength': 0
  , 'typingDurationMillis': 711, 'emojisEntered': 0, 'totalTokensEnteredEdited
': 0, 'tokensFlowedEdited': 0, 'tokensPredictedEdited': 0, '
tokensCorrectedEdited': 0, 'tokensVerbatimEdited': 0, 'toke
nsPartialEdited': 0}, 'languagesUsed': 0, 'termsPerLanguage': {}, '
tokensPerSource': {'': 1, 'tokensShownPerSource': {'': 3, 'en_GB/en_GB.
lm': 8, 'en_GB/en_GB_word_c.lm1': 2, 'user/dynamic.lm':
5}, 'userHandle': 0}}
```

```
{'event': {'metadata': {'installId': b'\xe7\x19\xec\xa8KD\xff\xa1&E\
xa3\x066G\xfb', 'appVersion': '7.8.3.5', 'timestamp': {'utcTimestamp':
1628165014657, 'utcOffsetMins': 0}, 'vectorClock': {'ma
jor': 103, 'minor': 482, 'order': 100}}, 'application': '
com.google.android.apps.messaging', 'durationMillis': 6891, 'typingStats':
  {'totalTokensEntered': 0, 'tokensFlowed': 0, 'tokensPredicted':
  0, 'tokensCorrected': 0, 'tokensVerbatim': 0, 'tokensPartial': 0, '
netCharsEntered': 3, 'deletions': 1, 'characterKeystrokes': 0, '
predictionKeystrokes': 0, 'remainderKeystrokes': 0, 'prediction
SumLength': 0, 'typingDurationMillis': 837, 'emojisEntered': 0, '
totalTokensEnteredEdited': 0, 'tokensFlowedEdited': 0, '
tokensPredictedEdited': 0, 'tokensCorrectedEdited': 0, 'tokensVerbatimEdit
ed': 0, 'tokensPartialEdited': 0}, 'languagesUsed': 0, 'termsPerLanguage':
  {}, 'tokensPerSource': {}, 'tokensShownPerSource': {'': 6, 'en_GB/en_GB
.lm': 16, 'user/dynamic.lm': 6}, 'userHandle': 0}
}
```

```
{'event': {'metadata': {'installId': b'\xe7\x19\xec\xa8KD\xff\xa1&E\
xa3\x066G\xfb', 'appVersion': '7.8.3.5', 'timestamp': {'utcTimestamp':
1628249861067, 'utcOffsetMins': 0}, 'vectorClock': {'major': 20, 'minor':
73, 'order': 100}}, 'application': 'c
om.google.android.googlequicksearchbox', 'durationMillis': 11837, '
typingStats': {'totalTokensEntered': 0, 'tokensFlowed': 0, 'tokensPredicted':
  0, 'tokensCorrected': 0, 'tokensVerbatim': 0, 'tokensPartial': 0, '
netCharsEntered': 0, 'deletions': 0, 'characterKeystrokes': 0, '
predictionKeystrokes': 0, 'remainderKeystrokes': 0, 'predictionSumLength':
  0, 'typingDurationMillis': 0, 'emojisEntered': 0, 'totalTokensEnteredEdited':
  0, 'tokensFlowedEdited': 0, 'tokensPredictedEdited': 0, '
tokensCorrectedEdited': 0, 'tokensVerbatimEdited': 0, 'tokensPartialEdited':
  0}, 'languagesUsed': 0, 'termsPerLanguage': {}, 'tokensPerSource': {}, '
tokensShownPerSource': {}, 'userHandle': 0}}
```

Observe that interaction with each app is logged, together with a timestamp that as millisecond accuracy. User interactions with the swiftkey settings is also logged in detail:

```
{'event': {'metadata': {'installId': b'\xe7\x19\xec\xa8KD\xff\xa1&E\
xa3\x066G\xfb', 'appVersion': '7.8.3.5', 'timestamp': {'utcTimestamp':
1628164764733, 'utcOffsetMins': 0}, 'vectorClock': {'ma
jor': 103, 'minor': 32, 'order': 100}}, 'pageName': '
TYPING_AUTOCORRECT_SETTINGS', 'prevPageName': '
SETTINGS', 'pageOrigin': 'SETTINGS', 'id': '5a3e7a5d-eb76-4bb1-
85b1-c09c16b4056b'}}
```

```
{'event': {'metadata': {'installId': b'\xe7\x19\xec\xa8KD\xff\xa1&E\
xa3\x066G\xfb', 'appVersion': '7.8.3.5', 'timestamp': {'utcTimestamp':
1628164771391, 'utcOffsetMins': 0}, 'vectorClock': {'ma
jor': 103, 'minor': 34, 'order': 100}}, 'pageName': '
TYPING_STATS_SETTINGS', 'prevPageName': '
TYPING_AUTOCORRECT_SETTINGS', 'pageOrigin': 'SETTINGS', '
id': '5a3e7a5d-eb76-4bb1-85b1-c09c16b4056b'}}
```

```
}
```

```
{'event': {'metadata': {'installId': b'\xe7\x19\xec\xa8KD\xff\xa1&E\
xa3\x066G\xfb', 'appVersion': '7.8.3.5', 'timestamp': {'utcTimestamp':
162816477166, 'utcOffsetMins': 0}, 'vectorClock': {'ma
jor': 103, 'minor': 37, 'order': 100}}, 'action': 'OPEN_HEATMAP'}}
The 'id' appears to be a session id, linking together interactions in the
same session e.g. as the user moves from one settings page to another.
Similar logging occurs of user interactions with the keyboard e.g. opening
the clipboard.
```

GET https://www.dailymotion.com/embed/?app=com.huawei.himovie.overseas&like-enable=false&collections-enable=false&syndication=273832&sharing-enable=false&watchlater-enable=false&queue-enable=0&client_type=androidapp&fullscreen-action=trigger_event&ads_device_tracking=1&locale=en&api=nativeBridge&ads_device_id=228b814c-14fe-4da3-b2a8-cdf2382a02ba

Headers

```
X-Requested-With: com.huawei.himovie.overseas
<<< HTTP 200, 19.56KB
Set-Cookie: dmvk=60862392b6342; path=/; domain=.dailymotion.com;
Secure; SameSite=none;
Set-Cookie: v1st=93BA99B9572EFCF547B8E2F83A26CBD3; expires
=Thu, 26 May 2022 02:21:06 GMT; max-
<response is HTML/javascript that contains embedded identifiers:
```

```
...
window.__PLAYER_CONFIG__ = {"context":{"access_token":null,"
ad_sync_script_url":"https://dmxleo.dailymotion.com/cdn/manifest/
video/xnhs5y0.m3u8?auth=1619576466-25...kp2crmp&bs=1","advertising
":{"stack":"","dm":"","ima":false},"api":{"url":"https://graphql.api.dailymotion.
com"},"auth_url":"https://graphql.api.dailymotion.com/oauth/token","
client_id":"f1a362d288c1b98099c","client_secret":"
eea605b96e01c796ff369935357eca920c5da4c5"
...
referer=referer&syndication=273832&app=com.huawei.himovie.overseas&
client_type=androidapp&ads_device_tracking=1&locale=en&ads_device_id=
228b814c-14fe-4da3-b2a8-cdf2382a02ba"
...
>
```

The client_id value is the Google adid/rdid. The response sets several cookies and identifiers, including the Google adid/rdid, in the html response.

GET https://static1.dmcnd.net/player/v5/dmp.jq_flight.3033f0d7176196134921.es5.js

```
referer: https://www.dailymotion.com/embed/?app=com.huawei.himovie.
overseas&like-enable=false&collections-enable=false&syndication
=273832&sharing-enable=false&watchlater-enable=false&queue-enable
=0&client_type=androidapp&fullscreen-action=trigger_event&
ads_device_tracking=1&locale=en&api=nativeBridge&ads_device_id=
228b814c-14fe-4da3-b2a8-cdf2382a02ba
```

GET <https://static1.dmcnd.net/player/v5/dmp.vendor.cd429aba649c69f863e7.es5.js>

```
referer: https://www.dailymotion.com/embed/?app=com.huawei.himovie.
overseas&like-enable=false&collections-enable=false&syndication
=273832&sharing-enable=false&watchlater-enable=false&queue-enable
=0&client_type=androidapp&fullscreen-action=trigger_event&
ads_device_tracking=1&locale=en&api=nativeBridge&ads_device_id=
228b814c-14fe-4da3-b2a8-cdf2382a02ba
```

Followed by several similar connections

GET http://update.dbankcdn.com/download/data/pub_13/HWHOTA_hotaMigrate_900_9/bf/v3/9324011adc13435394ab409987f58b48/full/filelist.xml

POST <https://pebed.dm-event.net/>

Headers

```
Origin: https://www.dailymotion.com
X-Requested-With: com.huawei.himovie.overseas
Sec-Fetch-Site: cross-site
Sec-Fetch-Mode: cors
Sec-Fetch-Dest: empty
Content-Encoding: snappy
Referer: https://www.dailymotion.com/embed/?app=com.huawei.himovie.
overseas&like-enable=false&collections-enable=false&syndication
=273832&sharing-enable=false&watchlater-enable=false&queue-enable
=0&client_type=androidapp&fullscreen-action=trigger_event&
ads_device_tracking=1&locale=en&api=nativeBridge&ads_device_id=
```

228b814c-14fe-4da3-b2a8-cdf2382a02ba

The POST payload is encoded using the Snappy (see

https://google.github.io/snappy/) serialisation format. It decodes to:
 '{"channel":"player","edward":"1.1","sent_ts":1619403666630,"v1st":"93BA99B9572EFCF547B8E2F83A26CBD3","events":[{"name":"life_cycle.initStart","version":"1.0","data":{"context":{"visitor_group":33},"created_ts":1619403666629,"info":{"app":{"id":"com.huawei.himovie.overseas"},"is_native":true,"type":"androidapp"},"browser":{"data_saver_detected":false,"effective_connection_type":"4g","locale":"en","os_family":"Android"},"os_name":"Android 9.0 Pie","ua_family":"Dailymotion App","ua_name":"Dailymotion App","user_agent":"Mozilla/5.0 (Linux; Android 9; MAR-LX1B Build/HUAWEIMAR-L21B; wv) AppleWebKit/537.36 (KHTML, like Gecko) Version/4.0 Chrome/90.0.4430.82 Mobile Safari/537.36; dailymotion-player-sdk-android 0.1.31"},"device":{"type":"mobile"},"player":{"env":{"prod"},"instance_uuid":"2faccc77-e970-aca1-6660-a6051e8e8b52"},"integration":{"iframe"},"is_westeros_embed":false,"secure":true,"topdomain":"www.dailymotion.com","version":"v-0.0.2466-rc1"},"x_requested_with":"com.huawei.himovie.overseas"},"visitor":{"as_number":"AS6830","continent":"EU","country":"IE","id":"","onsite":false,"region":"07","timezone_offset":360000,"traffic_segment":326088},"publisher":{"xid":"x29y3xe"},"settings":{"empty_player":true}},"stack_ctx":{"player_stack":{"noworker"}}}'
 <<< HTTP 200, 15.00B

GET https://imasdk.googleapis.com/js/sdkloader/ima3.js HTTP/2.0
 x-requested-with: com.huawei.himovie.overseas
 sec-fetch-site: cross-site
 sec-fetch-mode: no-cors
 sec-fetch-dest: script
 referer: https://www.dailymotion.com/embed/?app=com.huawei.himovie.overseas&like-enable=false&collections-enable=false&syndication=273832&sharing-enable=false&watchlater-enable=false&queue-enable=0&client_type=androidapp&fullscreen-action=trigger_event&ads_device_tracking=1&locale=en&api=nativeBridge&ads_device_id=228b814c-14fe-4da3-b2a8-cdf2382a02ba

POST http://query.hicloud.com/himovie/v2/CheckEx.action
 '{"components":[{"PackageName":"com.huawei.himovie.overseas","PackageVersionCode":"80550308","PackageVersionName":"8.5.50.308","sign":"528E0A2B8...4092C","region":{"Local"}],"rules":{"FingerPrint":"HUAWEI/MAR-LX1BEEA/HWMAR-9/HUAWEIMAR-L21B/V9.1.0.372C431:user/release-keys","DeviceName":"MAR-LX1B","FirmWare":"MAR-L21B 9.1.0.372(C431E2R4P1)","IMEI":"RSA:aV9vb...kVXfxKAs","IMSI":"RSA:Uc3UldIua...Twhx60aZ8","Language":{"en-ie","OS":"Android 9","C_version":"C431","D_version":"D000"}}}'
 <<< HTTP 200, 289.00B

Despite the suggestive field names, the IMEI value appears to be an RSA encrypted random UUID and the IMSI value to be the RSA encrypted mobile operator MNC/MCC id i.e. not the SIM IMSI (a unique SIM identifier)

GET https://speedtest.dailymotion.com/latencies.js
 Headers
 X-Requested-With: com.huawei.himovie.overseas
 Referer: https://www.dailymotion.com/embed/?app=com.huawei.himovie.overseas&like-enable=false&collections-enable=false&syndication=273832&sharing-enable=false&watchlater-enable=false&queue-enable=0&client_type=androidapp&fullscreen-action=trigger_event&ads_device_tracking=1&locale=en&api=nativeBridge&ads_device_id=228b814c-14fe-4da3-b2a8-cdf2382a02ba
 Accept-Encoding: gzip, deflate
 Accept-Language: en-IE,en-US;q=0.9,en;q=0.8
 Cookie: ts=326088; dmvk=60862392b6342; v1st=93BA99B9572EFCF547B8E2F83A26CBD3
 <<< HTTP 200, 7.17KB
The cookie of this request is embedded in a request to pebed.dm-event.net as an id.

GET https://s0.2mdn.net/instream/video/client.js
 referer: https://www.dailymotion.com/embed/?app=com.huawei.himovie.overseas&like-enable=false&collections-enable=false&syndication=273832&sharing-enable=false&watchlater-enable=false&queue-enable=0&client_type=androidapp&fullscreen-action=trigger_event&ads_device_tracking=1&locale=en&api=nativeBridge&ads_device_id=228b814c-14fe-4da3-b2a8-cdf2382a02ba

GET https://pagead2.googlesyndication.com/omsdk/releases/live/omweb-v1.js
 x-requested-with: com.huawei.himovie.overseas
 sec-fetch-site: cross-site
 sec-fetch-mode: no-cors
 sec-fetch-dest: script
 referer: https://www.dailymotion.com/embed/?app=com.huawei.himovie.overseas&like-enable=false&collections-enable=false&syndication=273832&sharing-enable=false&watchlater-enable=false&queue-enable=0&client_type=androidapp&fullscreen-action=trigger_event&ads_device_tracking=1&locale=en&api=nativeBridge&ads_device_id=228b814c-14fe-4da3-b2a8-cdf2382a02ba

GET https://dmxleo.dailymotion.com/cdn/manifest/video/xnhs5y0.m3u8?auth=1619576466-2...4c3w0kp2crmp&bs=1&cookie_sync_ab_gk=1&reader_gdpr_flag=1&reader_gdpr_consent=&gdpr_binary_consent=opt-out&gdpr_comes_from_infopack=0&reader_us_privacy=1--- HTTP/2.0
 user-agent: Mozilla/5.0 (Linux; Android 9; MAR-LX1B Build/HUAWEIMAR-L21B; wv) AppleWebKit/537.36 (KHTML, like Gecko) Version/4.0 Chrome/90.0.4430.82 Mobile Safari/537.36;dailymotion-player-sdk-android 0.1.31
 x-requested-with: com.huawei.himovie.overseas
 sec-fetch-site: same-site
 sec-fetch-mode: no-cors
 sec-fetch-dest: script
 referer: https://www.dailymotion.com/embed/?app=com.huawei.himovie.overseas&like-enable=false&collections-enable=false&syndication=273832&sharing-enable=false&watchlater-enable=false&queue-enable=0&client_type=androidapp&fullscreen-action=trigger_event&ads_device_tracking=1&locale=en&api=nativeBridge&ads_device_id=228b814c-14fe-4da3-b2a8-cdf2382a02ba
 cookie: ts=326088; dmvk=60862392b6342;
 v1st=93BA99B9572EFCF547B8E2F83A26CBD3

GET https://www.dailymotion.com/favicon.ico
 referer: https://www.dailymotion.com/embed/?app=com.huawei.himovie.overseas&like-enable=false&collections-enable=false&syndication=273832&sharing-enable=false&watchlater-enable=false&queue-enable=0&client_type=androidapp&fullscreen-action=trigger_event&ads_device_tracking=1&locale=en&api=nativeBridge&ads_device_id=228b814c-14fe-4da3-b2a8-cdf2382a02ba
 cookie: ts=326088; dmvk=60862392b6342;
 v1st=93BA99B9572EFCF547B8E2F83A26CBD3

GET https://speedtest.dailymotion.com/speedtest
 Origin: https://www.dailymotion.com
 Sec-WebSocket-Version: 13
 Cookie: ts=326088; dmvk=60862392b6342;
 v1st=93BA99B9572EFCF547B8E2F83A26CBD3
 Sec-WebSocket-Key: Bh6zGCdgwRkoMwKg4ms6QQ==
 Sec-WebSocket-Extensions: permessage-deflate;
 client_max_window_bits
 Sec-WebSocket-Protocol: speedtest

Additional connections when logged in to Google:
Connections to Google servers are similar to those for the Samsung handset, in particular connections to: android.googleapis.com/auth, inbox.google.com, mail.google.com, lamssettings-pa.googleapis.com, cryptauthdevicesync.googleapis.com, youtubei.googleapis.com, i.ytimg.com, www.googleadservices.com, app-measurement.com, www.google.com/complete/search, footprints-pa.googleapis.com, people-pa.googleapis.com, geller-pa.googleapis.com.

POST https://android.googleapis.com/auth
 Headers
 device: 3dd0519daa2cc3ba
 app: com.google.android.videos
 Accept-Encoding: gzip
 User-Agent: GoogleAuth/1.4 (HWMAR HUAWEIMAR-L21B); gzip
 content-type: application/x-www-form-urlencoded

'androidId=3dd0519daa2cc3ba&lang=en-IE&google_play_services_version=211213030&sdk_version=28&device_country=ie&app=com.google.android.videos&check_email=1&oauth2_foreground=1&Email=doug.leith@gmail.com&token_request_options=CAA4AVAB&service=oauth2:https://www.googleapis.com/auth/android_video&client_sig=24bb24c05e4...613a600&system_partition=1&callerPkg=com.google.android.videos&Token=aas_et/AKppINbbMrV...tv8JXw4U=&callerSig=24bb24c0...613a600'

<<< HTTP 200, 451.00B

App com.google.android.videos authenticating with google. In addition the following services are authenticated: googleplay, android_video, drive.metadata.readonly, drive.labels.readonly, drive.activity.readonly, docs, drive.readonly, peopleapi.readonly, drive.apps, cloudprint, activity, drive.file, gmail.readonly, subscriptions, memento, notifications, spreadsheets, vouchers, discussions, userlocation.reporting, peopleapi.legacy.readwrite, reminders, calendar, playatoms, mobileapps.doritos.cookie, peopleapi.readwrite, OAuthLogin, sierra, webhistory, gmail.full_access, gmail.ads, gmail.locker.read, taskassist.readonly, gmail.publisher_first_party, experimentsandconfigs, tachyon, numberer, firebase.messaging, userinfo.email, gcm, android_checkin, login_manager, cryptauth, notifications

POST https://reminders-pa.googleapis.com/caribou.tasks.service.TasksApiService/ListTasks

Headers

authorization: Bearer ya29.m.CvkB...gIIAQ

POST https://people-pa.googleapis.com/google.internal.people.v2.InternalPeopleService/SyncPeople

Headers

x-goog-spatula: CjYK...oljhg=

authorization: Bearer ya29.m.Cv...CAE

POST https://footprints-pa.googleapis.com/footprints.oneplatform.FootprintsService/GetActivityControlsSettings

Headers

user-agent: com.google.android.gms/211213030 (Linux; U; Android 9; en_IE; MAR-LX1B; Build/HUAWEIMAR-L21B; Cronet/85.0.4183.127)
authorization: Bearer ya29.m.CvgB...ggB

POST https://chromesyncpasswords-pa.googleapis.com/google.internal.chrome.sync.passwords.v1.Passwords/GetMetadata

Headers

x-goog-spatula: CjY...MyjvAFsMmrVPZPoljhg=

authorization: Bearer ya29.m.Cvk...IAQ

GET https://www.google.com/complete/search?oe=utf-8&gcc=ie&ctzn=Europe%2FDublin&ctf=0&v=10.85.11.21.arm64&ntyp=1&ram_mb=5722&ar=0&innm=asst&hl=en-IE&noj=1&client=qsb-android-asbl-pb&qsubts=1619248256204&padt=200&padb=703&cds=0&gs_per=t&q=&cp=0&psi=CHUOqSbnfx.1619090017602.0&ech=22&dpr=3.0&gs_pcr=1&xssi=t

Headers

x-client-instance-id:

41ddb01e251426cd9bb6fbd51f8c408584c57421e72f11272350d51fd8aad588

x-agsa-user-is-unicom: 2

x-geo: w CAEQDJoBBloECAMQZA==

x-client-data: aqwD...BgCAAA

cookie: CONSENT=PENDING+078;

SID=8wefx2_ZJOe_VO...KTKn/AXy0WaXHMthuOM1_q

<<< HTTP 200, 24.00KB

POST https://inbox.google.com/sync/st/s?hl=en_IE&c=0

Headers

x-gmail-btai: GkwwAF...Lw==

user-agent: Android-Gmail/61993624 (sw360dp; 480dpi) (HWMAR HUAWEIMAR-L21B)

authorization: OAuth ya29.a0AFH6...IJM

Additional connections when location enabled

GET http://radiomap.vcdn.pos.here.com/p/d/radiomap/b/a/master.bin

GET http://radiomap.vcdn.pos.here.com/p/d/radiomap/b/ar/1544030032588/index.bin

Followed by several similar connections

POST https://mobilenetworkscoring-pa.googleapis.com/v1/GetWifiQuality?key=AlzaSyBr...A6ThtVczU

Headers

Content-Type: application/x-protobuf

X-Goog-Spatula: CjYKfMnVb...rvKPZPoljhg=

User-Agent: GmsCore/211213030 (HWMAR HUAWEIMAR-L21B);

gzip

Accept-Encoding: gzip

2 {

1: SxZvV-A2v6hCIsYTGnKDDcgxHor8RCW072wKBTEMEs0

}

3: 27211

4: ..

<<< HTTP 200, 132.00B

The POST body is a protobuf. The "27211" value is the mobile carrier id. Based on discussions with Google the other value is a of the Wifi MAC address of nearby access points, used to query a network quality database to determine the best Wifi network to connect to.

POST https://www.googleapis.com/geolocation/v1/geolocate?key=AlzaSyAP-gfH...hzIk

Headers

x-android-package: com.google.android.gms

x-android-cert: 38918A4...6562CED5788

user-agent: com.google.android.gms/211213030 (Linux; U; Android 9; en_IE; MAR-LX1B; Build/HUAWEIMAR-L21B; Cronet/85.0.4183.127)

'{"considerIp": "true"}'

<<< HTTP 200, 89.00B

{

"accuracy": 15736,

"location": {

"lat": 53.2840448,

"lng": -6.455296

}

Google may use the IP address to determine the geolocation. See the API: https://developers.google.com/maps/documentation/geolocation/overview. The returned GPS coordinates are within about 5-10Km of the true handset location.

GET https://74.125.193.94/spectrum-geolocation-scs/geofence/full_world_launch_v2_geofence.pb

user-agent: Dalvik/2.1.0 (Linux; U; Android 9; MAR-LX1B Build/HUAWEIMAR-L21B)

POST https://firebaseinstallations.googleapis.com/v1/projects/tachyon-android/installations

Headers

X-Android-Package: com.google.android.apps.tachyon

x-firebase-client: fire-core/19.3.2_1p fire-fcm/20.1.7_1p fire-android/fire-installations/16.3.6_1p fire-analytics/18.0.3 fire-iid/21.1.0

x-firebase-client-log-type: 3

X-Android-Cert: A0BC09AF...76F757BECC3

x-goog-api-key: AlzaSyAYU6...Ov6tQaIM

x-goog-fis-android-iid-migration-auth:

eEcXrIZORVU:A..EvrzMTg6c

'{"fid": "eEcXrIZORVU", "appId": "1206908507205:android:167bd0ff59cd7d44", "authVersion": "FIS_v2", "sdkVersion": "a:16.3.6_1p"}'

<<< HTTP 200, 538.00B

App com.google.android.apps.tachyon connecting to firebase. Similar connections are also made by: com.google.android.apps.messaging, com.google.android.googlequicksearchbox, com.google.android.youtube, com.google.android.gm, com.android.vending

Connections When Insert Sim

POST https://android.clients.google.com/fdfe/uploadDynamicConfig

Headers

user-agent: Android-Finsky/22.4.25-21%20%5B0%5D%20%5BPR%5D%20337959405 (api=3,versionCode=82242510,sdk=28,device=HWMAR,hardware=kirin710,product=MAR-LX1BEEA,platformVersionRelease=9,model=MAR-LX1B,buildId=HUAWEIMAR-L21B,isWideScreen=0,supportedAbis=arm64-v8a;armeabi-v7a;armeabi)

x-dfe-device-id: 3dd0519daa2cc3ba

x-dfe-device-config-token: CisaKQoTNDQ1...DgzOTU10TA4MTkx

accept-language: en-IE

x-dfe-device-checkin-consistency-token: ABFE...Gsj

```

x-dfe-network-type: 4
x-dfe-mccmnc: 27211
x-dfe-client-id: am-android-huawei
x-dfe-phenotype: H4s...AA
x-dfe-encoded-targets: CAESGYmbgQb...Bg
x-dfe-request-params: timeoutMs=4000
x-dfe-build-fingerprint: HUAWEI/MAR-LX1BEEA/HWMAR:9/
HUAWEIMAR-L21B/9.1.0.372C431:user/release-keys
content-type: application/x-protobuf
accept-encoding: gzip, deflate, br

```

POST body:

```

'1 {
  1: "GMT+00:00"
  2 {
    1 {
      1: 272110104300000
      2: "Tesco Mobile"
      3: "0AFFFFFFFFFFFFFFFFFFFFFFFFFFFFFFFF"
      6: 18446744073709551615
    }
  }
  3 {
    1: "com.google.android.gms"
    2: "OJGKRT0HGZNU-LGa8F7GViztV4g"
    3: "8P1sW0EPI...ICtay1g24M"
    4: 3
  }
  4: "cImAYqzEo2q4AM3q...NFWiKs"
}

```

The carrier name MCC and MNC are sent to google when the sim card is inserted, plus the SIM IMSI

POST https://query.hicloud.com/sp_ard_common/v2/onestopCheck.action?
verType=true&autoInstall=y&latest=true

Headers

```

Content-Type: application/json; charset=UTF-8
Accept-Encoding: identity
{"commonRules":{"FingerPrint":"HUAWEI/MAR-LX1BEEA/HWMAR:9/HUAWEIMAR-L21B/9.1.0.372C431:user/release-keys",
DeviceName":"MAR-L21B","VendorCountry":"hw-eu","BoardID":"7928",
Reserved1":"","Reserved2":"","vendor":"MAR-L21B-hw-eea","deviceId":
"FUNDU20924007430","PLMN":"27211","udid":"
0C3B05F3379E4EDB40D...7C177AF08F4A39E09C0B","Language":"en-
ie","OS":"Android 9","HotaVersion":"9.1.30.391","saleinfo":{"black|eea|hw|
N|EmotionUI_9.1.0|6.4 GB|256 GB|8_2.2GHz|N|blue|9.1.30.391|Y",
C_version":"C431","D_version":"D000","Subgroup":"","Vergroup":"","
devicetoken":"","PackageType":"increment","ControlFlag":"0",
DeviceDisplayVersion":"MAR-L21B 9.1.0.372(C431E2R4P1)",
ProductBaseVersion":"MAR-LGRP2-OVS 9.1.0.372","Emui":"
EmotionUI_9.1.0"},"versionPackageRules":[{"versionPackageType":2,"rules":
{"FirmWare":"MAR-LGRP2-OVS 9.1.0.372"}},{"versionPackageType":
3,"rules":{"FirmWare":"MAR-L21B-CUST 9.1.0.2(C431)}},{
versionPackageType":4,"rules":{"FirmWare":"MAR-L21B-PRELOAD
9.1.0.1(C431R4)"}]},"keyAttestation":"r00ABXN...="}
<<<< HTTP/200, 623.00B

```

The carrier MCC and MNC are sent to hicloud.com when the sim card is inserted.

C. Connections When Interacting With Settings App

POST http://mvconf.cloud.360safe.com/safe_update

The POST body is encrypted. It decrypts to:

```

sdkid=2
permission=com.qihoo.antivirus.update.permission.clear_sdk_default
uilang=936
pa=x86
mid=81d47510792a6f616daa03580517e215
cid=107415
product=clear_sdk_multilang
combo=mobile
sdk=28
updsene=2
updsetting=1
pkg=com.huawei.systemmanager
connect_type=1

```

```

ver=6.4.7.1005
date=2021.08.02 09:22:15
imei=3e8913e0-5b79-47b9-8fde-a6671bffb66d
free_disk=0
wifi=1
brand=HUAWEI
model=MAR-LX1B
free_disk_x=0
uv=1

```

The mid value is the imei value encoded as a hex string i.e. the imei string is decode to bytes and then the hex values of the bytes formed into a new string. The imei value appears to be a random UUID generated when the app is first started i.e. following a factory reset.

GET http://msafedl.ssl.qihucdn.com/clear_sdk_multilang/20201015/o_c_tcw.dat

POST https://aiclean.us.cloud.360safe.com/video/clean

POST http://mclean.cloud.360safe.com/CleanQuery

The POST body appears is encrypted.

GET https://pagead2.googlesyndication.com/pagead/gen_204?id=gmob-apps
&action=ads_settings_page_view&device=HUAWEI%20MAR-LX1B&js
=211213030.211213000&os=9&api=28&eids=318497136%2C318475417%2
C318489659%2C318495357%2C318495359%2C318494842&appid=com.
google.android.gms

POST https://query.hicloud.com/sp_ard_common/v2/onestopCheck.action?
verType=true&autoInstall=y&latest=true
{"commonRules":{"FingerPrint":"HUAWEI/MAR-LX1BEEA/HWMAR:9/HUAWEIMAR-L21B/9.1.0.372C431:user/release-keys",
DeviceName":"MAR-L21B","BoardID":"7928","VendorCountry":"hw-eea",
Reserved1":"","Reserved2":"","deviceId":"FUNDU20924007430","udid":
"0C3B05F3379E4E...A17C177AF08F4A39E09C0B","Language":"en-ie",
OS":"Android 9","HotaVersion":"9.1.30.391","C_version":"C431",
D_version":"D000","DeviceDisplayVersion":"MAR-L21B 9.1.0.372(C431E2R4P1)",
ProductBaseVersion":"MAR-LGRP2-OVS 9.1.0.397",
Emui":"EmotionUI_9.1.0","vnkey":"27211","vendorCota":"hw-eu",
vendorExpiredTime":"1619686874155","ControlFlag":"0","PackageType":
full","Type":"ATL"},"versionPackageRules":[{"versionPackageType":5,
rules":{"FirmWare":"Cota000"}]},"keyAttestation":"r00ABXNyAC1...
VguNTA5","deviceCertificate":"r00ABX...WC41MDk="}

D. Connections When Sending a Text

POST https://app-measurement.com/a

When sending a text the messaging app com.google.android.apps.messaging logs user interactions using Google Analytics. The POST body is a protobuf that decodes to:

POST https://app-measurement.com/a

```

body {
  always_one: 1
  event {
    event_info {
      setting_code: "o" // firebase_event_origin
      data_str: "auto"
    }
    event_info {
      setting_code: "et" // engagement_time_msec
      data_int: 14080
    }
    event_info {
      setting_code: "sc" // firebase_screen_class
      data_str: "HomeActivity"
    }
    event_info {
      setting_code: "si" // firebase_screen_id
      data_int: 7145169158988050373
    }
    event_info {
      setting_code: "fr"
      data_int: 1
    }
    event_code: "e" // user_engagement
    event_timestamp: 1619420409836
    previous_event_timestamp: 1619420395722
  }
}

```

```

event {
  event_info {
    setting_code: "_o" // firebase_event_origin
    data_str: "auto"
  }
  event_info {
    setting_code: "_pc" // firebase_previous_class
    data_str: "HomeActivity"
  }
  event_info {
    setting_code: "_pi" // firebase_previous_id
    data_int: 7145169158988050373
  }
  event_info {
    setting_code: "_sc" // firebase_screen_class
    data_str: "ConversationActivity"
  }
  event_info {
    setting_code: "_si" // firebase_screen_id
    data_int: 7145169158988050375
  }
  event_info {
    setting_code: "_et" // engagement_time_msec
    data_int: 14080
  }
  event_code: "_vs" // screen_view
  event_timestamp: 1619420410003
  previous_event_timestamp: 1619420395778
}
event {
  event_info {
    setting_code: "_o" // firebase_event_origin
    data_str: "app"
  }
  event_info {
    setting_code: "_sc" // firebase_screen_class
    data_str: "ConversationActivity"
  }
  event_info {
    setting_code: "_si" // firebase_screen_id
    data_int: 7145169158988050375
  }
  event_info {
    setting_code: "_c" // firebase_conversion
    data_int: 1
  }
  event_info {
    setting_code: "_r"
    data_int: 1
  }
  event_code: "ACTIVE_EVENT"
  event_timestamp: 1619420432814
}
event {
  event_info {
    setting_code: "_o" // firebase_event_origin
    data_str: "app"
  }
  event_info {
    setting_code: "_sc" // firebase_screen_class
    data_str: "ConversationActivity"
  }
  event_info {
    setting_code: "_si" // firebase_screen_id
    data_int: 7145169158988050375
  }
  event_info {
    setting_code: "_c" // firebase_conversion
    data_int: 1
  }
  event_info {
    setting_code: "_r"
    data_int: 1
  }
  event_code: "FIRST_MESSAGE_SENT"
  event_timestamp: 1619420432814
}

```

```

user {
  timestamp: 1619075380570
  setting: "_fot"
  data_int: 1619078400000
}
user {
  timestamp: 1619075380570
  setting: "_fi"
  data_int: 1
}
user {
  timestamp: 1619420394445
  setting: "_sno" // session_number
  data_int: 1
}
user {
  timestamp: 1619420394445
  setting: "_sid" // session_id
  data_int: 1619420394
}
user {
  timestamp: 1619420442973
  setting: "_lte"
  data_int: 15118
}
user {
  timestamp: 1619420442976
  setting: "_se"
  data_int: 15117
}
message_timestamp: 1619420442957
event_timestamp: 1619420409836
bundle_end_timestamp: 1619420432814
last_bundle_end_timestamp: 1619420395778
operating_system: "android"
operating_system_version: "9"
Build_MODEL: "MAR-LX1B"
language_country: "en-ie"
timezone_offset_mins: 60
package_name: "com.google.android.apps.messaging"
app_version: "7.7.050 (Neem_RC02.phone_dynamic)"
gmp_version: 39058
gms_version: 211213
google_ad_id: "228b814c-14fe-4da3-b2a8-cdf2382a02ba"
random_hex: "0704d96e627718e4e33f3085d3ca0372"
dev_cert_hash: 7599436411597265477
daily_conversions_count: 5
gmp_app_id: "1:357317899610:android:4765c0ded882c665"
last_bundle_end_timestamp2: 1619420394445
always_true: true
firebase_instance_id: "eidUaKjTTpk"
app_version_int: 77050063
config_version: 1591635597861345
M: 23180929
M: 23180918
M: 23180912
dynamite_version: 48
unknown: "G1—"
}

```

commentIt can be seen that user interactions with the HomeActivity and ConversationActivity are logged, and a FIRST_MESSAGE_SENT event is also logged.

IV. REALME

Summary:

<i>Realme system app endpoints:</i><br>ifrus-eu.coloros.com, ifotaeu.realmemobile.com<br>icosa-eu.coloros.com<br>esa-reg-eup.myoppo.com	<i>Identifiers Sent:</i><br>guid, registrationId<br><br>guid<br>IMEI, guid
<i>Third-party (non-Google) system app endpoints:</i><br>httpdns-euex-push.heyta mobile.com<br>adx-f.ads.heyta mobile.com<br>shorteux.push.heyta mobile.com<br><br>dceux.push.heyta mobile.com	<i>Identifiers Sent:</i><br><br>deviceId, DUID/VAID<br>GAID, OUID<br>DUID/VAID, OUID, device_id, fcm_token (Firebase)<br>DUID/VAID, OIUD
Identifiers observed to persist across factory reset	IMEI, deviceId, guid
Notes:<br>1. device_id value is returned by shorteux.push.heyta mobile.com in response to request containing DUID/VAID and OUID values and so device_id, DUID/VAID, OIUD are linked.<br>2. registrationId value is returned by shorteux.push.heyta mobile.com in response to req containing the device_id, so registrationId, device_id, DUID/VAID, OIUD are linked.	

TABLE VII

SUMMARY OF IDENTIFIERS SENT IN SYSTEM APP CONNECTIONS (EXCLUDING GOOGLE SYSTEM APPS).

<i>Telemetry</i><br>dceux.push.heyta mobile.com	Logs events associated with com. heyta.mcs, e.g. launch success and push_register, together with a timestamp
<i>Device Data</i><br>ifrus-eu.coloros.com,<br>ifotaeu.realmemobile.com<br>icosa-eu.coloros.com<br>adx-f.ads.heyta mobile.com<br>shorteux.push.heyta mobile.com<br>dceux.push.heyta mobile.com	Device details<br><br>Installed apps<br>Device details<br>Device details, installed apps<br>Device details

TABLE VIII

SUMMARY OF DATA SENT IN SYSTEM APP CONNECTIONS (EXCLUDING GOOGLE SYSTEM APPS).

- 1) The handset sends DUID/VAID, OAID and device_id and identifier values in connections to shorteux.push.heyta mobile.com. It also sends the Google Firebase ID and authentication token associated with the com. heyta.mcs app. The OAID value and Google adid/r-did Advertising Id (GAID) are sent in connections to adx-f.ads.heyta mobile.com, linking data collection by Google and Realme. The encrypted device_id value is sent in MQTT encoded messages over a TCP connection. All of these identifiers change upon a factory reset of the device. However, in connections to httpdns-euex-push.heyta mobile.com a long-lived deviceId value is sent that persists across factory resets, along with the VAID value. While the VAID value changes on a factory reset, because the deviceId value remains unchanged the new VAID value can be relinked to the device. Since the VAID is sent along with the DUID/VAID, OAID, device_id and Firebase ID/token values, these also can be relinked. Since the OAID and GAID are sent together, the GAID can be reinked to the device.

The domain shorteux.push.heyta mobile.com does not appear to be registered to Realme. The SSL cert offered by shorteux.push.heyta mobile.com indicates that it is operated by Bravo Unicorn Pte. Ltd, Singapore, a private holding company. This matches the company information at <https://www.heyta.com/en/about-us.html>.

- 2) The handset also connects to icosaeu.coloros.com, ifruseu.coloros.com, classifyeu.apps.coloros.com and ifotaeu.realmemobile.com. These domains are associated with Oppo, the parent company of Realme, and Realme. In connections a guid value and a registrationId value are sent. The registrationId changes upon a factory reset. However, the registrationId value is obtained in the response to a request to shorteux.push.heyta mobile.com that sends the device_id value, and so as noted above can be relinked to the device. The guid value does not change upon a factory reset.
- 3) The encrypted guid value and handset IMEI are sent to esa-reg-eup.myoppo.com/lmeiEncryptRegister.ashx by app com.coloros.activation. A custom reversible encryption scheme is used, and we have confirmed that the sent values can be decrypted to recover the guid and device IMEI.
- 4) Details of installed apps are sent to shorteux.push.heyta mobile.com and to icosaeu.coloros.com.
- 5) The app com.heyta.mcs registers with Google's c2dm (Cloud to Device) service, and the Firebase ID and authorization token returned by Google are sent to shorteux.push.heyta mobile.com/api/push/device/updateFcmToken. The app also registers with Google Analytics but was not observed to send data to it.
- 6) A connection to httpdnseuexpush.heyta mobile.com/getdns/v1 returns a list of IP addresses, followed by occasional TCP/MQTT connections made to one IP in the list. The MQTT message ClientId acts as a persistent identifier. The MQTT message payload is a protobuf encrypted with a custom AES algorithm, but perhaps due to a programming error does not seem to include identifiers or sensitive information.

Pre-installed Non-Realme System Apps

- 1) *Microsoft*
  - a) A connection is made to www.bing.com immediately after factory reset, in which the response sets a number of cookies. However the cookies appear to be scrubbed since they are not resent in later connections, and the cookie values in the response change.

2) *Google*

The following google connections are observed:

- a) Youtube sends device data, including device id (a unique identifier used by youtube only) and

Google gaid to youtubei.googleapis.com and www.googleadservices.com.

- b) Occasionally there are connections made to https://www.google.com/m/voice-search/up and https://www.google.com/m/voice-search/down with a persistent identifier 31f13aa1-fbb2-4302-bb02-b03bd076c118h. These connections are initiated by com.google.android.googlequicksearchbox.
- c) After logged into google account, a range requests are made to ask for the permission of google services, including googleplay, googlenow, OAuthLogin, memento, drive, mobileapps.doritos.cookie, gcm, numberer, firebase.messaging, userinfo.email, tachyon, sierra, webhistory, peopleapi.readwrite, cryptauth, playatoms, android_checkin, gmail.publisher_first_party, reminders, gmail.ads, chat, gmail.full_access, login_manager, experimentsandconfigs, userlocation.reporting, tasks, meetings, hangouts, notifications, clog.
- d) Afterwards, connections are made to sync up calendars, tasks, mails, contacts, passwords from calendarsync-pa.googleapis.com, tasks-pa.googleapis.com, inbox.google.com, people-pa.googleapis.com and chromesyncpasswords-pa.googleapis.com respectively. Note that attachments in the mail box are also downloaded.
- e) Google makes connections to get promotion text of its app, *keep note*.
- f) When location is turned on, there is a request sent to www.googleapis.com/geolocation/v1/geolocate to acquire the geolocation, most likely by the ip address of this handset, but it is unclear which pre-installed app made this request.
- g) com.google.android.googlequicksearchbox logs the activity name through app-measurement.com/a
- h) When a SIM is inserted into the handset SIM details are sent to Google as observed in previous studies, see “Mobile Handset Privacy: Measuring The Data iOS and Android Send to Apple And Google”, Proc Securecomm 2021.

A. Selected Connections During Startup After Factory Reset and When Idle

POST https://omes-sec.heytaipmobile.com/api/client/open/query-app-developer
 {"appPackage":"com.heytaip.mcs","appSign":"6e6a2cc35...c10b4","appTrusted":true,"bizNo":"1000007","sign":"dd4ba8...4c9c4870","timestamp":"1609477451274"}
 <<< HTTP 200, 150.00B

POST http://www.bing.com/
 -----hello word-----
 <0x00>...
 -----hello word-----
 <<< HTTP 200, 58.56KB
Set-Cookie: MUID=1DDCFA6A85A766FE1991EA74844D67A6; domain=.bing.com; ...0IjowLCJbXAiOjF9

Response sets cookies and sends an HTML document.

GET https://httpdns-euex-push.heytaipmobile.com/getdns/v1?region_code=IE&region_mark=EUEX&mcs_version=3306&netType=WIFI&brand=realme&deviceId=e3b0c44298fc1c14...49b934ca495991b7852b855&void=B6287A96E9D24...979CC9C51830BD62C567888AB

The deviceId value here persists across factory resets and so acts as a long-lived device identifier. The void value changes on a factory reset, but because the deviceId value remains unchanged the new void value can be relinked to the device.

POST https://adx-f.ads.heytaipmobile.com/ads/mix-frgn/V2/stg

```
1: 100
2 {
  1: "92855"
  3 {
    1: "com.oppo.launcher"
    2: "7.0.0"
    3: 7000
  }...
  1 {
    1: "1_0_"
    2: "1_0_"
    3: "1_0_"
    4: "6205256A17A34...7399b011fa3470bf8aa33"
    5: ""
    6: "Dalvik/2.1.0 (Linux; U; Android 10; RMX2063 Build/QKQ1.191222.002)"
    7: "b11da5e0-3e87-44aa-a5dc-cac792ac22d5"
    9: ""
  }
  2 {
    1: "V7.2"
    2: "RMX2063_11_A.38"
    3: "10"
  }
  3 {
    1: 2400
    2: 1080
    3: 0x40400000
  }
  4 {
    1: 0
    2: ""
  }
  5: "RMX2063"
  6: "realme"
  7: "REALME"
}
6 {
  1: "com.facebook.katana"
  2: ""
  3: 18446744073709551615
}
6 {
  1: "com.android.vending"
  2: "20.1.17-all [0] [PR] 310643216"
  3: 82011700
}
...
9: "V2"
<<< HTTP 200, 33.00B
```

POST https://www.google.com/m/voice-search/up?pair=054c6623-8224-4eb2-a00c-18e2b0b53317

POST https://youtubei.googleapis.com/deviceregistration/v1/devices?key=AlzaSyA8...2KTyQ_vz_yYM39w&rawDeviceId=f6ad0e31d43f642f
 <<< HTTP 200, 233.00B

GET https://www.google.com/complete/search?oe=utf-8&safe=images&gcc=US&ctzn=Europe/Dublin&ctf=1&v=11.28.7.21.arm64&ntyp=1&ram_mb=7577&ar=0&inm=asst&hl=en-GB&noj=1&client=qsbl-android-asbl-pb&qsubts=1618831850214&padt=200&padb=720&cads=0&psm=0&gs_per=t&q=&cp=0&psi=st3gBrQiGqE.1618831850251.0&ech=0&dpr=3.0&gs_pcr=1&xssi=t&getexp=1

GET https://app-measurement.com/config/app/1:747419181236:android:662e5d00e1b9fd34?app_instance_id=6eabae03897e35cac5bec9061add3983&platform=android&gmp_version=203315
<<< HTTP 200, 387.00B

POST https://app-measurement.com/a

```
1 {
...
13: "manual_install"
14: "com.heytag.mcs"
16: "3.3.6"
17: 16250
18: 203315
19: "b11da5e0-3e87-44aa-a5dc-cac792ac22d5"
20: 0
21: "6eabae03897e35cac5bec9061add3983"
22: 15734226478779455339
23: 1
25: "1:747419181236:android:662e5d00e1b9fd34"
28: 1
30: "ftYUbabCHWT"
31: 3306
35: 1615942156917192
46: 0
}
```

App com.heytag.mcs registering with Google Analytics.

POST https://shorteuex.push.heytagmobile.com/api/push/device/register
sign=e54c6c6...c48f06020&language=en&android_version=10&DUID=**B6287A96...C51830BD62C567888AB**&operator=data&region_mark=EUEX
&ota_version=RMX2061EU_11.A.38_0380_202103031731&
persistent_region_mark=EUEX&region_user=IE&model=RMX2063&
region_market=EUEX&brand=realme&timestamp=1618831850508&OUID=**6205256A17A34B0...399b011fa3470bf8aa33**&os_version=V7.2&
user_serial_number=0&api_version=1.0&app_key=iT4VvYW...qJw0hA1&
kernel_version=4.14.117-perf%2B&user_id=0&sign_method=md5&
rom_version=RMX2063_11.A.38&region_code=EUEX&mcs_version=3.3.6
<<< HTTP 200, 77.00B

```
{
  "code": 0,
  "data": {
    "deviceId": "607d69ebf9b995737748e8a6"
  },
  "message": "success"
}
```

Registering with Realme push service. There are two unique identifiers DUID and OUID in the request, which also appear in other connections to heytagmobile.com. The response is a device_id value 607d69ebf9b995737748e8a6 that is therefore linked to the DUID and OIUD values. It is sent in later requests.

POST https://shorteuex.push.heytagmobile.com/api/push/client/notificationBarPermission/get

The POST body in this request sends details of installed apps.

POST https://shorteuex.push.heytagmobile.com/api/push/client/register
app_key=3ZZKap...zqYpPle3X&device_id=607d69ebf9b995737748e8a6&
extra={"versionName":"3.2.40","versionCode":30040,"sdkVersion":"2.0.0","
androidVersion":29,"osVersion":"V7.2","targetValue":29}&sign_method=
md5&sign=47a73370eb...b4eac89&region_user=IE&region_market=EUEX&
api_version=1.0&brand=realme&timestamp=1618831860625&region_code=
EUEX
<<< HTTP 200, 123.00B

response:
{"code":0,"message":"success","data":{"notification":true,"registrationId":"**realme_EUEX_ee6b480ec5956ebc14814aeb57584fe5**"}}

The returned registrationId value is incorporated in later connections to ifrus-eu.coloros.com/post/Query_Update

POST https://dceuex.push.heytagmobile.com/v1/mcs/dc
event: 0F91CC357243...79294D70A9C

The event value sent in the POST body is an AES encrypted protobuf. After decrypting and decoding as a protobuf an example message is:

```
1: "GMT+00:00"
2: "EUEX"
3: "EUEX"
4: "RMX2063"
```

5: "3.3.6"

6: ""

7: ""

8 {

1: "com.oppo.ota"

2: ""

3: ""

4: ""

5: ""

7: "push_register"

8: 1618831850871

9: "{\"AppPackage\":\"com.oppo.ota\",\"TaskID\":\"\",\"StatusTime\":\"1618831850826\",\"MessageType\":\"\"}"

10: "10002"

}

8 {

1: "com.heytag.mcs"

2: ""

3: ""

4: ""

5: ""

7: "launch"

8: 1618831850277

9: "{\"launch\":\"success\"}"

10: "10001"

}

8 {

1: "com.heytag.mcs"

2: ""

3: ""

4: ""

5: ""

7: "launch"

8: 1618831850273

9: "{\"launch\":\"success\"}"

10: "10001"

}

8 {

1: "com.nearme.romupdate"

2: ""

3: ""

4: ""

5: ""

7: "push_register"

8: 1618831849258

9: "{\"AppPackage\":\"com.nearme.romupdate\",\"TaskID\":\"\",\"StatusTime\":\"1618831849230\",\"MessageType\":\"\"}"

10: "10002"

}

8 {

1: "com.nearme.romupdate"

2: ""

3: ""

4: ""

5: ""

7: "push_register"

8: 1618831849163

9: "{\"AppPackage\":\"com.nearme.romupdate\",\"TaskID\":\"\",\"StatusTime\":\"1618831849139\",\"MessageType\":\"\"}"

10: "10002"

}

9: "B6287A96E9D2...1830BD62C567888AB"

10: "6205256A1...47399b011fa3470bf8aa33"

<<< HTTP 200, 30.00B

Observe that this contains persistent identifiers, namely the DUID/VAID and OUID. The request registers with the notification push service for several pre-installed apps, including com.coloros.sau, com.oppo.ota, com.nearme.romupdate, com.coloros.lockassistant, and also logs the timestamps every time com.heytag.mcs launches.

POST https://dceuex.push.heytagmobile.com/v1/mcs/dc

1: "GMT+00:00"

2: "EUEX"

3: "EUEX"

4: "RMX2063"

5: "3.3.6"

6: ""

```

7: "607d69ebf9b995737748e8a6"
8 {
  1: "com.heyta.mcs"
  2: ""
  3: ""
  4: ""
  5: ""
  6: "launch"
  7: "1618880505049"
  8: "{ \"launch\": \"success\" }"
  9: "10001"
}
9: "B6287A96E9D2429...C9C51830BD62C567888AB"
10: "6205256A17A34B...764347399b011fa3470bf8aa33"

```

App *com.heyta.mcs* also opens a raw TCP connection to an IP address specified in the response to the request to *dceux.push.heyta.mcs.com*. Messages are transmitted over this TCP connection in MQTT format, see <https://docs.oasis-open.org/mqtt/mqtt/v5.0/mqtt-v5.0.html>. An example of the transmitted bytes is:

```

\x10\x98\x04\x00\x04MQTT\x04>\x02X\x00@
D12B4653187478DE...A96441034DB44A8BD2154D6581F177F4267\
x00@3E532B36FEB35EF228...71C16624956C8C8\
x00@F0A2086308820CACF...3492E6039E\x01\
x00700DF32F04D7E2332597DF598EB620F1C...453DD5F77\
x00@D68335F538E7AA91A78...5E72AB80836FD76DFD5C50F5\x00\x00
\x00\x00

```

Parsing this as an MQTT CONNECT request gives:

```

ClientId: D12B46531874...D6581F177F4267
Will Topic: 3E532B36FEB35E...6671C16624956C8C8
Will Message: F0A2086308820CAC...27173492E6039
User Property 1 (Metadata): 700DF32F04D7E23325...1
C58DE9AA252A453DD5F77
User Property 2 (DigitalEnvelope): D68335F538E7A...
FB265E72AB80836FD76DFD5C50F5

```

The *ClientId* value is the (likely AES) encrypted *device_id* value *607d69ebf9b995737748e8a6*. The *Topic* and *Message* values appear to be encrypted API keys/secrets (they change between messages, but apparently so does the AES key). The *Metadata* value is likely the RSA encrypted AES key, and appears to change in each message. The *DigitalEnvelope* value is an encrypted protobuf. In this example the original protobuf (before encryption) decodes as:

```

1: "GMT+00:00"
2: "EUEX"
3: "WIFI"
4: ""
5: ""
6: ""
7: ""
8: ""
9: ""
10: 1
11: "1609477264"
12: "3.3.6"
13: "RMX2063"
14: "B6287A96E9D24296924DBE8F...0BD62C567888AB"
15: "6205256A17A34B0BB654...99b011fa3470bf8aa33"

```

Here the 14 value is the DUID/OAID and the 15 value the OAID. However, before encryption the binary protobuf is first converted to a UTF-8 string and so truncated after the *WIFI* text (presumably because there is a zero byte and so the *Envelope* value sent decrypts to:

```
GMT+00:00EUEXWIFI"
```

and does not contain the DUID/OAID and OAID values. Note that encryption is carried out using an embedded JNI C library (*libheyta.mcs_cipheralgo.so*, also labelled *MCS2.0_CIPHER_ALGO*) and rather than fully reverse engineering this we used Frida to hook the API calls to extract the payload content before encryption.

POST <https://android.googleapis.com/checkin>

Sends, and links together, many device identifiers. Followed by many other connections to Google.

POST <https://icosa-eu.coloros.com/cosa/apk/info>
 guid: 14500699503cec...a01bdece856a51814ec60

POST body is a list of installed apps.

POST <https://icosa-eu.coloros.com/cosa/apk/config>

guid: 14500699503cec...997c31b0246b3a01bdece856a51814ec60
 POST body is a list of installed apps.

GET <https://icosa-eu.coloros.com/cosa/device/config>
 guid: 14500699503cec...997c31b0246b3a01bdece856a51814ec60

POST https://ifrus-eu.coloros.com/post/Query_Update
 Headers

```

user-agent: RMX2063EEA/10/V7.2/3.2.40
{"params":{"androidVersion":"Android10","colorOSVersion":"ColorOS7.2","guid":"14500699503cec...b0246b3a01bdece856a51814ec60","imei":"14500699503cec84b4bb...b3a01bdece856a51814ec60","infos":[],"language":"en-GB","mode":"0","operator":"","otaVersion":"RMX2061EU_11.A.38_0380_202103031731","productName":"RMX2063EEA","trackRegion":"EUEX","registrationId":"","realme_EUEX_ee6b480ec5956ebc14814aeb57584fe5","romVersion":"","RMX2063_11_A.38","time":1618831860692,"type":"0","uRegion":"IE","version":"3"}"}
<<< HTTP 200, 18.14KB
Followed by several similar connections.

```

POST <https://shorteux.push.heyta.mcs.com/api/push/device/update>
 sign=508a73...50ee3cf55c8&language=en&android_version=10&DUID=B6287A96E9D24...979CC9C51830BD62C567888AB&operator=data&region_mark=EUEX&ota_version=RMX2061EU_11.A.38_0380_202103031731&persistent_region_mark=EUEX&region_user=IE&model=RMX2063&region_market=EUEX&brand=realme&timestamp=1618831878817&OUID=6205256A17A34B0BB654D7305AF3A7CD8cf5be764347399b011fa3470bf8aa33&device_id=607d69ebf9b995737748e8a6&os_version=V7.2&user_serial_number=0&api_version=1.0&app_key=iT4Vv...Jw0hA1&kernel_version=4.14.117-perf+&user_id=0&sign_method=md5&rom_version=RMX2063_11_A.38&region_code=EUEX&mcs_version=3.3.6
 <<< HTTP 200, 77.00B

GET https://dl.google.com/vision/1/creditcard/params_expdata_c45e5b7.dat
 <<< HTTP 200, 130.04KB

POST <https://android.clients.google.com/c2dm/register3>

Headers

```

Authorization: AidLogin 3797961596346014448:3331276467019263121
app: com.heyta.mcs
User-Agent: Android-GCM/1.5 (RMX2063L1 QKQ1.191222.002)
X-subtype=747419181236&sender=747419181236&X-app_ver=3306&X-osv=29&X-cliv=fiid-19.0.1&X-gmsv=211213039&X-appid=ftYUbabCHWI&X-scope=*X-gmp_app_id=1:747419181236:android:662e5d00e1b9fd34&X-Firebase-Client=fire-core/17.0.0+fire-analytics/16.5.0+fire-iid/19.0.1+fire-android/&X-app_ver_name=3.3.6&app=com.heyta.mcs&device=3797961596346014448&app_ver=3306&info=Q8y7OQ7uiWEVQKri541rkWUtltyejhc&gcm_ver=211213039&plat=0&cert=555ab807ffcd...937a746&target_ver=29
<<< HTTP 200, 158.00B

```

POST <https://shorteux.push.heyta.mcs.com/api/push/device/updateFcmToken>
 app_key=iT4Vv...Jw0hA1&device_id=607d69ebf9b995737748e8a6&fcm_token=ftYUbabCHWI:APA91...jTYVSCWfUvQ&package_name=com.heyta.mcs&sign_method=md5&sign=d33d84d...ba31c9ba&type=0&api_version=1.0&timestamp=161887069779&region_code=EUEX
 <<< HTTP 200, 32.00B

App *com.heyta.mcs* registering with Google Firebase, the app *firebaseID* and the authentication token sent by google are then copied to *shorteux.push.heyta.mcs.com/api/push/device/updateFcmToken*

Additional connections when logged in to Google:

POST <https://android.googleapis.com/auth>

Headers

```

device: 34b511f30cc79af0
app: com.android.vending
User-Agent: GoogleAuth/1.4 (RMX2063L1 QKQ1.191222.002); gzip
'androidId=34b511f30cc79af0&lang=en-GB&google_play_services_version=211213039&sdk_version=29&device_country=ie&app=com.android.vending&oauth2_foreground=1&Email=doug.leith@gmail.com&token_request_options=CAA4AVAB&client_sig=38918a45...2ced5788&Token=aas...iO3nPkR6Jx0=&check_email=1&service=oauth2:https://www.googleapis.com/auth/googleplay&system_partition=1&

```

```
get_accountid=1&callerPkg=com.google.android.gms&
_opt_is_called_from_account_manager=1&is_called_from_account_manager
=1&callerSig=38918a...05ec6562ced5788'
<<< HTTP 200, 848.00
```

One of many requests that authenticate with Google services. Permission to access the following services is also requested: googleplay, googlenow, OAuthLogin, memento, drive, mobileapps.doritos.cookie, gcm, numberer, firebase.messaging, userinfo.email, tachyon, sierra, webhistory, peopleapi.readwrite, cryptauth, playatoms, android_checkin, gmail.publisher_first_party, reminders, gmail.ads, chat, gmail.full_access, login_manager, experimentsandconfigs, userlocation.reporting, tasks, meetings, hangouts, notifications, cclog.

POST <https://people-pa.googleapis.com/google.internal.people.v2.InternalPeopleService/SyncPeople>
Headers

```
x-goog-spatula: CjYKFm...wP5U9
authorization: Bearer ya29.m.Cv...AE
```

POST <https://chromesyncpasswords-pa.googleapis.com/google.internal.chrome.sync.passwords.v1.Passwords/ListPasswords>
Headers

```
x-goog-spatula: CjYKF...P5U9
authorization: Bearer ya29.m.CvkBA...AQ
```

POST <https://tasks-pa.googleapis.com/google.internal.tasks.v1.TasksApiService/Sync>
Headers

```
user-agent: Calendar-Android(versionCode=2016990472)
authorization: Bearer ya29.a0AfH...2Kw
```

POST <https://calendarsync-pa.googleapis.com/google.internal.calendar.v1.SyncService/Sync>
Headers

```
user-agent: Calendar-Android(versionCode=2016990472) x-goog-ext
-202964622-bin: Cv4CCAES+Q...5nw
authorization: Bearer ya29.a0AfH6SM...cZs
```

POST https://inbox.google.com/sync/i/s?hl=en_GB&c=1
Headers

```
x-gmail-btai: Gsw...0ji8=
user-agent: Android-Gmail/62632206 (sw360dp; 480dpi) (
RMX2063L1 QKQ1.191222.002)
authorization: Bearer ya29.a0AfH6...9A
cookie: NID=213=iFdOc...nY;
```

COMPASS=bigtop-sync=CrQBAAIriVdm9...9IFQ

After google account is logged in a series of requests are made to sync contacts, calendars, tasks and emails.

POST <https://growth-pa.googleapis.com/google.internal.identity.growth.v1.GrowthApiService/GetPromos>
Headers

```
authorization: Bearer ya29.m.CvkBAR...AQ
```

```
1 {
2 {
3: 6
2: 361705490
3 {
3: "211010340"
4: "com.google.android.keep"
5 {
6: 0x2e31322e
6: 0x30342e33302e3130
}
}
}
3 {
2 {
12: 0x42472d6e
}
3: 1
4: "29"
7 {
1: "realme"
2: "RMX2063_11_A.38"
3: "RMX2063"
}
}
```

```
}
}
4: ""
<<< HTTP 200, 1.13KB
```

POST <https://languagef-eu.coloros.com/queryUpdate/package>
Headers

```
udid: B6287A96E9D242....9CC9C51830BD62C567888AB
otaversion: RMX2061EU_11.A.38_0380_202103031731
colorosversion: V7.2
androidversion: Android10
romversion: 0
productname: RMX2063
time: 1618910477432
trackregion: EUEX
uregion: en
operator: AAAA
brand: realme
mode: 0
```

POST <https://icosa-eu.coloros.com/cosa/apk/info>
Headers

```
colorosversion: RMX2063_11_A.38
trackregion:
otaversion: RMX2061EU_11.A.38_0380_202103031731
romversion: RMX2063_11_A.38
androidversion: RMX2063_11_A.38
sign: cd1a2372cd...06a58580da
guid: 145006995...0246b3a01bdece856a51814ec60
model: RMX2063
language: en-GB
operator:
uregion:
timestamp: 1618947085546
["com.coloros.backupstore","com.google.android.youtube","com.google.android.googlequicksearchbox","com.qualcomm.qti.modemtestmode","com.android.vending","com.android.contacts","com.android.mms","com.android.stk","com.google.android.gm","com.google.android.apps.wellbeing","com.google.android.apps.maps","com.coloros.weather2","com.android.chrome","com.coloros.compass2","com.google.android.gms","com.coloros.calculator","com.topjohnwu.magisk","com.google.android.calendar","com.oppo.camera","com.google.android.documentsui","com.coloros.alarmclock","com.coloros.phonemanager","com.android.settings","com.google.android.projection.gearhead","com.oppo.music","com.coloros.video","com.coloros.filemanager","com.google.android.keep","com.coloros.soundrecorder","com.coloros.gallery3d","com.google.android.inputmethod.latin"]
```

A subset of installed apps are sent to coloros in this connection, followed by several similar ones.

POST <https://classify-eu.apps.coloros.com/api/getCategoryInfo>
'sign=1e6679...fbce6&packageName=com.android.chrome&timestamp=1618951051771'

<<<< HTTP 200, 76.00B
several app names are sent to coloros to check the category. Followed by several similar requests.

POST https://ifota-eu.realmemobile.com/post/Query_Update
Headers

```
user-agent: RMX2063EEA/10/V7.2/5065
{"params":{"version":"","otaPrefix":"","RMX2061EU":"","otaVersion":"","RMX2061EU_11.A.38_0380_202103031731","imei":"","14500699503cec84b4bba6b...ece856a51814ec60","mode":0,"productName":"","RMX2063EEA","language":"en-GB","type":"1","isRooted":"1","canCheckSelf":"0","time":1618937643875,"romVersion":"","RMX2063_11_A.38","androidVersion":"","Android10","colorOSVersion":"","ColorOS7.2","isOnePlus":0,"registrationId":"","realme_EUEX_ee6b480ec5956ebc14814aeb57584fe","trackRegion":"","EUEX","uRegion":"","IE","guid":"","14500699503cec8...e856a51814ec60","securityPatch":"","2021-02-05","securityPatchVendor":"","2021-02-05","nvCarrier":"","01000100","partCarrier":"","01000100","modules":[{"otaPkgType":"","1","version":"","RMX2061EU_11.A.38_0380_202103031731"}]}
,"version":"","4"}
<<< HTTP 200, 155.00B
```

POST <https://esa-reg-eup.myoppo.com/IndexService.ashx>

'authorKey=94e86941...47c65a&appKey=smartphoneregisterhttps'
<<< HTTP 200, 60.00B

POST https://esa-reg-eup.myoppo.com/**ImeiEncryptRegister.ashx**
openid=A%1Ci%60%3CV%11UE%18g%13q%03%10Pr%1C%16%12mV%3ATB%20m%14DY%11UGKganP%0APFJg%11%3CQ%3A%00uK%19hAV9QA%20edq%03%0EL&authorKey=6eae50358d3fd...c1592&imei=eee%5Eeah%5Dh%5E%5Dgad&activation=y'
<<< HTTP 200, 35.00B
This connection is made by app com.coloros.activation. The POST body is URL encoded. When decoded, the openid and imei values are encrypted using a custom reversible cipher. The openid value URL decodes to Ai'(VUEgqPrmV:TBmDYUGKganP.PFJg(Q:uKhAV9QAedqL, and this then decrypts to 14500699503cec84b4bba6b8289d89997c31b0246b3a01bdece856a51814ec60 i.e. the guid (a persistent device identifier). The imei value URL decodes to eee^eah[h]^gad which decrypts to 867331040902513 i.e. the IMEI of the first SIM slot on the handset (a unique persistent device identifier).

GET https://confe.dc.oppomobile.com/v1/conf/dcs?appid=21000&logtag=0&nonce=10940&timestamp=1618996349&sign=07bc9c...0643360e8&region=27211,27205&checksum=b26090...5e02a4f
The mobile operator MCC and MNC are sent in this request.

Additional connections when location enabled

POST https://www.googleapis.com/geolocation/v1/geolocate?key=AIZAyAP...mXHHzIk
Headers
user-agent: com.google.android.gms/211213039 (Linux; U; Android 10; en_GB; RMX2063; Build/QKQ1.191222.002; Cronet/85.0.4183.127)
'{"considerIp":true}'
<<< HTTP 200, 89.00B
{
 "location": {
 "lat": 53.3004288,
 "lng": -6.422528
 },
 "accuracy": 14340
}
Google may use the IP address to determine the geolocation. See the API: <https://developers.google.com/maps/documentation/geolocation/overview>.
The returned GPS coordinates are within about 5-10Km of the true handset location.

POST https://www.googleapis.com/socialuserlocation/v1/userLocationFrontend/readsharingstate
Authorization: OAuth ya29.m.Cv4DARM...gIIAQ
X-Goog-Spatula: CjYKFmNvbS5nb29nbG...X5wP5U9
Accept-Encoding: gzip
User-Agent: GmsCore/211213039 (RMX2063L1 QKQ1.191222.002);
gzip

3 {
 2 {
 1: 2
 2: 560
 }
}

Connections When Insert Sim

POST https://android.clients.google.com/fdfe/uploadDynamicConfig
Headers
user-agent: Android-Finsky/24.9.17-21%20%5B0%5D%20%5BPR%5D%20367727371 (api=3,versionCode=82491710, sdk=29, device=RMX2063L1, hardware=qcom, product=RMX2063EEA, platformVersionRelease=10, model=RMX2063, buildId=QKQ1.191222.002, isWideScreen=0, supportedAbis=arm64-v8a;armeabi-v7a;armeabi)
x-dfe-device-id: 34b511f30cc79af0
x-dfe-device-config-token: CisaKQoTMz...M1NjE0MDUyMzM1
x-dfe-device-checkin-consistency-token: ABFET...m6mv5hEbCB
x-dfe-mccmnc: 27211
x-dfe-client-id: am-android-oppo
x-dfe-phenotype: H4sIAAA...Z-WeTDf8_nPj_8D8RYEmQEAAA

x-dfe-encoded-targets: CAESBPYigQY...HO4sv3yI
x-dfe-build-fingerprint: realme/RMX2063EEA/RMX2063L1:10/QKQ1.191222.002/1610628567:user/release-keys
'1 {
 1: "GMT+00:00"
 2 {
 1 {
 1: 272110103800000
 2: "Tesco Mobile"
 3: "0AFFFFFFFFFFFFFFFFFFFFFFFFFFFFFFFFFFFFFFFF"
 6: 2154
 7: 18446744073709551615
 }
 }
 4: "ddt6CEm...uSzN_L"
}
<<< HTTP 200, 102.00B
MCC, MNC and the operator's name are sent to Google when the sim card is inserted.

POST https://android.googleapis.com/checkin
Google logs the SIM details together with a collection of device identifiers.

B. Connections When Interacting With Settings App

POST https://confe.dc.oppomobile.com/v1/conf/key?appid=21000&logtag=0&nonce=5802&timestamp=1618908719&sign=8be0239cb8fc7042...20535
GET https://confe.dc.oppomobile.com/v1/conf/dcs?appid=21000&logtag=0&nonce=10009&timestamp=1618908719&sign=372afb2e0...313d&region=27211,27205&checksum=
GET https://ifota-eu.coloros.com/post/
POST https://ifota-eu.realmemobile.com/post/Query_Update
https://ifota-eu.realmemobile.com/post/Query_Description

https://pagead2.googlesyndication.com/pagead/gen_204?id=gmob-apps&action=ads_settings_page_view&device=realme RMX2063&js=211213039.211213000&os=10&api=29&eids=318497135,318475417,318489659,318495357,318495359,318494842&appid=com.google.android.gms

V. PIXEL 2 LINEAGEOS

Summary:

- 1) The handset is considerably “quieter” than the Samsung, Xiaomi, Realme and Huawei handsets. A single connection to download.lineageos.org sends device details but contains no identifiers. The remaining connections are associated with Google system apps, which send a similar data content to the Google apps on the Samsung, Xiaomi, Realme and Huawei handsets.

Pre-installed Non-LineageOS System Apps

1) Google

- a) Google Play Services and Google Play store make many connections to Google servers. These share persistent device and user identifiers with Google including the device hardware serial number, SIM IMEI, Wifi MAC address, SIM IMSI and phone number, user email (when logged in). A substantial quantity of data is sent, in particular, to play.googleapis.com/play/log and www.googleapis.com/experimentsandconfigs (but not to play.googleapis.com/vn/log/batch, unlike on the other handsets studied).
- b) Periodic connections are made to www.google.com/m/voice-search with a unique identifier in the url path. Less frequent connections are made to www.google.com/complete/search in which the headers incorporate a field: *x-client-data*. These are associated with the com.google.android.googlequicksearchbox searchbar app. The com.google.android.googlequicksearchbox app also sends telemetry data to Google Analytics.
- c) When logged in to a Google account, connections are made to mail.google.com/mail/ads, inbox.google.com/sync and www.googleapis.com/calendar that send identifiers linked to the device and user account. Note that account login was carried out via the Google Play app only. Syncing of gmail, contacts, calendar took place without the user being asked or opting in. In addition, the following services are authenticated: gms, googlenow, OAuthLogin, mobileapps.doritos.cookie, experimentsandconfigs, numberer, firebase.messaging, googleplay, web-history, tachyon, reminders, userlocation.reporting, android_checkin, peopleapi.legacy.readwrite, cryptauth, login_manager, playatoms, peopleapi.readonly, gcm, notifications, calendar.
- d) Google Chrome makes connections to Google servers. These connections are consistent with previously documented behaviour, see “Web Browser Privacy: What Do Browsers Say When They Phone Home?”, IEEE Access. DOI 10.1109/ACCESS.2021.3065243.
- e) When a SIM is inserted into the handset SIM details are sent to Google as observed in previous studies, see “Mobile Handset Privacy: Measuring The Data

iOS and Android Send to Apple And Google”, Proc Securecomm 2021.

A. Selected Connections During Startup After Factory Reset (Incl Idle)

GET https://download.lineageos.org/api/v1/walleye/nightly/87d0b05a87
<<< HTTP 200, 1.57KB

POST https://android.googleapis.com/checkin
This checkin connection as been documented elsewhere. It links together several device identifiers including the IMEI, hardware serial number and Wifi MAC address, and sends extensive details of the device hardware and software to Google.

POST https://android.clients.google.com/fdfe/uploadDeviceConfig
Headers

User-Agent: Android-Finsky/24.4.23-21%20%5B0%5D%20%5BPR%5D%20361682659 (api=3,versionCode=82442310, sdk=29,device=walleye, hardware=walleye,product=walleye,platformVersionRelease=10,model=Pixel %202,buidId=QQ3A.200805.001,isWideScreen=0,supportedAbis=arm64-v8a;armeabi-v7a;armeabi)

X-DFE-MCCMNC: 27211
X-DFE-Client-Id: am-android-google
X-DFE-Phenotype: H4sIAAAAAA...PTxAgAAAA
X-DFE-Build-Fingerprint: google/walleye/walleye:10/QQ3A.200805.001/6578210:user/release-keys

POST body contains details of handset hardware and software.

POST https://android.clients.google.com/c2dm/register3

Headers

Authorization: AidLogin 4227391861695796777:3228209926479926894
app: com.android.vending

gcm_ver: 210613064

User-Agent: Android-GCM/1.5 (walleye QQ3A.200805.001)

X-subtype=932144863878&sender=932144863878&X-app_ver=82442310&X-osv=29&X-cliv=fiid-21.1.0&X-gmsv=210613064&X-appid=ehR5AzhTg61151HHDn5RF&X-scope=&X-Goog-Firebase-Installations-Auth=eyJhbGciOiJIUzI1NiIsInR5cGE6YWV0IjoiPTxAgAAAAA221571841318%3AAndroid%3A9c547b5ed466b580&X-firebase-app-name-hash=R1dAH9Ui7M-ynoznwBdw01tLxhI&X-app_ver_name=24.4.23-21+%5B0%5D+%5BPR%5D+361682659&app=com.android.vending&device=4227391861695796777&app_ver=82442310&gcm_ver=210613064&plat=0&cert=38918a453...d5788&target_ver=29
Google Play store app com.android.vending registering with Firebase. Similar connections are made by com.google.android.wfcactivation, com.google.android.gms.

POST https://firebaseinstallations.googleapis.com/v1/projects/google.com:api--project=1086610230652/installations

Headers

Cache-Control: no-cache
X-Android-Package: com.google.android.googlequicksearchbox
x-firebase-client: fire-core/19.3.2_1p fire-android/ fire-installations/16.3.6_1p fire-analytics/18.0.3 fire-iid/21.1.0
x-firebase-client-log-type: 3
x-goog-api-key: AlzaSyC...1K-n7Uby
{“fid”:“dULX8HN4QyO5KSJgliewdW”,“appId”:“1:1086610230652:android:131e4c3db28fca84”,“authVersion”:“FIS_v2”,“sdkVersion”:“a:16.3.6_1p”}
<<< HTTP 200, 577.00B

POST https://www.google.com/m/voice-search/up?pair=7a6e1c58-b4b1-4e30-a2b1-e8c7f7ae4c74

Headers

x-device-elapsed-time: 999955414351

POST https://www.google.com/m/voice-search/down?pair=7a6e1c58-b4b1-4e30-a2b1-e8c7f7ae4c74

Headers

x-device-elapsed-time: 1000002459616

POST https://app-measurement.com/a

com.google.android.googlequicksearchbox logs the activity name through app-measurement.com. The POST body decodes, for example, to:

```
body {
  always_one: 1
  event {
    event_info {
      setting_code: "_o" // firebase_event_origin
      data_str: "auto"
    }
    event_info {
      setting_code: "_sid" // session_id
      data_int: 1616140977
    }
    event_info {
      setting_code: "_sno" // session_number
      data_int: 1
    }
    event_info {
      setting_code: "_c" // firebase_conversion
      data_int: 1
    }
    event_info {
      setting_code: "_r" // realtime
      data_int: 1
    }
    event_code: "_s" // session_start
    event_timestamp: 1616140977961
  }
  event {
    event_info {
      setting_code: "_o" // firebase_event_origin
      data_str: "auto"
    }
    event_info {
      setting_code: "_sc" // firebase_screen_class
      data_str: "SearchNowActivity"
    }
    event_info {
      setting_code: "_si" // firebase_screen_id
      data_int: 6679454159444222111
    }
    event_info {
      setting_code: "_efs"
      data_int: 1
    }
    event_info {
      setting_code: "_sr"
      data_int: 10
    }
    event_code: "_vs" // screen_view
    event_timestamp: 1616140978025
  }
  event {
    event_info {
      setting_code: "_o" // firebase_event_origin
      data_str: "auto"
    }
    event_info {
      setting_code: "_et" // engagement_time_msec
      data_int: 1017
    }
    event_info {
      setting_code: "_sc" // firebase_screen_class
      data_str: "SearchNowActivity"
    }
    event_info {
      setting_code: "_si" // firebase_screen_id
      data_int: 6679454159444222111
    }
    event_info {
      setting_code: "_fr"
      data_int: 1
    }
    event_info {
      setting_code: "_efs"
      data_int: 1
    }
  }
  event_info {
    setting_code: "_sr"
    data_int: 10
  }
  event_code: "_e" // user_engagement
  event_timestamp: 1616140978973
  previous_event_timestamp: 1616107342777
}
user {
  timestamp: 1616107342777
  setting: "_fot"
  data_int: 1616108400000
}
user {
  timestamp: 1616107342777
  setting: "_fi" // first_install
  data_int: 1
}
user {
  timestamp: 1616140988577
  setting: "_lte" // lifetime_engagement
  data_int: 1018
}
user {
  timestamp: 1616140977961
  setting: "_sno" // session_number
  data_int: 1
}
user {
  timestamp: 1616140977961
  setting: "_sid" // session_id
  data_int: 1616140977
}
user {
  timestamp: 1616140988579
  setting: "_se" // session_scoped_engagement
  data_int: 1017
}
message_timestamp: 1616140988568
event_timestamp: 1616140977961
bundle_end_timestamp: 1616140979045
last_bundle_end_timestamp: 1616107342777
operating_system: "android"
operating_system_version: "10"
Build_MODEL: "Pixel 2"
language_country: "en-ie"
app_store: "manual_install"
package_name: "com.google.android.googlequicksearchbox"
app_version: "12.6.13.29.arm64"
gmp_version: 39015
gms_version: 210613
google_ad_id: "5d513fb7-b415-4aa4-8293-17d7666c602e"
random_hex: "a9d3491048ababf08bfb778e00ac5dea"
dev_cert_hash: 12815780039134672363
daily_conversions_count: 2
gmp_app_id: "1:1086610230652:android:131e4c3db28fca84"
last_bundle_end_timestamp2: 1616107342777
always_true: true
filter_list {
  filter_id: 4
  c {
    1: 1
    2: 1
  }
  e: true
}
filter_list {
  filter_id: 12
  c {
    1: 1
  }
  d {
    1: 1
  }
}
filter_list {
  filter_id: 29
}
```

```

c {
  1: 2
  2: 2
  3 {
    1: 1
    2: 1616140977
  }
}
e: true
}
filter_list {
  filter_id: 76
  c {
    1: 2
  }
  e: true
}
firebase_instance_id: "dULX8HN4QyO5KSJgliewdW"
app_version_int: 301132231
config_version: 1606952846514987
M: 23180913
dynamite_version: 46
unknown: "G1—"
}
<<< HTTP 204, 0.00B

```

GET https://www.google.com/complete/search?oe=utf-8&safe=images&gcc=ie&ctzn=Europe/Dublin&ctf=1&v=12.6.13.29.arm64&ntyp=1&ram_mb=3651&ar=0&inm=asst&hl=en-IE&noj=1&client=qsab-android-asbl-pb&qsubts=1616112840286&padt=200&padb=684&cs=0&cds=2&psm=0&gs_pcr=t&q=cp=0&psi=e-Yle9rXBRI.1616112840303.0&ech=0&dpr=2.625&gs_pcr=1&xssi=t&getexp=1

Headers
 x-client-data: aos...CgAA
 cookie: CONSENT=PENDING+515

GET http://xtrapath2.izatcloud.net/xtra3grcej.bin

<<< HTTP 200, 46.79KB

This connection is to a qualcom server to fetch a config file associated with assisted GPS.

POST https://auditrecording-pa.googleapis.com/google.internal.api.auditrecording.v1.AuditRecordingMobileService/CreateAuditRecord

Headers
 te: trailers
 x-goog-spatula: CjYKFmNvbS5n...cn+B/g
 POST payload is opaque protobuf.

POST https://infinitedata-pa.googleapis.com/mdi.InfiniteData/Lookup

Headers
 x-goog-api-key: AIzaSyAP-gfH3qvi6...mXHhzIk
 x-android-package: com.google.android.gms
 x-android-cert: 38918A45...C6562CED5788

POST body includes details of installed apps.

GET https://android.clients.google.com/fdfe/selfUpdate?ex=1&susp=EAAAYASABMAE%3D

Headers
 User-Agent: Android-Finsky/24.4.23-21%20%5B0%5D%20%5BPR%5D%20361682659 (api=3,versionCode=82442310,sdk=29,device=walleye,hardware=walleye,product=walleye,platformVersionRelease=10,model=Pixel%202,buildId=QQ3A.200805.001,isWideScreen=0,supportedAbis=arm64-v8a;armeabi-v7a;armeabi)
 X-DFE-Device-Id: **3aaab68022c15629**
 X-DFE-Device-Config-Token: CisaKQoTNDI...OTQ1NDQ4ODc5
 X-DFE-MCCMNC: 27211
 X-DFE-Client-Id: am-android-google
 X-DFE-UserLanguages: en-IE
 X-DFE-Phenotype: H4sIAAAAAA...r3e6wAAAA
 X-DFE-Encoded-Targets: CAESBomBgQbz...QbwAaUB4gRd
 google/walleye/walleye:10/QQ3A.200805.001/6578210:user/release-keys
 <<< HTTP 200, 1.37KB

Connections When Insert Sim

POST https://android.clients.google.com/fdfe/uploadDynamicConfig

Headers
 user-agent: Android-Finsky/24.4.23-21%20%5B0%5D%20%5BPR%5D%20361682659 (api=3,versionCode=82442310,sdk=29,device=walleye,hardware=walleye,product=walleye,platformVersionRelease=10,model=Pixel%202,buildId=QQ3A.200805.001,isWideScreen=0,supportedAbis=arm64-v8a;armeabi-v7a;armeabi)
 x-dfe-device-id: **3aaab68022c15629**
 x-dfe-device-config-token: CisaKQ..EyMzk5
 x-dfe-device-checkin-consistency-token: ABF...r2
 x-dfe-network-type: 4
 x-dfe-mccmnc: 27211
 x-dfe-client-id: am-android-google
 x-dfe-phenotype: H4s...AA
 x-dfe-encoded-targets: CAESBo...vBg
 x-dfe-build-fingerprint: google/walleye/walleye:10/QQ3A.200805.001/6578210:user/release-keys
 POST body decoded as protobuf:

```

1 {
  1: "GMT+00:00"
  2 {
    1 {
      1: 272110103800000
      2: "Tesco Mobile"
      3: "0AFFFFFFFFFFFFFFFFFFFFFFFFFFFFFFFFFFFFFFFF"
      4: "tescomobile.liffeytelecom.com"
      6: 2154
      7: 18446744073709551615
    }
  }
  4: "ehrRA5zhTg...1MoJ"
}

```

<<< HTTP 200, 102.00B

Mobile operator, mcc and mnc sent to google. Similar connections observed in other other handsets.

Additional connections when logged in to Google:

POST https://android.googleapis.com/auth

Headers
 device: **3aaab68022c15629**
 app: com.google.android.gms
 User-Agent: GoogleAuth/1.4 (walleye QQ3A.200805.001); gzip
 androidId=3aaab68022c15629&lang=en-IE&google_play_services_version=210613064&sdk_version=29&device_country=ie&Email=**doug.leith@gmail.com**&build_product=walleye&build_brand=google&Token=oauth2_4/0AY0e-g4PjuW0laB...vCp6iA4nQYxrpA&build_fingerprint=google/walleye/walleye:10/QQ3A.200805.001/6578210:user/release-keys&build_device=walleye&service=ac2dm&get_accountid=1&ACCESS_TOKEN=1&callerPkg=com.google.android.gms&add_account=1&droidguard_results=CgYGY7UPSE..ASIA&callerSig=38918a45...62ced5788
 <<< HTTP 200, 800.00B

The first of a sequence of authentication requests to google services. In total, the following services are authenticated: gms, googlenow, OAuthLogin, mobileapps.doritos.cookie, experimentsandconfigs, numberer, firebase.messaging, googleplay, webhistory, tachyon, reminders, userlocation.reporting, android_checkin, peopleapi.legacy.readwrite, cryptauth, login_manager, playatoms, peopleapi.readonly, gcm, notifications, calendar.

POST https://people-pa.googleapis.com/google.internal.people.v2.

InternalPeopleService/SyncPeople
 Headers
 te: trailers
 x-goog-spatula: CjYKFm...cn+B/g
 authorization: Bearer **ya29.m.Cvo..CCA**

POST https://chromesyncpasswords-pa.googleapis.com/google.internal.chrome.sync.passwords.v1.Passwords/ListPasswords

Headers
 x-goog-spatula: CjYKFm...cn+B/g
 authorization: Bearer **ya29.m....IAQ**

GET https://www.googleapis.com/calendar/v3/internal/calendars/en.irish#holiday@group.v.calendar.google.com/events?maxAttendees=50&maxResults=200&supportsAllDayReminders=true&timeMax=2022-04-28T00:00:00.000Z&timeMin=2020-03-19T16:13:43.000Z

Headers

Authorization: OAuth ya29.a0AfH...MeIQ

User-Agent: google/walleye/walleye:10/QQ3A.200805.001/6578210:user/release-keys:com.google.android.syncadapters.calendar:2016267990:release
Google-HTTP-Java-Client/1.26.0-SNAPSHOT (gzip)

x-goog-api-client: java/0 http-google-calendar/1.26.0 linux/4.4.210

<<< HTTP 200, 28.57KB

Connections are made to sync up contacts, password and calendars.

Additional connections when location enabled

POST https://android.googleapis.com/auth

Headers

device: **3aaab68022c15629**

app: com.google.android.gms

User-Agent: GoogleAuth/1.4 (walleye QQ3A.200805.001); gzip
androidId=**3aaab68022c15629**&lang=en-IE&google_play_services_version=210613064&sdk_version=29&device_country=ie&it_caveat_types=2&app=com.google.android.gms&oauth2_foreground=1&Email=**doug.leith@gmail.com**&token_request_options=CAA4AVAB&client_sig=38918a45...2ced5788&Token=aas_et%2FAKppINa__vyzJEOj...Kom8ItG1JMs%3D&check_email=1&service=oauth2%3Ahttps%3A%2F%2Fwww.googleapis.com%2Fauth%2Fsocial.userlocation&system_partition=1&callerPkg=com.google.android.gms&request_visible_actions=&callerSig=38918a453d...ced5788

<<< HTTP 200, 587.00B

POST https://www.googleapis.com/socialuserlocation/v1/userLocationFrontend/readsharingstate

Headers

Authorization: OAuth ya29.m.CvkBARMX...JQTVa-7yYCIgHIAQ

X-Goog-Spatula: **CjYKFmNvbS5nb...aOdcn+B/g**

User-Agent: GmsCore/210613064 (walleye QQ3A.200805.001); gzip

POST body decoded as protobuf:

```
3 {
  2 {
    1: 2
    2: 560
  }
}
```

VI. PIXEL 2 EOS

Summary

- 1) The only connection made during startup after a factory reset is to `xtrapath3.izatcloud.net` to download a GPS configuration file from qualcom, no identifiers are sent.
- 2) When navigating the Settings app a slimstat cookie is set when the privacy policy page is viewed. This seems to be associated with fetching of web page `https://e.foundation/legal-notice-privacy/`. The privacy policy at that web page says “We use Slimstat to create visitors statistics for our internal use”.
- 3) No connections were observed to Google servers.

A. Connections During Startup After Fresh Install & Idle

GET `http://xtrapath3.izatcloud.net/xtra3grcej.bin`

<<< HTTP 200, 46.58KB

This connection is to a qualcom server to fetch a config file associated with assisted GPS.

B. Connections When Interacting With Settings App

POST `https://e.foundation/wp-admin/admin-ajax.php`

Headers

DNT: 1

X-Requested-With: XMLHttpRequest

Origin: `https://e.foundation`

Referer: `https://e.foundation/legal-notice-privacy/`

`action=slimtrack&ref=&res=aHR0cHM....ab65149de9&sw=732&sh=412&bw=412&bh=604&sl=720&pp=1566&fh=`

`888bc904532fc607aa9287e69dde0412&tz=0`

<<< HTTP 200, 39.00B

Set-Cookie: `slimstat_tracking_code=354778.4dbaef4f1780d5ff46d24efdd7dc273; expires=Sat, 20-Mar-2021 05:57:13 GMT; Max-Age=1800; path=/`

The response to this connection sets a cookie. There are several id-like strings in the posted content, but they are used in a one-off manner and not found elsewhere.

GET `https://e.foundation/wp-content/themes/page-builder-framework/fonts/page-builder-framework.woff2`

Headers

DNT: 1

Referer: `https://e.foundation/wp-content/themes/page-builder-framework/style.css?ver=2.2`

Cookie:

`slimstat_tracking_code=354778.4dbaef4f1780d5ff46d24efdd7dc273`

VII. ESTIMATED SERVER LOCATIONS

Below is the mapping from IP address to location using <https://ipinfo.io/> service. With the exception of capi.samsungcloud.com, the servers estimated to be located in the US by <https://ipinfo.io/> are in fact located in Europe based on ping times/traceroute (which are under 40ms from Ireland). The ping times from Ireland to the servers estimated by <https://ipinfo.io/> to be located in Singapore are around 270ms.

A. Samsung Handset

74.125.193.113, android.clients.google.com, IE, Leinster, Dublin, 53.3331,-6.2489
 193.155.127.31, fota-apis.samsungdm.com, DE, Hesse, Herborn, 50.6814,8.3037
 213.71.30.150, www.ospserver.net, DE, Hesse, Frankfurt am Main, 50.1155,8.6842
 87.248.214.69, fota-cloud-dn.ospserver.net, GB, England, London, 51.5085,-0.1257
 74.125.193.95, play.googleapis.com, IE, Leinster, Dublin, 53.3331,-6.2489
 54.74.219.194, sspapi-prd.samsungrs.com, IE, Leinster, Dublin, 53.3331,-6.2489
 74.125.193.104, www.google.com, IE, Leinster, Dublin, 53.3331,-6.2489
 74.125.193.94, www.gstatic.com, IE, Leinster, Dublin, 53.3331,-6.2489
 209.85.202.95, play.googleapis.com, US, California, Mountain View, 38.0088,-122.1175
 74.125.193.99, www.google.com, IE, Leinster, Dublin, 53.3331,-6.2489
 213.71.30.137, dms.ospserver.net, DE, Hesse, Frankfurt am Main, 50.1155,8.6842
 209.85.203.94, connectivitycheck.gstatic.com, US, California, Mountain View, 38.0088,-122.1175
 74.125.193.103, www.google.com, IE, Leinster, Dublin, 53.3331,-6.2489
 74.125.193.102, android.clients.google.com, IE, Leinster, Dublin, 53.3331,-6.2489
 52.51.246.198, vas.samsungapps.com, IE, Leinster, Dublin, 53.3331,-6.2489
 52.213.86.174, sdk.pushmessage.samsung.com, IE, Leinster, Dublin, 53.3331,-6.2489
 74.125.193.147, www.google.com, IE, Leinster, Dublin, 53.3331,-6.2489
 74.125.193.105, www.google.com, IE, Leinster, Dublin, 53.3331,-6.2489
 74.125.193.139, app-measurement.com, IE, Leinster, Dublin, 53.3331,-6.2489
 74.125.193.138, app-measurement.com, IE, Leinster, Dublin, 53.3331,-6.2489
 74.125.193.106, www.google.com, IE, Leinster, Dublin, 53.3331,-6.2489
 172.253.116.95, mdh-pa.googleapis.com, IE, Leinster, Dublin, 53.3331,-6.2489
 52.211.221.138, vas.samsungapps.com, IE, Leinster, Dublin, 53.3331,-6.2489
 99.80.143.231, gos-api.gos-gsp.io, IE, Leinster, Dublin, 53.3331,-6.2489
 209.85.203.95, play.googleapis.com, US, California, Mountain View, 38.0088,-122.1175
 74.125.193.100, android.clients.google.com, IE, Leinster, Dublin, 53.3331,-6.2489
 99.86.125.158, pinning-02.sec2b2b.com, IE, Leinster, Dublin, 53.3331,-6.2489
 52.210.187.74, eu-kaf.samsungknox.com, IE, Leinster, Dublin, 53.3331,-6.2489
 54.194.177.241, vas.samsungapps.com, IE, Leinster, Dublin, 53.3331,-6.2489
 151.101.36.223, www.python.org, NL, North Holland, Amsterdam, 52.3740,4.8897
 54.72.236.3, gos-api.gos-gsp.io, IE, Leinster, Dublin, 53.3331,-6.2489
 104.26.9.126, 104.26.9.126, US, California, San Francisco, 37.7621,-122.3971
 209.85.203.100, android.clients.google.com, US, California, Mountain View, 38.0088,-122.1175
 34.249.76.93, dir-apis.samsungdm.com, IE, Leinster, Dublin, 53.3331,-6.2489
 74.125.193.157, www.googleadservices.com, IE, Leinster, Dublin, 53.3331,-6.2489
 209.85.202.190, www.youtube.com, US, California, Mountain View, 38.0088,-122.1175
 104.17.89.51, www.change.org, US, California, San Francisco, 37.7621,-122.3971

13.107.42.23, config.edge.skype.com, US, Washington, Redmond, 47.6740,-122.1215
 92.123.125.101, oneclient.sfx.ms, NL, North Holland, Amsterdam, 52.3740,4.8897
 13.88.28.53, mobile.pipe.aria.microsoft.com, US, California, San Jose, 37.3476,-121.8870
 74.125.193.91, www.youtube.com, IE, Leinster, Dublin, 53.3331,-6.2489
 52.211.89.231, vas.samsungapps.com, IE, Leinster, Dublin, 53.3331,-6.2489
 52.19.221.59, gos-api.gos-gsp.io, IE, Leinster, Dublin, 53.3331,-6.2489
 34.247.17.140, ie-odc.samsungapps.com, IE, Leinster, Dublin, 53.3331,-6.2489
 185.151.204.12, app.adjust.com, DE, Berlin, Berlin, 52.5352,13.4257
 13.105.66.144, skyapi.live.net, NL, North Holland, Amsterdam, 52.3740,4.8897
 128.30.52.100, www.w3.org, US, Massachusetts, Cambridge, 42.3751,-71.1056
 52.50.196.133, vas.samsungapps.com, IE, Leinster, Dublin, 53.3331,-6.2489
 52.13.204.79, capi.samsungcloud.com, US, Oregon, Boardman, 45.8399,-119.7006

B. Xiaomi Handset

74.125.193.94, www.gstatic.com, IE, Leinster, Dublin, 53.3331,-6.2489
 74.125.193.95, play.googleapis.com, IE, Leinster, Dublin, 53.3331,-6.2489
 47.241.69.47, api.ad.intl.xiaomi.com, SG, Singapore, Singapore, 1.2897,103.8501
 74.125.193.102, app-measurement.com, IE, Leinster, Dublin, 53.3331,-6.2489
 47.241.67.215, api.ad.intl.xiaomi.com, SG, Singapore, Singapore, 1.2897,103.8501
 74.125.193.132, lh3.googleusercontent.com, IE, Leinster, Dublin, 53.3331,-6.2489
 74.125.90.100, www.google.com, US, California, Mountain View, 38.0088,-122.1175
 209.85.203.94, connectivitycheck.gstatic.com, US, California, Mountain View, 38.0088,-122.1175
 209.85.203.190, android-safebrowsing.google.com, US, California, Mountain View, 38.0088,-122.1175
 209.85.202.94, connectivitycheck.gstatic.com, US, California, Mountain View, 38.0088,-122.1175
 74.125.193.91, dl.google.com, IE, Leinster, Dublin, 53.3331,-6.2489
 74.125.193.103, www.google.com, IE, Leinster, Dublin, 53.3331,-6.2489
 47.241.108.144, api.ad.intl.xiaomi.com, SG, Singapore, Singapore, 1.2897,103.8501
 74.125.193.104, www.google.com, IE, Leinster, Dublin, 53.3331,-6.2489
 74.125.193.101, app-measurement.com, IE, Leinster, Dublin, 53.3331,-6.2489
 47.241.28.162, api.ad.intl.xiaomi.com, SG, Singapore, Singapore, 1.2897,103.8501
 161.117.71.226, sdkconfig.ad.intl.xiaomi.com, SG, Singapore, Singapore, 1.2897,103.8501
 163.181.57.226, f4.market.xiaomi.com, GB, England, London, 51.5085,-0.1257
 74.125.193.93, android-safebrowsing.google.com, IE, Leinster, Dublin, 53.3331,-6.2489
 47.88.231.18, sdkconfig.ad.xiaomi.com, SG, Singapore, Singapore, 1.2897,103.8501
 47.241.9.240, api.ad.intl.xiaomi.com, SG, Singapore, Singapore, 1.2897,103.8501
 172.217.171.4, www.google.com, IE, Leinster, Dublin, 53.3331,-6.2489
 74.125.193.113, android.clients.google.com, IE, Leinster, Dublin, 53.3331,-6.2489
 173.194.129.230, r1---sn-q0c7m76.gvt1.com, IE, Leinster, Dublin, 53.3331,-6.2489
 173.194.129.233, r4---sn-q0c7m76.gvt1.com, IE, Leinster, Dublin, 53.3331,-6.2489
 74.125.168.8, r3---sn-q0cedn7s.gvt1.com, IE, Leinster, Dublin, 53.3331,-6.2489
 74.125.193.147, www.google.com, IE, Leinster, Dublin, 53.3331,-6.2489
 74.125.193.139, app-measurement.com, IE, Leinster, Dublin, 53.3331,-6.2489
 172.253.116.103, www.google.com, IE, Leinster, Dublin, 53.3331,-6.2489
 172.253.116.95, www.googleapis.com, IE, Leinster, Dublin, 53.3331,-6.2489

47.74.223.198, mcc.intl.inf.miui.com, SG, Singapore, Singapore,
1.2897,103.8501
161.117.183.182, api.ad.intl.xiaomi.com, SG, Singapore, Singapore,
1.2897,103.8501
173.194.129.234, r5---sn-q0c7rn76.gvt1.com, IE, Leinster, Dublin,
53.3331,-6.2489
173.194.129.232, r3---sn-q0c7rn76.gvt1.com, IE, Leinster, Dublin,
53.3331,-6.2489
74.125.168.9, r4---sn-q0cedn7s.gvt1.com, IE, Leinster, Dublin,
53.3331,-6.2489
74.125.193.138, android.clients.google.com, IE, Leinster, Dublin,
53.3331,-6.2489
47.241.124.98, api.ad.intl.xiaomi.com, SG, Singapore, Singapore,
1.2897,103.8501
47.241.125.132, api.ad.intl.xiaomi.com, SG, Singapore, Singapore,
1.2897,103.8501
74.125.90.68, www.google.com, US, California, Mountain View,
38.0088,-122.1175
161.117.190.253, tracking.intl.miui.com, SG, Singapore, Singapore,
1.2897,103.8501
161.117.97.83, data.mistat.intl.xiaomi.com, SG, Singapore, Singapore,
1.2897,103.8501
74.125.193.105, www.google.com, IE, Leinster, Dublin, 53.3331,-6.2489
47.241.36.250, api.ad.intl.xiaomi.com, SG, Singapore, Singapore,
1.2897,103.8501
161.117.191.87, update.intl.miui.com, SG, Singapore, Singapore,
1.2897,103.8501
161.117.71.156, global.market.xiaomi.com, SG, Singapore, Singapore,
1.2897,103.8501
47.88.222.244, data.mistat.intl.xiaomi.com, SG, Singapore, Singapore,
1.2897,103.8501
74.125.193.106, www.google.com, IE, Leinster, Dublin, 53.3331,-6.2489
209.85.203.95, www.googleapis.com, US, California, Mountain View,
38.0088,-122.1175
47.241.21.203, api.ad.intl.xiaomi.com, SG, Singapore, Singapore,
1.2897,103.8501
161.117.179.99, api.ad.intl.xiaomi.com, SG, Singapore, Singapore,
1.2897,103.8501
161.117.71.92, data.mistat.intl.xiaomi.com, SG, Singapore, Singapore,
1.2897,103.8501
209.85.203.154, www.googleadservices.com, US, California, Mountain
View, 38.0088,-122.1175
74.125.193.190, www.youtube.com, IE, Leinster, Dublin, 53.3331,-6.2489
209.85.202.95, play.googleapis.com, US, California, Mountain View,
38.0088,-122.1175
74.125.193.157, www.googleadservices.com, IE, Leinster, Dublin,
53.3331,-6.2489
209.85.203.101, android.clients.google.com, US, California, Mountain View,
38.0088,-122.1175
74.125.168.6, r1---sn-q0cedn7s.gvt1.com, IE, Leinster, Dublin,
53.3331,-6.2489

C. Huawei Handset

80.158.20.103, grs.dbankcloud.com, DE, Hesse, Hofheim am Taunus,
50.0902,8.4493
80.158.19.121, query.hicloud.com, DE, North Rhine-Westphalia, Kln,
50.9333,6.9500
209.85.202.95, play.googleapis.com, US, California, Mountain View,
38.0088,-122.1175
80.158.6.93, grs.hicloud.com, DE, North Rhine-Westphalia, Kln,
50.9333,6.9500
74.125.193.94, connectivitycheck.gstatic.com, IE, Leinster, Dublin,
53.3331,-6.2489
74.125.193.95, www.googleapis.com, IE, Leinster, Dublin, 53.3331,-6.2489
74.125.193.100, android.clients.google.com, IE, Leinster, Dublin,
53.3331,-6.2489
173.194.129.231, r2---sn-q0c7rn76.gvt1.com, IE, Leinster, Dublin,
53.3331,-6.2489
74.125.193.190, android-safebrowsing.google.com, IE, Leinster, Dublin,
53.3331,-6.2489
74.125.193.157, www.googleadservices.com, IE, Leinster, Dublin,
53.3331,-6.2489
74.125.193.136, www.youtube.com, IE, Leinster, Dublin, 53.3331,-6.2489
74.125.193.138, app-measurement.com, IE, Leinster, Dublin,
53.3331,-6.2489

74.125.168.10, r5---sn-q0cedn7s.gvt1.com, IE, Leinster, Dublin,
53.3331,-6.2489
74.125.168.7, r2---sn-q0cedn7s.gvt1.com, IE, Leinster, Dublin,
53.3331,-6.2489
74.125.193.113, android.clients.google.com, IE, Leinster, Dublin,
53.3331,-6.2489
74.125.193.101, app-measurement.com, IE, Leinster, Dublin,
53.3331,-6.2489
5.62.40.178, auth.ff.avast.com, DE, Hesse, Frankfurt am Main,
50.1107,8.6730
5.62.40.189, apkrep.ff.avast.com, DE, Hesse, Frankfurt am Main,
50.1107,8.6730
69.94.77.204, analytics.ff.avast.com, GB, England, London,
51.5085,-0.1257
173.194.129.233, r4---sn-q0c7rn76.gvt1.com, IE, Leinster, Dublin,
53.3331,-6.2489
74.125.193.99, www.google.com, IE, Leinster, Dublin, 53.3331,-6.2489
74.125.193.103, www.google.com, IE, Leinster, Dublin, 53.3331,-6.2489
74.125.193.104, www.google.com, IE, Leinster, Dublin, 53.3331,-6.2489
160.44.207.113, servicesupport.hicloud.com, DE, Baden-Wrttemberg,
Friedrichshafen, 47.6569,9.4755
172.253.116.95, play.googleapis.com, IE, Leinster, Dublin, 53.3331,-6.2489
209.85.203.94, connectivitycheck.gstatic.com, US, California, Mountain
View, 38.0088,-122.1175
209.85.203.95, play.googleapis.com, US, California, Mountain View,
38.0088,-122.1175
173.194.129.230, r1---sn-q0c7rn76.gvt1.com, IE, Leinster, Dublin,
53.3331,-6.2489
173.194.129.232, r3---sn-q0c7rn76.gvt1.com, IE, Leinster, Dublin,
53.3331,-6.2489
74.125.168.8, r3---sn-q0cedn7s.gvt1.com, IE, Leinster, Dublin,
53.3331,-6.2489
74.125.193.106, www.google.com, IE, Leinster, Dublin, 53.3331,-6.2489
74.125.193.147, www.google.com, IE, Leinster, Dublin, 53.3331,-6.2489

D. Realme Handset

74.125.193.95, play.googleapis.com, IE, Leinster, Dublin, 53.3331,-6.2489
74.125.193.103, www.google.com, IE, Leinster, Dublin, 53.3331,-6.2489
209.85.203.139, android.clients.google.com, US, California, Mountain View,
38.0088,-122.1175
209.85.203.105, www.google.com, US, California, Mountain View,
38.0088,-122.1175
74.125.193.97, ssl.google-analytics.com, IE, Leinster, Dublin,
53.3331,-6.2489
74.125.193.102, android.clients.google.com, IE, Leinster, Dublin,
53.3331,-6.2489
74.125.193.84, accounts.google.com, IE, Leinster, Dublin, 53.3331,-6.2489
74.125.168.7, r2---sn-q0cedn7s.gvt1.com, IE, Leinster, Dublin,
53.3331,-6.2489
173.194.129.231, r2---sn-q0c7rn76.gvt1.com, IE, Leinster, Dublin,
53.3331,-6.2489
74.125.193.94, update.googleapis.com, IE, Leinster, Dublin,
53.3331,-6.2489
142.250.34.2, edgedl.gvt1.com, US, California, Mountain View,
38.0088,-122.1175
74.125.193.100, redirector.gvt1.com, IE, Leinster, Dublin, 53.3331,-6.2489
74.125.168.8, r3---sn-q0cedn7s.gvt1.com, IE, Leinster, Dublin,
53.3331,-6.2489
74.125.168.9, r4---sn-q0cedn7s.gvt1.com, IE, Leinster, Dublin,
53.3331,-6.2489
74.125.168.10, r5---sn-q0cedn7s.gvt1.com, IE, Leinster, Dublin,
53.3331,-6.2489
74.125.168.6, r1---sn-q0cedn7s.gvt1.com, IE, Leinster, Dublin,
53.3331,-6.2489
172.253.116.154, pagead2.googlesyndication.com, IE, Leinster, Dublin,
53.3331,-6.2489
74.125.193.101, app-measurement.com, IE, Leinster, Dublin,
53.3331,-6.2489
74.125.193.155, www.googleadservices.com, IE, Leinster, Dublin,
53.3331,-6.2489
74.125.193.93, www.youtube.com, IE, Leinster, Dublin, 53.3331,-6.2489
35.180.239.129, classify-eu.apps.coloros.com, FR, le-de-France, Paris,
48.8534,2.3488
13.36.142.16, icosas-eu.coloros.com, FR, le-de-France, Paris,
48.8534,2.3488

173.194.129.234, r5---sn-q0c7rn76.gvt1.com, IE, Leinster, Dublin,
 53.3331,-6.2489
 173.194.129.233, r4---sn-q0c7rn76.gvt1.com, IE, Leinster, Dublin,
 53.3331,-6.2489
 173.194.129.232, r3---sn-q0c7rn76.gvt1.com, IE, Leinster, Dublin,
 53.3331,-6.2489
 173.194.129.230, r1---sn-q0c7rn76.gvt1.com, IE, Leinster, Dublin,
 53.3331,-6.2489
 13.36.123.156, icosu-eu.coloros.com, FR, le-de-France, Paris,
 48.8534,2.3488
 74.125.90.106, growth-pa.googleapis.com, US, California, Mountain View,
 38.0088,-122.1175
 74.125.193.104, www.google.com, IE, Leinster, Dublin, 53.3331,-6.2489
 74.125.193.119, i.ytimg.com, IE, Leinster, Dublin, 53.3331,-6.2489
 74.125.193.136, dl.google.com, IE, Leinster, Dublin, 53.3331,-6.2489
 74.125.193.154, www.googleadservices.com, IE, Leinster, Dublin,
 53.3331,-6.2489
 74.125.193.99, www.google.com, IE, Leinster, Dublin, 53.3331,-6.2489
 209.85.203.99, www.google.com, US, California, Mountain View,
 38.0088,-122.1175
 15.237.166.135, omes-sec.heyta mobile.com, FR, le-de-France, Paris,
 48.8534,2.3488
 13.36.103.12, languagef-eu.coloros.com, FR, le-de-France, Paris,
 48.8534,2.3488
 74.125.193.105, www.google.com, IE, Leinster, Dublin, 53.3331,-6.2489
 74.125.193.156, www.googleadservices.com, IE, Leinster, Dublin,
 53.3331,-6.2489
 209.85.203.95, play.googleapis.com, US, California, Mountain View,
 38.0088,-122.1175
 74.125.193.106, www.google.com, IE, Leinster, Dublin, 53.3331,-6.2489
 74.125.193.113, redirector.gvt1.com, IE, Leinster, Dublin, 53.3331,-6.2489
 209.85.202.119, i.ytimg.com, US, California, Mountain View,
 38.0088,-122.1175
 35.181.94.110, languagef-eu.coloros.com, FR, le-de-France, Paris,
 48.8534,2.3488
 35.180.20.161, ifota-eu.realmemobile.com, FR, le-de-France, Paris,
 48.8534,2.3488
 74.125.193.157, www.googleadservices.com, IE, Leinster, Dublin,
 53.3331,-6.2489
 209.85.202.94, connectivitycheck.gstatic.com, US, California, Mountain
 View, 38.0088,-122.1175
 172.253.116.97, ssl.google-analytics.com, IE, Leinster, Dublin,
 53.3331,-6.2489
 209.85.203.119, i.ytimg.com, US, California, Mountain View,
 38.0088,-122.1175
 74.125.193.190, dl.google.com, IE, Leinster, Dublin, 53.3331,-6.2489
 35.181.165.253, shorueux.push.heyta mobile.com, FR, le-de-France, Paris,
 48.8534,2.3488
 15.188.169.134, dceux.push.heyta mobile.com, FR, le-de-France, Paris,
 48.8534,2.3488
 35.180.155.130, dceux.push.heyta mobile.com, FR, le-de-France, Paris,
 48.8534,2.3488
